# TMPRSS2, a SARS-CoV-2 internalization protease is downregulated in head and neck cancer patients

**DOI:** 10.1186/s13046-020-01708-6

**Published:** 2020-09-23

**Authors:** Andrea Sacconi, Sara Donzelli, Claudio Pulito, Stefano Ferrero, Francesca Spinella, Aldo Morrone, Marta Rigoni, Fulvia Pimpinelli, Fabrizio Ensoli, Giuseppe Sanguineti, Raul Pellini, Nishant Agrawal, Evgeny Izumchenko, Gennaro Ciliberto, Aldo Giannì, Paola Muti, Sabrina Strano, Giovanni Blandino

**Affiliations:** 1 UOSD Clinical Trial Center, Biostatistics and Bioinformatics, IRCCS Regina Elena National Cancer Institute, Rome, Italy; 2 Oncogenomic and Epigenetic Unit, IRCCS Regina Elena National Cancer Institute, Rome, Italy; 3 Department of Biomedical, Surgical and Dental Sciences, University of Milan La Statale, Milan, Italy; 4 Fondazione IRCCS Ca’ Granda Ospedale Maggiore Policlinico Milano, Milan, Italy; 5 GENOMA-Molecular Genetics Laboratory, Rome, Italy; 6 Scientific Direction, San Gallicano Dermatological Institute IRCCS, Rome, Italy; 7 Department of Industrial Engineering, University of Trento, Trento, Italy; 8 Clinical Pathology and Microbiology, San Gallicano Dermatologic Institute IRCCS, Rome, Italy; 9 Radiotherapy Unit, IRCCS Regina Elena National Cancer Institute, Rome, Italy; 10 Otolaryngology Unit, IRCCS Regina Elena National Cancer Institute, Rome, Italy; 11 Department of Surgery, University of Chicago Medicine and Biological Sciences, Chicago, IL USA; 12 Department of Medicine, University of Chicago Medicine and Biological Sciences, Chicago, IL USA; 13 Scientific Direction, IRCCS Regina Elena National Cancer Institute, Rome, Italy; 14 SAFU Unit, IRCCS Regina Elena National Cancer Institute, Rome, Italy

**Keywords:** SARS-CoV-2, TMPRSS2, HNSCC, microRNAs TP53, MYC

## Abstract

**Background:**

SARS-coronavirus-2 enters host cells through binding of the Spike protein to ACE2 receptor and subsequent S priming by the TMPRSS2 protease. We aim to assess differences in both ACE2 and TMPRSS2 expression in normal tissues from oral cavity, pharynx, larynx and lung tissues as well as neoplastic tissues from the same areas.

**Methods:**

The study has been conducted using the TCGA and the Regina Elena Institute databases and validated by experimental model in HNSCC cells. We also included data from one COVID19 patient who went under surgery for HNSCC.

**Results:**

TMPRSS2 expression in HNSCC was significantly reduced compared to the normal tissues. It was more evident in women than in men, in TP53 mutated versus wild TP53 tumors, in HPV negative patients compared to HPV positive counterparts. Functionally, we modeled the multivariate effect of TP53, HPV, and other inherent variables on TMPRSS2. All variables had a statistically significant independent effect on TMPRSS2. In particular, in tumor tissues, HPV negative, TP53 mutated status and elevated TP53-dependent Myc-target genes were associated with low TMPRSS2 expression. The further analysis of both TCGA and our institutional HNSCC datasets identified a signature anti-correlated to TMPRSS2. As proof-of-principle we also validated the anti-correlation between microRNAs and TMPRSS2 expression in a SARS-CoV-2 positive HNSCC patient tissues Finally, we did not find TMPRSS2 promoter methylation.

**Conclusions:**

Collectively, these findings suggest that tumoral tissues, herein exemplified by HNSCC and lung cancers might be more resistant to SARS-CoV-2 infection due to reduced expression of TMPRSS2. These observations may help to better assess the frailty of SARS-CoV-2 positive cancer patients.

## Background

Unlike other members of the *Coronaviridae* that circulate in the human population and cause only mild respiratory disease, severe acute respiratory syndrome coronavirus 2 (SARS-CoV-2) is a novel betacoronavirus which is transmitted from animals to humans and severely affects pulmonary respiration [1–5]. SARS-CoV-2 enters host cells through the binding of Spike protein to ACE2 receptor and subsequent S protein priming performed by host proteases including TPMRSS2 [6–8].

HNSCC is the sixth leading cancer by incidence worldwide and the eighth most common cause of cancer death [9, 10]. Although in the past two decades new surgical and medical treatments have improved patients’ quality of life, the 5-year survival remains 40–50% of patients [11]. HNSCC is typically characterized by a high incidence of local recurrences, which are the most common cause of death in HNSCC patients, occurring in 60% of the cases [12]. The current standard therapies are surgical and systemic treatment followed by adjuvant radiotherapy (RT) with or without chemotherapy. Unfortunately, advances in treatments for HNSCC over the past two decades failed to substantially improve the overall disease outcome, with radio and chemo-resistance (intrinsic or acquired) remains one of the major challenges in the current therapy of HNSCC.

Here we sought to investigate the expression of both ACE2 and TMPRSS2 in head and neck cancer specimens. We found that unlike ACE2, whose expression was unchanged in HNSCC patients, TPMRSS2 expression was significantly reduced in tumor tissues compared to non-tumorous ones in both HNSCC TCGA and IRE datasets. Notably, TMPRSS2 downregulation associated with poorer survival in HNSCC patients with TP53 mutations, HPV negative status, aberrant MYC activation and low immune signature. Mechanistically, downregulation of TMPRSS2 significantly correlated with aberrant upregulation of specific microRNAs, which might target TMPRSS2 post-transcriptionally, thereby leading to its reduced expression levels in tumor tissues. microRNAs are an abundant class of small noncoding RNAs of approximately 22 nucleotides long. They act as negative regulators of gene expression at the post-transcriptional level, by binding their target mRNAs through imperfect base pairing with the respective 3′-untranslated region (3′-UTR). Deregulation of microRNAs leads to an altered expression of genes involved in many cell functions and cell fate regulation. Therefore, by regulating genes and pathways, microRNAs could contribute to modulate biological functions including potential effect on epithelial cell interaction with viruses and tumorigenesis [13, 14]. We found that the expression of a group of microRNAs (miR-193b-3p; miR-503-5p; miR-455-5p; miR-31-3p; miR-193b-5p; miR-2355-5p) was anti-correlated to that of their target TMPRSS2. This anti-correlated expression was also evidenced in an HNSCC patient positive for SARS-CoV-2 infection.

## Methods

The study has been conducted using the TCGA and the Regina Elena Institute (IRE) databases and validated by experimental model in HNSCC and Lung cancer cells. We also included data from one COVID19 patients who underwent surgery for HNSCC.

The ethical committee of the Regina Elena National Cancer Institute and of University of Milan approved the study.

### Bioinformatic analysis

#### Gene expression analysis

Analysis of 23 matched tumor and normal samples with gene expression profile from Affymetrix platform were background adjusted and quantile normalized. The gene expression values were obtained by using Robust Multiple-array Average (RMA) procedure.

MiRNAs expression for 66 matched tumor and normal samples from Agilent platform were analyzed as described in Ganci et al. [15].

mRNA expression data from IRE cohort used during the current study has been deposited in NCBI’s Gene Expression Omnibus and is accessible through GEO series accession number GSE107591 (https://www.ncbi.nlm.nih.gov/geo/query/acc.cgi?acc=GSE107591).

Normalized TCGA HNSC gene expression and miRNA expression of 478 tumor samples and 44 normal samples were obtained from Broad Institute TCGA Genome Data Analysis Center (http://gdac.broadinstitute.org/): Firehose stddata__2016_01_28. Broad Institute of MIT and Harvard. doi:10.7908/C11G0KM9

Significance of miRNA and gene modulation between expression values of normal and tumor samples was assessed by two-side paired or unpaired Student’s test and ANOVA test was used for comparisons among more than two groups. Significance was defined at the *p* < 0.05 level.

A generalized linear model was fitted to evaluate linear regression of TMPRSS2 with immune signature, MYC-dependent gene signatures and clinical variables.

TMPRSS2 gene and protein expression data in normal tissues were obtained from EMBL-EBI Expression Atlas public repository (https://www.ebi.ac.uk/gxa/home).

#### Methylation analysis

DNA methylation data of TCGA casuistry were obtained from Wanderer (http://maplab.imppc.org/wanderer/).

#### MiRNA target and pathway analysis

We used miRWalk (http://mirwalk.umm.uni-heidelberg.de/) and miRNet (https://www.mirnet.ca/miRNet/home.xhtml) web tools for miRNA-target interaction prediction and pathway enrichment analysis. Spearman’s correlation coefficient was used to establish significance of negative association on patient samples for each predicted interaction.

#### Survival analysis

Disease-free survival (DFS) and overall survival (OS) were performed by using Kaplan-Meier analysis and the log-rank test was used to assess differences between curves. Patients with high and low signal intensity were defined by considering positive and negative z-score values, if not differently specified.

The correlation and regression analyses as well as the miRNA and gene modulation and the survival analysis were completely conducted with Matlab R2019.

### Cell cultures

Cal-27 (mutp53H193L) and Detroit-562 (mutp53R175H) cell lines were obtained from ATCC.

Cells were cultured in RPMI1640 (Cal27) and DMEM (Detroit 562) medium (Invitrogen-GIBCO) supplemented with 10% FBS, penicillin (100 U/mL), and streptomycin (100 mg/mL; Invitrogen-GIBCO). All cell lines were grown at 37 °C in a balanced air humidified incubator with 5% CO2.

### Cell transfection

The transfections were performed with Lipofectamine RNAiMax (Life Technologies). All experiments were conducted according to the manufacturer’s recommendations. siRNAs were purchased from Eurofins MWG and sequences are as follows: si-SCR: 5′-AAGUUCAGCGUGUCCGGGGAG-3′; si-MYC: 5’GCCACAGCAUACAUCCUGU-3′; si-YAP: 5′-GACAUCUUCUGGUCAGAGA-3′; Si-p53: 5′-GACUCCAGUGGUAAUCUAC-3′. The cells were transfected for 48–72 h according to the cell line and the experiments.

### RNA extraction and expression analysis

Total RNA from cells was extracted using the TRIzol Reagents (GIBCO) following the manufacturer’s instructions. RNA from FFPE samples was extracted using the miRneasy FFPE kit (QIAGEN) following the manufacturer’s instructions. The concentration and purity of total RNA was assessed using a Nanodrop TM1000 spectrophotometer (Nanodrop Technologies). Reverse transcription and qRT-PCR quantification were performed, respectively, by MMLV RT assay and SYBR Green or Taqman assays (Applied Biosystems) according to the manufacturer’s protocol. GAPDH and RNU48 were used as endogenous controls to standardize gene expression. Primers and Taqman assays used are indicated in supplementary Table 2.

### SARS-CoV-2 detection

For the detection of SARS-CoV-2 in RNAs extracted from tissue samples we used Bosphore Novel Coronavirus (2019-nCoV) Detection Kit v2 (Anatolia GeneWork). This kit is a Real-Time PCR-based in vitro diagnostic medical device that allows to detect two regions of the virus in two separate reactions: E gene is used for screening purpose, where 2019-nCoV and also the closely related coronaviruses are detected, and the orf1ab target region is used to discriminate 2019-nCoV specifically. This kit includes also an internal control in order to check RNA extraction, PCR inhibition and application errors.

### Targeted DNA NGS

Genomic DNA was extracted on the QIAcube® platform using the QIAamp DNA FFPE tissue kit (Qiagen) according to the manufacturer’s instructions. All DNA samples were then quantified by a Qubit Fluorometer (Termofisher Scientific, Waltham, Massachusetts, USA) using a Qubit® dsDNA HS Assay Kit. Library preparation was performed on 10 ng DNA by the Ion AmpliSeq Library Kit 2.0 (Termofisher Scientific) and the Colon and Lung Panel (Life Technologies) was used to sequencing the entire coding regions of TP53, as previously described [16]. Briefly, the prepared libraries were sequenced on Ion S5 Sequencer using an Ion 540 Chip and an Ion 540 kit–Chef (all Thermo Fisher Scientific) with configuration 500 flows covering the 200 bp library read length. Raw data were analyzed using the Torrent Mapping Alignment Program aligner implemented in v5.2 of the Torrent Suite software (Thermo Fisher Scientific). All NGS variants were manually reviewed with Integrative Genomics Viewer (IGV version 2.2, Broad Institute, Cambridge, MA, USA) and Biomedical Genomics Workbench Version 4.0 (Qiagen), and then matched against the ClinVar (https://www.ncbi.nlm.nih.gov/clinvar/) and COSMIC (https://cancer.sanger.ac.uk/cosmic) databases.

### Immunohistochemistry

Two paraffin blocks of the tumour under study were selected and 2 sections, 3 μm in thickness were cut. P 53 immunohistochemical analysis were performed via DO-7 Clone (DAKO) on Dako Omnis platform. Reactions were revealed using UltraView Universal DAB [17].

## Results

### ACE2 and TMPRSS2 expression in HNSCC patients

**Fig. 1:**
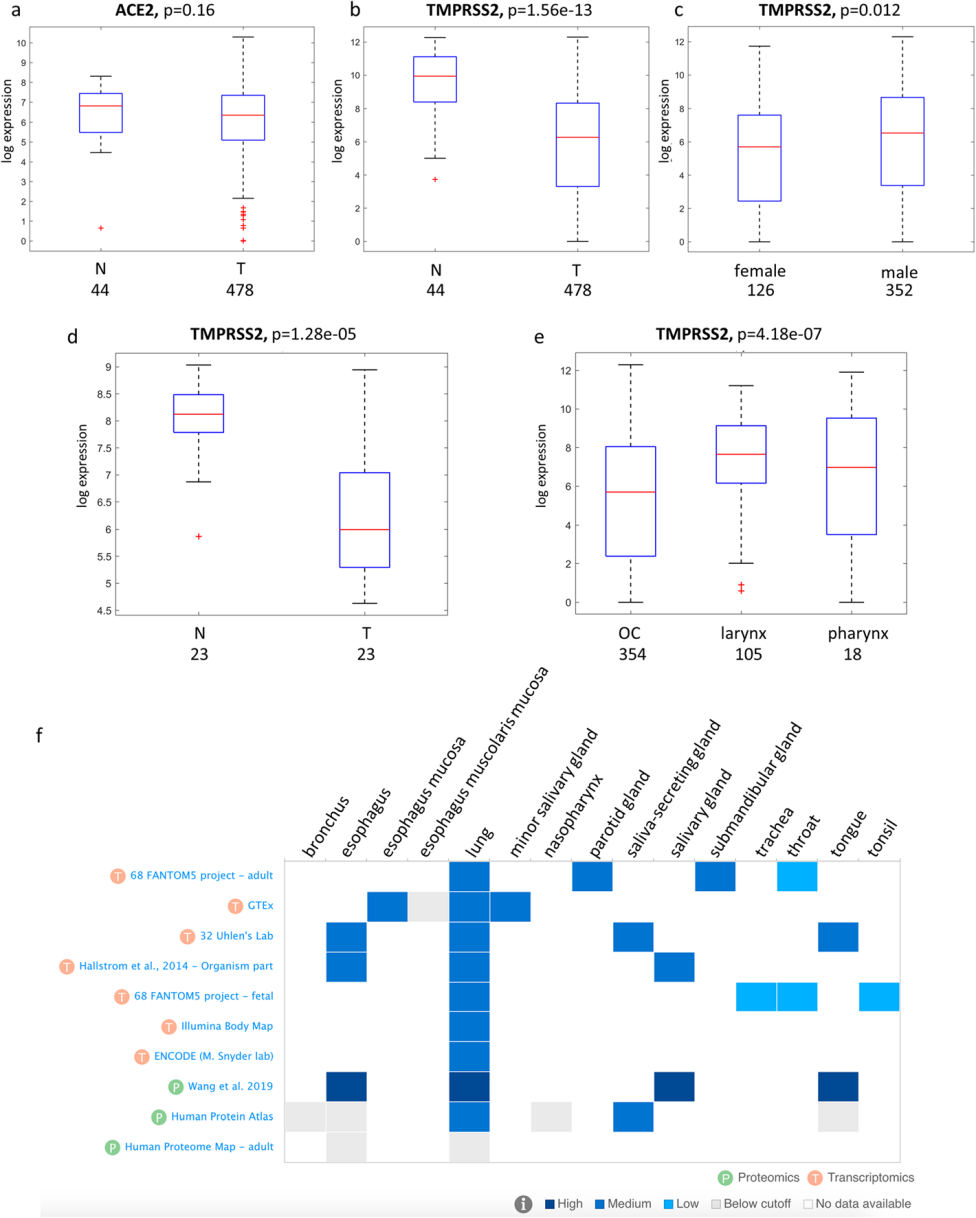
Distribution of ACE2 and TMPRSS2 gene expression in HNSCC patients. (a-b) Box-plot analysis representing ACE2 (**a**) and TMPRSS2 (**b**) gene expression levels in non-tumorous (N) and tumor (T) tissues from the HNSCC TCGA dataset. **c** Box-plot analysis representing TMPRSS2 gene expression levels in tumoral HNSCC TCGA samples according to the gender (female or male). **d** Box-plot analysis representing TMPRSS2 gene expression levels in non-tumorous (N) and tumor (T) tissues from the HNSCC cohort of IRCSS Regina Elena National Cancer Institute of Rome. **e** Box-plot analysis representing TMPRSS2 gene expression levels in tumoral HNSCC TCGA samples according to anatomical site. **f** Expression analysis of TMPRSS2 in the selected normal tissues and in the indicated studies, by using EMBL-EBI Expression Atlas public repository. The analysis includes both transcriptomics (T) and proteomics (P) data

To test our hypothesis, we first assessed the expression of both ACE2 and TMPRSS2 in TCGA HNSCC dataset. We found that while ACE2 expression level was comparable between non-tumorous versus malignant tissues, the TMPRSS2 expression was significantly downregulated in HNSCC patient samples (Fig. 1a-b). Interestingly, while female patients showed a more pronounced downregulation of TMPRSS2 than males (Fig. 1c), the level of ACE2 expression in female patients was upregulated (Suppl. Fig. 1a).

To validate these observations, we have assessed the expression of TMPRSS2 in an additional cohort of HNSCC patients enrolled at Regina Elena Cancer Institute [15]. This cohort includes naïve HNSCC patients for which tumor and peritumoral tissues and resection margin specimens are available [18]. Confirming our findings, TMPRSS2 expression was significantly reduced in tumor samples compared to non-tumorous tissues (Fig. 1d). Interestingly, expression of TMPRSS2 was significantly higher in tumors from larynx and pharynx compared to malignancies of the oral cavity (Fig. 1e). In non-tumorous tissues from either TCGA or IRE datasets no correlation between the TPMRSS2 expression and sex or histological site (oral cavity, larynx and pharynx) was detected (Suppl. Fig. 1b).

To further assess the pattern of TMPRSS2 expression in normal tissues, we have used ATLAS, and showed data of transcript and protein expression of different tissue sites from which HNSCC develops (Fig. 1f). Lung tissue was included as a reference, as high expression of TMPRSS2 was reported in lung by RNA-Seq and proteomic analyses conducted by several studies (Fig. 1f). A widespread expression of TMPRSS2 was evidenced in lung and head neck tissues (Fig. 1f).

### TMPRSS2 expression is prognostic and associates with TP53 mutations and HPV status in HNSCC patients

**Fig. 2:**
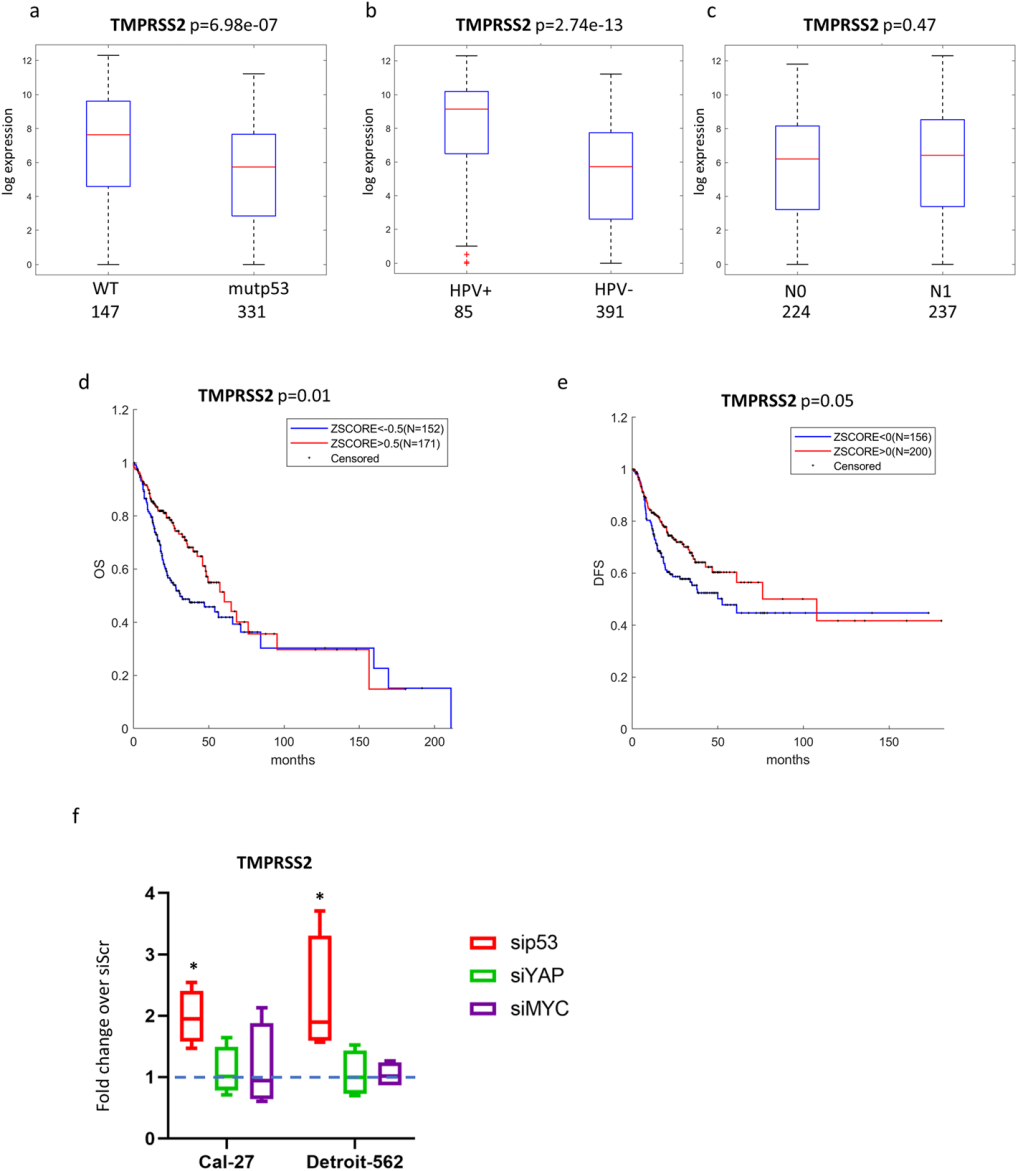
TMPRSS2 gene expression, HNSCC patient clinical variables and correlation with survival. **a**-**c** Box-plot analysis representing TMPRSS2 gene expression levels in tumoral HNSCC TCGA samples according to TP53 status (**a**), or HPV status (**b**), or N status (**c**). **d**-**e** Kaplan–Meier survival curves for TCGA HNSCC patients showing overall survival (OS) (**d**) and disease-free survival (DFS) (**e**), according to TMPRSS2 gene expression. **f** qRT-PCR analysis of TMPRSS2 expression levels in Cal-27 and Detroit-562 cell lines upon depletion of mutantp53 (sip53) or YAP (siYAP) or MYC (siMYC) compared to cells transduced with scramble molecules (value = 1). *p-value < 0.05

As for many human cancers, TP53 is the most frequently mutated gene in HNSCC [9, 19]. In TCGA dataset, which includes 478 HNSCC molecularly well characterized cases we found that patients carrying TP53 mutation exhibited a significantly lower level of TMPRSS2 expression compared to the patients with intact TP53 gene (Fig. 2a).

Since the vast majority of TP53 mutated patients are HPV negative, we next looked at HPV negative cohort independently, and found that TMPRSS2 expression was substantially lower in HPV negative patients than in HPV positive ones (Fig. 2b). TMPRSS2 expression did not vary accordingly to N status (Fig. 2c). As it was previously reported that patients with HNSCC HPV negative and TP53 mutated cancer exhibit shorter overall survival (OS) and disease free survival (DFS) [20], we next performed a Kaplan Meyer analysis on data obtained from TCGA database, which revealed that lower TMPRSS2 expression associated with shorter OS and DFS in HNSCC patients (Fig. 2d-e).

We have next analysed the role of TMPRSS2 using two HNSCC cell lines (Cal-27 and Detroit-562) carrying TP53 mutations that exert gain of function activities. When p53 protein in these cell lines was depleted, expression of TMPRSS2 transcript was significantly up-regulated (Fig. 2f), suggesting that mutant p53 oncogenic protein may regulate (either directly or indirectly) TMPRSS2 expression in HNSCC cell lines. We also analysed Cal-27, and Detroit-562 cell lines depleted for YAP and MYC, two important co-factors of transcriptional activity of gain of function mutant p53 proteins [21]. Unlike mutant p53, neither YAP nor MYC depletion did not affect the TMPRSS2 level in these cell lines (Fig. 2f). ACE2 expression was unaffected by mutant p53, YAP and MYC depletion in both HNSCC cell lines (Suppl. Fig. 2a). Consistently, while the expression of TMPRSS2 was unaffected upon wt-p53 protein depletion that of ACE was only slightly modulated (Suppl. Fig. 2b-c). In summary, these observations indicate that low expression of TMPRSS2, in a context of TP53 mutations and HPV negative status is associated with poor prognosis in HNSCC patients.

### TMPRSS2 expression is associated with aberrant MYC activity and mutant p53 in HNSCC patients

**Fig. 3:**
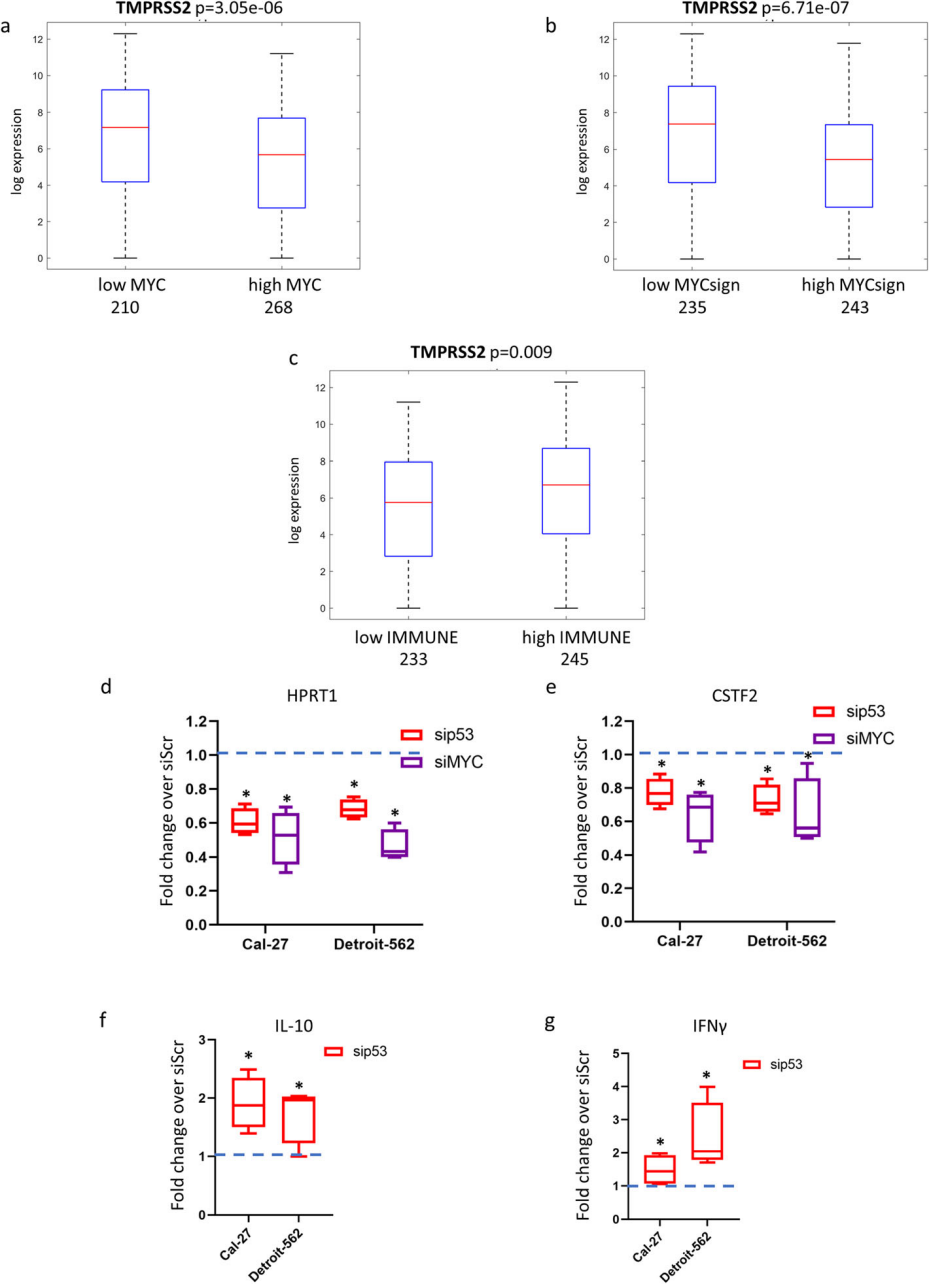
TMPRSS2 gene association with MYC signature and immune signature in HNSCC patients. **a** Box-plot analysis representing TMPRSS2 gene expression levels in tumoral HNSCC TCGA samples according to low or high expression values of MYC gene. **b** Box-plot analysis representing TMPRSS2 gene expression levels in tumoral HNSCC TCGA samples according to low or high expression values of 22-gene MYC signature. **c** Box-plot analysis representing TMPRSS2 gene expression levels in tumoral HNSCC TCGA samples according to low or high expression values of 17-gene immune signature. **d**-**e** qRT-PCR analysis of HPRT1 (**d**) and CSTF2 (**e**) expression levels in Cal-27 and Detroit-562 cell lines upon depletion of mutant p53 (sip53) or MYC (siMYC) compared to scramble control cells (value = 1). (f, g) qRT-PCR analysis of IL-10 (f) and IFNγ (g) expression levels in Cal-27 and Detroit-562 cell lines upon depletion of mutant p53 (sip53) or MYC (siMYC) compared to scramble control cells (value = 1). *p-value < 0.05

MYC is a proto-oncogene that plays a crucial role in different steps of tumorigenesis [22]. In HNSCC, aberrant MYC expression is associated with poor survival [23]. In our study, at univariate levels we found that low TMPRSS2 expression was significantly associated with high levels of MYC in TCGA HNSCC patients (Fig. 3a).

We have recently reported that MYC is a pivotal mediator of gain of function mutant p53 signalling in HNSCC [24]. We have identified a mutantp53/MYC dependent signature whose aberrant activation (high expression levels) associated with shorter overall survival in HNSCC patients (Suppl. Fig. 3a) [24].

Notably, low expression of TMPRSS2 negatively associated with the high level of this previously reported 22-gene mutant p53/MYC signature (Fig. 3b).

It has been extensively reported that mutant p53 proteins actively promote resistance to therapies, for example by inhibiting the onset of apoptosis [25]. However, failure of patients to respond to anti-cancer therapies is also dependent on blockade of effector immune cell function by tumor cells (immune evasion) [26]. It was reported that mutant p53 is associated with the inability of the immune system to recognize neoantigens, which should be abundant in these tumors characterized by high mutational burden [27]. To explore whether mutant p53 is the driver of immune suppression in HNSCC, we evaluated the association between TP53 status and immune-related signatures using the TCGA dataset of HNSCC. In this study we have used an immune signature reported by Wood et al., due to its high stability in HNSCC specimens (Suppl. Fig. 3b) [28]. We found that low expression of TMPRSS2 significantly associates with low immune signature in TCGA HNSCC patients’ cohort (Fig. 3c). The transcriptional crosstalk between mutant p53, MYC/MYC signature and immune signature was assessed in two HNSCC cell lines, Cal-27 and Detroit-562 cells, As expected, both mutant p53 and MYC depletion reduced the expression of two MYC target genes, HPRT1 and CSTF2 (Fig. 3d-e). Interestingly, depletion of mutant p53 protein increased the expression of both IL-10 and IFNγ transcripts in Cal-27 and Detroit-562 cell lines (Fig. 3f-g).

We next conducted a linear regression analysis which includes the immune signature expression, HPV and TP53 status as well as MYC signature, to assess whether addition of the immune signature could improve the total variance seen in the model which did not include the immune signature.

We subsequently assessed the effect of MYC, MYC signature, HPV status (positive and negative), the wild-type and mutated TP53 and immune-signature on TMPRSS2 expression at univariate and multivariate levels. The univariate analysis showed that all the variables were able to significantly and independently modulate TMPRSS2 expression (Table 1, upper panel). When the same variables were included in one logic regression model, beside the immune signature, all other variables have significantly and independently contributed to the TMPRSS2 expression with a 15% of its total variance explained (Table 1, lower panel). In general, these findings reveal the association between TMPRSS2 downregulation with mutant p53 and MYC oncogenic activities in HNSCC patients, and indicate that the immune signature did not add any substantial effect when all other variables contribution was taken into consideration. The lack of independent effect of immune signature on TMPRSS2 expression could be related to the biological interdependence of the immune signature with TP53, which has a strong effect on inducing immune suppression [29].

**Table 1:** TMPRSS2 gene association with immune signature, MYC and MYC signature, HPV status and P53 mutation in HNSCC patients. Linear univariate and multivariate regression models were built from TCGA HNSCC patients considering TMPRSS2 as outcome variable. In the table is indicated the percentage of variance explained (R^2^) and the total *p*-value of the multivariate model

univariate					
**VAR**	**OR[CI95%]**	**p-value**	**beta**	**R**^**2**^	
**HPV**	15.77 [7.69–32.36]	2.74E-13	2.76	0.11	
**mutp53**	0.21 [0.11–0.38]	6.98E-07	−1.57	0.05	
**signatureIMMUNO**	2.15 [1.21–3.83]	9.36E-03	0.77	0.01	
**signatureMYC**	0.23 [0.13–0.41]	6.71E-07	−1.45	0.05	
**MYC**	0.25 [0.14–0.45]	3.05E-06	−1.38	0.04	
multivariate					
**VAR**	**OR[CI95%]**	**p-value**	**beta**	**R**^**2**^	**model p-value**
**HPV**	7.70 [3.46–17.14]	7.83E-07	2.04	0.15	3.24E-15
**mutp53**	0.54 [0.28–1.05]	7.07E-02	−0.61		
**signatureIMMUNO**	1.11 [0.63–1.95]	7.07E-01	0.11		
**signatureMYC**	0.41 [0.23–0.74]	3.39E-03	−0.88		
**MYC**	0.53 [0.30–0.97]	3.89E-02	−0.62		

### Epigenetic control of TMPRSS2 expression in HNSCC

**Fig. 4:**
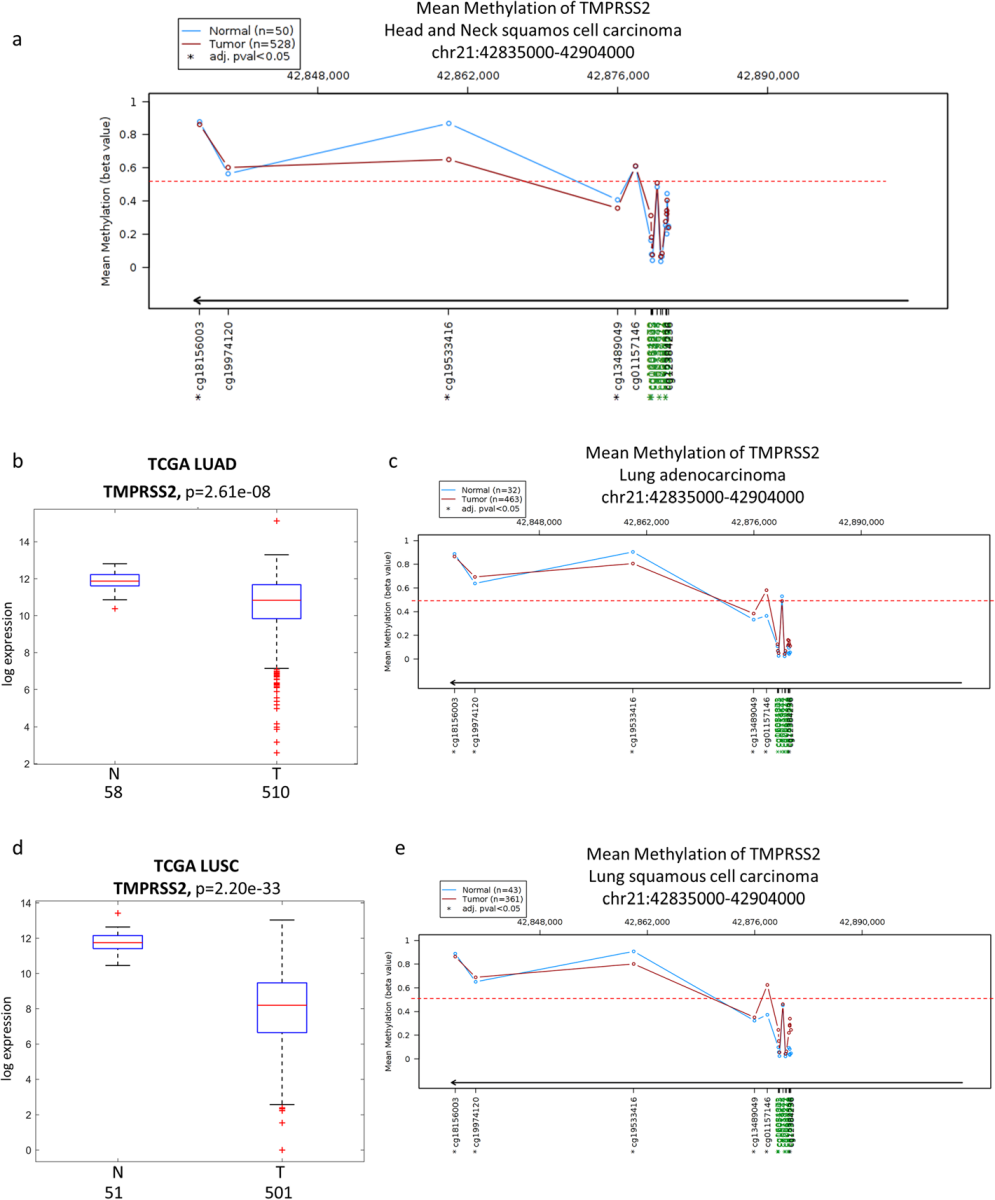
TMPRSS2 gene methylation status in normal and tumoral samples of HNSCC and lung cancer patients. **a** DNA methylation profile of TMPRSS2 gene in normal (blue line) and tumoral (red line) samples of HNSCC TCGA patients. **b** Box-plot analysis representing TMPRSS2 gene expression levels in non-tumorous (N) and tumor (T) tissues from the lung adenocarcinoma (LUAD) TCGA dataset. **c** DNA methylation profile of TMPRSS2 gene in normal (blue line) and tumoral (red line) samples of LUAD TCGA patients. **d** Box-plot analysis representing TMPRSS2 gene expression levels in non-tumorous (N) and tumor (T) tissues from the lung squamous cell carcinoma (LUSC) TCGA dataset. **e** DNA methylation profile of TMPRSS2 gene in normal (blue line) and tumoral (red line) samples of LUAD TCGA patients. DNA methylation profile has been performed by using Wanderer software. Beta values (β) are the estimate of methylation level using the ratio of intensities between methylated and unmethylated alleles. β values are between 0 and 1, with 0 being unmethylated and 1 fully methylated. The tool provides the detailed individual beta values of all the HumanMethylation450 probes inside or in the vicinity of the gene. Probes for the CpG island are indicated in green. Dotted red line indicates the threshold β value of 0.5

To further investigate the molecular mechanisms underlying TMPRSS2 downregulation in HNSCC we have analyzed the extent of TMPRSS2 promoter methylation. To this end, we used a Wanderer tool that includes data of methylation specific sequencing for cases in TCGA databases [30]. We compared the TMPRSS2 promoter methylation in tumor versus non-tumorous tissues in HNSCC TCGA dataset. As shown in Fig. 4a, the intensity ratio of methylated and unmethylated alleles (β value) was lower than 0.5, indicating that CpG islands either within or in the vicinity of TPMRSS2 promoter were unmethylated both in tumor and normal tissues. A similar unmethylated pattern for TMPRSS2 promoter was evidenced in both lung adenocarcinoma (LUAD) and lung squamous cell carcinoma (LUAS) specimens, where expression levels of TMPRSS2 were significantly downregulated (Fig. 4b-e).

**Fig. 5:**
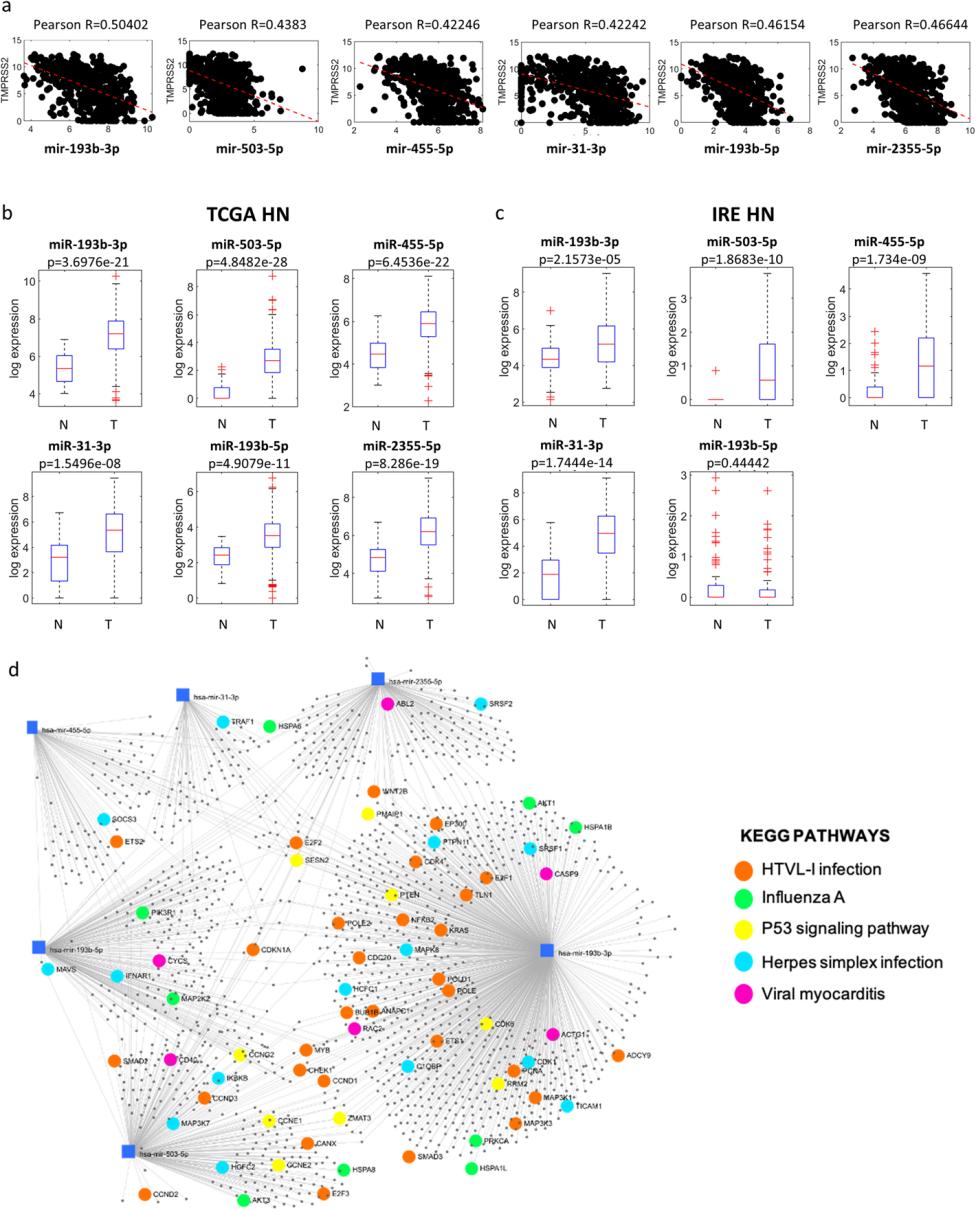
Expression levels of miRNAs predicted to target TMPRSS2 gene in HNSCC patients. **a** Graphs showing the correlation (Spearman coefficients) between indicated miRNAs and TMPRSS2 in the TCGA HNSCC dataset. **b** Box-plot analysis representing expression levels of miRNAs predicted to target TMPRSS2 in non-tumorous (N) and tumor (T) tissues from TCGA HNSCC patients. **c** Box-plot analysis representing expression levels of miRNAs predicted to target TMPRSS2 in non-tumorous (N), peri-tumor (PT) and tumor (T) tissues from IRCSS Regina Elena National Cancer Institute (IRE) HNSCC patients. **d** miRNA-centric network using the web tool miRNet. The network shows main validated miRNA-target interaction of the six selected miRNA signature. Genes involved in specific KEGG pathways are highlighted

We next investigated whether TMPRSS2 downregulation in HNSCC patients could be due to selective targeting by microRNAs. Using miRWalk tool we searched for microRNAs that could putatively target the TMPRSS2 (Suppl. Table 1). We have selected a number of microRNAs candidates (miR-193b-3p; miR-503-5p; miR-455-5p; miR-31-3p; miR-193b-5p; miR-2355-5p) whose expression levels were inversely correlated with Pearson R = 0.50402; 0.4383; R = 0.42242; R = 0.46154; R = 0.42246; R = 0.46644 compared to that of TMPRSS2 expression in TCGA HNSCC tumoral tissues (Fig. 5a). As a corollary information on the expression of the microRNA signature, we also observed that miR-193b-5p and e miR-193b-3p resulted to be inversely correlated to TMPRSS2 both in LUAD as LUSC, miR-31-3p resulted to be inversely correlated to TMPRSS2 in LUAD, miR-503-5p and miR-2355-5p resulted to be weakly inversely correlated to TMPRSS2 in LUAD (Suppl. Fig. 4a-b). Coherently, we found that all six selected microRNAs were significantly upregulated in tumoral tissues compared to the non-tumorous samples (Fig. 5b). This upregulation was confirmed in IRE cohort for all but miR-193-5p whose expression was unchanged and for miR-2355-5p that was not present on the arrays used to profile HNSCC matched tumor and non-tumorous samples (Fig. 5c). Interestingly, miR-193b-3p, miR-455-5p, miR-193b-5p and miR-2355-5p expression is significantly higher in HNSCC patients carrying TP53 mutations than those with intact TP53 gene.

In descriptive terms, we observed significant correlations between microRNA signature, or part of it, and HPV status, gender and tumor site. As matter of fact, miR-31-3p, miR-193b-5p, miR-193b-3p, miR-455-5p and miR-2355-5p were downregulated in HPV^+^ patients while the same microRNAs were upregulated in female patients compared to male ones. Finally, miR-193b-5p, miR-193b-3p, miR-455-5p, miR-503-5p and miR-2355-5p were significantly deregulated according to tumor site (Suppl. Fig. 6a-c).

miRNet online based tool was used to identify the potential targets of miR-193b-3p, miR-503-5p, miR-455-5p, miR-31-3p, miR-193b-5p and miR-2355-5p (Suppl. Table 2). Subsequently, the identified list of microRNA targets was assessed using KEGG pathway enrichment analysis to reveal cell signaling pathways impacted by the aberrant activities of the selected panel of microRNAs (Table 2). In Fig. 5d we reported those pathways whose microRNA validated targets impinged on viral infections and p53 pathway.

**Table 2:** KEGG enriched pathways evaluated from validated miRNA-target interactions (miRNet). Total number of genes included in each pathway and number of genes represented in each pathway (hits) with respective *p*-values are shown

Pathway	Total	Hits	Pvalue
Cell cycle	124	42	3.08E-15
Small cell lung cancer	80	22	9.85E-07
Prostate cancer	87	23	1.19E-06
Pathways in cancer	310	52	3.53E-06
Focal adhesion	200	38	4.18E-06
p53 signaling pathway	68	18	1.73E-05
Pancreatic cancer	69	18	2.15E-05
HTLV-I infection	199	36	2.37E-05
Chronic myeloid leukemia	73	18	4.90E-05
Colorectal cancer	49	14	6.03E-05
Melanoma	68	17	6.57E-05
Adherens junction	70	17	9.74E-05
Non-small cell lung cancer	52	14	1.23E-04
Neurotrophin signaling pathway	123	23	4.43E-04
Glioma	65	15	4.53E-04
One carbon pool by folate	19	7	8.40E-04
Toxoplasmosis	93	18	1.21E-03
Fanconi anemia pathway	39	10	1.72E-03
Renal cell carcinoma	60	13	1.99E-03
Selenocompound metabolism	12	5	2.59E-03
Oocyte meiosis	108	19	2.87E-03
Bladder cancer	29	8	3.02E-03
Acute myeloid leukemia	57	12	3.78E-03
Progesterone-mediated oocyte maturation	80	15	4.17E-03
Glyoxylate and dicarboxylate metabolism	19	6	4.85E-03
Regulation of actin cytoskeleton	182	27	5.39E-03
Pyrimidine metabolism	101	17	7.41E-03
ErbB signaling pathway	87	15	9.27E-03
Thyroid cancer	28	7	9.75E-03
MAPK signaling pathway	265	35	1.08E-02
Influenza A	107	17	1.31E-02
Endometrial cancer	44	9	1.40E-02
RNA transport	126	19	1.54E-02
Herpes simplex infection	103	16	1.93E-02
Protein processing in endoplasmic reticulum	129	19	1.94E-02
Glycine, serine and threonine metabolism	33	7	2.39E-02
Viral myocarditis	26	6	2.41E-02
Cysteine and methionine metabolism	34	7	2.79E-02
Jak-STAT signaling pathway	99	15	2.83E-02
mRNA surveillance pathway	82	13	2.85E-02
Epstein-Barr virus infection	91	14	2.97E-02
Valine, leucine and isoleucine degradation	44	8	3.84E-02
Pertussis	52	9	3.85E-02
mTOR signaling pathway	45	8	4.32E-02
Tight junction	118	16	5.85E-02

**Fig. 6:**
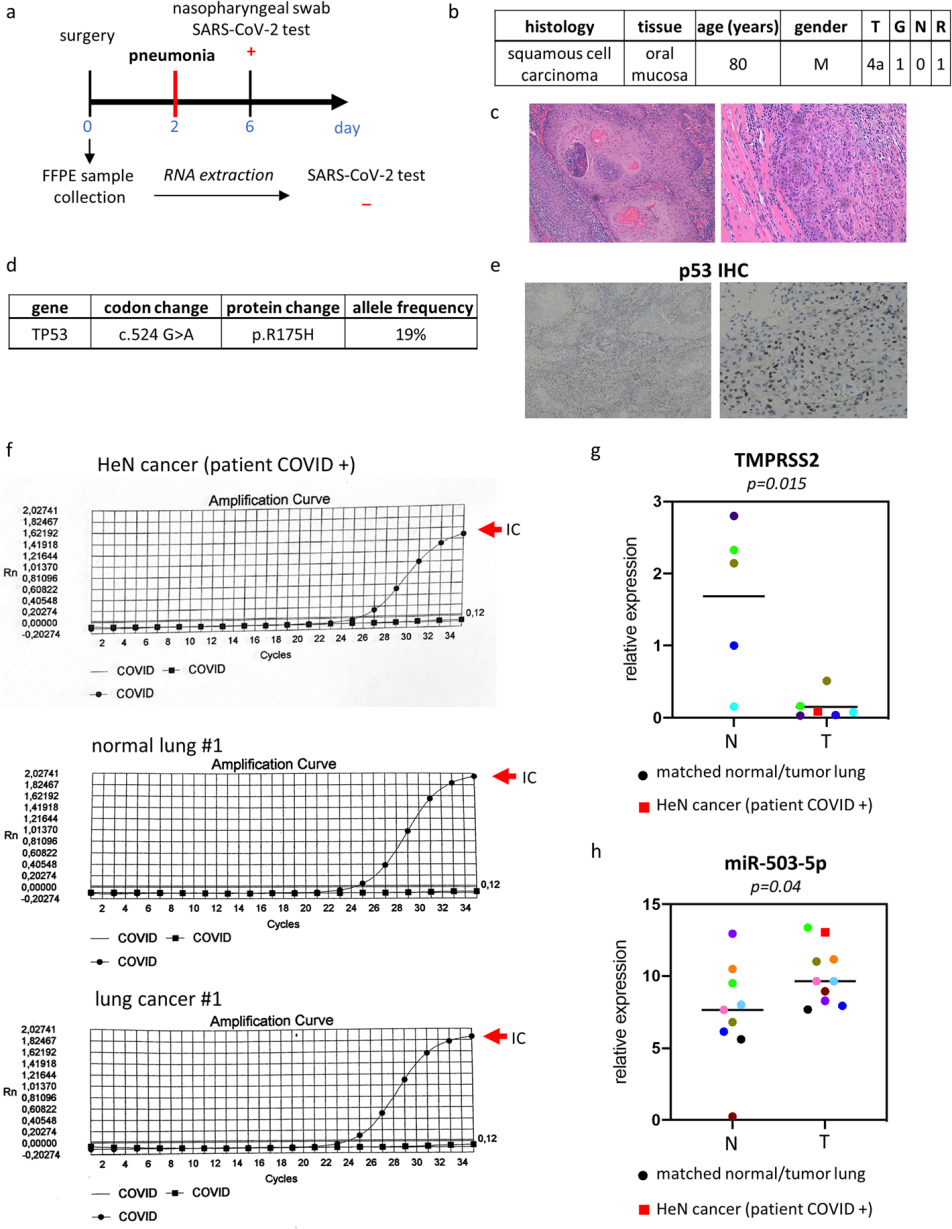
Case report: COVID-19 positive HNSCC patient. **a** Clinical history of COVID-19 positive HNSCC patient: at day 0 patient underwent surgical resection of the tumor that was formalin fixed and paraffin embedded. 2 days after surgery the patient developed pneumonia symptoms and nasopharyngeal swab SARS-CoV-2 test at day 6 was positive. SARS-CoV-2 test was also performed in FFPE tumoral tissue and resulted to be negative. **b** Clinical characteristics of COVID-19 positive HNSCC patient. **c** Well differentiated squamous cell carcinoma (G1) showing areas of keratinization with infiltration of muscular layer. **d** DNA sequencing results. **e** Immunostaining for p53 protein expression in COVID-19 positive HNSCC patient. **f** Real-time PCR curves from SARS-CoV-2 test run of RNA extracted from FFPE tumor tissue of HNSCC patient and from FFPE non-tumorous and tumor lung tissues of a lung cancer patient enrolled in 2011. The internal control was indicated by the red arrow. **g** qRT-PCR analysis of TMPRSS2 in 5 normal lung tissues (N) and 5 matched lung cancer tissues plus COVID-19 positive HNSCC tissue (T). **h** qRT-PCR analysis of miR-503-5p in 9 non-tumorous lung tissues (N) and 9 matched lung cancer tissues plus COVID-19 positive HNSCC tissue (T)

To further confirm the negative correlation between the level of TMPRSS2 transcript and microRNAs we assessed the expression of miR-503-5p and TMPRSS2 in an HNSCC patient positive to COVID-19. 80 years old male patient underwent surgery in February 2020 for resection of squamous cell carcinoma (T4aN1G0R1). Two days after surgery, the patient developed pneumonia symptoms and was found positive for SARS-CoV-2 infection by nasopharyngeal swab (Fig. 6a-c). Interestingly, the sequencing of entire coding region of TP53 revealed a mutation in codon 524 (G > A) that encoded for mutant p53-R175H protein (Fig. 6d). This finding was also confirmed by diffuse p53 staining as for mutant p53 proteins whose half-life is strongly increased (Fig. 6e).

Excised FPPE tumoral tissue was assessed for viral gene expression and found negative to SARS-CoV-2 infection (Fig. 6f, upper panel). Matched tumoral and non-tumorous FPPE specimens from a male lung cancer patient who underwent surgical resection in 2011 were used as a reference negative control and found negative for expression of SARS-CoV-2 target gene expression (Fig. 6f medium and lower panels). Expression levels of TMPRSS2 were assessed by RT-PCR in COVID 19 HNSCC patient as well as in five lung cancer patient samples resected from 2011 to 2014 respectively (Fig. 6g). Matched non-tumorous specimens were also analyzed. Interestingly, we found that TMPRSS2 expression was significantly lower in both HNSCC-COVID19 patients and lung cancer tissues compared to matched non-tumorous counterparts. Unlike TMPRSS2 expression, miR-503-5p levels were significantly higher in tumors than in non-tumorous tissues (Fig. 6h).

**Fig. 7:**
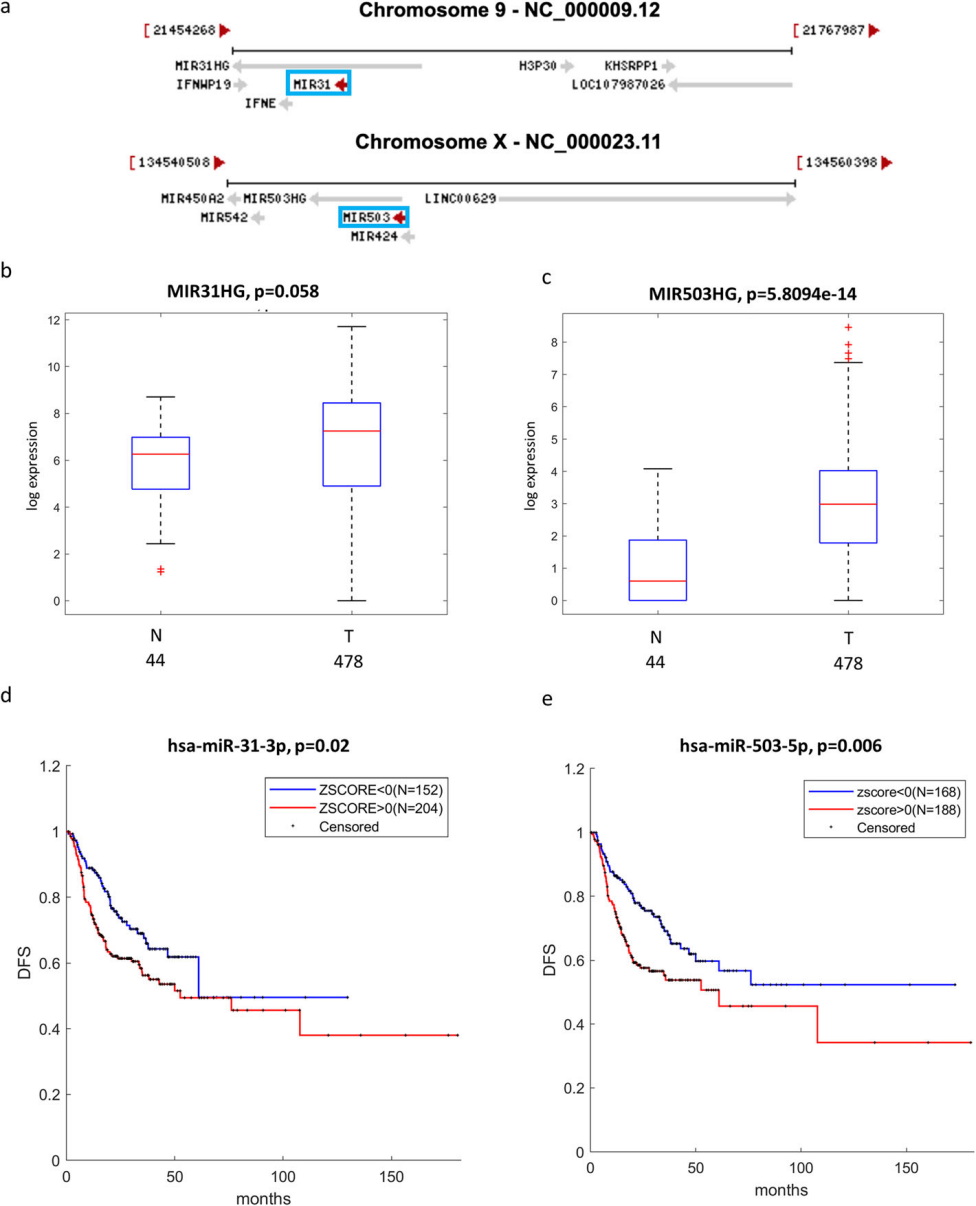
miR-31 and miR-503 locus in HNSCC patients. **a** Schematic representation of miR-31 and miR-503 genes localization. **b**-**c** Box-plot analysis representing expression levels of MIR31HG (**b**) and MIR503HG (**c**) in non-tumorous (N) and tumor (T) tissues from TCGA HNSCC patients. **e**-**f** Kaplan–Meier survival curves for TCGA HNSCC patients showing disease-free survival (DFS) according to miR-31-3p (**e**) or miR-503-5p (**f**) gene expression

Both miR-31-3p (located on Chr 9) and miR-503-5p (located on Chr X) are hosted in long non-coding RNAs, named MIR31HG and MIR503HG, respectively (Fig. 7a). We found that the expression of both MIR31HG and MIR503HG is higher in HNSCC tumors than in non-tumorous tissues (Fig. 7b-c) similarly to miR-31-3p and miR-503-5p. Interestingly, HNSCC patients with high expression of these two microRNAs exhibit shorter disease free survival (Fig. 7d-e).

Collectively these findings suggest that downregulation of TMPRSS2 expression evidenced in HNSCC and LUAD/LUSC is not due to methylation of its regulatory regions. Our study further suggests that aberrant expression of microRNAs targeting TMPRSS2 may assemble a post-transcriptional regulatory network leading to a reduced expression of TMPRSS2 in HNSCC. This regulatory network might also include other non-coding RNA molecules such as LNC-RNAs.

## Discussion

**Fig. 8:**
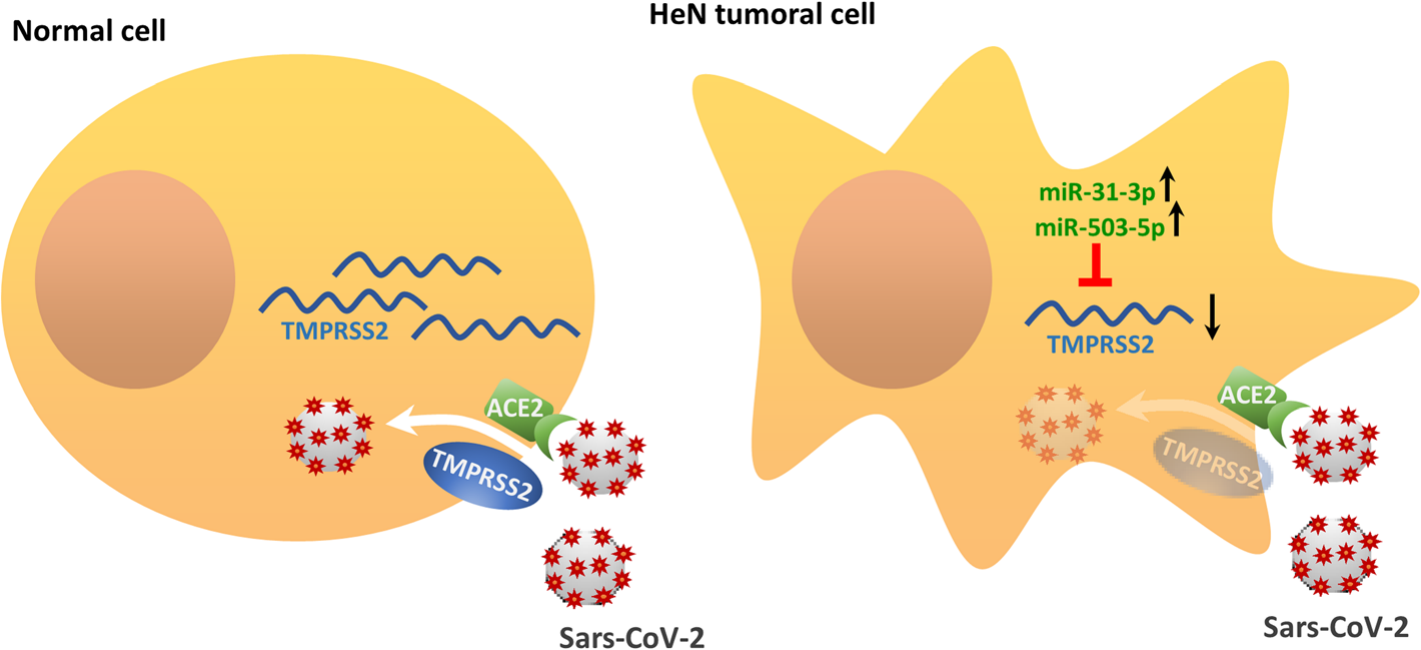
Schematic representation of the proposed molecular mechanism. In normal cell, TMPRSS2 mRNA translation leads to the production of the transmembrane-bound serine protease that allows the internalization of SARS-CoV-2 bound to the ACE2 receptor. On the contrary, in tumoral cell, in particular head and neck tumoral cell, TMPRSS2 mRNA levels are downregulated by the targeting activity of upregulated miRNAs, such as miR-31-3p and miR-503-5p. This might determine a reduction in the expression of the protease and a consequent inhibition of the internalization of SARS-CoV-2-ACE2 comple

The present work indicates that neoplastic tissue from SARS-CoV-2 target organs such as head and neck and lung, might be more resistant to SARS-CoV-2 infection due to reduced expression of TMPRSS2 (Fig. 8). The study was based on the bioinformatics and biostatistics analysis as well as on the subsequent validation of the results in cell models and, as a-proof-of-principle, in neoplastic tissue directly collected from a HNSCC patient affected by Covid19. Notably, we found that reduced expression of TMPRSS2 associates with HPV negative status and TP53 mutations, both of which are important determinants of poor survival in HNSCC patients. We have recently shown that MYC as oncogenic protein di per se and a MYC-dependent gene signature cooperate with gain of function TP53 mutations to foster HNSCC proliferation and to increase resistance to the treatment [24]. Indeed, we found that depletion of mutant p53 proteins in HNSCC cell lines increased TMPRSS2 expression, suggesting that mutant p53 contributes either directly or indirectly to reduced TMPRSS2 expression. Unlike depletion of mutant p53, silencing of YAP and MYC, two oncogenic co-factors of gain of function mutant p53 proteins did not have any effect of TMPRSS2 expression [24, 31, 32]. While these findings may entirely rule out a possibility that gain of function mutant p53-dependent transcriptional networks controls TMPRSS2 expression in HNSCC cells, our observations highlight that tumor cells carrying TP53 mutations may activate post-transcriptional events affecting TMPRSS2 expression. Supporting such possibility, we have detected that the up-regulation of TMPRSS2 targeting microRNAs inversely correlated with expression of TMPRSS2 in HNSCC. We and others have previously reported that gain of function mutant p53 proteins are able to either up-regulate or down-regulate the expression of microRNAs [33, 34]. The lack of methylation in regulatory regions of TMPRSS2 supports further the proposed working model (Fig. 8). Our findings derived from TCGA indicate that similar molecular mechanisms might underlie the reduced expression of TMPRSS2 in both LUAD and LUSC, as no evidence of promoter methylation was also evidenced for lung cancer patients.

These findings further support our hypothesis that TMPRSS2 downregulation in HNSCC patients was associated with selective targeting of microRNAs, in particular those that putatively target TMPRSS2. Expression of six selected microRNAs (miR-193b-3p; miR-503-5p; miR-455-5p; miR-31-3p; miR-193b-5p; miR-2355-5p) significantly anti-correlated with expression of TMPRSS2 (Fig. 5b). Interestingly, the expression of four (miR-193b-3p; miR-193b-5p; miR-2355-5p and miR-455-5p) out of six microRNAs targeting TMPRSS2 is higher in TP53 mutant HNSCC patients than in those with intact TP53 gene (Suppl. Fig. 5). Despite our findings do not prove that the aberrant expression of these microRNAs is directly related to the activity of gain of function mutant p53 proteins, they might suggest that a tumoral context carrying TP53 mutation favors aberrant expression of microRNAs targeting TMPRSS2. It is particularly noteworthy to mention that part of the most significantly predicted pathways targeted by the six microRNAs, were related to respiratory virus infections (e.g., Influenza A) and other viral infections (e.g., Herpes Simplex). The observed microRNA-induced modulation of TMPRSS2 provides new insights for potential assessment of agents capable of regulating the microRNA expression and induces TMPRSS2 downregulation, as SARS-CoV-2 infection prevention strategy [35, 36].

## Conclusions

Clinically, it is increasingly evident that cancer patients represent at least in part, the most vulnerable population target of SARS-CoV-2 infection [5, 37]. This is certainly due to many factors, including the aggressiveness of the type of tumor and the side effects of cancer treatment. Here we provide additional evidence suggesting that tumor tissues are less prone to SARS-CoV-2 infection than non-tumorous tissues, due to reduced expression of TMPRSS2. Furthermore, this reduction is more evident in HNSCC patients with shorter overall survival as well as those with HPV negative status and TP53 mutations.

## Supplementary information

**Additional file 1.**

## Data Availability

The datasets used and analysed during the current study are available from the corresponding author on reasonable request.

## Abbreviations

SARS-CoV-2: Severe acute respiratory syndrome coronavirus 2
ACE2: Angiotensin I Converting Enzyme 2
TPMRSS2: Transmembrane Serine Protease 2
TCGA: The Cancer Genome Atlas
HNSCC: head and neck squamous cell carcinoma
RT: Radiotherapy
HPV: Human Papilloma Virus
3′-UTR: 3′-untranslated region
miRNAs: microRNAs
IRE: Regina Elena Institute
DFS: Disease-free survival
OS: Overall survival
HPRT1: Hypoxanthine phosphoribosyltransferase
CSTF2: Cleavage Stimulation Factor Subunit 2
IL-10: Interleukin 10
IFNγ: Interferon gamma
LUAD: Lung adenocarcinoma
LUAS: Lung squamous cell carcinoma
FPPE: Formalin-Fixed Paraffin-Embedded
MIR31HG: miR-31 host gene
MIR503HG: miR-503 host gene
LNC-RNAs: Long non coding RNAs
