# Supplementary material for: TMPRSS2, a SARS-CoV-2 internalization protease is downregulated in head and neck cancer patients
Source: J Exp Clin Cancer Res. 2020 Sep 23;39:200. doi: 10.1186/s13046-020-01708-6 (PMC7510014; doi:10.1186/s13046-020-01708-6)
Supplement: Supplementary file 1 — Additional file 1. [file 13046_2020_1708_MOESM1_ESM.pdf]

Supplementary figure 1

a

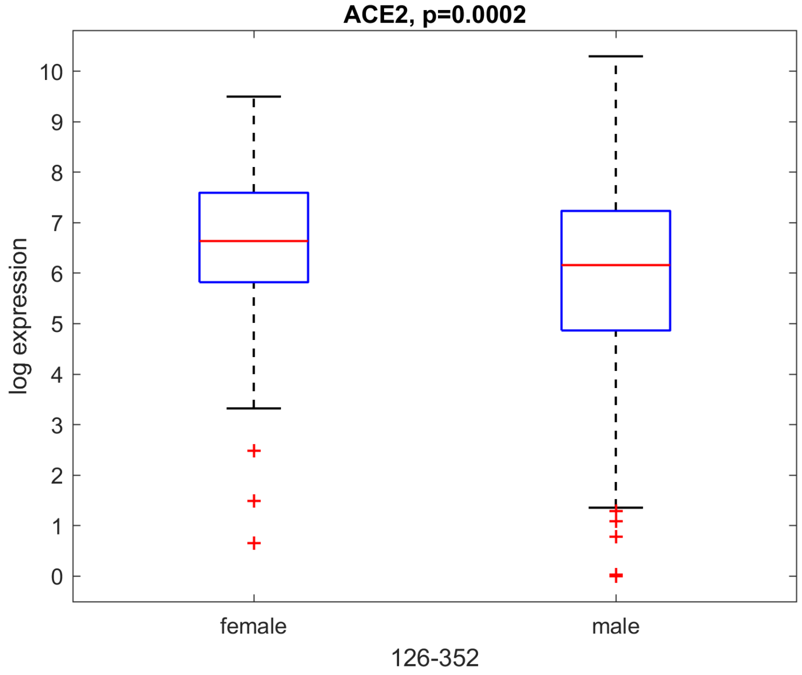

b

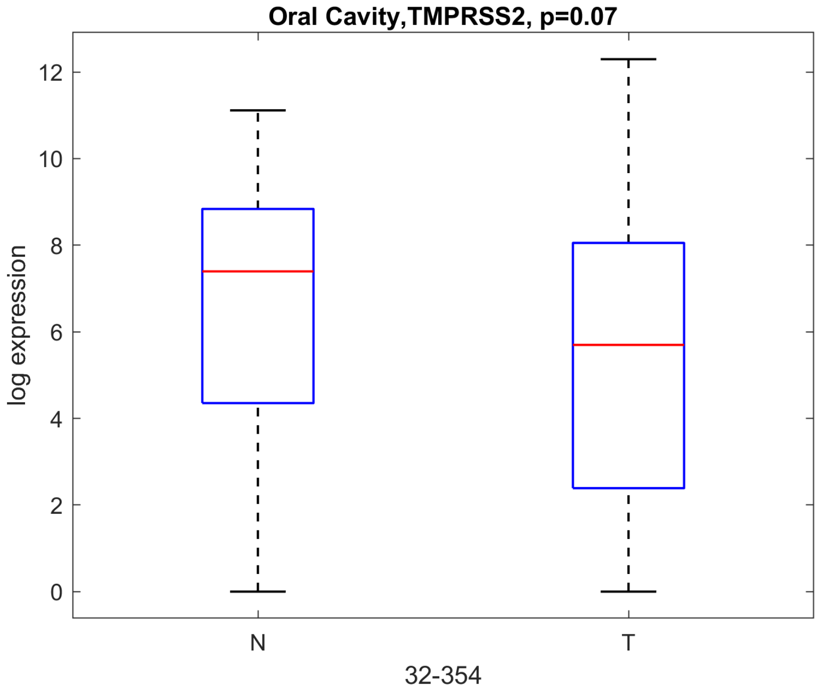

Supplementary figure 2

a

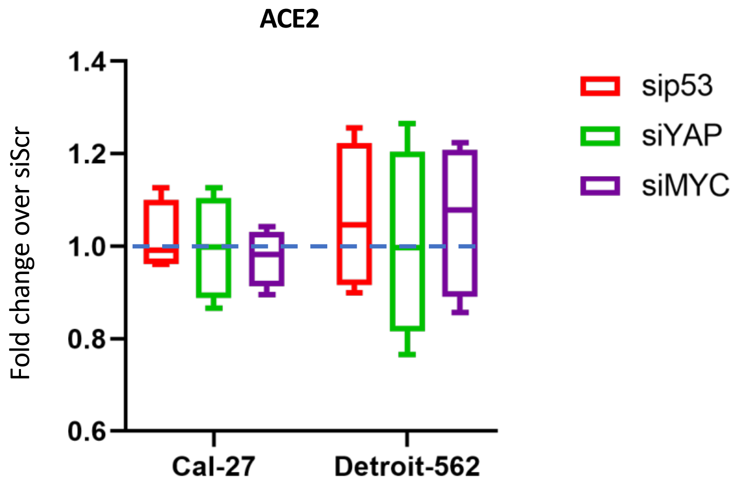

b

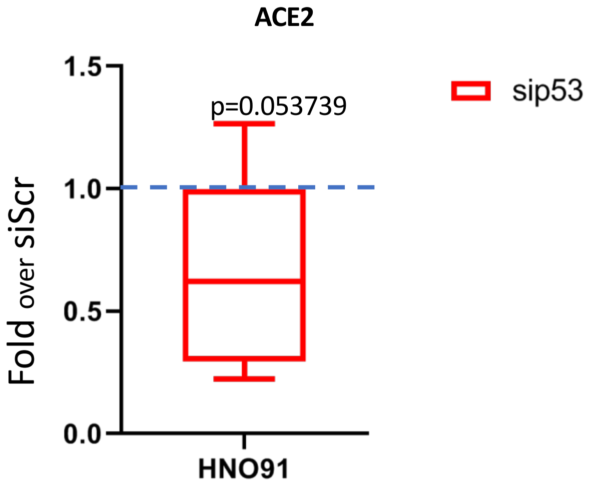

c

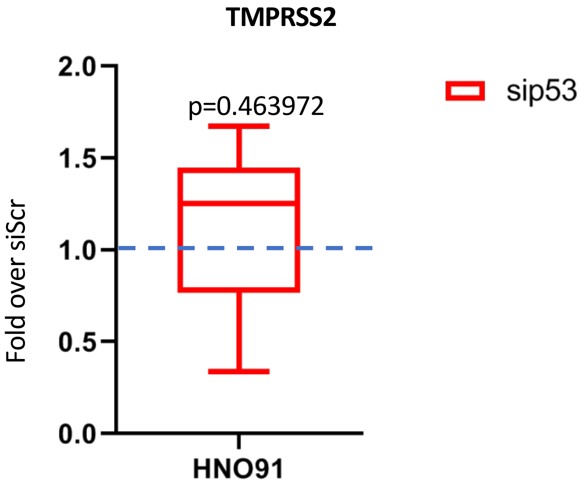

Supplementary figure 3

a

MYC signature

MYC	SF3B3	CCT2	CCT7	EIF3B
PA2G4	PSMD1	CCT3	G3BP1	PWP1
POLE3	PSMA2	NME1	CSTF2	ODC1
NOLC1	RRP9	HPRT1	EIF3D	TCP1
XRCC6	HSP90AB1			

b

immune signature

CTLA4	TNFRSF9	KIR2DL3	IL10	IFNG
PDCD1	TNFRSF4	TNFRSF18	CD40LG	LAG3
CD274	CD40	ICOS	IDO1	CD4
KIR2DL1	HAVCR2			

Supplementary figure 4

a

LUSC

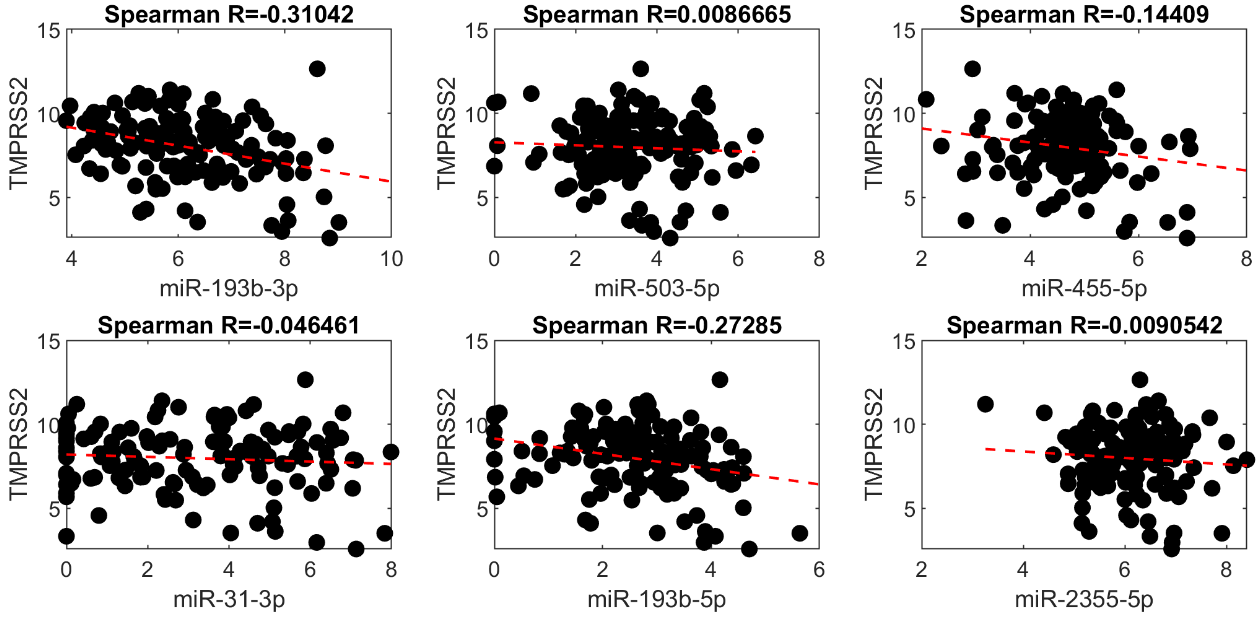

b

LUAD

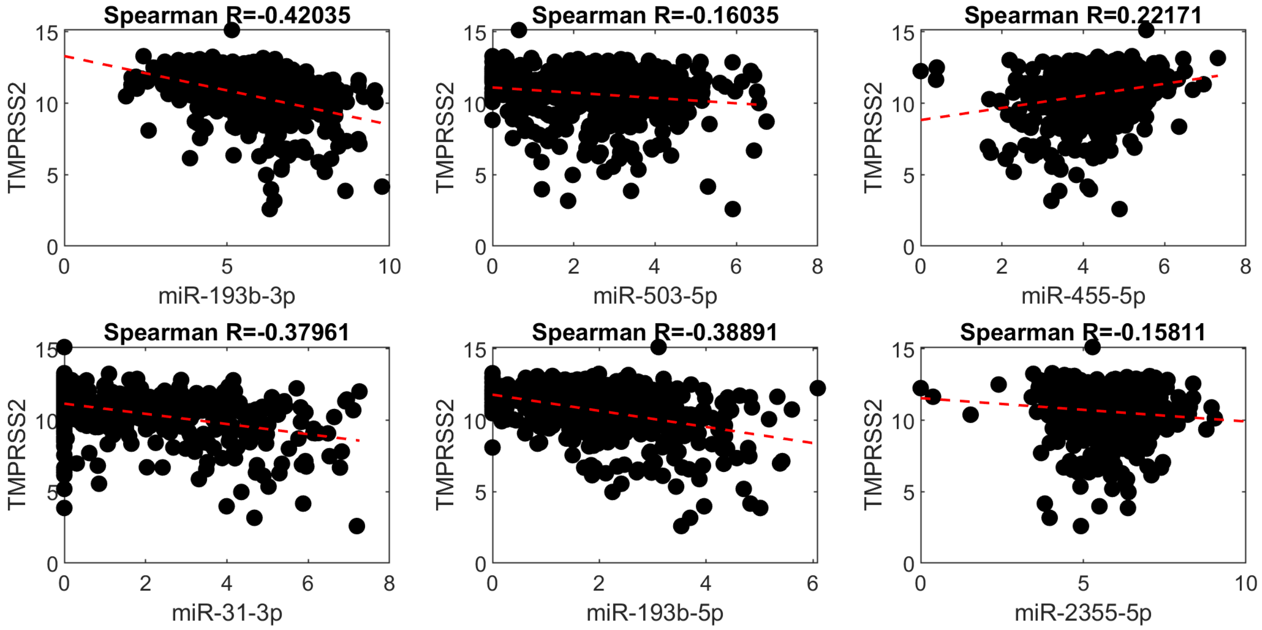

Supplementary figure 5

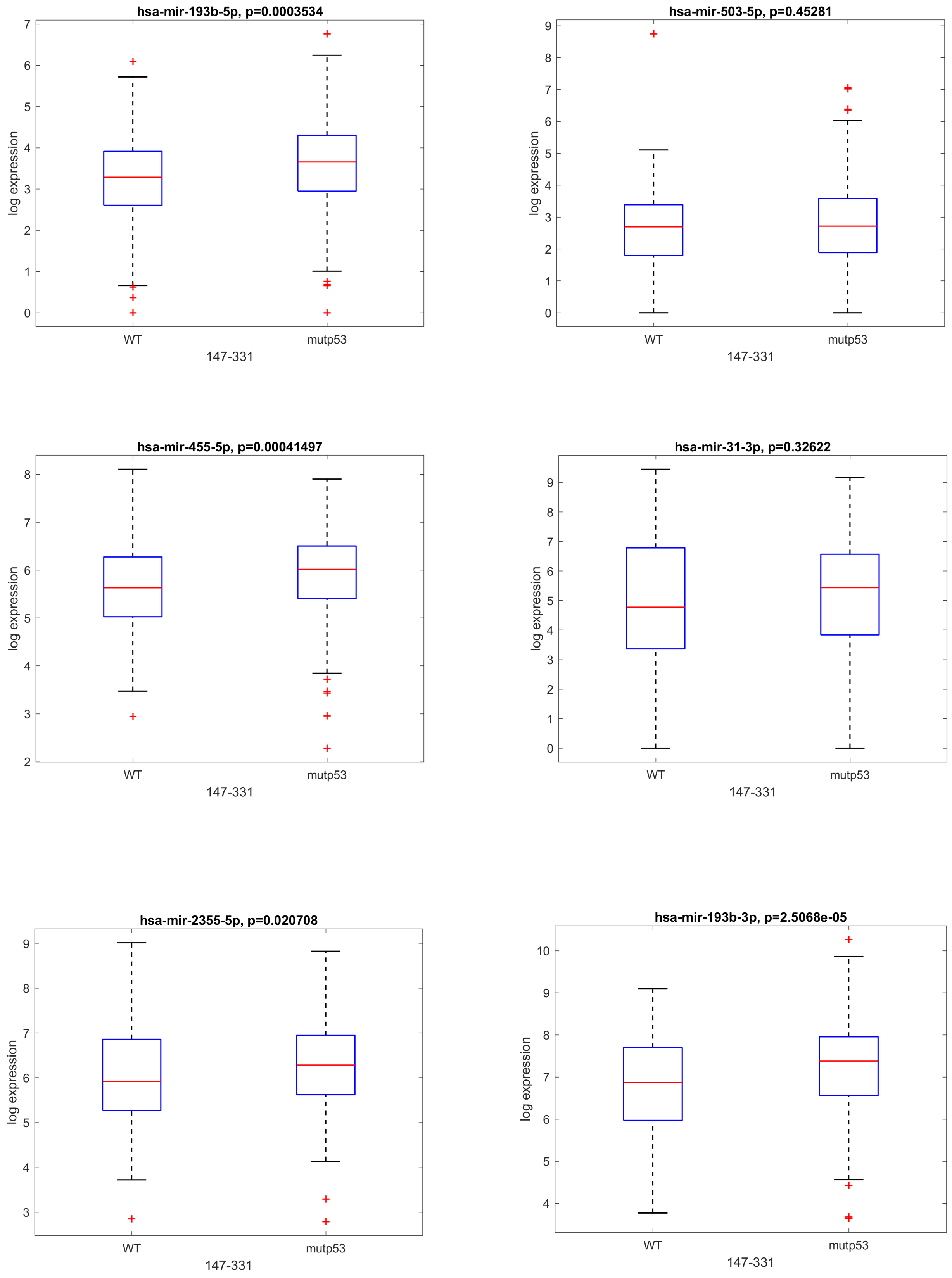

Supplementary figure 6

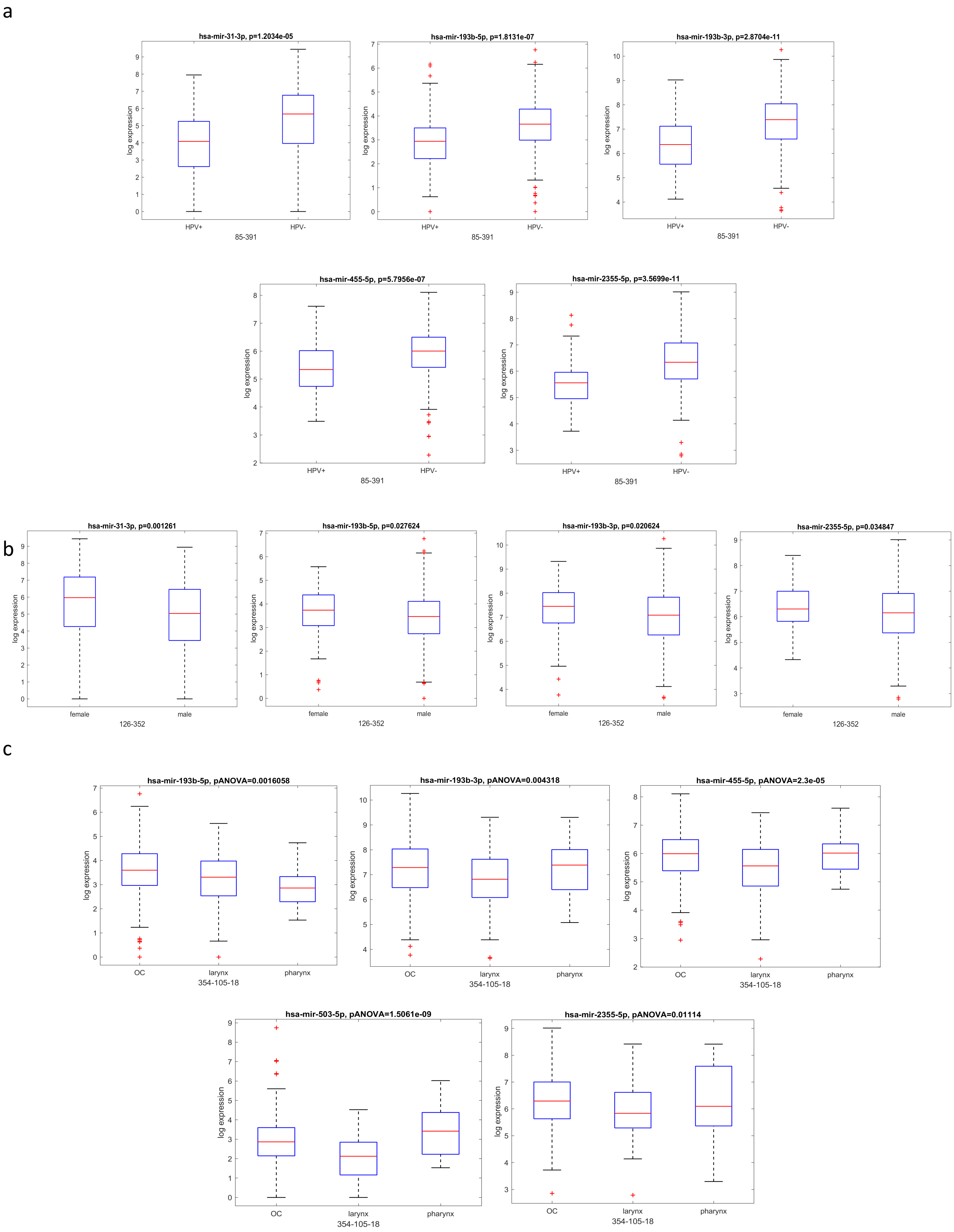

Supplementary Table 1

MiRNA\TMPRSS2 predicted interactions (miRWalk)

mirnaid	refseqid	genesymbol	bindingp	position
hsa-miR-372-3p	NM_005656	TMPRSS2	0.807692307692	3UTR
hsa-miR-520a-3p	NM_005656	TMPRSS2	0.807692307692	3UTR
hsa-miR-587	NM_005656	TMPRSS2	0.807692307692	3UTR
hsa-miR-33b-3p	NM_005656	TMPRSS2	0.807692307692	3UTR
hsa-miR-1224-3p	NM_005656	TMPRSS2	0.807692307692	3UTR
hsa-miR-1227-3p	NM_005656	TMPRSS2	0.807692307692	3UTR
hsa-miR-4284	NM_005656	TMPRSS2	0.807692307692	3UTR
hsa-miR-3616-3p	NM_005656	TMPRSS2	0.807692307692	3UTR
hsa-miR-3620-3p	NM_005656	TMPRSS2	0.807692307692	3UTR
hsa-miR-6762-3p	NM_005656	TMPRSS2	0.807692307692	3UTR
hsa-miR-10527-5p	NM_005656	TMPRSS2	0.807692307692	3UTR
hsa-miR-9851-3p	NM_005656	TMPRSS2	0.807692307692	3UTR
hsa-miR-372-3p	NM_001135099	TMPRSS2	0.807692307692	3UTR
hsa-miR-520a-3p	NM_001135099	TMPRSS2	0.807692307692	3UTR
hsa-miR-513a-3p	NM_001135099	TMPRSS2	0.807692307692	3UTR
hsa-miR-659-3p	NM_001135099	TMPRSS2	0.807692307692	3UTR
hsa-miR-513c-3p	NM_001135099	TMPRSS2	0.807692307692	3UTR
hsa-miR-4270	NM_001135099	TMPRSS2	0.807692307692	3UTR
hsa-miR-3616-3p	NM_001135099	TMPRSS2	0.807692307692	3UTR
hsa-miR-3619-3p	NM_001135099	TMPRSS2	0.807692307692	3UTR
hsa-miR-3620-3p	NM_001135099	TMPRSS2	0.807692307692	3UTR
hsa-miR-4462	NM_001135099	TMPRSS2	0.807692307692	3UTR
hsa-miR-4494	NM_001135099	TMPRSS2	0.807692307692	3UTR
hsa-miR-3976	NM_001135099	TMPRSS2	0.807692307692	3UTR
hsa-miR-6827-5p	NM_001135099	TMPRSS2	0.807692307692	3UTR
hsa-miR-6870-5p	NM_001135099	TMPRSS2	0.807692307692	3UTR
hsa-miR-6881-5p	NM_001135099	TMPRSS2	0.807692307692	3UTR
hsa-miR-7109-5p	NM_001135099	TMPRSS2	0.807692307692	3UTR
hsa-miR-10527-5p	NM_001135099	TMPRSS2	0.807692307692	3UTR
hsa-miR-3652	NM_005656	TMPRSS2	0.815384615385	3UTR
hsa-miR-6870-3p	NM_005656	TMPRSS2	0.815384615385	3UTR
hsa-miR-491-3p	NM_001135099	TMPRSS2	0.815384615385	3UTR
hsa-miR-6870-3p	NM_001135099	TMPRSS2	0.815384615385	3UTR
hsa-miR-628-3p	NM_005656	TMPRSS2	0.820512820513	3UTR
hsa-miR-4308	NM_005656	TMPRSS2	0.820512820513	3UTR
hsa-miR-6754-5p	NM_005656	TMPRSS2	0.820512820513	3UTR
hsa-miR-423-5p	NM_001135099	TMPRSS2	0.820512820513	3UTR
hsa-miR-3175	NM_001135099	TMPRSS2	0.820512820513	3UTR
hsa-miR-6809-5p	NM_001135099	TMPRSS2	0.820512820513	3UTR
hsa-miR-12120	NM_001135099	TMPRSS2	0.826923076923	3UTR
hsa-miR-500b-3p	NM_005656	TMPRSS2	0.835164835165	3UTR

hsa-miR-19b-3p	NM_005656	TMPRSS2	0.846153846154	3UTR
hsa-miR-20a-5p	NM_005656	TMPRSS2	0.846153846154	3UTR
hsa-miR-24-1-5p	NM_005656	TMPRSS2	0.846153846154	3UTR
hsa-miR-26a-5p	NM_005656	TMPRSS2	0.846153846154	3UTR
hsa-miR-28-5p	NM_005656	TMPRSS2	0.846153846154	3UTR
hsa-miR-31-3p	NM_005656	TMPRSS2	0.846153846154	3UTR
hsa-miR-92a-1-5p	NM_005656	TMPRSS2	0.846153846154	3UTR
hsa-miR-103a-3p	NM_005656	TMPRSS2	0.846153846154	3UTR
hsa-miR-105-3p	NM_005656	TMPRSS2	0.846153846154	3UTR
hsa-miR-107	NM_005656	TMPRSS2	0.846153846154	3UTR
hsa-miR-192-3p	NM_005656	TMPRSS2	0.846153846154	3UTR
hsa-miR-197-5p	NM_005656	TMPRSS2	0.846153846154	3UTR
hsa-miR-129-5p	NM_005656	TMPRSS2	0.846153846154	3UTR
hsa-miR-148a-3p	NM_005656	TMPRSS2	0.846153846154	3UTR
hsa-miR-30c-2-3p	NM_005656	TMPRSS2	0.846153846154	3UTR
hsa-miR-30c-2-3p	NM_005656	TMPRSS2	0.846153846154	3UTR
hsa-miR-139-5p	NM_005656	TMPRSS2	0.846153846154	3UTR
hsa-miR-181b-5p	NM_005656	TMPRSS2	0.846153846154	3UTR
hsa-miR-181c-5p	NM_005656	TMPRSS2	0.846153846154	3UTR
hsa-miR-181c-3p	NM_005656	TMPRSS2	0.846153846154	3UTR
hsa-miR-183-3p	NM_005656	TMPRSS2	0.846153846154	3UTR
hsa-miR-204-3p	NM_005656	TMPRSS2	0.846153846154	3UTR
hsa-miR-205-5p	NM_005656	TMPRSS2	0.846153846154	3UTR
hsa-miR-212-3p	NM_005656	TMPRSS2	0.846153846154	3UTR
hsa-miR-214-3p	NM_005656	TMPRSS2	0.846153846154	3UTR
hsa-miR-217-5p	NM_005656	TMPRSS2	0.846153846154	3UTR
hsa-miR-219a-1-3p	NM_005656	TMPRSS2	0.846153846154	3UTR
hsa-miR-222-3p	NM_005656	TMPRSS2	0.846153846154	3UTR
hsa-let-7g-3p	NM_005656	TMPRSS2	0.846153846154	3UTR
hsa-miR-15b-5p	NM_005656	TMPRSS2	0.846153846154	3UTR
hsa-miR-23b-3p	NM_005656	TMPRSS2	0.846153846154	3UTR
hsa-miR-122-3p	NM_005656	TMPRSS2	0.846153846154	3UTR
hsa-miR-138-2-3p	NM_005656	TMPRSS2	0.846153846154	3UTR
hsa-miR-141-5p	NM_005656	TMPRSS2	0.846153846154	3UTR
hsa-miR-143-5p	NM_005656	TMPRSS2	0.846153846154	3UTR
hsa-miR-145-5p	NM_005656	TMPRSS2	0.846153846154	3UTR
hsa-miR-191-5p	NM_005656	TMPRSS2	0.846153846154	3UTR
hsa-miR-9-3p	NM_005656	TMPRSS2	0.846153846154	3UTR
hsa-miR-138-1-3p	NM_005656	TMPRSS2	0.846153846154	3UTR
hsa-miR-150-5p	NM_005656	TMPRSS2	0.846153846154	3UTR
hsa-miR-185-5p	NM_005656	TMPRSS2	0.846153846154	3UTR
hsa-miR-185-3p	NM_005656	TMPRSS2	0.846153846154	3UTR
hsa-miR-188-5p	NM_005656	TMPRSS2	0.846153846154	3UTR
hsa-miR-195-3p	NM_005656	TMPRSS2	0.846153846154	3UTR
hsa-miR-200c-5p	NM_005656	TMPRSS2	0.846153846154	3UTR
hsa-miR-155-5p	NM_005656	TMPRSS2	0.846153846154	3UTR
hsa-miR-301a-3p	NM_005656	TMPRSS2	0.846153846154	3UTR

hsa-miR-130b-5p	NM_005656	TMPRSS2	0.846153846154	3UTR
hsa-miR-130b-5p	NM_005656	TMPRSS2	0.846153846154	3UTR
hsa-miR-365a-5p	NM_005656	TMPRSS2	0.846153846154	3UTR
hsa-miR-365b-5p	NM_005656	TMPRSS2	0.846153846154	3UTR
hsa-miR-302d-5p	NM_005656	TMPRSS2	0.846153846154	3UTR
hsa-miR-370-3p	NM_005656	TMPRSS2	0.846153846154	3UTR
hsa-miR-371a-3p	NM_005656	TMPRSS2	0.846153846154	3UTR
hsa-miR-378a-3p	NM_005656	TMPRSS2	0.846153846154	3UTR
hsa-miR-378a-3p	NM_005656	TMPRSS2	0.846153846154	3UTR
hsa-miR-383-3p	NM_005656	TMPRSS2	0.846153846154	3UTR
hsa-miR-328-5p	NM_005656	TMPRSS2	0.846153846154	3UTR
hsa-miR-342-5p	NM_005656	TMPRSS2	0.846153846154	3UTR
hsa-miR-342-5p	NM_005656	TMPRSS2	0.846153846154	3UTR
hsa-miR-326	NM_005656	TMPRSS2	0.846153846154	3UTR
hsa-miR-135b-5p	NM_005656	TMPRSS2	0.846153846154	3UTR
hsa-miR-331-3p	NM_005656	TMPRSS2	0.846153846154	3UTR
hsa-miR-345-5p	NM_005656	TMPRSS2	0.846153846154	3UTR
hsa-miR-345-3p	NM_005656	TMPRSS2	0.846153846154	3UTR
hsa-miR-425-5p	NM_005656	TMPRSS2	0.846153846154	3UTR
hsa-miR-425-3p	NM_005656	TMPRSS2	0.846153846154	3UTR
hsa-miR-20b-5p	NM_005656	TMPRSS2	0.846153846154	3UTR
hsa-miR-433-5p	NM_005656	TMPRSS2	0.846153846154	3UTR
hsa-miR-329-3p	NM_005656	TMPRSS2	0.846153846154	3UTR
hsa-miR-409-5p	NM_005656	TMPRSS2	0.846153846154	3UTR
hsa-miR-484	NM_005656	TMPRSS2	0.846153846154	3UTR
hsa-miR-490-5p	NM_005656	TMPRSS2	0.846153846154	3UTR
hsa-miR-490-3p	NM_005656	TMPRSS2	0.846153846154	3UTR
hsa-miR-490-3p	NM_005656	TMPRSS2	0.846153846154	3UTR
hsa-miR-491-3p	NM_005656	TMPRSS2	0.846153846154	3UTR
hsa-miR-432-5p	NM_005656	TMPRSS2	0.846153846154	3UTR
hsa-miR-494-3p	NM_005656	TMPRSS2	0.846153846154	3UTR
hsa-miR-193b-3p	NM_005656	TMPRSS2	0.846153846154	3UTR
hsa-miR-498-5p	NM_005656	TMPRSS2	0.846153846154	3UTR
hsa-miR-520e-3p	NM_005656	TMPRSS2	0.846153846154	3UTR
hsa-miR-520f-5p	NM_005656	TMPRSS2	0.846153846154	3UTR
hsa-miR-519c-3p	NM_005656	TMPRSS2	0.846153846154	3UTR
hsa-miR-525-3p	NM_005656	TMPRSS2	0.846153846154	3UTR
hsa-miR-525-3p	NM_005656	TMPRSS2	0.846153846154	3UTR
hsa-miR-518f-3p	NM_005656	TMPRSS2	0.846153846154	3UTR
hsa-miR-520b-5p	NM_005656	TMPRSS2	0.846153846154	3UTR
hsa-miR-518b	NM_005656	TMPRSS2	0.846153846154	3UTR
hsa-miR-526a-5p	NM_005656	TMPRSS2	0.846153846154	3UTR
hsa-miR-520c-5p	NM_005656	TMPRSS2	0.846153846154	3UTR
hsa-miR-518c-3p	NM_005656	TMPRSS2	0.846153846154	3UTR
hsa-miR-518d-5p	NM_005656	TMPRSS2	0.846153846154	3UTR
hsa-miR-522-3p	NM_005656	TMPRSS2	0.846153846154	3UTR
hsa-miR-519a-2-5p	NM_005656	TMPRSS2	0.846153846154	3UTR

hsa-miR-499a-3p	NM_005656	TMPRSS2	0.846153846154	3UTR
hsa-miR-500a-3p	NM_005656	TMPRSS2	0.846153846154	3UTR
hsa-miR-501-3p	NM_005656	TMPRSS2	0.846153846154	3UTR
hsa-miR-502-5p	NM_005656	TMPRSS2	0.846153846154	3UTR
hsa-miR-502-5p	NM_005656	TMPRSS2	0.846153846154	3UTR
hsa-miR-450a-2-3p	NM_005656	TMPRSS2	0.846153846154	3UTR
hsa-miR-503-5p	NM_005656	TMPRSS2	0.846153846154	3UTR
hsa-miR-503-5p	NM_005656	TMPRSS2	0.846153846154	3UTR
hsa-miR-513a-3p	NM_005656	TMPRSS2	0.846153846154	3UTR
hsa-miR-508-5p	NM_005656	TMPRSS2	0.846153846154	3UTR
hsa-miR-510-3p	NM_005656	TMPRSS2	0.846153846154	3UTR
hsa-miR-551a	NM_005656	TMPRSS2	0.846153846154	3UTR
hsa-miR-552-3p	NM_005656	TMPRSS2	0.846153846154	3UTR
hsa-miR-554	NM_005656	TMPRSS2	0.846153846154	3UTR
hsa-miR-571	NM_005656	TMPRSS2	0.846153846154	3UTR
hsa-miR-584-5p	NM_005656	TMPRSS2	0.846153846154	3UTR
hsa-miR-595	NM_005656	TMPRSS2	0.846153846154	3UTR
hsa-miR-602	NM_005656	TMPRSS2	0.846153846154	3UTR
hsa-miR-615-3p	NM_005656	TMPRSS2	0.846153846154	3UTR
hsa-miR-616-3p	NM_005656	TMPRSS2	0.846153846154	3UTR
hsa-miR-622	NM_005656	TMPRSS2	0.846153846154	3UTR
hsa-miR-625-5p	NM_005656	TMPRSS2	0.846153846154	3UTR
hsa-miR-630	NM_005656	TMPRSS2	0.846153846154	3UTR
hsa-miR-630	NM_005656	TMPRSS2	0.846153846154	3UTR
hsa-miR-635	NM_005656	TMPRSS2	0.846153846154	3UTR
hsa-miR-642a-3p	NM_005656	TMPRSS2	0.846153846154	3UTR
hsa-miR-644a	NM_005656	TMPRSS2	0.846153846154	3UTR
hsa-miR-647	NM_005656	TMPRSS2	0.846153846154	3UTR
hsa-miR-650	NM_005656	TMPRSS2	0.846153846154	3UTR
hsa-miR-650	NM_005656	TMPRSS2	0.846153846154	3UTR
hsa-miR-449b-5p	NM_005656	TMPRSS2	0.846153846154	3UTR
hsa-miR-654-5p	NM_005656	TMPRSS2	0.846153846154	3UTR
hsa-miR-542-5p	NM_005656	TMPRSS2	0.846153846154	3UTR
hsa-miR-542-5p	NM_005656	TMPRSS2	0.846153846154	3UTR
hsa-miR-767-5p	NM_005656	TMPRSS2	0.846153846154	3UTR
hsa-miR-767-3p	NM_005656	TMPRSS2	0.846153846154	3UTR
hsa-miR-320c	NM_005656	TMPRSS2	0.846153846154	3UTR
hsa-miR-1323	NM_005656	TMPRSS2	0.846153846154	3UTR
hsa-miR-1271-5p	NM_005656	TMPRSS2	0.846153846154	3UTR
hsa-miR-764	NM_005656	TMPRSS2	0.846153846154	3UTR
hsa-miR-765	NM_005656	TMPRSS2	0.846153846154	3UTR
hsa-miR-770-5p	NM_005656	TMPRSS2	0.846153846154	3UTR
hsa-miR-874-5p	NM_005656	TMPRSS2	0.846153846154	3UTR
hsa-miR-541-5p	NM_005656	TMPRSS2	0.846153846154	3UTR
hsa-miR-875-3p	NM_005656	TMPRSS2	0.846153846154	3UTR
hsa-miR-877-5p	NM_005656	TMPRSS2	0.846153846154	3UTR
hsa-miR-877-3p	NM_005656	TMPRSS2	0.846153846154	3UTR

hsa-miR-665	NM_005656	TMPRSS2	0.846153846154	3UTR
hsa-miR-301b-5p	NM_005656	TMPRSS2	0.846153846154	3UTR
hsa-miR-920	NM_005656	TMPRSS2	0.846153846154	3UTR
hsa-miR-922	NM_005656	TMPRSS2	0.846153846154	3UTR
hsa-miR-934	NM_005656	TMPRSS2	0.846153846154	3UTR
hsa-miR-1229-3p	NM_005656	TMPRSS2	0.846153846154	3UTR
hsa-miR-1229-3p	NM_005656	TMPRSS2	0.846153846154	3UTR
hsa-miR-663b	NM_005656	TMPRSS2	0.846153846154	3UTR
hsa-miR-1207-5p	NM_005656	TMPRSS2	0.846153846154	3UTR
hsa-miR-1286	NM_005656	TMPRSS2	0.846153846154	3UTR
hsa-miR-1248	NM_005656	TMPRSS2	0.846153846154	3UTR
hsa-miR-1249-5p	NM_005656	TMPRSS2	0.846153846154	3UTR
hsa-miR-1307-5p	NM_005656	TMPRSS2	0.846153846154	3UTR
hsa-miR-1307-5p	NM_005656	TMPRSS2	0.846153846154	3UTR
hsa-miR-513c-3p	NM_005656	TMPRSS2	0.846153846154	3UTR
hsa-miR-320d	NM_005656	TMPRSS2	0.846153846154	3UTR
hsa-miR-1912-3p	NM_005656	TMPRSS2	0.846153846154	3UTR
hsa-miR-1913	NM_005656	TMPRSS2	0.846153846154	3UTR
hsa-miR-1914-3p	NM_005656	TMPRSS2	0.846153846154	3UTR
hsa-miR-1976	NM_005656	TMPRSS2	0.846153846154	3UTR
hsa-miR-2110	NM_005656	TMPRSS2	0.846153846154	3UTR
hsa-miR-2115-5p	NM_005656	TMPRSS2	0.846153846154	3UTR
hsa-miR-2116-5p	NM_005656	TMPRSS2	0.846153846154	3UTR
hsa-miR-2116-3p	NM_005656	TMPRSS2	0.846153846154	3UTR
hsa-miR-2278	NM_005656	TMPRSS2	0.846153846154	3UTR
hsa-miR-2278	NM_005656	TMPRSS2	0.846153846154	3UTR
hsa-miR-2681-5p	NM_005656	TMPRSS2	0.846153846154	3UTR
hsa-miR-3117-3p	NM_005656	TMPRSS2	0.846153846154	3UTR
hsa-miR-3125	NM_005656	TMPRSS2	0.846153846154	3UTR
hsa-miR-3126-5p	NM_005656	TMPRSS2	0.846153846154	3UTR
hsa-miR-3127-5p	NM_005656	TMPRSS2	0.846153846154	3UTR
hsa-miR-3129-3p	NM_005656	TMPRSS2	0.846153846154	3UTR
hsa-miR-3130-3p	NM_005656	TMPRSS2	0.846153846154	3UTR
hsa-miR-3131	NM_005656	TMPRSS2	0.846153846154	3UTR
hsa-miR-378b	NM_005656	TMPRSS2	0.846153846154	3UTR
hsa-miR-3141	NM_005656	TMPRSS2	0.846153846154	3UTR
hsa-miR-3142	NM_005656	TMPRSS2	0.846153846154	3UTR
hsa-miR-3147	NM_005656	TMPRSS2	0.846153846154	3UTR
hsa-miR-3074-3p	NM_005656	TMPRSS2	0.846153846154	3UTR
hsa-miR-3155a	NM_005656	TMPRSS2	0.846153846154	3UTR
hsa-miR-3158-3p	NM_005656	TMPRSS2	0.846153846154	3UTR
hsa-miR-3160-3p	NM_005656	TMPRSS2	0.846153846154	3UTR
hsa-miR-3162-5p	NM_005656	TMPRSS2	0.846153846154	3UTR
hsa-miR-3163	NM_005656	TMPRSS2	0.846153846154	3UTR
hsa-miR-3163	NM_005656	TMPRSS2	0.846153846154	3UTR
hsa-miR-3169	NM_005656	TMPRSS2	0.846153846154	3UTR
hsa-miR-3183	NM_005656	TMPRSS2	0.846153846154	3UTR

hsa-miR-3065-3p	NM_005656	TMPRSS2	0.846153846154	3UTR
hsa-miR-3189-3p	NM_005656	TMPRSS2	0.846153846154	3UTR
hsa-miR-3192-5p	NM_005656	TMPRSS2	0.846153846154	3UTR
hsa-miR-514b-3p	NM_005656	TMPRSS2	0.846153846154	3UTR
hsa-miR-4296	NM_005656	TMPRSS2	0.846153846154	3UTR
hsa-miR-378c	NM_005656	TMPRSS2	0.846153846154	3UTR
hsa-miR-4293	NM_005656	TMPRSS2	0.846153846154	3UTR
hsa-miR-4298	NM_005656	TMPRSS2	0.846153846154	3UTR
hsa-miR-4300	NM_005656	TMPRSS2	0.846153846154	3UTR
hsa-miR-4320	NM_005656	TMPRSS2	0.846153846154	3UTR
hsa-miR-4317	NM_005656	TMPRSS2	0.846153846154	3UTR
hsa-miR-4323	NM_005656	TMPRSS2	0.846153846154	3UTR
hsa-miR-4257	NM_005656	TMPRSS2	0.846153846154	3UTR
hsa-miR-4254	NM_005656	TMPRSS2	0.846153846154	3UTR
hsa-miR-4252	NM_005656	TMPRSS2	0.846153846154	3UTR
hsa-miR-4266	NM_005656	TMPRSS2	0.846153846154	3UTR
hsa-miR-2355-5p	NM_005656	TMPRSS2	0.846153846154	3UTR
hsa-miR-4269	NM_005656	TMPRSS2	0.846153846154	3UTR
hsa-miR-4271	NM_005656	TMPRSS2	0.846153846154	3UTR
hsa-miR-4273	NM_005656	TMPRSS2	0.846153846154	3UTR
hsa-miR-4279	NM_005656	TMPRSS2	0.846153846154	3UTR
hsa-miR-4278	NM_005656	TMPRSS2	0.846153846154	3UTR
hsa-miR-4282	NM_005656	TMPRSS2	0.846153846154	3UTR
hsa-miR-4285	NM_005656	TMPRSS2	0.846153846154	3UTR
hsa-miR-4291	NM_005656	TMPRSS2	0.846153846154	3UTR
hsa-miR-3610	NM_005656	TMPRSS2	0.846153846154	3UTR
hsa-miR-3610	NM_005656	TMPRSS2	0.846153846154	3UTR
hsa-miR-3619-3p	NM_005656	TMPRSS2	0.846153846154	3UTR
hsa-miR-23c	NM_005656	TMPRSS2	0.846153846154	3UTR
hsa-miR-3621	NM_005656	TMPRSS2	0.846153846154	3UTR
hsa-miR-3622b-5p	NM_005656	TMPRSS2	0.846153846154	3UTR
hsa-miR-3650	NM_005656	TMPRSS2	0.846153846154	3UTR
hsa-miR-3654	NM_005656	TMPRSS2	0.846153846154	3UTR
hsa-miR-3654	NM_005656	TMPRSS2	0.846153846154	3UTR
hsa-miR-3659	NM_005656	TMPRSS2	0.846153846154	3UTR
hsa-miR-3664-5p	NM_005656	TMPRSS2	0.846153846154	3UTR
hsa-miR-3665	NM_005656	TMPRSS2	0.846153846154	3UTR
hsa-miR-3665	NM_005656	TMPRSS2	0.846153846154	3UTR
hsa-miR-3675-3p	NM_005656	TMPRSS2	0.846153846154	3UTR
hsa-miR-3679-3p	NM_005656	TMPRSS2	0.846153846154	3UTR
hsa-miR-3680-5p	NM_005656	TMPRSS2	0.846153846154	3UTR
hsa-miR-3685	NM_005656	TMPRSS2	0.846153846154	3UTR
hsa-miR-3689a-5p	NM_005656	TMPRSS2	0.846153846154	3UTR
hsa-miR-3692-3p	NM_005656	TMPRSS2	0.846153846154	3UTR
hsa-miR-3714	NM_005656	TMPRSS2	0.846153846154	3UTR
hsa-miR-3689b-5p	NM_005656	TMPRSS2	0.846153846154	3UTR
hsa-miR-3908	NM_005656	TMPRSS2	0.846153846154	3UTR

hsa-miR-3909	NM_005656	TMPRSS2	0.846153846154	3UTR
hsa-miR-3911	NM_005656	TMPRSS2	0.846153846154	3UTR
hsa-miR-3916	NM_005656	TMPRSS2	0.846153846154	3UTR
hsa-miR-3917	NM_005656	TMPRSS2	0.846153846154	3UTR
hsa-miR-3924	NM_005656	TMPRSS2	0.846153846154	3UTR
hsa-miR-3934-5p	NM_005656	TMPRSS2	0.846153846154	3UTR
hsa-miR-3934-5p	NM_005656	TMPRSS2	0.846153846154	3UTR
hsa-miR-3934-5p	NM_005656	TMPRSS2	0.846153846154	3UTR
hsa-miR-3934-3p	NM_005656	TMPRSS2	0.846153846154	3UTR
hsa-miR-3936	NM_005656	TMPRSS2	0.846153846154	3UTR
hsa-miR-3943	NM_005656	TMPRSS2	0.846153846154	3UTR
hsa-miR-374c-3p	NM_005656	TMPRSS2	0.846153846154	3UTR
hsa-miR-4420	NM_005656	TMPRSS2	0.846153846154	3UTR
hsa-miR-4420	NM_005656	TMPRSS2	0.846153846154	3UTR
hsa-miR-4421	NM_005656	TMPRSS2	0.846153846154	3UTR
hsa-miR-4421	NM_005656	TMPRSS2	0.846153846154	3UTR
hsa-miR-4428	NM_005656	TMPRSS2	0.846153846154	3UTR
hsa-miR-4433a-5p	NM_005656	TMPRSS2	0.846153846154	3UTR
hsa-miR-4433a-3p	NM_005656	TMPRSS2	0.846153846154	3UTR
hsa-miR-4433a-3p	NM_005656	TMPRSS2	0.846153846154	3UTR
hsa-miR-4433a-3p	NM_005656	TMPRSS2	0.846153846154	3UTR
hsa-miR-4437	NM_005656	TMPRSS2	0.846153846154	3UTR
hsa-miR-4448	NM_005656	TMPRSS2	0.846153846154	3UTR
hsa-miR-4458	NM_005656	TMPRSS2	0.846153846154	3UTR
hsa-miR-4472	NM_005656	TMPRSS2	0.846153846154	3UTR
hsa-miR-4473	NM_005656	TMPRSS2	0.846153846154	3UTR
hsa-miR-4476	NM_005656	TMPRSS2	0.846153846154	3UTR
hsa-miR-4478	NM_005656	TMPRSS2	0.846153846154	3UTR
hsa-miR-3689e	NM_005656	TMPRSS2	0.846153846154	3UTR
hsa-miR-4479	NM_005656	TMPRSS2	0.846153846154	3UTR
hsa-miR-3155b	NM_005656	TMPRSS2	0.846153846154	3UTR
hsa-miR-4485-5p	NM_005656	TMPRSS2	0.846153846154	3UTR
hsa-miR-4487	NM_005656	TMPRSS2	0.846153846154	3UTR
hsa-miR-4496	NM_005656	TMPRSS2	0.846153846154	3UTR
hsa-miR-4498	NM_005656	TMPRSS2	0.846153846154	3UTR
hsa-miR-4500	NM_005656	TMPRSS2	0.846153846154	3UTR
hsa-miR-4502	NM_005656	TMPRSS2	0.846153846154	3UTR
hsa-miR-2392	NM_005656	TMPRSS2	0.846153846154	3UTR
hsa-miR-4514	NM_005656	TMPRSS2	0.846153846154	3UTR
hsa-miR-4518	NM_005656	TMPRSS2	0.846153846154	3UTR
hsa-miR-4524a-5p	NM_005656	TMPRSS2	0.846153846154	3UTR
hsa-miR-4535	NM_005656	TMPRSS2	0.846153846154	3UTR
hsa-miR-1587	NM_005656	TMPRSS2	0.846153846154	3UTR
hsa-miR-4536-5p	NM_005656	TMPRSS2	0.846153846154	3UTR
hsa-miR-3960	NM_005656	TMPRSS2	0.846153846154	3UTR
hsa-miR-3975	NM_005656	TMPRSS2	0.846153846154	3UTR
hsa-miR-4632-5p	NM_005656	TMPRSS2	0.846153846154	3UTR

hsa-miR-4638-3p	NM_005656	TMPRSS2	0.846153846154	3UTR
hsa-miR-4640-3p	NM_005656	TMPRSS2	0.846153846154	3UTR
hsa-miR-4642	NM_005656	TMPRSS2	0.846153846154	3UTR
hsa-miR-4645-5p	NM_005656	TMPRSS2	0.846153846154	3UTR
hsa-miR-4648	NM_005656	TMPRSS2	0.846153846154	3UTR
hsa-miR-4655-5p	NM_005656	TMPRSS2	0.846153846154	3UTR
hsa-miR-4661-3p	NM_005656	TMPRSS2	0.846153846154	3UTR
hsa-miR-4664-5p	NM_005656	TMPRSS2	0.846153846154	3UTR
hsa-miR-4669	NM_005656	TMPRSS2	0.846153846154	3UTR
hsa-miR-4671-3p	NM_005656	TMPRSS2	0.846153846154	3UTR
hsa-miR-4681	NM_005656	TMPRSS2	0.846153846154	3UTR
hsa-miR-4685-5p	NM_005656	TMPRSS2	0.846153846154	3UTR
hsa-miR-4685-5p	NM_005656	TMPRSS2	0.846153846154	3UTR
hsa-miR-1343-3p	NM_005656	TMPRSS2	0.846153846154	3UTR
hsa-miR-4690-5p	NM_005656	TMPRSS2	0.846153846154	3UTR
hsa-miR-4695-5p	NM_005656	TMPRSS2	0.846153846154	3UTR
hsa-miR-4696	NM_005656	TMPRSS2	0.846153846154	3UTR
hsa-miR-4700-5p	NM_005656	TMPRSS2	0.846153846154	3UTR
hsa-miR-4701-5p	NM_005656	TMPRSS2	0.846153846154	3UTR
hsa-miR-203b-5p	NM_005656	TMPRSS2	0.846153846154	3UTR
hsa-miR-4710	NM_005656	TMPRSS2	0.846153846154	3UTR
hsa-miR-4712-3p	NM_005656	TMPRSS2	0.846153846154	3UTR
hsa-miR-4716-3p	NM_005656	TMPRSS2	0.846153846154	3UTR
hsa-miR-4723-3p	NM_005656	TMPRSS2	0.846153846154	3UTR
hsa-miR-451b	NM_005656	TMPRSS2	0.846153846154	3UTR
hsa-miR-4724-5p	NM_005656	TMPRSS2	0.846153846154	3UTR
hsa-miR-4731-5p	NM_005656	TMPRSS2	0.846153846154	3UTR
hsa-miR-4736	NM_005656	TMPRSS2	0.846153846154	3UTR
hsa-miR-3064-3p	NM_005656	TMPRSS2	0.846153846154	3UTR
hsa-miR-4739	NM_005656	TMPRSS2	0.846153846154	3UTR
hsa-miR-4742-5p	NM_005656	TMPRSS2	0.846153846154	3UTR
hsa-miR-4747-5p	NM_005656	TMPRSS2	0.846153846154	3UTR
hsa-miR-4747-3p	NM_005656	TMPRSS2	0.846153846154	3UTR
hsa-miR-4748	NM_005656	TMPRSS2	0.846153846154	3UTR
hsa-miR-4753-3p	NM_005656	TMPRSS2	0.846153846154	3UTR
hsa-miR-4764-3p	NM_005656	TMPRSS2	0.846153846154	3UTR
hsa-miR-4768-5p	NM_005656	TMPRSS2	0.846153846154	3UTR
hsa-miR-4769-5p	NM_005656	TMPRSS2	0.846153846154	3UTR
hsa-miR-4774-5p	NM_005656	TMPRSS2	0.846153846154	3UTR
hsa-miR-4774-3p	NM_005656	TMPRSS2	0.846153846154	3UTR
hsa-miR-4777-3p	NM_005656	TMPRSS2	0.846153846154	3UTR
hsa-miR-4777-3p	NM_005656	TMPRSS2	0.846153846154	3UTR
hsa-miR-4780	NM_005656	TMPRSS2	0.846153846154	3UTR
hsa-miR-4436b-3p	NM_005656	TMPRSS2	0.846153846154	3UTR
hsa-miR-4781-5p	NM_005656	TMPRSS2	0.846153846154	3UTR
hsa-miR-4786-5p	NM_005656	TMPRSS2	0.846153846154	3UTR
hsa-miR-4793-5p	NM_005656	TMPRSS2	0.846153846154	3UTR

hsa-miR-4794	NM_005656	TMPRSS2	0.846153846154	3UTR
hsa-miR-4796-5p	NM_005656	TMPRSS2	0.846153846154	3UTR
hsa-miR-4796-3p	NM_005656	TMPRSS2	0.846153846154	3UTR
hsa-miR-4797-5p	NM_005656	TMPRSS2	0.846153846154	3UTR
hsa-miR-4802-5p	NM_005656	TMPRSS2	0.846153846154	3UTR
hsa-miR-4802-3p	NM_005656	TMPRSS2	0.846153846154	3UTR
hsa-miR-5006-3p	NM_005656	TMPRSS2	0.846153846154	3UTR
hsa-miR-5010-3p	NM_005656	TMPRSS2	0.846153846154	3UTR
hsa-miR-5087	NM_005656	TMPRSS2	0.846153846154	3UTR
hsa-miR-5093	NM_005656	TMPRSS2	0.846153846154	3UTR
hsa-miR-5194	NM_005656	TMPRSS2	0.846153846154	3UTR
hsa-miR-5196-5p	NM_005656	TMPRSS2	0.846153846154	3UTR
hsa-miR-5196-3p	NM_005656	TMPRSS2	0.846153846154	3UTR
hsa-miR-5571-5p	NM_005656	TMPRSS2	0.846153846154	3UTR
hsa-miR-5571-5p	NM_005656	TMPRSS2	0.846153846154	3UTR
hsa-miR-5580-3p	NM_005656	TMPRSS2	0.846153846154	3UTR
hsa-miR-5581-3p	NM_005656	TMPRSS2	0.846153846154	3UTR
hsa-miR-548au-3p	NM_005656	TMPRSS2	0.846153846154	3UTR
hsa-miR-1295b-5p	NM_005656	TMPRSS2	0.846153846154	3UTR
hsa-miR-5591-5p	NM_005656	TMPRSS2	0.846153846154	3UTR
hsa-miR-5682	NM_005656	TMPRSS2	0.846153846154	3UTR
hsa-miR-5685	NM_005656	TMPRSS2	0.846153846154	3UTR
hsa-miR-5691	NM_005656	TMPRSS2	0.846153846154	3UTR
hsa-miR-5739	NM_005656	TMPRSS2	0.846153846154	3UTR
hsa-miR-6070	NM_005656	TMPRSS2	0.846153846154	3UTR
hsa-miR-6072	NM_005656	TMPRSS2	0.846153846154	3UTR
hsa-miR-6076	NM_005656	TMPRSS2	0.846153846154	3UTR
hsa-miR-6085	NM_005656	TMPRSS2	0.846153846154	3UTR
hsa-miR-6086	NM_005656	TMPRSS2	0.846153846154	3UTR
hsa-miR-6124	NM_005656	TMPRSS2	0.846153846154	3UTR
hsa-miR-6125	NM_005656	TMPRSS2	0.846153846154	3UTR
hsa-miR-6131	NM_005656	TMPRSS2	0.846153846154	3UTR
hsa-miR-6131	NM_005656	TMPRSS2	0.846153846154	3UTR
hsa-miR-6132	NM_005656	TMPRSS2	0.846153846154	3UTR
hsa-miR-6133	NM_005656	TMPRSS2	0.846153846154	3UTR
hsa-miR-6504-5p	NM_005656	TMPRSS2	0.846153846154	3UTR
hsa-miR-6508-3p	NM_005656	TMPRSS2	0.846153846154	3UTR
hsa-miR-6509-3p	NM_005656	TMPRSS2	0.846153846154	3UTR
hsa-miR-6511a-5p	NM_005656	TMPRSS2	0.846153846154	3UTR
hsa-miR-6515-5p	NM_005656	TMPRSS2	0.846153846154	3UTR
hsa-miR-6515-3p	NM_005656	TMPRSS2	0.846153846154	3UTR
hsa-miR-6715a-3p	NM_005656	TMPRSS2	0.846153846154	3UTR
hsa-miR-6717-5p	NM_005656	TMPRSS2	0.846153846154	3UTR
hsa-miR-6719-3p	NM_005656	TMPRSS2	0.846153846154	3UTR
hsa-miR-6720-5p	NM_005656	TMPRSS2	0.846153846154	3UTR
hsa-miR-892c-5p	NM_005656	TMPRSS2	0.846153846154	3UTR
hsa-miR-6727-3p	NM_005656	TMPRSS2	0.846153846154	3UTR

hsa-miR-6728-5p	NM_005656	TMPRSS2	0.846153846154	3UTR
hsa-miR-6729-5p	NM_005656	TMPRSS2	0.846153846154	3UTR
hsa-miR-6730-5p	NM_005656	TMPRSS2	0.846153846154	3UTR
hsa-miR-6732-5p	NM_005656	TMPRSS2	0.846153846154	3UTR
hsa-miR-6734-5p	NM_005656	TMPRSS2	0.846153846154	3UTR
hsa-miR-6735-5p	NM_005656	TMPRSS2	0.846153846154	3UTR
hsa-miR-6737-5p	NM_005656	TMPRSS2	0.846153846154	3UTR
hsa-miR-6739-3p	NM_005656	TMPRSS2	0.846153846154	3UTR
hsa-miR-6742-3p	NM_005656	TMPRSS2	0.846153846154	3UTR
hsa-miR-6743-3p	NM_005656	TMPRSS2	0.846153846154	3UTR
hsa-miR-6746-5p	NM_005656	TMPRSS2	0.846153846154	3UTR
hsa-miR-6746-3p	NM_005656	TMPRSS2	0.846153846154	3UTR
hsa-miR-6747-5p	NM_005656	TMPRSS2	0.846153846154	3UTR
hsa-miR-6747-5p	NM_005656	TMPRSS2	0.846153846154	3UTR
hsa-miR-6756-3p	NM_005656	TMPRSS2	0.846153846154	3UTR
hsa-miR-6757-5p	NM_005656	TMPRSS2	0.846153846154	3UTR
hsa-miR-6758-3p	NM_005656	TMPRSS2	0.846153846154	3UTR
hsa-miR-6759-5p	NM_005656	TMPRSS2	0.846153846154	3UTR
hsa-miR-6760-3p	NM_005656	TMPRSS2	0.846153846154	3UTR
hsa-miR-6765-3p	NM_005656	TMPRSS2	0.846153846154	3UTR
hsa-miR-6771-3p	NM_005656	TMPRSS2	0.846153846154	3UTR
hsa-miR-6772-5p	NM_005656	TMPRSS2	0.846153846154	3UTR
hsa-miR-6772-3p	NM_005656	TMPRSS2	0.846153846154	3UTR
hsa-miR-6773-5p	NM_005656	TMPRSS2	0.846153846154	3UTR
hsa-miR-6773-5p	NM_005656	TMPRSS2	0.846153846154	3UTR
hsa-miR-6777-5p	NM_005656	TMPRSS2	0.846153846154	3UTR
hsa-miR-6779-5p	NM_005656	TMPRSS2	0.846153846154	3UTR
hsa-miR-6779-3p	NM_005656	TMPRSS2	0.846153846154	3UTR
hsa-miR-6781-3p	NM_005656	TMPRSS2	0.846153846154	3UTR
hsa-miR-6787-5p	NM_005656	TMPRSS2	0.846153846154	3UTR
hsa-miR-6792-5p	NM_005656	TMPRSS2	0.846153846154	3UTR
hsa-miR-6792-3p	NM_005656	TMPRSS2	0.846153846154	3UTR
hsa-miR-6793-5p	NM_005656	TMPRSS2	0.846153846154	3UTR
hsa-miR-6797-5p	NM_005656	TMPRSS2	0.846153846154	3UTR
hsa-miR-6797-3p	NM_005656	TMPRSS2	0.846153846154	3UTR
hsa-miR-6801-5p	NM_005656	TMPRSS2	0.846153846154	3UTR
hsa-miR-6802-5p	NM_005656	TMPRSS2	0.846153846154	3UTR
hsa-miR-6803-3p	NM_005656	TMPRSS2	0.846153846154	3UTR
hsa-miR-6809-5p	NM_005656	TMPRSS2	0.846153846154	3UTR
hsa-miR-6810-5p	NM_005656	TMPRSS2	0.846153846154	3UTR
hsa-miR-6812-5p	NM_005656	TMPRSS2	0.846153846154	3UTR
hsa-miR-6815-5p	NM_005656	TMPRSS2	0.846153846154	3UTR
hsa-miR-6816-5p	NM_005656	TMPRSS2	0.846153846154	3UTR
hsa-miR-6819-5p	NM_005656	TMPRSS2	0.846153846154	3UTR
hsa-miR-6820-3p	NM_005656	TMPRSS2	0.846153846154	3UTR
hsa-miR-6823-5p	NM_005656	TMPRSS2	0.846153846154	3UTR
hsa-miR-6824-5p	NM_005656	TMPRSS2	0.846153846154	3UTR

hsa-miR-6825-5p	NM_005656	TMPRSS2	0.846153846154	3UTR
hsa-miR-6831-5p	NM_005656	TMPRSS2	0.846153846154	3UTR
hsa-miR-6832-5p	NM_005656	TMPRSS2	0.846153846154	3UTR
hsa-miR-6833-3p	NM_005656	TMPRSS2	0.846153846154	3UTR
hsa-miR-6834-5p	NM_005656	TMPRSS2	0.846153846154	3UTR
hsa-miR-6835-5p	NM_005656	TMPRSS2	0.846153846154	3UTR
hsa-miR-6835-3p	NM_005656	TMPRSS2	0.846153846154	3UTR
hsa-miR-6780b-5p	NM_005656	TMPRSS2	0.846153846154	3UTR
hsa-miR-6780b-5p	NM_005656	TMPRSS2	0.846153846154	3UTR
hsa-miR-6836-3p	NM_005656	TMPRSS2	0.846153846154	3UTR
hsa-miR-6845-5p	NM_005656	TMPRSS2	0.846153846154	3UTR
hsa-miR-6845-3p	NM_005656	TMPRSS2	0.846153846154	3UTR
hsa-miR-6851-5p	NM_005656	TMPRSS2	0.846153846154	3UTR
hsa-miR-6853-5p	NM_005656	TMPRSS2	0.846153846154	3UTR
hsa-miR-6853-3p	NM_005656	TMPRSS2	0.846153846154	3UTR
hsa-miR-6856-3p	NM_005656	TMPRSS2	0.846153846154	3UTR
hsa-miR-6858-5p	NM_005656	TMPRSS2	0.846153846154	3UTR
hsa-miR-6859-5p	NM_005656	TMPRSS2	0.846153846154	3UTR
hsa-miR-6769b-3p	NM_005656	TMPRSS2	0.846153846154	3UTR
hsa-miR-6865-5p	NM_005656	TMPRSS2	0.846153846154	3UTR
hsa-miR-6871-5p	NM_005656	TMPRSS2	0.846153846154	3UTR
hsa-miR-6872-5p	NM_005656	TMPRSS2	0.846153846154	3UTR
hsa-miR-6877-5p	NM_005656	TMPRSS2	0.846153846154	3UTR
hsa-miR-6886-5p	NM_005656	TMPRSS2	0.846153846154	3UTR
hsa-miR-6886-3p	NM_005656	TMPRSS2	0.846153846154	3UTR
hsa-miR-6888-3p	NM_005656	TMPRSS2	0.846153846154	3UTR
hsa-miR-7107-5p	NM_005656	TMPRSS2	0.846153846154	3UTR
hsa-miR-7109-5p	NM_005656	TMPRSS2	0.846153846154	3UTR
hsa-miR-7150	NM_005656	TMPRSS2	0.846153846154	3UTR
hsa-miR-7152-3p	NM_005656	TMPRSS2	0.846153846154	3UTR
hsa-miR-7153-3p	NM_005656	TMPRSS2	0.846153846154	3UTR
hsa-miR-7154-5p	NM_005656	TMPRSS2	0.846153846154	3UTR
hsa-miR-7157-5p	NM_005656	TMPRSS2	0.846153846154	3UTR
hsa-miR-7160-5p	NM_005656	TMPRSS2	0.846153846154	3UTR
hsa-miR-7160-3p	NM_005656	TMPRSS2	0.846153846154	3UTR
hsa-miR-7702	NM_005656	TMPRSS2	0.846153846154	3UTR
hsa-miR-7703	NM_005656	TMPRSS2	0.846153846154	3UTR
hsa-miR-7845-5p	NM_005656	TMPRSS2	0.846153846154	3UTR
hsa-miR-7845-5p	NM_005656	TMPRSS2	0.846153846154	3UTR
hsa-miR-7846-3p	NM_005656	TMPRSS2	0.846153846154	3UTR
hsa-miR-7856-5p	NM_005656	TMPRSS2	0.846153846154	3UTR
hsa-miR-8063	NM_005656	TMPRSS2	0.846153846154	3UTR
hsa-miR-8065	NM_005656	TMPRSS2	0.846153846154	3UTR
hsa-miR-8078	NM_005656	TMPRSS2	0.846153846154	3UTR
hsa-miR-8085	NM_005656	TMPRSS2	0.846153846154	3UTR
hsa-miR-8087	NM_005656	TMPRSS2	0.846153846154	3UTR
hsa-miR-9500	NM_005656	TMPRSS2	0.846153846154	3UTR

hsa-miR-9903	NM_005656	TMPRSS2	0.846153846154	3UTR
hsa-miR-10226	NM_005656	TMPRSS2	0.846153846154	3UTR
hsa-miR-10394-5p	NM_005656	TMPRSS2	0.846153846154	3UTR
hsa-miR-10395-5p	NM_005656	TMPRSS2	0.846153846154	3UTR
hsa-miR-10399-3p	NM_005656	TMPRSS2	0.846153846154	3UTR
hsa-miR-11399	NM_005656	TMPRSS2	0.846153846154	3UTR
hsa-miR-3085-5p	NM_005656	TMPRSS2	0.846153846154	3UTR
hsa-miR-12114	NM_005656	TMPRSS2	0.846153846154	3UTR
hsa-miR-12122	NM_005656	TMPRSS2	0.846153846154	3UTR
hsa-miR-12122	NM_005656	TMPRSS2	0.846153846154	3UTR
hsa-miR-12124	NM_005656	TMPRSS2	0.846153846154	3UTR
hsa-miR-12127	NM_005656	TMPRSS2	0.846153846154	3UTR
hsa-miR-12128	NM_005656	TMPRSS2	0.846153846154	3UTR
hsa-miR-16-1-3p	NM_001135099	TMPRSS2	0.846153846154	3UTR
hsa-miR-17-5p	NM_001135099	TMPRSS2	0.846153846154	3UTR
hsa-miR-24-3p	NM_001135099	TMPRSS2	0.846153846154	3UTR
hsa-miR-25-5p	NM_001135099	TMPRSS2	0.846153846154	3UTR
hsa-miR-28-5p	NM_001135099	TMPRSS2	0.846153846154	3UTR
hsa-miR-28-3p	NM_001135099	TMPRSS2	0.846153846154	3UTR
hsa-miR-99a-3p	NM_001135099	TMPRSS2	0.846153846154	3UTR
hsa-miR-103a-1-5p	NM_001135099	TMPRSS2	0.846153846154	3UTR
hsa-miR-105-3p	NM_001135099	TMPRSS2	0.846153846154	3UTR
hsa-miR-106a-5p	NM_001135099	TMPRSS2	0.846153846154	3UTR
hsa-miR-148a-3p	NM_001135099	TMPRSS2	0.846153846154	3UTR
hsa-miR-148a-3p	NM_001135099	TMPRSS2	0.846153846154	3UTR
hsa-miR-30c-2-3p	NM_001135099	TMPRSS2	0.846153846154	3UTR
hsa-miR-30c-2-3p	NM_001135099	TMPRSS2	0.846153846154	3UTR
hsa-miR-139-5p	NM_001135099	TMPRSS2	0.846153846154	3UTR
hsa-miR-139-5p	NM_001135099	TMPRSS2	0.846153846154	3UTR
hsa-miR-7-1-3p	NM_001135099	TMPRSS2	0.846153846154	3UTR
hsa-miR-34a-5p	NM_001135099	TMPRSS2	0.846153846154	3UTR
hsa-miR-34a-5p	NM_001135099	TMPRSS2	0.846153846154	3UTR
hsa-miR-181a-5p	NM_001135099	TMPRSS2	0.846153846154	3UTR
hsa-miR-181c-3p	NM_001135099	TMPRSS2	0.846153846154	3UTR
hsa-miR-183-3p	NM_001135099	TMPRSS2	0.846153846154	3UTR
hsa-miR-210-5p	NM_001135099	TMPRSS2	0.846153846154	3UTR
hsa-miR-212-5p	NM_001135099	TMPRSS2	0.846153846154	3UTR
hsa-miR-214-3p	NM_001135099	TMPRSS2	0.846153846154	3UTR
hsa-miR-217-5p	NM_001135099	TMPRSS2	0.846153846154	3UTR
hsa-miR-219a-1-3p	NM_001135099	TMPRSS2	0.846153846154	3UTR
hsa-miR-222-3p	NM_001135099	TMPRSS2	0.846153846154	3UTR
hsa-miR-224-3p	NM_001135099	TMPRSS2	0.846153846154	3UTR
hsa-miR-224-3p	NM_001135099	TMPRSS2	0.846153846154	3UTR
hsa-let-7g-3p	NM_001135099	TMPRSS2	0.846153846154	3UTR
hsa-miR-23b-5p	NM_001135099	TMPRSS2	0.846153846154	3UTR
hsa-miR-27b-3p	NM_001135099	TMPRSS2	0.846153846154	3UTR
hsa-miR-122-3p	NM_001135099	TMPRSS2	0.846153846154	3UTR

hsa-miR-122-3p	NM_001135099	TMPRSS2	0.846153846154	3UTR
hsa-miR-128-3p	NM_001135099	TMPRSS2	0.846153846154	3UTR
hsa-miR-130a-5p	NM_001135099	TMPRSS2	0.846153846154	3UTR
hsa-miR-135a-2-3p	NM_001135099	TMPRSS2	0.846153846154	3UTR
hsa-miR-145-5p	NM_001135099	TMPRSS2	0.846153846154	3UTR
hsa-miR-9-3p	NM_001135099	TMPRSS2	0.846153846154	3UTR
hsa-miR-125a-5p	NM_001135099	TMPRSS2	0.846153846154	3UTR
hsa-miR-138-1-3p	NM_001135099	TMPRSS2	0.846153846154	3UTR
hsa-miR-150-5p	NM_001135099	TMPRSS2	0.846153846154	3UTR
hsa-miR-185-5p	NM_001135099	TMPRSS2	0.846153846154	3UTR
hsa-miR-185-3p	NM_001135099	TMPRSS2	0.846153846154	3UTR
hsa-miR-188-5p	NM_001135099	TMPRSS2	0.846153846154	3UTR
hsa-miR-320a-5p	NM_001135099	TMPRSS2	0.846153846154	3UTR
hsa-miR-155-5p	NM_001135099	TMPRSS2	0.846153846154	3UTR
hsa-miR-128-2-5p	NM_001135099	TMPRSS2	0.846153846154	3UTR
hsa-miR-29c-5p	NM_001135099	TMPRSS2	0.846153846154	3UTR
hsa-miR-299-3p	NM_001135099	TMPRSS2	0.846153846154	3UTR
hsa-miR-130b-5p	NM_001135099	TMPRSS2	0.846153846154	3UTR
hsa-miR-130b-5p	NM_001135099	TMPRSS2	0.846153846154	3UTR
hsa-miR-365a-5p	NM_001135099	TMPRSS2	0.846153846154	3UTR
hsa-miR-365b-5p	NM_001135099	TMPRSS2	0.846153846154	3UTR
hsa-miR-370-3p	NM_001135099	TMPRSS2	0.846153846154	3UTR
hsa-miR-371a-3p	NM_001135099	TMPRSS2	0.846153846154	3UTR
hsa-miR-373-5p	NM_001135099	TMPRSS2	0.846153846154	3UTR
hsa-miR-373-5p	NM_001135099	TMPRSS2	0.846153846154	3UTR
hsa-miR-373-3p	NM_001135099	TMPRSS2	0.846153846154	3UTR
hsa-miR-376a-5p	NM_001135099	TMPRSS2	0.846153846154	3UTR
hsa-miR-378a-3p	NM_001135099	TMPRSS2	0.846153846154	3UTR
hsa-miR-330-3p	NM_001135099	TMPRSS2	0.846153846154	3UTR
hsa-miR-326	NM_001135099	TMPRSS2	0.846153846154	3UTR
hsa-miR-324-3p	NM_001135099	TMPRSS2	0.846153846154	3UTR
hsa-miR-345-5p	NM_001135099	TMPRSS2	0.846153846154	3UTR
hsa-miR-196b-3p	NM_001135099	TMPRSS2	0.846153846154	3UTR
hsa-miR-425-5p	NM_001135099	TMPRSS2	0.846153846154	3UTR
hsa-miR-20b-5p	NM_001135099	TMPRSS2	0.846153846154	3UTR
hsa-miR-20b-5p	NM_001135099	TMPRSS2	0.846153846154	3UTR
hsa-miR-20b-5p	NM_001135099	TMPRSS2	0.846153846154	3UTR
hsa-miR-329-5p	NM_001135099	TMPRSS2	0.846153846154	3UTR
hsa-miR-409-5p	NM_001135099	TMPRSS2	0.846153846154	3UTR
hsa-miR-412-3p	NM_001135099	TMPRSS2	0.846153846154	3UTR
hsa-miR-483-5p	NM_001135099	TMPRSS2	0.846153846154	3UTR
hsa-miR-485-3p	NM_001135099	TMPRSS2	0.846153846154	3UTR
hsa-miR-487a-3p	NM_001135099	TMPRSS2	0.846153846154	3UTR
hsa-miR-490-3p	NM_001135099	TMPRSS2	0.846153846154	3UTR
hsa-miR-491-3p	NM_001135099	TMPRSS2	0.846153846154	3UTR
hsa-miR-432-5p	NM_001135099	TMPRSS2	0.846153846154	3UTR
hsa-miR-432-3p	NM_001135099	TMPRSS2	0.846153846154	3UTR

hsa-miR-494-3p	NM_001135099	TMPRSS2	0.846153846154	3UTR
hsa-miR-497-5p	NM_001135099	TMPRSS2	0.846153846154	3UTR
hsa-miR-498-5p	NM_001135099	TMPRSS2	0.846153846154	3UTR
hsa-miR-498-5p	NM_001135099	TMPRSS2	0.846153846154	3UTR
hsa-miR-520e-3p	NM_001135099	TMPRSS2	0.846153846154	3UTR
hsa-miR-515-5p	NM_001135099	TMPRSS2	0.846153846154	3UTR
hsa-miR-520f-5p	NM_001135099	TMPRSS2	0.846153846154	3UTR
hsa-miR-525-3p	NM_001135099	TMPRSS2	0.846153846154	3UTR
hsa-miR-523-3p	NM_001135099	TMPRSS2	0.846153846154	3UTR
hsa-miR-523-3p	NM_001135099	TMPRSS2	0.846153846154	3UTR
hsa-miR-520b-3p	NM_001135099	TMPRSS2	0.846153846154	3UTR
hsa-miR-526a-5p	NM_001135099	TMPRSS2	0.846153846154	3UTR
hsa-miR-520c-5p	NM_001135099	TMPRSS2	0.846153846154	3UTR
hsa-miR-518c-3p	NM_001135099	TMPRSS2	0.846153846154	3UTR
hsa-miR-516b-3p	NM_001135099	TMPRSS2	0.846153846154	3UTR
hsa-miR-518a-5p	NM_001135099	TMPRSS2	0.846153846154	3UTR
hsa-miR-518d-5p	NM_001135099	TMPRSS2	0.846153846154	3UTR
hsa-miR-520h	NM_001135099	TMPRSS2	0.846153846154	3UTR
hsa-miR-522-3p	NM_001135099	TMPRSS2	0.846153846154	3UTR
hsa-miR-527	NM_001135099	TMPRSS2	0.846153846154	3UTR
hsa-miR-516a-3p	NM_001135099	TMPRSS2	0.846153846154	3UTR
hsa-miR-499a-3p	NM_001135099	TMPRSS2	0.846153846154	3UTR
hsa-miR-503-5p	NM_001135099	TMPRSS2	0.846153846154	3UTR
hsa-miR-503-5p	NM_001135099	TMPRSS2	0.846153846154	3UTR
hsa-miR-513a-5p	NM_001135099	TMPRSS2	0.846153846154	3UTR
hsa-miR-532-5p	NM_001135099	TMPRSS2	0.846153846154	3UTR
hsa-miR-551a	NM_001135099	TMPRSS2	0.846153846154	3UTR
hsa-miR-552-5p	NM_001135099	TMPRSS2	0.846153846154	3UTR
hsa-miR-554	NM_001135099	TMPRSS2	0.846153846154	3UTR
hsa-miR-558	NM_001135099	TMPRSS2	0.846153846154	3UTR
hsa-miR-564	NM_001135099	TMPRSS2	0.846153846154	3UTR
hsa-miR-571	NM_001135099	TMPRSS2	0.846153846154	3UTR
hsa-miR-584-5p	NM_001135099	TMPRSS2	0.846153846154	3UTR
hsa-miR-587	NM_001135099	TMPRSS2	0.846153846154	3UTR
hsa-miR-550a-3p	NM_001135099	TMPRSS2	0.846153846154	3UTR
hsa-miR-601	NM_001135099	TMPRSS2	0.846153846154	3UTR
hsa-miR-608	NM_001135099	TMPRSS2	0.846153846154	3UTR
hsa-miR-609	NM_001135099	TMPRSS2	0.846153846154	3UTR
hsa-miR-610	NM_001135099	TMPRSS2	0.846153846154	3UTR
hsa-miR-612	NM_001135099	TMPRSS2	0.846153846154	3UTR
hsa-miR-615-3p	NM_001135099	TMPRSS2	0.846153846154	3UTR
hsa-miR-616-3p	NM_001135099	TMPRSS2	0.846153846154	3UTR
hsa-miR-627-5p	NM_001135099	TMPRSS2	0.846153846154	3UTR
hsa-miR-635	NM_001135099	TMPRSS2	0.846153846154	3UTR
hsa-miR-643	NM_001135099	TMPRSS2	0.846153846154	3UTR
hsa-miR-644a	NM_001135099	TMPRSS2	0.846153846154	3UTR
hsa-miR-645	NM_001135099	TMPRSS2	0.846153846154	3UTR

hsa-miR-646	NM_001135099	TMPRSS2	0.846153846154	3UTR
hsa-miR-646	NM_001135099	TMPRSS2	0.846153846154	3UTR
hsa-miR-651-5p	NM_001135099	TMPRSS2	0.846153846154	3UTR
hsa-miR-449b-3p	NM_001135099	TMPRSS2	0.846153846154	3UTR
hsa-miR-449b-3p	NM_001135099	TMPRSS2	0.846153846154	3UTR
hsa-miR-657	NM_001135099	TMPRSS2	0.846153846154	3UTR
hsa-miR-658	NM_001135099	TMPRSS2	0.846153846154	3UTR
hsa-miR-542-5p	NM_001135099	TMPRSS2	0.846153846154	3UTR
hsa-miR-766-5p	NM_001135099	TMPRSS2	0.846153846154	3UTR
hsa-miR-670-5p	NM_001135099	TMPRSS2	0.846153846154	3UTR
hsa-miR-764	NM_001135099	TMPRSS2	0.846153846154	3UTR
hsa-miR-765	NM_001135099	TMPRSS2	0.846153846154	3UTR
hsa-miR-675-5p	NM_001135099	TMPRSS2	0.846153846154	3UTR
hsa-miR-450b-3p	NM_001135099	TMPRSS2	0.846153846154	3UTR
hsa-miR-874-3p	NM_001135099	TMPRSS2	0.846153846154	3UTR
hsa-miR-890	NM_001135099	TMPRSS2	0.846153846154	3UTR
hsa-miR-541-5p	NM_001135099	TMPRSS2	0.846153846154	3UTR
hsa-miR-875-5p	NM_001135099	TMPRSS2	0.846153846154	3UTR
hsa-miR-147b-3p	NM_001135099	TMPRSS2	0.846153846154	3UTR
hsa-miR-877-5p	NM_001135099	TMPRSS2	0.846153846154	3UTR
hsa-miR-887-5p	NM_001135099	TMPRSS2	0.846153846154	3UTR
hsa-miR-665	NM_001135099	TMPRSS2	0.846153846154	3UTR
hsa-miR-301b-5p	NM_001135099	TMPRSS2	0.846153846154	3UTR
hsa-miR-216b-5p	NM_001135099	TMPRSS2	0.846153846154	3UTR
hsa-miR-920	NM_001135099	TMPRSS2	0.846153846154	3UTR
hsa-miR-922	NM_001135099	TMPRSS2	0.846153846154	3UTR
hsa-miR-922	NM_001135099	TMPRSS2	0.846153846154	3UTR
hsa-miR-934	NM_001135099	TMPRSS2	0.846153846154	3UTR
hsa-miR-937-3p	NM_001135099	TMPRSS2	0.846153846154	3UTR
hsa-miR-943	NM_001135099	TMPRSS2	0.846153846154	3UTR
hsa-miR-1178-5p	NM_001135099	TMPRSS2	0.846153846154	3UTR
hsa-miR-1182	NM_001135099	TMPRSS2	0.846153846154	3UTR
hsa-miR-1231	NM_001135099	TMPRSS2	0.846153846154	3UTR
hsa-miR-1203	NM_001135099	TMPRSS2	0.846153846154	3UTR
hsa-miR-1286	NM_001135099	TMPRSS2	0.846153846154	3UTR
hsa-miR-548k	NM_001135099	TMPRSS2	0.846153846154	3UTR
hsa-miR-1249-5p	NM_001135099	TMPRSS2	0.846153846154	3UTR
hsa-miR-1249-5p	NM_001135099	TMPRSS2	0.846153846154	3UTR
hsa-miR-1251-5p	NM_001135099	TMPRSS2	0.846153846154	3UTR
hsa-miR-1253	NM_001135099	TMPRSS2	0.846153846154	3UTR
hsa-miR-1263	NM_001135099	TMPRSS2	0.846153846154	3UTR
hsa-miR-1270	NM_001135099	TMPRSS2	0.846153846154	3UTR
hsa-miR-1275	NM_001135099	TMPRSS2	0.846153846154	3UTR
hsa-miR-1281	NM_001135099	TMPRSS2	0.846153846154	3UTR
hsa-miR-513c-5p	NM_001135099	TMPRSS2	0.846153846154	3UTR
hsa-miR-1321	NM_001135099	TMPRSS2	0.846153846154	3UTR
hsa-miR-320d	NM_001135099	TMPRSS2	0.846153846154	3UTR

hsa-miR-1910-5p	NM_001135099	TMPRSS2	0.846153846154	3UTR
hsa-miR-1911-3p	NM_001135099	TMPRSS2	0.846153846154	3UTR
hsa-miR-1913	NM_001135099	TMPRSS2	0.846153846154	3UTR
hsa-miR-2114-5p	NM_001135099	TMPRSS2	0.846153846154	3UTR
hsa-miR-2114-5p	NM_001135099	TMPRSS2	0.846153846154	3UTR
hsa-miR-2117	NM_001135099	TMPRSS2	0.846153846154	3UTR
hsa-miR-2276-3p	NM_001135099	TMPRSS2	0.846153846154	3UTR
hsa-miR-2278	NM_001135099	TMPRSS2	0.846153846154	3UTR
hsa-miR-2909	NM_001135099	TMPRSS2	0.846153846154	3UTR
hsa-miR-3116	NM_001135099	TMPRSS2	0.846153846154	3UTR
hsa-miR-548s	NM_001135099	TMPRSS2	0.846153846154	3UTR
hsa-miR-3125	NM_001135099	TMPRSS2	0.846153846154	3UTR
hsa-miR-3130-3p	NM_001135099	TMPRSS2	0.846153846154	3UTR
hsa-miR-3132	NM_001135099	TMPRSS2	0.846153846154	3UTR
hsa-miR-378b	NM_001135099	TMPRSS2	0.846153846154	3UTR
hsa-miR-3139	NM_001135099	TMPRSS2	0.846153846154	3UTR
hsa-miR-3141	NM_001135099	TMPRSS2	0.846153846154	3UTR
hsa-miR-3141	NM_001135099	TMPRSS2	0.846153846154	3UTR
hsa-miR-1273c	NM_001135099	TMPRSS2	0.846153846154	3UTR
hsa-miR-3150a-5p	NM_001135099	TMPRSS2	0.846153846154	3UTR
hsa-miR-3157-3p	NM_001135099	TMPRSS2	0.846153846154	3UTR
hsa-miR-3162-5p	NM_001135099	TMPRSS2	0.846153846154	3UTR
hsa-miR-3162-5p	NM_001135099	TMPRSS2	0.846153846154	3UTR
hsa-miR-3163	NM_001135099	TMPRSS2	0.846153846154	3UTR
hsa-miR-3164	NM_001135099	TMPRSS2	0.846153846154	3UTR
hsa-miR-3170	NM_001135099	TMPRSS2	0.846153846154	3UTR
hsa-miR-1193	NM_001135099	TMPRSS2	0.846153846154	3UTR
hsa-miR-3174	NM_001135099	TMPRSS2	0.846153846154	3UTR
hsa-miR-3184-3p	NM_001135099	TMPRSS2	0.846153846154	3UTR
hsa-miR-3189-5p	NM_001135099	TMPRSS2	0.846153846154	3UTR
hsa-miR-3189-3p	NM_001135099	TMPRSS2	0.846153846154	3UTR
hsa-miR-3192-3p	NM_001135099	TMPRSS2	0.846153846154	3UTR
hsa-miR-3197	NM_001135099	TMPRSS2	0.846153846154	3UTR
hsa-miR-378c	NM_001135099	TMPRSS2	0.846153846154	3UTR
hsa-miR-4294	NM_001135099	TMPRSS2	0.846153846154	3UTR
hsa-miR-4306	NM_001135099	TMPRSS2	0.846153846154	3UTR
hsa-miR-4313	NM_001135099	TMPRSS2	0.846153846154	3UTR
hsa-miR-4320	NM_001135099	TMPRSS2	0.846153846154	3UTR
hsa-miR-4320	NM_001135099	TMPRSS2	0.846153846154	3UTR
hsa-miR-4317	NM_001135099	TMPRSS2	0.846153846154	3UTR
hsa-miR-4259	NM_001135099	TMPRSS2	0.846153846154	3UTR
hsa-miR-4266	NM_001135099	TMPRSS2	0.846153846154	3UTR
hsa-miR-4269	NM_001135099	TMPRSS2	0.846153846154	3UTR
hsa-miR-4271	NM_001135099	TMPRSS2	0.846153846154	3UTR
hsa-miR-4271	NM_001135099	TMPRSS2	0.846153846154	3UTR
hsa-miR-4273	NM_001135099	TMPRSS2	0.846153846154	3UTR
hsa-miR-4279	NM_001135099	TMPRSS2	0.846153846154	3UTR

hsa-miR-4278	NM_001135099	TMPRSS2	0.846153846154	3UTR
hsa-miR-4285	NM_001135099	TMPRSS2	0.846153846154	3UTR
hsa-miR-4289	NM_001135099	TMPRSS2	0.846153846154	3UTR
hsa-miR-4291	NM_001135099	TMPRSS2	0.846153846154	3UTR
hsa-miR-500b-5p	NM_001135099	TMPRSS2	0.846153846154	3UTR
hsa-miR-500b-5p	NM_001135099	TMPRSS2	0.846153846154	3UTR
hsa-miR-3610	NM_001135099	TMPRSS2	0.846153846154	3UTR
hsa-miR-3610	NM_001135099	TMPRSS2	0.846153846154	3UTR
hsa-miR-3614-3p	NM_001135099	TMPRSS2	0.846153846154	3UTR
hsa-miR-3617-3p	NM_001135099	TMPRSS2	0.846153846154	3UTR
hsa-miR-23c	NM_001135099	TMPRSS2	0.846153846154	3UTR
hsa-miR-3650	NM_001135099	TMPRSS2	0.846153846154	3UTR
hsa-miR-3654	NM_001135099	TMPRSS2	0.846153846154	3UTR
hsa-miR-3664-5p	NM_001135099	TMPRSS2	0.846153846154	3UTR
hsa-miR-3665	NM_001135099	TMPRSS2	0.846153846154	3UTR
hsa-miR-3675-3p	NM_001135099	TMPRSS2	0.846153846154	3UTR
hsa-miR-3678-3p	NM_001135099	TMPRSS2	0.846153846154	3UTR
hsa-miR-3680-5p	NM_001135099	TMPRSS2	0.846153846154	3UTR
hsa-miR-3680-3p	NM_001135099	TMPRSS2	0.846153846154	3UTR
hsa-miR-3689a-5p	NM_001135099	TMPRSS2	0.846153846154	3UTR
hsa-miR-3689b-5p	NM_001135099	TMPRSS2	0.846153846154	3UTR
hsa-miR-3689b-3p	NM_001135099	TMPRSS2	0.846153846154	3UTR
hsa-miR-3916	NM_001135099	TMPRSS2	0.846153846154	3UTR
hsa-miR-3924	NM_001135099	TMPRSS2	0.846153846154	3UTR
hsa-miR-3925-5p	NM_001135099	TMPRSS2	0.846153846154	3UTR
hsa-miR-3934-5p	NM_001135099	TMPRSS2	0.846153846154	3UTR
hsa-miR-374c-3p	NM_001135099	TMPRSS2	0.846153846154	3UTR
hsa-miR-550b-2-5p	NM_001135099	TMPRSS2	0.846153846154	3UTR
hsa-miR-378e	NM_001135099	TMPRSS2	0.846153846154	3UTR
hsa-miR-4421	NM_001135099	TMPRSS2	0.846153846154	3UTR
hsa-miR-4421	NM_001135099	TMPRSS2	0.846153846154	3UTR
hsa-miR-378g	NM_001135099	TMPRSS2	0.846153846154	3UTR
hsa-miR-4424	NM_001135099	TMPRSS2	0.846153846154	3UTR
hsa-miR-4433a-5p	NM_001135099	TMPRSS2	0.846153846154	3UTR
hsa-miR-4433a-5p	NM_001135099	TMPRSS2	0.846153846154	3UTR
hsa-miR-4433a-3p	NM_001135099	TMPRSS2	0.846153846154	3UTR
hsa-miR-4439	NM_001135099	TMPRSS2	0.846153846154	3UTR
hsa-miR-4448	NM_001135099	TMPRSS2	0.846153846154	3UTR
hsa-miR-378h	NM_001135099	TMPRSS2	0.846153846154	3UTR
hsa-miR-4465	NM_001135099	TMPRSS2	0.846153846154	3UTR
hsa-miR-4472	NM_001135099	TMPRSS2	0.846153846154	3UTR
hsa-miR-4473	NM_001135099	TMPRSS2	0.846153846154	3UTR
hsa-miR-4474-3p	NM_001135099	TMPRSS2	0.846153846154	3UTR
hsa-miR-4478	NM_001135099	TMPRSS2	0.846153846154	3UTR
hsa-miR-3689c	NM_001135099	TMPRSS2	0.846153846154	3UTR
hsa-miR-3689e	NM_001135099	TMPRSS2	0.846153846154	3UTR
hsa-miR-3155b	NM_001135099	TMPRSS2	0.846153846154	3UTR

hsa-miR-4485-5p	NM_001135099	TMPRSS2	0.846153846154	3UTR
hsa-miR-4487	NM_001135099	TMPRSS2	0.846153846154	3UTR
hsa-miR-4487	NM_001135099	TMPRSS2	0.846153846154	3UTR
hsa-miR-4488	NM_001135099	TMPRSS2	0.846153846154	3UTR
hsa-miR-2392	NM_001135099	TMPRSS2	0.846153846154	3UTR
hsa-miR-4514	NM_001135099	TMPRSS2	0.846153846154	3UTR
hsa-miR-4520-5p	NM_001135099	TMPRSS2	0.846153846154	3UTR
hsa-miR-4525	NM_001135099	TMPRSS2	0.846153846154	3UTR
hsa-miR-4533	NM_001135099	TMPRSS2	0.846153846154	3UTR
hsa-miR-1587	NM_001135099	TMPRSS2	0.846153846154	3UTR
hsa-miR-4540	NM_001135099	TMPRSS2	0.846153846154	3UTR
hsa-miR-3975	NM_001135099	TMPRSS2	0.846153846154	3UTR
hsa-miR-3976	NM_001135099	TMPRSS2	0.846153846154	3UTR
hsa-miR-4632-5p	NM_001135099	TMPRSS2	0.846153846154	3UTR
hsa-miR-4633-5p	NM_001135099	TMPRSS2	0.846153846154	3UTR
hsa-miR-4635	NM_001135099	TMPRSS2	0.846153846154	3UTR
hsa-miR-4640-5p	NM_001135099	TMPRSS2	0.846153846154	3UTR
hsa-miR-4642	NM_001135099	TMPRSS2	0.846153846154	3UTR
hsa-miR-4644	NM_001135099	TMPRSS2	0.846153846154	3UTR
hsa-miR-4645-5p	NM_001135099	TMPRSS2	0.846153846154	3UTR
hsa-miR-4647	NM_001135099	TMPRSS2	0.846153846154	3UTR
hsa-miR-4648	NM_001135099	TMPRSS2	0.846153846154	3UTR
hsa-miR-4669	NM_001135099	TMPRSS2	0.846153846154	3UTR
hsa-miR-4671-3p	NM_001135099	TMPRSS2	0.846153846154	3UTR
hsa-miR-4681	NM_001135099	TMPRSS2	0.846153846154	3UTR
hsa-miR-4682	NM_001135099	TMPRSS2	0.846153846154	3UTR
hsa-miR-4685-5p	NM_001135099	TMPRSS2	0.846153846154	3UTR
hsa-miR-4685-5p	NM_001135099	TMPRSS2	0.846153846154	3UTR
hsa-miR-1343-3p	NM_001135099	TMPRSS2	0.846153846154	3UTR
hsa-miR-4690-3p	NM_001135099	TMPRSS2	0.846153846154	3UTR
hsa-miR-4691-5p	NM_001135099	TMPRSS2	0.846153846154	3UTR
hsa-miR-4695-3p	NM_001135099	TMPRSS2	0.846153846154	3UTR
hsa-miR-4696	NM_001135099	TMPRSS2	0.846153846154	3UTR
hsa-miR-4704-5p	NM_001135099	TMPRSS2	0.846153846154	3UTR
hsa-miR-4704-5p	NM_001135099	TMPRSS2	0.846153846154	3UTR
hsa-miR-4705	NM_001135099	TMPRSS2	0.846153846154	3UTR
hsa-miR-4706	NM_001135099	TMPRSS2	0.846153846154	3UTR
hsa-miR-4708-3p	NM_001135099	TMPRSS2	0.846153846154	3UTR
hsa-miR-203b-5p	NM_001135099	TMPRSS2	0.846153846154	3UTR
hsa-miR-4712-3p	NM_001135099	TMPRSS2	0.846153846154	3UTR
hsa-miR-4713-5p	NM_001135099	TMPRSS2	0.846153846154	3UTR
hsa-miR-4714-5p	NM_001135099	TMPRSS2	0.846153846154	3UTR
hsa-miR-4716-3p	NM_001135099	TMPRSS2	0.846153846154	3UTR
hsa-miR-3529-5p	NM_001135099	TMPRSS2	0.846153846154	3UTR
hsa-miR-4721	NM_001135099	TMPRSS2	0.846153846154	3UTR
hsa-miR-4724-5p	NM_001135099	TMPRSS2	0.846153846154	3UTR
hsa-miR-4731-5p	NM_001135099	TMPRSS2	0.846153846154	3UTR

hsa-miR-4732-5p	NM_001135099	TMPRSS2	0.846153846154	3UTR
hsa-miR-4737	NM_001135099	TMPRSS2	0.846153846154	3UTR
hsa-miR-3064-3p	NM_001135099	TMPRSS2	0.846153846154	3UTR
hsa-miR-4743-5p	NM_001135099	TMPRSS2	0.846153846154	3UTR
hsa-miR-4747-5p	NM_001135099	TMPRSS2	0.846153846154	3UTR
hsa-miR-4747-3p	NM_001135099	TMPRSS2	0.846153846154	3UTR
hsa-miR-4748	NM_001135099	TMPRSS2	0.846153846154	3UTR
hsa-miR-4748	NM_001135099	TMPRSS2	0.846153846154	3UTR
hsa-miR-4750-3p	NM_001135099	TMPRSS2	0.846153846154	3UTR
hsa-miR-4752	NM_001135099	TMPRSS2	0.846153846154	3UTR
hsa-miR-4753-3p	NM_001135099	TMPRSS2	0.846153846154	3UTR
hsa-miR-4761-5p	NM_001135099	TMPRSS2	0.846153846154	3UTR
hsa-miR-4768-5p	NM_001135099	TMPRSS2	0.846153846154	3UTR
hsa-miR-4771	NM_001135099	TMPRSS2	0.846153846154	3UTR
hsa-miR-4775	NM_001135099	TMPRSS2	0.846153846154	3UTR
hsa-miR-4779	NM_001135099	TMPRSS2	0.846153846154	3UTR
hsa-miR-4780	NM_001135099	TMPRSS2	0.846153846154	3UTR
hsa-miR-4436b-3p	NM_001135099	TMPRSS2	0.846153846154	3UTR
hsa-miR-4785	NM_001135099	TMPRSS2	0.846153846154	3UTR
hsa-miR-4785	NM_001135099	TMPRSS2	0.846153846154	3UTR
hsa-miR-1245b-5p	NM_001135099	TMPRSS2	0.846153846154	3UTR
hsa-miR-2467-3p	NM_001135099	TMPRSS2	0.846153846154	3UTR
hsa-miR-4786-5p	NM_001135099	TMPRSS2	0.846153846154	3UTR
hsa-miR-4790-5p	NM_001135099	TMPRSS2	0.846153846154	3UTR
hsa-miR-4793-5p	NM_001135099	TMPRSS2	0.846153846154	3UTR
hsa-miR-4794	NM_001135099	TMPRSS2	0.846153846154	3UTR
hsa-miR-4796-3p	NM_001135099	TMPRSS2	0.846153846154	3UTR
hsa-miR-4797-3p	NM_001135099	TMPRSS2	0.846153846154	3UTR
hsa-miR-4802-5p	NM_001135099	TMPRSS2	0.846153846154	3UTR
hsa-miR-4802-3p	NM_001135099	TMPRSS2	0.846153846154	3UTR
hsa-miR-5001-3p	NM_001135099	TMPRSS2	0.846153846154	3UTR
hsa-miR-5003-3p	NM_001135099	TMPRSS2	0.846153846154	3UTR
hsa-miR-5006-3p	NM_001135099	TMPRSS2	0.846153846154	3UTR
hsa-miR-5010-5p	NM_001135099	TMPRSS2	0.846153846154	3UTR
hsa-miR-5092	NM_001135099	TMPRSS2	0.846153846154	3UTR
hsa-miR-5187-3p	NM_001135099	TMPRSS2	0.846153846154	3UTR
hsa-miR-5193	NM_001135099	TMPRSS2	0.846153846154	3UTR
hsa-miR-5193	NM_001135099	TMPRSS2	0.846153846154	3UTR
hsa-miR-5194	NM_001135099	TMPRSS2	0.846153846154	3UTR
hsa-miR-5581-3p	NM_001135099	TMPRSS2	0.846153846154	3UTR
hsa-miR-548at-5p	NM_001135099	TMPRSS2	0.846153846154	3UTR
hsa-miR-5584-3p	NM_001135099	TMPRSS2	0.846153846154	3UTR
hsa-miR-5587-5p	NM_001135099	TMPRSS2	0.846153846154	3UTR
hsa-miR-1295b-5p	NM_001135099	TMPRSS2	0.846153846154	3UTR
hsa-miR-5588-3p	NM_001135099	TMPRSS2	0.846153846154	3UTR
hsa-miR-5591-5p	NM_001135099	TMPRSS2	0.846153846154	3UTR
hsa-miR-5690	NM_001135099	TMPRSS2	0.846153846154	3UTR

hsa-miR-5691	NM_001135099	TMPRSS2	0.846153846154	3UTR
hsa-miR-5699-5p	NM_001135099	TMPRSS2	0.846153846154	3UTR
hsa-miR-5703	NM_001135099	TMPRSS2	0.846153846154	3UTR
hsa-miR-6072	NM_001135099	TMPRSS2	0.846153846154	3UTR
hsa-miR-6085	NM_001135099	TMPRSS2	0.846153846154	3UTR
hsa-miR-6086	NM_001135099	TMPRSS2	0.846153846154	3UTR
hsa-miR-6124	NM_001135099	TMPRSS2	0.846153846154	3UTR
hsa-miR-6124	NM_001135099	TMPRSS2	0.846153846154	3UTR
hsa-miR-378j	NM_001135099	TMPRSS2	0.846153846154	3UTR
hsa-miR-6132	NM_001135099	TMPRSS2	0.846153846154	3UTR
hsa-miR-6132	NM_001135099	TMPRSS2	0.846153846154	3UTR
hsa-miR-6165	NM_001135099	TMPRSS2	0.846153846154	3UTR
hsa-miR-6499-3p	NM_001135099	TMPRSS2	0.846153846154	3UTR
hsa-miR-6501-3p	NM_001135099	TMPRSS2	0.846153846154	3UTR
hsa-miR-6504-5p	NM_001135099	TMPRSS2	0.846153846154	3UTR
hsa-miR-6505-3p	NM_001135099	TMPRSS2	0.846153846154	3UTR
hsa-miR-6509-5p	NM_001135099	TMPRSS2	0.846153846154	3UTR
hsa-miR-6509-3p	NM_001135099	TMPRSS2	0.846153846154	3UTR
hsa-miR-6510-3p	NM_001135099	TMPRSS2	0.846153846154	3UTR
hsa-miR-6511a-5p	NM_001135099	TMPRSS2	0.846153846154	3UTR
hsa-miR-6513-3p	NM_001135099	TMPRSS2	0.846153846154	3UTR
hsa-miR-6721-5p	NM_001135099	TMPRSS2	0.846153846154	3UTR
hsa-miR-892c-5p	NM_001135099	TMPRSS2	0.846153846154	3UTR
hsa-miR-6726-3p	NM_001135099	TMPRSS2	0.846153846154	3UTR
hsa-miR-6727-3p	NM_001135099	TMPRSS2	0.846153846154	3UTR
hsa-miR-6729-5p	NM_001135099	TMPRSS2	0.846153846154	3UTR
hsa-miR-6734-5p	NM_001135099	TMPRSS2	0.846153846154	3UTR
hsa-miR-6734-3p	NM_001135099	TMPRSS2	0.846153846154	3UTR
hsa-miR-6738-5p	NM_001135099	TMPRSS2	0.846153846154	3UTR
hsa-miR-6739-3p	NM_001135099	TMPRSS2	0.846153846154	3UTR
hsa-miR-6746-5p	NM_001135099	TMPRSS2	0.846153846154	3UTR
hsa-miR-6750-3p	NM_001135099	TMPRSS2	0.846153846154	3UTR
hsa-miR-6752-5p	NM_001135099	TMPRSS2	0.846153846154	3UTR
hsa-miR-6753-5p	NM_001135099	TMPRSS2	0.846153846154	3UTR
hsa-miR-6753-5p	NM_001135099	TMPRSS2	0.846153846154	3UTR
hsa-miR-6754-5p	NM_001135099	TMPRSS2	0.846153846154	3UTR
hsa-miR-6757-5p	NM_001135099	TMPRSS2	0.846153846154	3UTR
hsa-miR-6759-5p	NM_001135099	TMPRSS2	0.846153846154	3UTR
hsa-miR-6760-5p	NM_001135099	TMPRSS2	0.846153846154	3UTR
hsa-miR-6762-5p	NM_001135099	TMPRSS2	0.846153846154	3UTR
hsa-miR-6767-3p	NM_001135099	TMPRSS2	0.846153846154	3UTR
hsa-miR-6768-3p	NM_001135099	TMPRSS2	0.846153846154	3UTR
hsa-miR-6769a-5p	NM_001135099	TMPRSS2	0.846153846154	3UTR
hsa-miR-6771-3p	NM_001135099	TMPRSS2	0.846153846154	3UTR
hsa-miR-6772-5p	NM_001135099	TMPRSS2	0.846153846154	3UTR
hsa-miR-6778-3p	NM_001135099	TMPRSS2	0.846153846154	3UTR
hsa-miR-6779-5p	NM_001135099	TMPRSS2	0.846153846154	3UTR

hsa-miR-6781-5p	NM_001135099	TMPRSS2	0.846153846154	3UTR
hsa-miR-6784-3p	NM_001135099	TMPRSS2	0.846153846154	3UTR
hsa-miR-6787-3p	NM_001135099	TMPRSS2	0.846153846154	3UTR
hsa-miR-6788-3p	NM_001135099	TMPRSS2	0.846153846154	3UTR
hsa-miR-6792-5p	NM_001135099	TMPRSS2	0.846153846154	3UTR
hsa-miR-6792-3p	NM_001135099	TMPRSS2	0.846153846154	3UTR
hsa-miR-6793-3p	NM_001135099	TMPRSS2	0.846153846154	3UTR
hsa-miR-6794-5p	NM_001135099	TMPRSS2	0.846153846154	3UTR
hsa-miR-6796-5p	NM_001135099	TMPRSS2	0.846153846154	3UTR
hsa-miR-6797-5p	NM_001135099	TMPRSS2	0.846153846154	3UTR
hsa-miR-6797-3p	NM_001135099	TMPRSS2	0.846153846154	3UTR
hsa-miR-6801-5p	NM_001135099	TMPRSS2	0.846153846154	3UTR
hsa-miR-6803-5p	NM_001135099	TMPRSS2	0.846153846154	3UTR
hsa-miR-6804-5p	NM_001135099	TMPRSS2	0.846153846154	3UTR
hsa-miR-6811-5p	NM_001135099	TMPRSS2	0.846153846154	3UTR
hsa-miR-6814-5p	NM_001135099	TMPRSS2	0.846153846154	3UTR
hsa-miR-6815-5p	NM_001135099	TMPRSS2	0.846153846154	3UTR
hsa-miR-6826-5p	NM_001135099	TMPRSS2	0.846153846154	3UTR
hsa-miR-6828-5p	NM_001135099	TMPRSS2	0.846153846154	3UTR
hsa-miR-6829-5p	NM_001135099	TMPRSS2	0.846153846154	3UTR
hsa-miR-6830-5p	NM_001135099	TMPRSS2	0.846153846154	3UTR
hsa-miR-6830-3p	NM_001135099	TMPRSS2	0.846153846154	3UTR
hsa-miR-6831-5p	NM_001135099	TMPRSS2	0.846153846154	3UTR
hsa-miR-6831-5p	NM_001135099	TMPRSS2	0.846153846154	3UTR
hsa-miR-6832-3p	NM_001135099	TMPRSS2	0.846153846154	3UTR
hsa-miR-6833-3p	NM_001135099	TMPRSS2	0.846153846154	3UTR
hsa-miR-6834-3p	NM_001135099	TMPRSS2	0.846153846154	3UTR
hsa-miR-6835-5p	NM_001135099	TMPRSS2	0.846153846154	3UTR
hsa-miR-6835-3p	NM_001135099	TMPRSS2	0.846153846154	3UTR
hsa-miR-6780b-5p	NM_001135099	TMPRSS2	0.846153846154	3UTR
hsa-miR-6780b-5p	NM_001135099	TMPRSS2	0.846153846154	3UTR
hsa-miR-6845-5p	NM_001135099	TMPRSS2	0.846153846154	3UTR
hsa-miR-6845-3p	NM_001135099	TMPRSS2	0.846153846154	3UTR
hsa-miR-6847-5p	NM_001135099	TMPRSS2	0.846153846154	3UTR
hsa-miR-6847-5p	NM_001135099	TMPRSS2	0.846153846154	3UTR
hsa-miR-6848-3p	NM_001135099	TMPRSS2	0.846153846154	3UTR
hsa-miR-6849-3p	NM_001135099	TMPRSS2	0.846153846154	3UTR
hsa-miR-6853-5p	NM_001135099	TMPRSS2	0.846153846154	3UTR
hsa-miR-6854-3p	NM_001135099	TMPRSS2	0.846153846154	3UTR
hsa-miR-6857-3p	NM_001135099	TMPRSS2	0.846153846154	3UTR
hsa-miR-6858-5p	NM_001135099	TMPRSS2	0.846153846154	3UTR
hsa-miR-6769b-5p	NM_001135099	TMPRSS2	0.846153846154	3UTR
hsa-miR-6865-5p	NM_001135099	TMPRSS2	0.846153846154	3UTR
hsa-miR-6866-3p	NM_001135099	TMPRSS2	0.846153846154	3UTR
hsa-miR-6867-3p	NM_001135099	TMPRSS2	0.846153846154	3UTR
hsa-miR-6871-5p	NM_001135099	TMPRSS2	0.846153846154	3UTR
hsa-miR-6872-5p	NM_001135099	TMPRSS2	0.846153846154	3UTR

hsa-miR-6876-5p	NM_001135099	TMPRSS2	0.846153846154	3UTR
hsa-miR-6877-5p	NM_001135099	TMPRSS2	0.846153846154	3UTR
hsa-miR-6877-5p	NM_001135099	TMPRSS2	0.846153846154	3UTR
hsa-miR-6878-5p	NM_001135099	TMPRSS2	0.846153846154	3UTR
hsa-miR-6882-5p	NM_001135099	TMPRSS2	0.846153846154	3UTR
hsa-miR-6883-5p	NM_001135099	TMPRSS2	0.846153846154	3UTR
hsa-miR-6885-5p	NM_001135099	TMPRSS2	0.846153846154	3UTR
hsa-miR-6886-5p	NM_001135099	TMPRSS2	0.846153846154	3UTR
hsa-miR-6890-5p	NM_001135099	TMPRSS2	0.846153846154	3UTR
hsa-miR-6893-5p	NM_001135099	TMPRSS2	0.846153846154	3UTR
hsa-miR-6893-3p	NM_001135099	TMPRSS2	0.846153846154	3UTR
hsa-miR-6894-5p	NM_001135099	TMPRSS2	0.846153846154	3UTR
hsa-miR-7107-5p	NM_001135099	TMPRSS2	0.846153846154	3UTR
hsa-miR-7111-5p	NM_001135099	TMPRSS2	0.846153846154	3UTR
hsa-miR-7113-3p	NM_001135099	TMPRSS2	0.846153846154	3UTR
hsa-miR-7150	NM_001135099	TMPRSS2	0.846153846154	3UTR
hsa-miR-7151-3p	NM_001135099	TMPRSS2	0.846153846154	3UTR
hsa-miR-7154-5p	NM_001135099	TMPRSS2	0.846153846154	3UTR
hsa-miR-7156-5p	NM_001135099	TMPRSS2	0.846153846154	3UTR
hsa-miR-7157-5p	NM_001135099	TMPRSS2	0.846153846154	3UTR
hsa-miR-7158-5p	NM_001135099	TMPRSS2	0.846153846154	3UTR
hsa-miR-7162-5p	NM_001135099	TMPRSS2	0.846153846154	3UTR
hsa-miR-7702	NM_001135099	TMPRSS2	0.846153846154	3UTR
hsa-miR-7706	NM_001135099	TMPRSS2	0.846153846154	3UTR
hsa-miR-7843-3p	NM_001135099	TMPRSS2	0.846153846154	3UTR
hsa-miR-1273h-3p	NM_001135099	TMPRSS2	0.846153846154	3UTR
hsa-miR-7845-5p	NM_001135099	TMPRSS2	0.846153846154	3UTR
hsa-miR-7845-5p	NM_001135099	TMPRSS2	0.846153846154	3UTR
hsa-miR-7851-3p	NM_001135099	TMPRSS2	0.846153846154	3UTR
hsa-miR-7854-3p	NM_001135099	TMPRSS2	0.846153846154	3UTR
hsa-miR-7856-5p	NM_001135099	TMPRSS2	0.846153846154	3UTR
hsa-miR-7974	NM_001135099	TMPRSS2	0.846153846154	3UTR
hsa-miR-8059	NM_001135099	TMPRSS2	0.846153846154	3UTR
hsa-miR-8060	NM_001135099	TMPRSS2	0.846153846154	3UTR
hsa-miR-8063	NM_001135099	TMPRSS2	0.846153846154	3UTR
hsa-miR-8065	NM_001135099	TMPRSS2	0.846153846154	3UTR
hsa-miR-8078	NM_001135099	TMPRSS2	0.846153846154	3UTR
hsa-miR-8082	NM_001135099	TMPRSS2	0.846153846154	3UTR
hsa-miR-8086	NM_001135099	TMPRSS2	0.846153846154	3UTR
hsa-miR-9901	NM_001135099	TMPRSS2	0.846153846154	3UTR
hsa-miR-10522-5p	NM_001135099	TMPRSS2	0.846153846154	3UTR
hsa-miR-11399	NM_001135099	TMPRSS2	0.846153846154	3UTR
hsa-miR-3085-5p	NM_001135099	TMPRSS2	0.846153846154	3UTR
hsa-miR-12114	NM_001135099	TMPRSS2	0.846153846154	3UTR
hsa-miR-12127	NM_001135099	TMPRSS2	0.846153846154	3UTR
hsa-miR-3674	NM_005656	TMPRSS2	0.858974358974	3UTR
hsa-miR-7153-3p	NM_005656	TMPRSS2	0.861538461538	3UTR

hsa-miR-7153-3p	NM_001135099	TMPRSS2	0.861538461538	3UTR
hsa-miR-4720-3p	NM_001135099	TMPRSS2	0.862637362637	3UTR
hsa-miR-133b	NM_005656	TMPRSS2	0.865384615385	3UTR
hsa-miR-302a-3p	NM_001135099	TMPRSS2	0.865384615385	3UTR
hsa-miR-4293	NM_001135099	TMPRSS2	0.865384615385	3UTR
hsa-miR-523-3p	NM_005656	TMPRSS2	0.871794871795	3UTR
hsa-miR-598-3p	NM_005656	TMPRSS2	0.871794871795	3UTR
hsa-miR-1207-5p	NM_005656	TMPRSS2	0.871794871795	3UTR
hsa-miR-1908-3p	NM_005656	TMPRSS2	0.871794871795	3UTR
hsa-miR-6749-5p	NM_005656	TMPRSS2	0.871794871795	3UTR
hsa-miR-6803-5p	NM_005656	TMPRSS2	0.871794871795	3UTR
hsa-miR-195-5p	NM_001135099	TMPRSS2	0.871794871795	3UTR
hsa-miR-381-3p	NM_001135099	TMPRSS2	0.871794871795	3UTR
hsa-miR-3945	NM_001135099	TMPRSS2	0.871794871795	3UTR
hsa-miR-4740-5p	NM_001135099	TMPRSS2	0.871794871795	3UTR
hsa-miR-6849-5p	NM_001135099	TMPRSS2	0.871794871795	3UTR
hsa-miR-7156-5p	NM_001135099	TMPRSS2	0.871794871795	3UTR
hsa-miR-10394-5p	NM_001135099	TMPRSS2	0.876923076923	3UTR
hsa-miR-608	NM_005656	TMPRSS2	0.884615384615	3UTR
hsa-miR-659-5p	NM_005656	TMPRSS2	0.884615384615	3UTR
hsa-miR-4314	NM_005656	TMPRSS2	0.884615384615	3UTR
hsa-miR-4270	NM_005656	TMPRSS2	0.884615384615	3UTR
hsa-miR-4290	NM_005656	TMPRSS2	0.884615384615	3UTR
hsa-miR-4430	NM_005656	TMPRSS2	0.884615384615	3UTR
hsa-miR-4689	NM_005656	TMPRSS2	0.884615384615	3UTR
hsa-miR-4720-3p	NM_005656	TMPRSS2	0.884615384615	3UTR
hsa-miR-4769-5p	NM_005656	TMPRSS2	0.884615384615	3UTR
hsa-miR-5094	NM_005656	TMPRSS2	0.884615384615	3UTR
hsa-miR-5588-5p	NM_005656	TMPRSS2	0.884615384615	3UTR
hsa-miR-5699-3p	NM_005656	TMPRSS2	0.884615384615	3UTR
hsa-miR-6132	NM_005656	TMPRSS2	0.884615384615	3UTR
hsa-miR-6501-3p	NM_005656	TMPRSS2	0.884615384615	3UTR
hsa-miR-6862-5p	NM_005656	TMPRSS2	0.884615384615	3UTR
hsa-miR-6870-5p	NM_005656	TMPRSS2	0.884615384615	3UTR
hsa-miR-6876-3p	NM_005656	TMPRSS2	0.884615384615	3UTR
hsa-miR-608	NM_001135099	TMPRSS2	0.884615384615	3UTR
hsa-miR-616-3p	NM_001135099	TMPRSS2	0.884615384615	3UTR
hsa-miR-4283	NM_001135099	TMPRSS2	0.884615384615	3UTR
hsa-miR-3663-5p	NM_001135099	TMPRSS2	0.884615384615	3UTR
hsa-miR-4430	NM_001135099	TMPRSS2	0.884615384615	3UTR
hsa-miR-5699-3p	NM_001135099	TMPRSS2	0.884615384615	3UTR
hsa-miR-6737-5p	NM_001135099	TMPRSS2	0.884615384615	3UTR
hsa-miR-6878-5p	NM_001135099	TMPRSS2	0.884615384615	3UTR
hsa-miR-3174	NM_005656	TMPRSS2	0.892307692308	3UTR
hsa-miR-3689a-3p	NM_001135099	TMPRSS2	0.892307692308	3UTR
hsa-miR-3929	NM_001135099	TMPRSS2	0.892307692308	3UTR
hsa-miR-520c-3p	NM_005656	TMPRSS2	0.897435897436	3UTR

hsa-miR-33b-3p	NM_005656	TMPRSS2	0.897435897436	3UTR
hsa-miR-6746-5p	NM_005656	TMPRSS2	0.897435897436	3UTR
hsa-miR-7114-5p	NM_005656	TMPRSS2	0.897435897436	3UTR
hsa-miR-141-3p	NM_001135099	TMPRSS2	0.897435897436	3UTR
hsa-miR-520c-3p	NM_001135099	TMPRSS2	0.897435897436	3UTR
hsa-miR-642b-3p	NM_001135099	TMPRSS2	0.897435897436	3UTR
hsa-miR-4802-5p	NM_001135099	TMPRSS2	0.897435897436	3UTR
hsa-miR-27b-5p	NM_005656	TMPRSS2	0.903846153846	3UTR
hsa-miR-6726-5p	NM_005656	TMPRSS2	0.903846153846	3UTR
hsa-miR-6726-5p	NM_001135099	TMPRSS2	0.903846153846	3UTR
hsa-miR-4769-5p	NM_001135099	TMPRSS2	0.910256410256	3UTR
hsa-miR-1251-5p	NM_005656	TMPRSS2	0.912087912088	3UTR
hsa-let-7a-5p	NM_005656	TMPRSS2	0.923076923077	3UTR
hsa-let-7a-2-3p	NM_005656	TMPRSS2	0.923076923077	3UTR
hsa-let-7c-5p	NM_005656	TMPRSS2	0.923076923077	3UTR
hsa-let-7e-5p	NM_005656	TMPRSS2	0.923076923077	3UTR
hsa-let-7f-5p	NM_005656	TMPRSS2	0.923076923077	3UTR
hsa-miR-17-5p	NM_005656	TMPRSS2	0.923076923077	3UTR
hsa-miR-17-5p	NM_005656	TMPRSS2	0.923076923077	3UTR
hsa-miR-17-5p	NM_005656	TMPRSS2	0.923076923077	3UTR
hsa-miR-21-3p	NM_005656	TMPRSS2	0.923076923077	3UTR
hsa-miR-24-3p	NM_005656	TMPRSS2	0.923076923077	3UTR
hsa-miR-30a-3p	NM_005656	TMPRSS2	0.923076923077	3UTR
hsa-miR-92a-1-5p	NM_005656	TMPRSS2	0.923076923077	3UTR
hsa-miR-93-5p	NM_005656	TMPRSS2	0.923076923077	3UTR
hsa-miR-95-3p	NM_005656	TMPRSS2	0.923076923077	3UTR
hsa-miR-103a-2-5p	NM_005656	TMPRSS2	0.923076923077	3UTR
hsa-miR-106a-5p	NM_005656	TMPRSS2	0.923076923077	3UTR
hsa-miR-106a-5p	NM_005656	TMPRSS2	0.923076923077	3UTR
hsa-miR-106a-5p	NM_005656	TMPRSS2	0.923076923077	3UTR
hsa-miR-16-2-3p	NM_005656	TMPRSS2	0.923076923077	3UTR
hsa-miR-196a-5p	NM_005656	TMPRSS2	0.923076923077	3UTR
hsa-miR-197-5p	NM_005656	TMPRSS2	0.923076923077	3UTR
hsa-miR-197-3p	NM_005656	TMPRSS2	0.923076923077	3UTR
hsa-miR-198	NM_005656	TMPRSS2	0.923076923077	3UTR
hsa-miR-199a-3p	NM_005656	TMPRSS2	0.923076923077	3UTR
hsa-miR-129-1-3p	NM_005656	TMPRSS2	0.923076923077	3UTR
hsa-miR-30d-3p	NM_005656	TMPRSS2	0.923076923077	3UTR
hsa-miR-7-5p	NM_005656	TMPRSS2	0.923076923077	3UTR
hsa-miR-182-5p	NM_005656	TMPRSS2	0.923076923077	3UTR
hsa-miR-187-5p	NM_005656	TMPRSS2	0.923076923077	3UTR
hsa-miR-199b-3p	NM_005656	TMPRSS2	0.923076923077	3UTR
hsa-miR-216a-3p	NM_005656	TMPRSS2	0.923076923077	3UTR
hsa-miR-218-1-3p	NM_005656	TMPRSS2	0.923076923077	3UTR
hsa-miR-224-5p	NM_005656	TMPRSS2	0.923076923077	3UTR
hsa-miR-224-3p	NM_005656	TMPRSS2	0.923076923077	3UTR
hsa-miR-224-3p	NM_005656	TMPRSS2	0.923076923077	3UTR

hsa-miR-23b-5p	NM_005656	TMPRSS2	0.923076923077	3UTR
hsa-miR-27b-3p	NM_005656	TMPRSS2	0.923076923077	3UTR
hsa-miR-128-1-5p	NM_005656	TMPRSS2	0.923076923077	3UTR
hsa-miR-128-1-5p	NM_005656	TMPRSS2	0.923076923077	3UTR
hsa-miR-132-3p	NM_005656	TMPRSS2	0.923076923077	3UTR
hsa-miR-135a-2-3p	NM_005656	TMPRSS2	0.923076923077	3UTR
hsa-miR-138-2-3p	NM_005656	TMPRSS2	0.923076923077	3UTR
hsa-miR-140-5p	NM_005656	TMPRSS2	0.923076923077	3UTR
hsa-miR-140-3p	NM_005656	TMPRSS2	0.923076923077	3UTR
hsa-miR-145-3p	NM_005656	TMPRSS2	0.923076923077	3UTR
hsa-miR-152-3p	NM_005656	TMPRSS2	0.923076923077	3UTR
hsa-miR-153-5p	NM_005656	TMPRSS2	0.923076923077	3UTR
hsa-miR-9-5p	NM_005656	TMPRSS2	0.923076923077	3UTR
hsa-miR-9-5p	NM_005656	TMPRSS2	0.923076923077	3UTR
hsa-miR-193a-5p	NM_005656	TMPRSS2	0.923076923077	3UTR
hsa-miR-195-5p	NM_005656	TMPRSS2	0.923076923077	3UTR
hsa-miR-29c-5p	NM_005656	TMPRSS2	0.923076923077	3UTR
hsa-miR-29c-5p	NM_005656	TMPRSS2	0.923076923077	3UTR
hsa-miR-34c-3p	NM_005656	TMPRSS2	0.923076923077	3UTR
hsa-miR-296-3p	NM_005656	TMPRSS2	0.923076923077	3UTR
hsa-miR-296-3p	NM_005656	TMPRSS2	0.923076923077	3UTR
hsa-miR-361-5p	NM_005656	TMPRSS2	0.923076923077	3UTR
hsa-miR-365b-5p	NM_005656	TMPRSS2	0.923076923077	3UTR
hsa-miR-370-3p	NM_005656	TMPRSS2	0.923076923077	3UTR
hsa-miR-331-5p	NM_005656	TMPRSS2	0.923076923077	3UTR
hsa-miR-324-5p	NM_005656	TMPRSS2	0.923076923077	3UTR
hsa-miR-339-5p	NM_005656	TMPRSS2	0.923076923077	3UTR
hsa-miR-339-3p	NM_005656	TMPRSS2	0.923076923077	3UTR
hsa-miR-423-5p	NM_005656	TMPRSS2	0.923076923077	3UTR
hsa-miR-20b-5p	NM_005656	TMPRSS2	0.923076923077	3UTR
hsa-miR-20b-5p	NM_005656	TMPRSS2	0.923076923077	3UTR
hsa-miR-20b-5p	NM_005656	TMPRSS2	0.923076923077	3UTR
hsa-miR-448	NM_005656	TMPRSS2	0.923076923077	3UTR
hsa-miR-449a	NM_005656	TMPRSS2	0.923076923077	3UTR
hsa-miR-449a	NM_005656	TMPRSS2	0.923076923077	3UTR
hsa-miR-329-5p	NM_005656	TMPRSS2	0.923076923077	3UTR
hsa-miR-329-5p	NM_005656	TMPRSS2	0.923076923077	3UTR
hsa-miR-483-3p	NM_005656	TMPRSS2	0.923076923077	3UTR
hsa-miR-485-3p	NM_005656	TMPRSS2	0.923076923077	3UTR
hsa-miR-486-3p	NM_005656	TMPRSS2	0.923076923077	3UTR
hsa-miR-491-5p	NM_005656	TMPRSS2	0.923076923077	3UTR
hsa-miR-491-3p	NM_005656	TMPRSS2	0.923076923077	3UTR
hsa-miR-146b-3p	NM_005656	TMPRSS2	0.923076923077	3UTR
hsa-miR-492	NM_005656	TMPRSS2	0.923076923077	3UTR
hsa-miR-432-5p	NM_005656	TMPRSS2	0.923076923077	3UTR
hsa-miR-512-5p	NM_005656	TMPRSS2	0.923076923077	3UTR
hsa-miR-512-5p	NM_005656	TMPRSS2	0.923076923077	3UTR

hsa-miR-498-5p	NM_005656	TMPRSS2	0.923076923077	3UTR
hsa-miR-520e-5p	NM_005656	TMPRSS2	0.923076923077	3UTR
hsa-miR-515-5p	NM_005656	TMPRSS2	0.923076923077	3UTR
hsa-miR-520f-3p	NM_005656	TMPRSS2	0.923076923077	3UTR
hsa-miR-519c-5p	NM_005656	TMPRSS2	0.923076923077	3UTR
hsa-miR-520a-5p	NM_005656	TMPRSS2	0.923076923077	3UTR
hsa-miR-526b-5p	NM_005656	TMPRSS2	0.923076923077	3UTR
hsa-miR-519b-5p	NM_005656	TMPRSS2	0.923076923077	3UTR
hsa-miR-523-5p	NM_005656	TMPRSS2	0.923076923077	3UTR
hsa-miR-523-3p	NM_005656	TMPRSS2	0.923076923077	3UTR
hsa-miR-518f-5p	NM_005656	TMPRSS2	0.923076923077	3UTR
hsa-miR-524-3p	NM_005656	TMPRSS2	0.923076923077	3UTR
hsa-miR-520d-5p	NM_005656	TMPRSS2	0.923076923077	3UTR
hsa-miR-516b-3p	NM_005656	TMPRSS2	0.923076923077	3UTR
hsa-miR-516b-3p	NM_005656	TMPRSS2	0.923076923077	3UTR
hsa-miR-518e-5p	NM_005656	TMPRSS2	0.923076923077	3UTR
hsa-miR-520h	NM_005656	TMPRSS2	0.923076923077	3UTR
hsa-miR-522-5p	NM_005656	TMPRSS2	0.923076923077	3UTR
hsa-miR-519a-5p	NM_005656	TMPRSS2	0.923076923077	3UTR
hsa-miR-516a-3p	NM_005656	TMPRSS2	0.923076923077	3UTR
hsa-miR-516a-3p	NM_005656	TMPRSS2	0.923076923077	3UTR
hsa-miR-500a-3p	NM_005656	TMPRSS2	0.923076923077	3UTR
hsa-miR-501-5p	NM_005656	TMPRSS2	0.923076923077	3UTR
hsa-miR-504-5p	NM_005656	TMPRSS2	0.923076923077	3UTR
hsa-miR-513a-5p	NM_005656	TMPRSS2	0.923076923077	3UTR
hsa-miR-507	NM_005656	TMPRSS2	0.923076923077	3UTR
hsa-miR-509-3p	NM_005656	TMPRSS2	0.923076923077	3UTR
hsa-miR-532-3p	NM_005656	TMPRSS2	0.923076923077	3UTR
hsa-miR-376a-2-5p	NM_005656	TMPRSS2	0.923076923077	3UTR
hsa-miR-562	NM_005656	TMPRSS2	0.923076923077	3UTR
hsa-miR-584-5p	NM_005656	TMPRSS2	0.923076923077	3UTR
hsa-miR-548a-3p	NM_005656	TMPRSS2	0.923076923077	3UTR
hsa-miR-548b-3p	NM_005656	TMPRSS2	0.923076923077	3UTR
hsa-miR-589-5p	NM_005656	TMPRSS2	0.923076923077	3UTR
hsa-miR-591	NM_005656	TMPRSS2	0.923076923077	3UTR
hsa-miR-601	NM_005656	TMPRSS2	0.923076923077	3UTR
hsa-miR-604	NM_005656	TMPRSS2	0.923076923077	3UTR
hsa-miR-609	NM_005656	TMPRSS2	0.923076923077	3UTR
hsa-miR-616-5p	NM_005656	TMPRSS2	0.923076923077	3UTR
hsa-miR-616-3p	NM_005656	TMPRSS2	0.923076923077	3UTR
hsa-miR-617	NM_005656	TMPRSS2	0.923076923077	3UTR
hsa-miR-618	NM_005656	TMPRSS2	0.923076923077	3UTR
hsa-miR-619-5p	NM_005656	TMPRSS2	0.923076923077	3UTR
hsa-miR-619-5p	NM_005656	TMPRSS2	0.923076923077	3UTR
hsa-miR-619-3p	NM_005656	TMPRSS2	0.923076923077	3UTR
hsa-miR-625-5p	NM_005656	TMPRSS2	0.923076923077	3UTR
hsa-miR-627-5p	NM_005656	TMPRSS2	0.923076923077	3UTR

hsa-miR-632	NM_005656	TMPRSS2	0.923076923077	3UTR
hsa-miR-635	NM_005656	TMPRSS2	0.923076923077	3UTR
hsa-miR-643	NM_005656	TMPRSS2	0.923076923077	3UTR
hsa-miR-646	NM_005656	TMPRSS2	0.923076923077	3UTR
hsa-miR-646	NM_005656	TMPRSS2	0.923076923077	3UTR
hsa-miR-648	NM_005656	TMPRSS2	0.923076923077	3UTR
hsa-miR-652-3p	NM_005656	TMPRSS2	0.923076923077	3UTR
hsa-miR-449b-3p	NM_005656	TMPRSS2	0.923076923077	3UTR
hsa-miR-449b-3p	NM_005656	TMPRSS2	0.923076923077	3UTR
hsa-miR-550a-3-5p	NM_005656	TMPRSS2	0.923076923077	3UTR
hsa-miR-151b	NM_005656	TMPRSS2	0.923076923077	3UTR
hsa-miR-1323	NM_005656	TMPRSS2	0.923076923077	3UTR
hsa-miR-1271-5p	NM_005656	TMPRSS2	0.923076923077	3UTR
hsa-miR-1271-3p	NM_005656	TMPRSS2	0.923076923077	3UTR
hsa-miR-1301-3p	NM_005656	TMPRSS2	0.923076923077	3UTR
hsa-miR-769-3p	NM_005656	TMPRSS2	0.923076923077	3UTR
hsa-miR-378d	NM_005656	TMPRSS2	0.923076923077	3UTR
hsa-miR-675-3p	NM_005656	TMPRSS2	0.923076923077	3UTR
hsa-miR-874-3p	NM_005656	TMPRSS2	0.923076923077	3UTR
hsa-miR-890	NM_005656	TMPRSS2	0.923076923077	3UTR
hsa-miR-892b	NM_005656	TMPRSS2	0.923076923077	3UTR
hsa-miR-875-5p	NM_005656	TMPRSS2	0.923076923077	3UTR
hsa-miR-885-5p	NM_005656	TMPRSS2	0.923076923077	3UTR
hsa-miR-877-5p	NM_005656	TMPRSS2	0.923076923077	3UTR
hsa-miR-887-5p	NM_005656	TMPRSS2	0.923076923077	3UTR
hsa-miR-665	NM_005656	TMPRSS2	0.923076923077	3UTR
hsa-miR-216b-5p	NM_005656	TMPRSS2	0.923076923077	3UTR
hsa-miR-936	NM_005656	TMPRSS2	0.923076923077	3UTR
hsa-miR-1181	NM_005656	TMPRSS2	0.923076923077	3UTR
hsa-miR-1182	NM_005656	TMPRSS2	0.923076923077	3UTR
hsa-miR-1184	NM_005656	TMPRSS2	0.923076923077	3UTR
hsa-miR-1236-5p	NM_005656	TMPRSS2	0.923076923077	3UTR
hsa-miR-1236-3p	NM_005656	TMPRSS2	0.923076923077	3UTR
hsa-miR-1202	NM_005656	TMPRSS2	0.923076923077	3UTR
hsa-miR-1289	NM_005656	TMPRSS2	0.923076923077	3UTR
hsa-miR-1289	NM_005656	TMPRSS2	0.923076923077	3UTR
hsa-miR-1291	NM_005656	TMPRSS2	0.923076923077	3UTR
hsa-miR-548k	NM_005656	TMPRSS2	0.923076923077	3UTR
hsa-miR-1243	NM_005656	TMPRSS2	0.923076923077	3UTR
hsa-miR-1249-5p	NM_005656	TMPRSS2	0.923076923077	3UTR
hsa-miR-1250-5p	NM_005656	TMPRSS2	0.923076923077	3UTR
hsa-miR-1251-5p	NM_005656	TMPRSS2	0.923076923077	3UTR
hsa-miR-1258	NM_005656	TMPRSS2	0.923076923077	3UTR
hsa-miR-1265	NM_005656	TMPRSS2	0.923076923077	3UTR
hsa-miR-1270	NM_005656	TMPRSS2	0.923076923077	3UTR
hsa-miR-1275	NM_005656	TMPRSS2	0.923076923077	3UTR
hsa-miR-1276	NM_005656	TMPRSS2	0.923076923077	3UTR

hsa-miR-1321	NM_005656	TMPRSS2	0.923076923077	3UTR
hsa-miR-1321	NM_005656	TMPRSS2	0.923076923077	3UTR
hsa-miR-1324	NM_005656	TMPRSS2	0.923076923077	3UTR
hsa-miR-320d	NM_005656	TMPRSS2	0.923076923077	3UTR
hsa-miR-1908-5p	NM_005656	TMPRSS2	0.923076923077	3UTR
hsa-miR-1909-3p	NM_005656	TMPRSS2	0.923076923077	3UTR
hsa-miR-1911-3p	NM_005656	TMPRSS2	0.923076923077	3UTR
hsa-miR-1912-5p	NM_005656	TMPRSS2	0.923076923077	3UTR
hsa-miR-2114-5p	NM_005656	TMPRSS2	0.923076923077	3UTR
hsa-miR-2117	NM_005656	TMPRSS2	0.923076923077	3UTR
hsa-miR-2276-3p	NM_005656	TMPRSS2	0.923076923077	3UTR
hsa-miR-2277-3p	NM_005656	TMPRSS2	0.923076923077	3UTR
hsa-miR-2682-5p	NM_005656	TMPRSS2	0.923076923077	3UTR
hsa-miR-711	NM_005656	TMPRSS2	0.923076923077	3UTR
hsa-miR-548s	NM_005656	TMPRSS2	0.923076923077	3UTR
hsa-miR-3132	NM_005656	TMPRSS2	0.923076923077	3UTR
hsa-miR-378b	NM_005656	TMPRSS2	0.923076923077	3UTR
hsa-miR-3138	NM_005656	TMPRSS2	0.923076923077	3UTR
hsa-miR-3141	NM_005656	TMPRSS2	0.923076923077	3UTR
hsa-miR-1273c	NM_005656	TMPRSS2	0.923076923077	3UTR
hsa-miR-3150a-3p	NM_005656	TMPRSS2	0.923076923077	3UTR
hsa-miR-3152-3p	NM_005656	TMPRSS2	0.923076923077	3UTR
hsa-miR-3153	NM_005656	TMPRSS2	0.923076923077	3UTR
hsa-miR-3154	NM_005656	TMPRSS2	0.923076923077	3UTR
hsa-miR-3158-5p	NM_005656	TMPRSS2	0.923076923077	3UTR
hsa-miR-3162-3p	NM_005656	TMPRSS2	0.923076923077	3UTR
hsa-miR-1260b	NM_005656	TMPRSS2	0.923076923077	3UTR
hsa-miR-3168	NM_005656	TMPRSS2	0.923076923077	3UTR
hsa-miR-3173-5p	NM_005656	TMPRSS2	0.923076923077	3UTR
hsa-miR-3173-5p	NM_005656	TMPRSS2	0.923076923077	3UTR
hsa-miR-3173-3p	NM_005656	TMPRSS2	0.923076923077	3UTR
hsa-miR-3179	NM_005656	TMPRSS2	0.923076923077	3UTR
hsa-miR-3180-5p	NM_005656	TMPRSS2	0.923076923077	3UTR
hsa-miR-3184-5p	NM_005656	TMPRSS2	0.923076923077	3UTR
hsa-miR-3184-5p	NM_005656	TMPRSS2	0.923076923077	3UTR
hsa-miR-3185	NM_005656	TMPRSS2	0.923076923077	3UTR
hsa-miR-3189-5p	NM_005656	TMPRSS2	0.923076923077	3UTR
hsa-miR-3189-3p	NM_005656	TMPRSS2	0.923076923077	3UTR
hsa-miR-3190-3p	NM_005656	TMPRSS2	0.923076923077	3UTR
hsa-miR-3194-5p	NM_005656	TMPRSS2	0.923076923077	3UTR
hsa-miR-3197	NM_005656	TMPRSS2	0.923076923077	3UTR
hsa-miR-3198	NM_005656	TMPRSS2	0.923076923077	3UTR
hsa-miR-3202	NM_005656	TMPRSS2	0.923076923077	3UTR
hsa-miR-4301	NM_005656	TMPRSS2	0.923076923077	3UTR
hsa-miR-4301	NM_005656	TMPRSS2	0.923076923077	3UTR
hsa-miR-4299	NM_005656	TMPRSS2	0.923076923077	3UTR
hsa-miR-4300	NM_005656	TMPRSS2	0.923076923077	3UTR

hsa-miR-4304	NM_005656	TMPRSS2	0.923076923077	3UTR
hsa-miR-4306	NM_005656	TMPRSS2	0.923076923077	3UTR
hsa-miR-4316	NM_005656	TMPRSS2	0.923076923077	3UTR
hsa-miR-4318	NM_005656	TMPRSS2	0.923076923077	3UTR
hsa-miR-4261	NM_005656	TMPRSS2	0.923076923077	3UTR
hsa-miR-4266	NM_005656	TMPRSS2	0.923076923077	3UTR
hsa-miR-4270	NM_005656	TMPRSS2	0.923076923077	3UTR
hsa-miR-4278	NM_005656	TMPRSS2	0.923076923077	3UTR
hsa-miR-4280	NM_005656	TMPRSS2	0.923076923077	3UTR
hsa-miR-4283	NM_005656	TMPRSS2	0.923076923077	3UTR
hsa-miR-4283	NM_005656	TMPRSS2	0.923076923077	3UTR
hsa-miR-500b-5p	NM_005656	TMPRSS2	0.923076923077	3UTR
hsa-miR-3612	NM_005656	TMPRSS2	0.923076923077	3UTR
hsa-miR-3617-3p	NM_005656	TMPRSS2	0.923076923077	3UTR
hsa-miR-3617-3p	NM_005656	TMPRSS2	0.923076923077	3UTR
hsa-miR-3619-3p	NM_005656	TMPRSS2	0.923076923077	3UTR
hsa-miR-3649	NM_005656	TMPRSS2	0.923076923077	3UTR
hsa-miR-3661	NM_005656	TMPRSS2	0.923076923077	3UTR
hsa-miR-3662	NM_005656	TMPRSS2	0.923076923077	3UTR
hsa-miR-3665	NM_005656	TMPRSS2	0.923076923077	3UTR
hsa-miR-3667-3p	NM_005656	TMPRSS2	0.923076923077	3UTR
hsa-miR-3677-3p	NM_005656	TMPRSS2	0.923076923077	3UTR
hsa-miR-3678-3p	NM_005656	TMPRSS2	0.923076923077	3UTR
hsa-miR-3680-3p	NM_005656	TMPRSS2	0.923076923077	3UTR
hsa-miR-3680-3p	NM_005656	TMPRSS2	0.923076923077	3UTR
hsa-miR-3682-3p	NM_005656	TMPRSS2	0.923076923077	3UTR
hsa-miR-3689a-5p	NM_005656	TMPRSS2	0.923076923077	3UTR
hsa-miR-3692-5p	NM_005656	TMPRSS2	0.923076923077	3UTR
hsa-miR-3689b-5p	NM_005656	TMPRSS2	0.923076923077	3UTR
hsa-miR-3689b-3p	NM_005656	TMPRSS2	0.923076923077	3UTR
hsa-miR-3913-3p	NM_005656	TMPRSS2	0.923076923077	3UTR
hsa-miR-3919	NM_005656	TMPRSS2	0.923076923077	3UTR
hsa-miR-3923	NM_005656	TMPRSS2	0.923076923077	3UTR
hsa-miR-3934-3p	NM_005656	TMPRSS2	0.923076923077	3UTR
hsa-miR-3939	NM_005656	TMPRSS2	0.923076923077	3UTR
hsa-miR-3940-5p	NM_005656	TMPRSS2	0.923076923077	3UTR
hsa-miR-3945	NM_005656	TMPRSS2	0.923076923077	3UTR
hsa-miR-550b-2-5p	NM_005656	TMPRSS2	0.923076923077	3UTR
hsa-miR-378g	NM_005656	TMPRSS2	0.923076923077	3UTR
hsa-miR-4429	NM_005656	TMPRSS2	0.923076923077	3UTR
hsa-miR-4433a-5p	NM_005656	TMPRSS2	0.923076923077	3UTR
hsa-miR-4442	NM_005656	TMPRSS2	0.923076923077	3UTR
hsa-miR-4448	NM_005656	TMPRSS2	0.923076923077	3UTR
hsa-miR-4451	NM_005656	TMPRSS2	0.923076923077	3UTR
hsa-miR-4458	NM_005656	TMPRSS2	0.923076923077	3UTR
hsa-miR-4465	NM_005656	TMPRSS2	0.923076923077	3UTR
hsa-miR-4466	NM_005656	TMPRSS2	0.923076923077	3UTR

hsa-miR-4470	NM_005656	TMPRSS2	0.923076923077	3UTR
hsa-miR-4471	NM_005656	TMPRSS2	0.923076923077	3UTR
hsa-miR-4474-3p	NM_005656	TMPRSS2	0.923076923077	3UTR
hsa-miR-3689c	NM_005656	TMPRSS2	0.923076923077	3UTR
hsa-miR-3689e	NM_005656	TMPRSS2	0.923076923077	3UTR
hsa-miR-4480	NM_005656	TMPRSS2	0.923076923077	3UTR
hsa-miR-4482-3p	NM_005656	TMPRSS2	0.923076923077	3UTR
hsa-miR-4487	NM_005656	TMPRSS2	0.923076923077	3UTR
hsa-miR-4494	NM_005656	TMPRSS2	0.923076923077	3UTR
hsa-miR-4515	NM_005656	TMPRSS2	0.923076923077	3UTR
hsa-miR-4519	NM_005656	TMPRSS2	0.923076923077	3UTR
hsa-miR-4530	NM_005656	TMPRSS2	0.923076923077	3UTR
hsa-miR-4533	NM_005656	TMPRSS2	0.923076923077	3UTR
hsa-miR-4534	NM_005656	TMPRSS2	0.923076923077	3UTR
hsa-miR-378i	NM_005656	TMPRSS2	0.923076923077	3UTR
hsa-miR-4540	NM_005656	TMPRSS2	0.923076923077	3UTR
hsa-miR-3972	NM_005656	TMPRSS2	0.923076923077	3UTR
hsa-miR-3976	NM_005656	TMPRSS2	0.923076923077	3UTR
hsa-miR-3978	NM_005656	TMPRSS2	0.923076923077	3UTR
hsa-miR-4635	NM_005656	TMPRSS2	0.923076923077	3UTR
hsa-miR-4640-3p	NM_005656	TMPRSS2	0.923076923077	3UTR
hsa-miR-4644	NM_005656	TMPRSS2	0.923076923077	3UTR
hsa-miR-4647	NM_005656	TMPRSS2	0.923076923077	3UTR
hsa-miR-4649-3p	NM_005656	TMPRSS2	0.923076923077	3UTR
hsa-miR-4653-3p	NM_005656	TMPRSS2	0.923076923077	3UTR
hsa-miR-4654	NM_005656	TMPRSS2	0.923076923077	3UTR
hsa-miR-4655-5p	NM_005656	TMPRSS2	0.923076923077	3UTR
hsa-miR-4656	NM_005656	TMPRSS2	0.923076923077	3UTR
hsa-miR-4659a-5p	NM_005656	TMPRSS2	0.923076923077	3UTR
hsa-miR-4663	NM_005656	TMPRSS2	0.923076923077	3UTR
hsa-miR-4667-5p	NM_005656	TMPRSS2	0.923076923077	3UTR
hsa-miR-4667-5p	NM_005656	TMPRSS2	0.923076923077	3UTR
hsa-miR-4682	NM_005656	TMPRSS2	0.923076923077	3UTR
hsa-miR-4687-5p	NM_005656	TMPRSS2	0.923076923077	3UTR
hsa-miR-4687-3p	NM_005656	TMPRSS2	0.923076923077	3UTR
hsa-miR-4689	NM_005656	TMPRSS2	0.923076923077	3UTR
hsa-miR-4690-5p	NM_005656	TMPRSS2	0.923076923077	3UTR
hsa-miR-4695-3p	NM_005656	TMPRSS2	0.923076923077	3UTR
hsa-miR-4704-5p	NM_005656	TMPRSS2	0.923076923077	3UTR
hsa-miR-4704-5p	NM_005656	TMPRSS2	0.923076923077	3UTR
hsa-miR-4706	NM_005656	TMPRSS2	0.923076923077	3UTR
hsa-miR-4708-3p	NM_005656	TMPRSS2	0.923076923077	3UTR
hsa-miR-4709-3p	NM_005656	TMPRSS2	0.923076923077	3UTR
hsa-miR-4710	NM_005656	TMPRSS2	0.923076923077	3UTR
hsa-miR-4713-5p	NM_005656	TMPRSS2	0.923076923077	3UTR
hsa-miR-4713-3p	NM_005656	TMPRSS2	0.923076923077	3UTR
hsa-miR-4715-3p	NM_005656	TMPRSS2	0.923076923077	3UTR

hsa-miR-4725-5p	NM_005656	TMPRSS2	0.923076923077	3UTR
hsa-miR-4731-3p	NM_005656	TMPRSS2	0.923076923077	3UTR
hsa-miR-4732-5p	NM_005656	TMPRSS2	0.923076923077	3UTR
hsa-miR-4738-3p	NM_005656	TMPRSS2	0.923076923077	3UTR
hsa-miR-4742-5p	NM_005656	TMPRSS2	0.923076923077	3UTR
hsa-miR-4743-5p	NM_005656	TMPRSS2	0.923076923077	3UTR
hsa-miR-4744	NM_005656	TMPRSS2	0.923076923077	3UTR
hsa-miR-4748	NM_005656	TMPRSS2	0.923076923077	3UTR
hsa-miR-4749-3p	NM_005656	TMPRSS2	0.923076923077	3UTR
hsa-miR-4755-5p	NM_005656	TMPRSS2	0.923076923077	3UTR
hsa-miR-4756-3p	NM_005656	TMPRSS2	0.923076923077	3UTR
hsa-miR-4760-3p	NM_005656	TMPRSS2	0.923076923077	3UTR
hsa-miR-4764-5p	NM_005656	TMPRSS2	0.923076923077	3UTR
hsa-miR-4775	NM_005656	TMPRSS2	0.923076923077	3UTR
hsa-miR-4776-5p	NM_005656	TMPRSS2	0.923076923077	3UTR
hsa-miR-4436b-3p	NM_005656	TMPRSS2	0.923076923077	3UTR
hsa-miR-4785	NM_005656	TMPRSS2	0.923076923077	3UTR
hsa-miR-2467-3p	NM_005656	TMPRSS2	0.923076923077	3UTR
hsa-miR-4786-3p	NM_005656	TMPRSS2	0.923076923077	3UTR
hsa-miR-4790-5p	NM_005656	TMPRSS2	0.923076923077	3UTR
hsa-miR-4793-5p	NM_005656	TMPRSS2	0.923076923077	3UTR
hsa-miR-4796-3p	NM_005656	TMPRSS2	0.923076923077	3UTR
hsa-miR-4797-3p	NM_005656	TMPRSS2	0.923076923077	3UTR
hsa-miR-4799-3p	NM_005656	TMPRSS2	0.923076923077	3UTR
hsa-miR-4799-3p	NM_005656	TMPRSS2	0.923076923077	3UTR
hsa-miR-4800-5p	NM_005656	TMPRSS2	0.923076923077	3UTR
hsa-miR-5004-5p	NM_005656	TMPRSS2	0.923076923077	3UTR
hsa-miR-5004-3p	NM_005656	TMPRSS2	0.923076923077	3UTR
hsa-miR-548ao-3p	NM_005656	TMPRSS2	0.923076923077	3UTR
hsa-miR-5006-5p	NM_005656	TMPRSS2	0.923076923077	3UTR
hsa-miR-5006-5p	NM_005656	TMPRSS2	0.923076923077	3UTR
hsa-miR-5008-3p	NM_005656	TMPRSS2	0.923076923077	3UTR
hsa-miR-5010-5p	NM_005656	TMPRSS2	0.923076923077	3UTR
hsa-miR-5047	NM_005656	TMPRSS2	0.923076923077	3UTR
hsa-miR-5088-5p	NM_005656	TMPRSS2	0.923076923077	3UTR
hsa-miR-5088-3p	NM_005656	TMPRSS2	0.923076923077	3UTR
hsa-miR-5090	NM_005656	TMPRSS2	0.923076923077	3UTR
hsa-miR-5092	NM_005656	TMPRSS2	0.923076923077	3UTR
hsa-miR-5187-3p	NM_005656	TMPRSS2	0.923076923077	3UTR
hsa-miR-5187-3p	NM_005656	TMPRSS2	0.923076923077	3UTR
hsa-miR-5190	NM_005656	TMPRSS2	0.923076923077	3UTR
hsa-miR-5192	NM_005656	TMPRSS2	0.923076923077	3UTR
hsa-miR-5192	NM_005656	TMPRSS2	0.923076923077	3UTR
hsa-miR-5193	NM_005656	TMPRSS2	0.923076923077	3UTR
hsa-miR-5193	NM_005656	TMPRSS2	0.923076923077	3UTR
hsa-miR-5193	NM_005656	TMPRSS2	0.923076923077	3UTR
hsa-miR-5196-5p	NM_005656	TMPRSS2	0.923076923077	3UTR

hsa-miR-5197-3p	NM_005656	TMPRSS2	0.923076923077	3UTR
hsa-miR-5571-3p	NM_005656	TMPRSS2	0.923076923077	3UTR
hsa-miR-5580-3p	NM_005656	TMPRSS2	0.923076923077	3UTR
hsa-miR-5584-3p	NM_005656	TMPRSS2	0.923076923077	3UTR
hsa-miR-5587-5p	NM_005656	TMPRSS2	0.923076923077	3UTR
hsa-miR-1295b-5p	NM_005656	TMPRSS2	0.923076923077	3UTR
hsa-miR-1295b-3p	NM_005656	TMPRSS2	0.923076923077	3UTR
hsa-miR-5589-5p	NM_005656	TMPRSS2	0.923076923077	3UTR
hsa-miR-5591-5p	NM_005656	TMPRSS2	0.923076923077	3UTR
hsa-miR-5591-5p	NM_005656	TMPRSS2	0.923076923077	3UTR
hsa-miR-5591-3p	NM_005656	TMPRSS2	0.923076923077	3UTR
hsa-miR-5682	NM_005656	TMPRSS2	0.923076923077	3UTR
hsa-miR-5690	NM_005656	TMPRSS2	0.923076923077	3UTR
hsa-miR-5693	NM_005656	TMPRSS2	0.923076923077	3UTR
hsa-miR-5704	NM_005656	TMPRSS2	0.923076923077	3UTR
hsa-miR-6068	NM_005656	TMPRSS2	0.923076923077	3UTR
hsa-miR-6077	NM_005656	TMPRSS2	0.923076923077	3UTR
hsa-miR-6085	NM_005656	TMPRSS2	0.923076923077	3UTR
hsa-miR-6127	NM_005656	TMPRSS2	0.923076923077	3UTR
hsa-miR-6127	NM_005656	TMPRSS2	0.923076923077	3UTR
hsa-miR-378j	NM_005656	TMPRSS2	0.923076923077	3UTR
hsa-miR-6134	NM_005656	TMPRSS2	0.923076923077	3UTR
hsa-miR-548ay-3p	NM_005656	TMPRSS2	0.923076923077	3UTR
hsa-miR-6500-5p	NM_005656	TMPRSS2	0.923076923077	3UTR
hsa-miR-6503-3p	NM_005656	TMPRSS2	0.923076923077	3UTR
hsa-miR-6503-3p	NM_005656	TMPRSS2	0.923076923077	3UTR
hsa-miR-6509-5p	NM_005656	TMPRSS2	0.923076923077	3UTR
hsa-miR-6509-3p	NM_005656	TMPRSS2	0.923076923077	3UTR
hsa-miR-6511a-5p	NM_005656	TMPRSS2	0.923076923077	3UTR
hsa-miR-6515-5p	NM_005656	TMPRSS2	0.923076923077	3UTR
hsa-miR-6716-3p	NM_005656	TMPRSS2	0.923076923077	3UTR
hsa-miR-6511b-5p	NM_005656	TMPRSS2	0.923076923077	3UTR
hsa-miR-6720-3p	NM_005656	TMPRSS2	0.923076923077	3UTR
hsa-miR-6728-5p	NM_005656	TMPRSS2	0.923076923077	3UTR
hsa-miR-6729-5p	NM_005656	TMPRSS2	0.923076923077	3UTR
hsa-miR-6731-5p	NM_005656	TMPRSS2	0.923076923077	3UTR
hsa-miR-6734-5p	NM_005656	TMPRSS2	0.923076923077	3UTR
hsa-miR-6734-5p	NM_005656	TMPRSS2	0.923076923077	3UTR
hsa-miR-6735-5p	NM_005656	TMPRSS2	0.923076923077	3UTR
hsa-miR-6735-3p	NM_005656	TMPRSS2	0.923076923077	3UTR
hsa-miR-6736-5p	NM_005656	TMPRSS2	0.923076923077	3UTR
hsa-miR-6736-3p	NM_005656	TMPRSS2	0.923076923077	3UTR
hsa-miR-6738-5p	NM_005656	TMPRSS2	0.923076923077	3UTR
hsa-miR-6740-5p	NM_005656	TMPRSS2	0.923076923077	3UTR
hsa-miR-6742-5p	NM_005656	TMPRSS2	0.923076923077	3UTR
hsa-miR-6747-5p	NM_005656	TMPRSS2	0.923076923077	3UTR
hsa-miR-6748-5p	NM_005656	TMPRSS2	0.923076923077	3UTR

hsa-miR-6751-3p	NM_005656	TMPRSS2	0.923076923077	3UTR
hsa-miR-6753-5p	NM_005656	TMPRSS2	0.923076923077	3UTR
hsa-miR-6754-5p	NM_005656	TMPRSS2	0.923076923077	3UTR
hsa-miR-6758-5p	NM_005656	TMPRSS2	0.923076923077	3UTR
hsa-miR-6763-5p	NM_005656	TMPRSS2	0.923076923077	3UTR
hsa-miR-6763-5p	NM_005656	TMPRSS2	0.923076923077	3UTR
hsa-miR-6765-5p	NM_005656	TMPRSS2	0.923076923077	3UTR
hsa-miR-6765-3p	NM_005656	TMPRSS2	0.923076923077	3UTR
hsa-miR-6766-3p	NM_005656	TMPRSS2	0.923076923077	3UTR
hsa-miR-6767-3p	NM_005656	TMPRSS2	0.923076923077	3UTR
hsa-miR-6768-3p	NM_005656	TMPRSS2	0.923076923077	3UTR
hsa-miR-6769a-5p	NM_005656	TMPRSS2	0.923076923077	3UTR
hsa-miR-6773-3p	NM_005656	TMPRSS2	0.923076923077	3UTR
hsa-miR-6774-5p	NM_005656	TMPRSS2	0.923076923077	3UTR
hsa-miR-6774-3p	NM_005656	TMPRSS2	0.923076923077	3UTR
hsa-miR-6780a-3p	NM_005656	TMPRSS2	0.923076923077	3UTR
hsa-miR-6781-5p	NM_005656	TMPRSS2	0.923076923077	3UTR
hsa-miR-6787-3p	NM_005656	TMPRSS2	0.923076923077	3UTR
hsa-miR-6791-5p	NM_005656	TMPRSS2	0.923076923077	3UTR
hsa-miR-6792-5p	NM_005656	TMPRSS2	0.923076923077	3UTR
hsa-miR-6793-3p	NM_005656	TMPRSS2	0.923076923077	3UTR
hsa-miR-6799-5p	NM_005656	TMPRSS2	0.923076923077	3UTR
hsa-miR-6799-3p	NM_005656	TMPRSS2	0.923076923077	3UTR
hsa-miR-6800-3p	NM_005656	TMPRSS2	0.923076923077	3UTR
hsa-miR-6801-5p	NM_005656	TMPRSS2	0.923076923077	3UTR
hsa-miR-6801-3p	NM_005656	TMPRSS2	0.923076923077	3UTR
hsa-miR-6802-5p	NM_005656	TMPRSS2	0.923076923077	3UTR
hsa-miR-6804-5p	NM_005656	TMPRSS2	0.923076923077	3UTR
hsa-miR-6804-5p	NM_005656	TMPRSS2	0.923076923077	3UTR
hsa-miR-6806-5p	NM_005656	TMPRSS2	0.923076923077	3UTR
hsa-miR-6811-5p	NM_005656	TMPRSS2	0.923076923077	3UTR
hsa-miR-6812-3p	NM_005656	TMPRSS2	0.923076923077	3UTR
hsa-miR-6812-3p	NM_005656	TMPRSS2	0.923076923077	3UTR
hsa-miR-6813-5p	NM_005656	TMPRSS2	0.923076923077	3UTR
hsa-miR-6815-5p	NM_005656	TMPRSS2	0.923076923077	3UTR
hsa-miR-6815-3p	NM_005656	TMPRSS2	0.923076923077	3UTR
hsa-miR-6815-3p	NM_005656	TMPRSS2	0.923076923077	3UTR
hsa-miR-6816-5p	NM_005656	TMPRSS2	0.923076923077	3UTR
hsa-miR-6821-3p	NM_005656	TMPRSS2	0.923076923077	3UTR
hsa-miR-6823-3p	NM_005656	TMPRSS2	0.923076923077	3UTR
hsa-miR-6826-5p	NM_005656	TMPRSS2	0.923076923077	3UTR
hsa-miR-6828-5p	NM_005656	TMPRSS2	0.923076923077	3UTR
hsa-miR-6829-5p	NM_005656	TMPRSS2	0.923076923077	3UTR
hsa-miR-6829-3p	NM_005656	TMPRSS2	0.923076923077	3UTR
hsa-miR-6833-3p	NM_005656	TMPRSS2	0.923076923077	3UTR
hsa-miR-6834-3p	NM_005656	TMPRSS2	0.923076923077	3UTR
hsa-miR-6838-3p	NM_005656	TMPRSS2	0.923076923077	3UTR

hsa-miR-6846-5p	NM_005656	TMPRSS2	0.923076923077	3UTR
hsa-miR-6848-5p	NM_005656	TMPRSS2	0.923076923077	3UTR
hsa-miR-6848-3p	NM_005656	TMPRSS2	0.923076923077	3UTR
hsa-miR-6849-3p	NM_005656	TMPRSS2	0.923076923077	3UTR
hsa-miR-6854-5p	NM_005656	TMPRSS2	0.923076923077	3UTR
hsa-miR-6856-5p	NM_005656	TMPRSS2	0.923076923077	3UTR
hsa-miR-6857-3p	NM_005656	TMPRSS2	0.923076923077	3UTR
hsa-miR-6859-5p	NM_005656	TMPRSS2	0.923076923077	3UTR
hsa-miR-6859-3p	NM_005656	TMPRSS2	0.923076923077	3UTR
hsa-miR-6861-5p	NM_005656	TMPRSS2	0.923076923077	3UTR
hsa-miR-6864-3p	NM_005656	TMPRSS2	0.923076923077	3UTR
hsa-miR-6865-5p	NM_005656	TMPRSS2	0.923076923077	3UTR
hsa-miR-6865-3p	NM_005656	TMPRSS2	0.923076923077	3UTR
hsa-miR-6866-3p	NM_005656	TMPRSS2	0.923076923077	3UTR
hsa-miR-6867-3p	NM_005656	TMPRSS2	0.923076923077	3UTR
hsa-miR-6867-3p	NM_005656	TMPRSS2	0.923076923077	3UTR
hsa-miR-6873-5p	NM_005656	TMPRSS2	0.923076923077	3UTR
hsa-miR-6876-5p	NM_005656	TMPRSS2	0.923076923077	3UTR
hsa-miR-6877-5p	NM_005656	TMPRSS2	0.923076923077	3UTR
hsa-miR-6877-3p	NM_005656	TMPRSS2	0.923076923077	3UTR
hsa-miR-6878-5p	NM_005656	TMPRSS2	0.923076923077	3UTR
hsa-miR-6878-3p	NM_005656	TMPRSS2	0.923076923077	3UTR
hsa-miR-6880-5p	NM_005656	TMPRSS2	0.923076923077	3UTR
hsa-miR-6882-5p	NM_005656	TMPRSS2	0.923076923077	3UTR
hsa-miR-6884-5p	NM_005656	TMPRSS2	0.923076923077	3UTR
hsa-miR-6887-5p	NM_005656	TMPRSS2	0.923076923077	3UTR
hsa-miR-6887-5p	NM_005656	TMPRSS2	0.923076923077	3UTR
hsa-miR-6889-3p	NM_005656	TMPRSS2	0.923076923077	3UTR
hsa-miR-6891-5p	NM_005656	TMPRSS2	0.923076923077	3UTR
hsa-miR-6892-5p	NM_005656	TMPRSS2	0.923076923077	3UTR
hsa-miR-6892-5p	NM_005656	TMPRSS2	0.923076923077	3UTR
hsa-miR-6892-3p	NM_005656	TMPRSS2	0.923076923077	3UTR
hsa-miR-6894-3p	NM_005656	TMPRSS2	0.923076923077	3UTR
hsa-miR-7106-5p	NM_005656	TMPRSS2	0.923076923077	3UTR
hsa-miR-7108-5p	NM_005656	TMPRSS2	0.923076923077	3UTR
hsa-miR-7108-3p	NM_005656	TMPRSS2	0.923076923077	3UTR
hsa-miR-7110-5p	NM_005656	TMPRSS2	0.923076923077	3UTR
hsa-miR-7111-5p	NM_005656	TMPRSS2	0.923076923077	3UTR
hsa-miR-7111-5p	NM_005656	TMPRSS2	0.923076923077	3UTR
hsa-miR-7111-3p	NM_005656	TMPRSS2	0.923076923077	3UTR
hsa-miR-7111-3p	NM_005656	TMPRSS2	0.923076923077	3UTR
hsa-miR-7112-3p	NM_005656	TMPRSS2	0.923076923077	3UTR
hsa-miR-7113-5p	NM_005656	TMPRSS2	0.923076923077	3UTR
hsa-miR-7152-5p	NM_005656	TMPRSS2	0.923076923077	3UTR
hsa-miR-7152-3p	NM_005656	TMPRSS2	0.923076923077	3UTR
hsa-miR-7152-3p	NM_005656	TMPRSS2	0.923076923077	3UTR
hsa-miR-7161-3p	NM_005656	TMPRSS2	0.923076923077	3UTR

hsa-miR-7162-3p	NM_005656	TMPRSS2	0.923076923077	3UTR
hsa-miR-7706	NM_005656	TMPRSS2	0.923076923077	3UTR
hsa-miR-7843-5p	NM_005656	TMPRSS2	0.923076923077	3UTR
hsa-miR-4433b-5p	NM_005656	TMPRSS2	0.923076923077	3UTR
hsa-miR-4433b-3p	NM_005656	TMPRSS2	0.923076923077	3UTR
hsa-miR-4433b-3p	NM_005656	TMPRSS2	0.923076923077	3UTR
hsa-miR-1273h-3p	NM_005656	TMPRSS2	0.923076923077	3UTR
hsa-miR-7851-3p	NM_005656	TMPRSS2	0.923076923077	3UTR
hsa-miR-7851-3p	NM_005656	TMPRSS2	0.923076923077	3UTR
hsa-miR-7854-3p	NM_005656	TMPRSS2	0.923076923077	3UTR
hsa-miR-7974	NM_005656	TMPRSS2	0.923076923077	3UTR
hsa-miR-7978	NM_005656	TMPRSS2	0.923076923077	3UTR
hsa-miR-8072	NM_005656	TMPRSS2	0.923076923077	3UTR
hsa-miR-8077	NM_005656	TMPRSS2	0.923076923077	3UTR
hsa-miR-8080	NM_005656	TMPRSS2	0.923076923077	3UTR
hsa-miR-8085	NM_005656	TMPRSS2	0.923076923077	3UTR
hsa-miR-8086	NM_005656	TMPRSS2	0.923076923077	3UTR
hsa-miR-10392-3p	NM_005656	TMPRSS2	0.923076923077	3UTR
hsa-miR-10397-3p	NM_005656	TMPRSS2	0.923076923077	3UTR
hsa-miR-10401-5p	NM_005656	TMPRSS2	0.923076923077	3UTR
hsa-miR-10522-5p	NM_005656	TMPRSS2	0.923076923077	3UTR
hsa-miR-10524-5p	NM_005656	TMPRSS2	0.923076923077	3UTR
hsa-miR-10526-3p	NM_005656	TMPRSS2	0.923076923077	3UTR
hsa-miR-11181-5p	NM_005656	TMPRSS2	0.923076923077	3UTR
hsa-miR-3059-5p	NM_005656	TMPRSS2	0.923076923077	3UTR
hsa-miR-12126	NM_005656	TMPRSS2	0.923076923077	3UTR
hsa-miR-12127	NM_005656	TMPRSS2	0.923076923077	3UTR
hsa-miR-17-5p	NM_001135099	TMPRSS2	0.923076923077	3UTR
hsa-miR-17-5p	NM_001135099	TMPRSS2	0.923076923077	3UTR
hsa-miR-19b-3p	NM_001135099	TMPRSS2	0.923076923077	3UTR
hsa-miR-20a-5p	NM_001135099	TMPRSS2	0.923076923077	3UTR
hsa-miR-21-3p	NM_001135099	TMPRSS2	0.923076923077	3UTR
hsa-miR-30a-3p	NM_001135099	TMPRSS2	0.923076923077	3UTR
hsa-miR-96-3p	NM_001135099	TMPRSS2	0.923076923077	3UTR
hsa-miR-103a-2-5p	NM_001135099	TMPRSS2	0.923076923077	3UTR
hsa-miR-106a-5p	NM_001135099	TMPRSS2	0.923076923077	3UTR
hsa-miR-106a-5p	NM_001135099	TMPRSS2	0.923076923077	3UTR
hsa-miR-107	NM_001135099	TMPRSS2	0.923076923077	3UTR
hsa-miR-192-3p	NM_001135099	TMPRSS2	0.923076923077	3UTR
hsa-miR-199a-3p	NM_001135099	TMPRSS2	0.923076923077	3UTR
hsa-miR-129-5p	NM_001135099	TMPRSS2	0.923076923077	3UTR
hsa-miR-30d-3p	NM_001135099	TMPRSS2	0.923076923077	3UTR
hsa-miR-7-5p	NM_001135099	TMPRSS2	0.923076923077	3UTR
hsa-miR-181b-5p	NM_001135099	TMPRSS2	0.923076923077	3UTR
hsa-miR-181c-5p	NM_001135099	TMPRSS2	0.923076923077	3UTR
hsa-miR-199b-3p	NM_001135099	TMPRSS2	0.923076923077	3UTR
hsa-miR-216a-3p	NM_001135099	TMPRSS2	0.923076923077	3UTR

hsa-miR-218-1-3p	NM_001135099	TMPRSS2	0.923076923077	3UTR
hsa-miR-222-3p	NM_001135099	TMPRSS2	0.923076923077	3UTR
hsa-miR-224-5p	NM_001135099	TMPRSS2	0.923076923077	3UTR
hsa-let-7g-3p	NM_001135099	TMPRSS2	0.923076923077	3UTR
hsa-let-7i-3p	NM_001135099	TMPRSS2	0.923076923077	3UTR
hsa-miR-15b-5p	NM_001135099	TMPRSS2	0.923076923077	3UTR
hsa-miR-128-1-5p	NM_001135099	TMPRSS2	0.923076923077	3UTR
hsa-miR-128-1-5p	NM_001135099	TMPRSS2	0.923076923077	3UTR
hsa-miR-140-5p	NM_001135099	TMPRSS2	0.923076923077	3UTR
hsa-miR-140-3p	NM_001135099	TMPRSS2	0.923076923077	3UTR
hsa-miR-9-5p	NM_001135099	TMPRSS2	0.923076923077	3UTR
hsa-miR-185-3p	NM_001135099	TMPRSS2	0.923076923077	3UTR
hsa-miR-195-3p	NM_001135099	TMPRSS2	0.923076923077	3UTR
hsa-miR-320a-3p	NM_001135099	TMPRSS2	0.923076923077	3UTR
hsa-miR-194-3p	NM_001135099	TMPRSS2	0.923076923077	3UTR
hsa-miR-106b-5p	NM_001135099	TMPRSS2	0.923076923077	3UTR
hsa-miR-29c-5p	NM_001135099	TMPRSS2	0.923076923077	3UTR
hsa-miR-34c-3p	NM_001135099	TMPRSS2	0.923076923077	3UTR
hsa-miR-296-3p	NM_001135099	TMPRSS2	0.923076923077	3UTR
hsa-miR-296-3p	NM_001135099	TMPRSS2	0.923076923077	3UTR
hsa-miR-365b-5p	NM_001135099	TMPRSS2	0.923076923077	3UTR
hsa-miR-302d-5p	NM_001135099	TMPRSS2	0.923076923077	3UTR
hsa-miR-302d-5p	NM_001135099	TMPRSS2	0.923076923077	3UTR
hsa-miR-367-3p	NM_001135099	TMPRSS2	0.923076923077	3UTR
hsa-miR-370-5p	NM_001135099	TMPRSS2	0.923076923077	3UTR
hsa-miR-370-3p	NM_001135099	TMPRSS2	0.923076923077	3UTR
hsa-miR-371a-5p	NM_001135099	TMPRSS2	0.923076923077	3UTR
hsa-miR-373-5p	NM_001135099	TMPRSS2	0.923076923077	3UTR
hsa-miR-342-5p	NM_001135099	TMPRSS2	0.923076923077	3UTR
hsa-miR-339-5p	NM_001135099	TMPRSS2	0.923076923077	3UTR
hsa-miR-339-5p	NM_001135099	TMPRSS2	0.923076923077	3UTR
hsa-miR-345-3p	NM_001135099	TMPRSS2	0.923076923077	3UTR
hsa-miR-20b-5p	NM_001135099	TMPRSS2	0.923076923077	3UTR
hsa-miR-448	NM_001135099	TMPRSS2	0.923076923077	3UTR
hsa-miR-449a	NM_001135099	TMPRSS2	0.923076923077	3UTR
hsa-miR-449a	NM_001135099	TMPRSS2	0.923076923077	3UTR
hsa-miR-431-3p	NM_001135099	TMPRSS2	0.923076923077	3UTR
hsa-miR-490-5p	NM_001135099	TMPRSS2	0.923076923077	3UTR
hsa-miR-490-3p	NM_001135099	TMPRSS2	0.923076923077	3UTR
hsa-miR-146b-3p	NM_001135099	TMPRSS2	0.923076923077	3UTR
hsa-miR-202-3p	NM_001135099	TMPRSS2	0.923076923077	3UTR
hsa-miR-493-3p	NM_001135099	TMPRSS2	0.923076923077	3UTR
hsa-miR-193b-3p	NM_001135099	TMPRSS2	0.923076923077	3UTR
hsa-miR-512-5p	NM_001135099	TMPRSS2	0.923076923077	3UTR
hsa-miR-520e-5p	NM_001135099	TMPRSS2	0.923076923077	3UTR
hsa-miR-520f-3p	NM_001135099	TMPRSS2	0.923076923077	3UTR
hsa-miR-519c-5p	NM_001135099	TMPRSS2	0.923076923077	3UTR

hsa-miR-520a-5p	NM_001135099	TMPRSS2	0.923076923077	3UTR
hsa-miR-519b-5p	NM_001135099	TMPRSS2	0.923076923077	3UTR
hsa-miR-523-5p	NM_001135099	TMPRSS2	0.923076923077	3UTR
hsa-miR-518f-5p	NM_001135099	TMPRSS2	0.923076923077	3UTR
hsa-miR-519d-5p	NM_001135099	TMPRSS2	0.923076923077	3UTR
hsa-miR-516b-3p	NM_001135099	TMPRSS2	0.923076923077	3UTR
hsa-miR-518e-5p	NM_001135099	TMPRSS2	0.923076923077	3UTR
hsa-miR-518a-5p	NM_001135099	TMPRSS2	0.923076923077	3UTR
hsa-miR-522-5p	NM_001135099	TMPRSS2	0.923076923077	3UTR
hsa-miR-519a-5p	NM_001135099	TMPRSS2	0.923076923077	3UTR
hsa-miR-527	NM_001135099	TMPRSS2	0.923076923077	3UTR
hsa-miR-516a-3p	NM_001135099	TMPRSS2	0.923076923077	3UTR
hsa-miR-500a-3p	NM_001135099	TMPRSS2	0.923076923077	3UTR
hsa-miR-450a-2-3p	NM_001135099	TMPRSS2	0.923076923077	3UTR
hsa-miR-510-3p	NM_001135099	TMPRSS2	0.923076923077	3UTR
hsa-miR-514a-5p	NM_001135099	TMPRSS2	0.923076923077	3UTR
hsa-miR-532-3p	NM_001135099	TMPRSS2	0.923076923077	3UTR
hsa-miR-557	NM_001135099	TMPRSS2	0.923076923077	3UTR
hsa-miR-548a-3p	NM_001135099	TMPRSS2	0.923076923077	3UTR
hsa-miR-591	NM_001135099	TMPRSS2	0.923076923077	3UTR
hsa-miR-595	NM_001135099	TMPRSS2	0.923076923077	3UTR
hsa-miR-598-3p	NM_001135099	TMPRSS2	0.923076923077	3UTR
hsa-miR-604	NM_001135099	TMPRSS2	0.923076923077	3UTR
hsa-miR-616-5p	NM_001135099	TMPRSS2	0.923076923077	3UTR
hsa-miR-617	NM_001135099	TMPRSS2	0.923076923077	3UTR
hsa-miR-618	NM_001135099	TMPRSS2	0.923076923077	3UTR
hsa-miR-619-5p	NM_001135099	TMPRSS2	0.923076923077	3UTR
hsa-miR-619-5p	NM_001135099	TMPRSS2	0.923076923077	3UTR
hsa-miR-623	NM_001135099	TMPRSS2	0.923076923077	3UTR
hsa-miR-625-5p	NM_001135099	TMPRSS2	0.923076923077	3UTR
hsa-miR-628-3p	NM_001135099	TMPRSS2	0.923076923077	3UTR
hsa-miR-630	NM_001135099	TMPRSS2	0.923076923077	3UTR
hsa-miR-33b-3p	NM_001135099	TMPRSS2	0.923076923077	3UTR
hsa-miR-639	NM_001135099	TMPRSS2	0.923076923077	3UTR
hsa-miR-647	NM_001135099	TMPRSS2	0.923076923077	3UTR
hsa-miR-648	NM_001135099	TMPRSS2	0.923076923077	3UTR
hsa-miR-650	NM_001135099	TMPRSS2	0.923076923077	3UTR
hsa-miR-650	NM_001135099	TMPRSS2	0.923076923077	3UTR
hsa-miR-652-3p	NM_001135099	TMPRSS2	0.923076923077	3UTR
hsa-miR-652-3p	NM_001135099	TMPRSS2	0.923076923077	3UTR
hsa-miR-449b-5p	NM_001135099	TMPRSS2	0.923076923077	3UTR
hsa-miR-654-3p	NM_001135099	TMPRSS2	0.923076923077	3UTR
hsa-miR-655-5p	NM_001135099	TMPRSS2	0.923076923077	3UTR
hsa-miR-549a-5p	NM_001135099	TMPRSS2	0.923076923077	3UTR
hsa-miR-659-3p	NM_001135099	TMPRSS2	0.923076923077	3UTR
hsa-miR-542-5p	NM_001135099	TMPRSS2	0.923076923077	3UTR
hsa-miR-671-5p	NM_001135099	TMPRSS2	0.923076923077	3UTR

hsa-miR-767-3p	NM_001135099	TMPRSS2	0.923076923077	3UTR
hsa-miR-151b	NM_001135099	TMPRSS2	0.923076923077	3UTR
hsa-miR-320b	NM_001135099	TMPRSS2	0.923076923077	3UTR
hsa-miR-320c	NM_001135099	TMPRSS2	0.923076923077	3UTR
hsa-miR-1323	NM_001135099	TMPRSS2	0.923076923077	3UTR
hsa-miR-1271-3p	NM_001135099	TMPRSS2	0.923076923077	3UTR
hsa-miR-1301-5p	NM_001135099	TMPRSS2	0.923076923077	3UTR
hsa-miR-378d	NM_001135099	TMPRSS2	0.923076923077	3UTR
hsa-miR-770-5p	NM_001135099	TMPRSS2	0.923076923077	3UTR
hsa-miR-675-3p	NM_001135099	TMPRSS2	0.923076923077	3UTR
hsa-miR-885-5p	NM_001135099	TMPRSS2	0.923076923077	3UTR
hsa-miR-873-5p	NM_001135099	TMPRSS2	0.923076923077	3UTR
hsa-miR-921	NM_001135099	TMPRSS2	0.923076923077	3UTR
hsa-miR-921	NM_001135099	TMPRSS2	0.923076923077	3UTR
hsa-miR-933	NM_001135099	TMPRSS2	0.923076923077	3UTR
hsa-miR-939-5p	NM_001135099	TMPRSS2	0.923076923077	3UTR
hsa-miR-1180-5p	NM_001135099	TMPRSS2	0.923076923077	3UTR
hsa-miR-1183	NM_001135099	TMPRSS2	0.923076923077	3UTR
hsa-miR-1184	NM_001135099	TMPRSS2	0.923076923077	3UTR
hsa-miR-1229-3p	NM_001135099	TMPRSS2	0.923076923077	3UTR
hsa-miR-1233-3p	NM_001135099	TMPRSS2	0.923076923077	3UTR
hsa-miR-1236-5p	NM_001135099	TMPRSS2	0.923076923077	3UTR
hsa-miR-1236-3p	NM_001135099	TMPRSS2	0.923076923077	3UTR
hsa-miR-1236-3p	NM_001135099	TMPRSS2	0.923076923077	3UTR
hsa-miR-1205	NM_001135099	TMPRSS2	0.923076923077	3UTR
hsa-miR-1207-5p	NM_001135099	TMPRSS2	0.923076923077	3UTR
hsa-miR-1285-5p	NM_001135099	TMPRSS2	0.923076923077	3UTR
hsa-miR-1286	NM_001135099	TMPRSS2	0.923076923077	3UTR
hsa-miR-1289	NM_001135099	TMPRSS2	0.923076923077	3UTR
hsa-miR-1291	NM_001135099	TMPRSS2	0.923076923077	3UTR
hsa-miR-1295a	NM_001135099	TMPRSS2	0.923076923077	3UTR
hsa-miR-1245a	NM_001135099	TMPRSS2	0.923076923077	3UTR
hsa-miR-1249-5p	NM_001135099	TMPRSS2	0.923076923077	3UTR
hsa-miR-1249-3p	NM_001135099	TMPRSS2	0.923076923077	3UTR
hsa-miR-1256	NM_001135099	TMPRSS2	0.923076923077	3UTR
hsa-miR-1258	NM_001135099	TMPRSS2	0.923076923077	3UTR
hsa-miR-1276	NM_001135099	TMPRSS2	0.923076923077	3UTR
hsa-miR-1306-5p	NM_001135099	TMPRSS2	0.923076923077	3UTR
hsa-miR-1539	NM_001135099	TMPRSS2	0.923076923077	3UTR
hsa-miR-320d	NM_001135099	TMPRSS2	0.923076923077	3UTR
hsa-miR-1912-5p	NM_001135099	TMPRSS2	0.923076923077	3UTR
hsa-miR-1915-3p	NM_001135099	TMPRSS2	0.923076923077	3UTR
hsa-miR-2277-3p	NM_001135099	TMPRSS2	0.923076923077	3UTR
hsa-miR-2682-5p	NM_001135099	TMPRSS2	0.923076923077	3UTR
hsa-miR-711	NM_001135099	TMPRSS2	0.923076923077	3UTR
hsa-miR-3117-3p	NM_001135099	TMPRSS2	0.923076923077	3UTR
hsa-miR-3124-5p	NM_001135099	TMPRSS2	0.923076923077	3UTR

hsa-miR-3127-3p	NM_001135099	TMPRSS2	0.923076923077	3UTR
hsa-miR-3129-3p	NM_001135099	TMPRSS2	0.923076923077	3UTR
hsa-miR-378b	NM_001135099	TMPRSS2	0.923076923077	3UTR
hsa-miR-3141	NM_001135099	TMPRSS2	0.923076923077	3UTR
hsa-miR-1273c	NM_001135099	TMPRSS2	0.923076923077	3UTR
hsa-miR-3147	NM_001135099	TMPRSS2	0.923076923077	3UTR
hsa-miR-3152-3p	NM_001135099	TMPRSS2	0.923076923077	3UTR
hsa-miR-3153	NM_001135099	TMPRSS2	0.923076923077	3UTR
hsa-miR-3154	NM_001135099	TMPRSS2	0.923076923077	3UTR
hsa-miR-3154	NM_001135099	TMPRSS2	0.923076923077	3UTR
hsa-miR-3154	NM_001135099	TMPRSS2	0.923076923077	3UTR
hsa-miR-3155a	NM_001135099	TMPRSS2	0.923076923077	3UTR
hsa-miR-3156-5p	NM_001135099	TMPRSS2	0.923076923077	3UTR
hsa-miR-3159	NM_001135099	TMPRSS2	0.923076923077	3UTR
hsa-miR-3162-3p	NM_001135099	TMPRSS2	0.923076923077	3UTR
hsa-miR-3163	NM_001135099	TMPRSS2	0.923076923077	3UTR
hsa-miR-3168	NM_001135099	TMPRSS2	0.923076923077	3UTR
hsa-miR-3169	NM_001135099	TMPRSS2	0.923076923077	3UTR
hsa-miR-3173-5p	NM_001135099	TMPRSS2	0.923076923077	3UTR
hsa-miR-3173-5p	NM_001135099	TMPRSS2	0.923076923077	3UTR
hsa-miR-3173-3p	NM_001135099	TMPRSS2	0.923076923077	3UTR
hsa-miR-3180-5p	NM_001135099	TMPRSS2	0.923076923077	3UTR
hsa-miR-3184-5p	NM_001135099	TMPRSS2	0.923076923077	3UTR
hsa-miR-3185	NM_001135099	TMPRSS2	0.923076923077	3UTR
hsa-miR-3189-3p	NM_001135099	TMPRSS2	0.923076923077	3UTR
hsa-miR-3191-3p	NM_001135099	TMPRSS2	0.923076923077	3UTR
hsa-miR-3191-3p	NM_001135099	TMPRSS2	0.923076923077	3UTR
hsa-miR-3192-5p	NM_001135099	TMPRSS2	0.923076923077	3UTR
hsa-miR-3194-5p	NM_001135099	TMPRSS2	0.923076923077	3UTR
hsa-miR-3198	NM_001135099	TMPRSS2	0.923076923077	3UTR
hsa-miR-514b-5p	NM_001135099	TMPRSS2	0.923076923077	3UTR
hsa-miR-3202	NM_001135099	TMPRSS2	0.923076923077	3UTR
hsa-miR-4299	NM_001135099	TMPRSS2	0.923076923077	3UTR
hsa-miR-4298	NM_001135099	TMPRSS2	0.923076923077	3UTR
hsa-miR-4298	NM_001135099	TMPRSS2	0.923076923077	3UTR
hsa-miR-4304	NM_001135099	TMPRSS2	0.923076923077	3UTR
hsa-miR-4316	NM_001135099	TMPRSS2	0.923076923077	3UTR
hsa-miR-4321	NM_001135099	TMPRSS2	0.923076923077	3UTR
hsa-miR-4257	NM_001135099	TMPRSS2	0.923076923077	3UTR
hsa-miR-4254	NM_001135099	TMPRSS2	0.923076923077	3UTR
hsa-miR-4252	NM_001135099	TMPRSS2	0.923076923077	3UTR
hsa-miR-4261	NM_001135099	TMPRSS2	0.923076923077	3UTR
hsa-miR-4270	NM_001135099	TMPRSS2	0.923076923077	3UTR
hsa-miR-4278	NM_001135099	TMPRSS2	0.923076923077	3UTR
hsa-miR-4280	NM_001135099	TMPRSS2	0.923076923077	3UTR
hsa-miR-4284	NM_001135099	TMPRSS2	0.923076923077	3UTR
hsa-miR-4290	NM_001135099	TMPRSS2	0.923076923077	3UTR

hsa-miR-3612	NM_001135099	TMPRSS2	0.923076923077	3UTR
hsa-miR-3619-3p	NM_001135099	TMPRSS2	0.923076923077	3UTR
hsa-miR-3621	NM_001135099	TMPRSS2	0.923076923077	3UTR
hsa-miR-3649	NM_001135099	TMPRSS2	0.923076923077	3UTR
hsa-miR-3652	NM_001135099	TMPRSS2	0.923076923077	3UTR
hsa-miR-3659	NM_001135099	TMPRSS2	0.923076923077	3UTR
hsa-miR-3661	NM_001135099	TMPRSS2	0.923076923077	3UTR
hsa-miR-3663-5p	NM_001135099	TMPRSS2	0.923076923077	3UTR
hsa-miR-3665	NM_001135099	TMPRSS2	0.923076923077	3UTR
hsa-miR-3667-3p	NM_001135099	TMPRSS2	0.923076923077	3UTR
hsa-miR-3677-3p	NM_001135099	TMPRSS2	0.923076923077	3UTR
hsa-miR-3678-3p	NM_001135099	TMPRSS2	0.923076923077	3UTR
hsa-miR-3679-5p	NM_001135099	TMPRSS2	0.923076923077	3UTR
hsa-miR-3680-3p	NM_001135099	TMPRSS2	0.923076923077	3UTR
hsa-miR-3689a-5p	NM_001135099	TMPRSS2	0.923076923077	3UTR
hsa-miR-3692-5p	NM_001135099	TMPRSS2	0.923076923077	3UTR
hsa-miR-3692-3p	NM_001135099	TMPRSS2	0.923076923077	3UTR
hsa-miR-3689b-5p	NM_001135099	TMPRSS2	0.923076923077	3UTR
hsa-miR-3908	NM_001135099	TMPRSS2	0.923076923077	3UTR
hsa-miR-3917	NM_001135099	TMPRSS2	0.923076923077	3UTR
hsa-miR-3919	NM_001135099	TMPRSS2	0.923076923077	3UTR
hsa-miR-3934-3p	NM_001135099	TMPRSS2	0.923076923077	3UTR
hsa-miR-3935	NM_001135099	TMPRSS2	0.923076923077	3UTR
hsa-miR-3943	NM_001135099	TMPRSS2	0.923076923077	3UTR
hsa-miR-3945	NM_001135099	TMPRSS2	0.923076923077	3UTR
hsa-miR-4425	NM_001135099	TMPRSS2	0.923076923077	3UTR
hsa-miR-4428	NM_001135099	TMPRSS2	0.923076923077	3UTR
hsa-miR-4429	NM_001135099	TMPRSS2	0.923076923077	3UTR
hsa-miR-4442	NM_001135099	TMPRSS2	0.923076923077	3UTR
hsa-miR-4448	NM_001135099	TMPRSS2	0.923076923077	3UTR
hsa-miR-548ah-5p	NM_001135099	TMPRSS2	0.923076923077	3UTR
hsa-miR-4451	NM_001135099	TMPRSS2	0.923076923077	3UTR
hsa-miR-4458	NM_001135099	TMPRSS2	0.923076923077	3UTR
hsa-miR-4466	NM_001135099	TMPRSS2	0.923076923077	3UTR
hsa-miR-4471	NM_001135099	TMPRSS2	0.923076923077	3UTR
hsa-miR-4476	NM_001135099	TMPRSS2	0.923076923077	3UTR
hsa-miR-3689e	NM_001135099	TMPRSS2	0.923076923077	3UTR
hsa-miR-4480	NM_001135099	TMPRSS2	0.923076923077	3UTR
hsa-miR-4482-3p	NM_001135099	TMPRSS2	0.923076923077	3UTR
hsa-miR-4494	NM_001135099	TMPRSS2	0.923076923077	3UTR
hsa-miR-4500	NM_001135099	TMPRSS2	0.923076923077	3UTR
hsa-miR-4515	NM_001135099	TMPRSS2	0.923076923077	3UTR
hsa-miR-4517	NM_001135099	TMPRSS2	0.923076923077	3UTR
hsa-miR-4524a-5p	NM_001135099	TMPRSS2	0.923076923077	3UTR
hsa-miR-4530	NM_001135099	TMPRSS2	0.923076923077	3UTR
hsa-miR-4534	NM_001135099	TMPRSS2	0.923076923077	3UTR
hsa-miR-378i	NM_001135099	TMPRSS2	0.923076923077	3UTR

hsa-miR-378i	NM_001135099	TMPRSS2	0.923076923077	3UTR
hsa-miR-4537	NM_001135099	TMPRSS2	0.923076923077	3UTR
hsa-miR-3978	NM_001135099	TMPRSS2	0.923076923077	3UTR
hsa-miR-4632-5p	NM_001135099	TMPRSS2	0.923076923077	3UTR
hsa-miR-4640-3p	NM_001135099	TMPRSS2	0.923076923077	3UTR
hsa-miR-4650-3p	NM_001135099	TMPRSS2	0.923076923077	3UTR
hsa-miR-4651	NM_001135099	TMPRSS2	0.923076923077	3UTR
hsa-miR-4655-5p	NM_001135099	TMPRSS2	0.923076923077	3UTR
hsa-miR-4656	NM_001135099	TMPRSS2	0.923076923077	3UTR
hsa-miR-4657	NM_001135099	TMPRSS2	0.923076923077	3UTR
hsa-miR-4661-3p	NM_001135099	TMPRSS2	0.923076923077	3UTR
hsa-miR-4663	NM_001135099	TMPRSS2	0.923076923077	3UTR
hsa-miR-4663	NM_001135099	TMPRSS2	0.923076923077	3UTR
hsa-miR-4664-5p	NM_001135099	TMPRSS2	0.923076923077	3UTR
hsa-miR-4667-5p	NM_001135099	TMPRSS2	0.923076923077	3UTR
hsa-miR-4667-3p	NM_001135099	TMPRSS2	0.923076923077	3UTR
hsa-miR-4687-5p	NM_001135099	TMPRSS2	0.923076923077	3UTR
hsa-miR-4687-3p	NM_001135099	TMPRSS2	0.923076923077	3UTR
hsa-miR-4689	NM_001135099	TMPRSS2	0.923076923077	3UTR
hsa-miR-4690-5p	NM_001135099	TMPRSS2	0.923076923077	3UTR
hsa-miR-4691-3p	NM_001135099	TMPRSS2	0.923076923077	3UTR
hsa-miR-4691-3p	NM_001135099	TMPRSS2	0.923076923077	3UTR
hsa-miR-4695-5p	NM_001135099	TMPRSS2	0.923076923077	3UTR
hsa-miR-4699-3p	NM_001135099	TMPRSS2	0.923076923077	3UTR
hsa-miR-4700-3p	NM_001135099	TMPRSS2	0.923076923077	3UTR
hsa-miR-4709-3p	NM_001135099	TMPRSS2	0.923076923077	3UTR
hsa-miR-4710	NM_001135099	TMPRSS2	0.923076923077	3UTR
hsa-miR-4711-3p	NM_001135099	TMPRSS2	0.923076923077	3UTR
hsa-miR-4713-3p	NM_001135099	TMPRSS2	0.923076923077	3UTR
hsa-miR-4715-3p	NM_001135099	TMPRSS2	0.923076923077	3UTR
hsa-miR-4723-3p	NM_001135099	TMPRSS2	0.923076923077	3UTR
hsa-miR-4726-5p	NM_001135099	TMPRSS2	0.923076923077	3UTR
hsa-miR-4730	NM_001135099	TMPRSS2	0.923076923077	3UTR
hsa-miR-4731-3p	NM_001135099	TMPRSS2	0.923076923077	3UTR
hsa-miR-4732-5p	NM_001135099	TMPRSS2	0.923076923077	3UTR
hsa-miR-3064-5p	NM_001135099	TMPRSS2	0.923076923077	3UTR
hsa-miR-4738-3p	NM_001135099	TMPRSS2	0.923076923077	3UTR
hsa-miR-4738-3p	NM_001135099	TMPRSS2	0.923076923077	3UTR
hsa-miR-4744	NM_001135099	TMPRSS2	0.923076923077	3UTR
hsa-miR-4746-5p	NM_001135099	TMPRSS2	0.923076923077	3UTR
hsa-miR-4747-5p	NM_001135099	TMPRSS2	0.923076923077	3UTR
hsa-miR-4748	NM_001135099	TMPRSS2	0.923076923077	3UTR
hsa-miR-4750-3p	NM_001135099	TMPRSS2	0.923076923077	3UTR
hsa-miR-4755-5p	NM_001135099	TMPRSS2	0.923076923077	3UTR
hsa-miR-4756-3p	NM_001135099	TMPRSS2	0.923076923077	3UTR
hsa-miR-4772-3p	NM_001135099	TMPRSS2	0.923076923077	3UTR
hsa-miR-4773	NM_001135099	TMPRSS2	0.923076923077	3UTR

hsa-miR-4775	NM_001135099	TMPRSS2	0.923076923077	3UTR
hsa-miR-4776-5p	NM_001135099	TMPRSS2	0.923076923077	3UTR
hsa-miR-1245b-3p	NM_001135099	TMPRSS2	0.923076923077	3UTR
hsa-miR-4786-3p	NM_001135099	TMPRSS2	0.923076923077	3UTR
hsa-miR-4788	NM_001135099	TMPRSS2	0.923076923077	3UTR
hsa-miR-4790-3p	NM_001135099	TMPRSS2	0.923076923077	3UTR
hsa-miR-4793-5p	NM_001135099	TMPRSS2	0.923076923077	3UTR
hsa-miR-4796-5p	NM_001135099	TMPRSS2	0.923076923077	3UTR
hsa-miR-4797-5p	NM_001135099	TMPRSS2	0.923076923077	3UTR
hsa-miR-4799-3p	NM_001135099	TMPRSS2	0.923076923077	3UTR
hsa-miR-4800-5p	NM_001135099	TMPRSS2	0.923076923077	3UTR
hsa-miR-5001-3p	NM_001135099	TMPRSS2	0.923076923077	3UTR
hsa-miR-5002-5p	NM_001135099	TMPRSS2	0.923076923077	3UTR
hsa-miR-5004-5p	NM_001135099	TMPRSS2	0.923076923077	3UTR
hsa-miR-5008-3p	NM_001135099	TMPRSS2	0.923076923077	3UTR
hsa-miR-5047	NM_001135099	TMPRSS2	0.923076923077	3UTR
hsa-miR-5087	NM_001135099	TMPRSS2	0.923076923077	3UTR
hsa-miR-5089-5p	NM_001135099	TMPRSS2	0.923076923077	3UTR
hsa-miR-5090	NM_001135099	TMPRSS2	0.923076923077	3UTR
hsa-miR-5187-3p	NM_001135099	TMPRSS2	0.923076923077	3UTR
hsa-miR-5193	NM_001135099	TMPRSS2	0.923076923077	3UTR
hsa-miR-5195-3p	NM_001135099	TMPRSS2	0.923076923077	3UTR
hsa-miR-5196-5p	NM_001135099	TMPRSS2	0.923076923077	3UTR
hsa-miR-5197-3p	NM_001135099	TMPRSS2	0.923076923077	3UTR
hsa-miR-5580-3p	NM_001135099	TMPRSS2	0.923076923077	3UTR
hsa-miR-5585-3p	NM_001135099	TMPRSS2	0.923076923077	3UTR
hsa-miR-5589-5p	NM_001135099	TMPRSS2	0.923076923077	3UTR
hsa-miR-5591-5p	NM_001135099	TMPRSS2	0.923076923077	3UTR
hsa-miR-5591-5p	NM_001135099	TMPRSS2	0.923076923077	3UTR
hsa-miR-5591-3p	NM_001135099	TMPRSS2	0.923076923077	3UTR
hsa-miR-5682	NM_001135099	TMPRSS2	0.923076923077	3UTR
hsa-miR-5682	NM_001135099	TMPRSS2	0.923076923077	3UTR
hsa-miR-5687	NM_001135099	TMPRSS2	0.923076923077	3UTR
hsa-miR-5693	NM_001135099	TMPRSS2	0.923076923077	3UTR
hsa-miR-5704	NM_001135099	TMPRSS2	0.923076923077	3UTR
hsa-miR-5708	NM_001135099	TMPRSS2	0.923076923077	3UTR
hsa-miR-5739	NM_001135099	TMPRSS2	0.923076923077	3UTR
hsa-miR-5739	NM_001135099	TMPRSS2	0.923076923077	3UTR
hsa-miR-1199-3p	NM_001135099	TMPRSS2	0.923076923077	3UTR
hsa-miR-6081	NM_001135099	TMPRSS2	0.923076923077	3UTR
hsa-miR-6085	NM_001135099	TMPRSS2	0.923076923077	3UTR
hsa-miR-6088	NM_001135099	TMPRSS2	0.923076923077	3UTR
hsa-miR-6127	NM_001135099	TMPRSS2	0.923076923077	3UTR
hsa-miR-6127	NM_001135099	TMPRSS2	0.923076923077	3UTR
hsa-miR-378j	NM_001135099	TMPRSS2	0.923076923077	3UTR
hsa-miR-6131	NM_001135099	TMPRSS2	0.923076923077	3UTR
hsa-miR-6134	NM_001135099	TMPRSS2	0.923076923077	3UTR

hsa-miR-6165	NM_001135099	TMPRSS2	0.923076923077	3UTR
hsa-miR-6165	NM_001135099	TMPRSS2	0.923076923077	3UTR
hsa-miR-6499-5p	NM_001135099	TMPRSS2	0.923076923077	3UTR
hsa-miR-6503-3p	NM_001135099	TMPRSS2	0.923076923077	3UTR
hsa-miR-6509-3p	NM_001135099	TMPRSS2	0.923076923077	3UTR
hsa-miR-6510-5p	NM_001135099	TMPRSS2	0.923076923077	3UTR
hsa-miR-6511a-3p	NM_001135099	TMPRSS2	0.923076923077	3UTR
hsa-miR-6515-5p	NM_001135099	TMPRSS2	0.923076923077	3UTR
hsa-miR-6515-5p	NM_001135099	TMPRSS2	0.923076923077	3UTR
hsa-miR-6715b-3p	NM_001135099	TMPRSS2	0.923076923077	3UTR
hsa-miR-6716-3p	NM_001135099	TMPRSS2	0.923076923077	3UTR
hsa-miR-6717-5p	NM_001135099	TMPRSS2	0.923076923077	3UTR
hsa-miR-6511b-5p	NM_001135099	TMPRSS2	0.923076923077	3UTR
hsa-miR-6511b-5p	NM_001135099	TMPRSS2	0.923076923077	3UTR
hsa-miR-6719-3p	NM_001135099	TMPRSS2	0.923076923077	3UTR
hsa-miR-6720-5p	NM_001135099	TMPRSS2	0.923076923077	3UTR
hsa-miR-6726-3p	NM_001135099	TMPRSS2	0.923076923077	3UTR
hsa-miR-6728-5p	NM_001135099	TMPRSS2	0.923076923077	3UTR
hsa-miR-6728-5p	NM_001135099	TMPRSS2	0.923076923077	3UTR
hsa-miR-6731-3p	NM_001135099	TMPRSS2	0.923076923077	3UTR
hsa-miR-6732-5p	NM_001135099	TMPRSS2	0.923076923077	3UTR
hsa-miR-6734-5p	NM_001135099	TMPRSS2	0.923076923077	3UTR
hsa-miR-6741-5p	NM_001135099	TMPRSS2	0.923076923077	3UTR
hsa-miR-6746-5p	NM_001135099	TMPRSS2	0.923076923077	3UTR
hsa-miR-6747-5p	NM_001135099	TMPRSS2	0.923076923077	3UTR
hsa-miR-6747-5p	NM_001135099	TMPRSS2	0.923076923077	3UTR
hsa-miR-6747-3p	NM_001135099	TMPRSS2	0.923076923077	3UTR
hsa-miR-6747-3p	NM_001135099	TMPRSS2	0.923076923077	3UTR
hsa-miR-6748-5p	NM_001135099	TMPRSS2	0.923076923077	3UTR
hsa-miR-6749-5p	NM_001135099	TMPRSS2	0.923076923077	3UTR
hsa-miR-6750-5p	NM_001135099	TMPRSS2	0.923076923077	3UTR
hsa-miR-6751-3p	NM_001135099	TMPRSS2	0.923076923077	3UTR
hsa-miR-6753-5p	NM_001135099	TMPRSS2	0.923076923077	3UTR
hsa-miR-6758-5p	NM_001135099	TMPRSS2	0.923076923077	3UTR
hsa-miR-6760-3p	NM_001135099	TMPRSS2	0.923076923077	3UTR
hsa-miR-6762-3p	NM_001135099	TMPRSS2	0.923076923077	3UTR
hsa-miR-6763-5p	NM_001135099	TMPRSS2	0.923076923077	3UTR
hsa-miR-6763-5p	NM_001135099	TMPRSS2	0.923076923077	3UTR
hsa-miR-6765-5p	NM_001135099	TMPRSS2	0.923076923077	3UTR
hsa-miR-6765-3p	NM_001135099	TMPRSS2	0.923076923077	3UTR
hsa-miR-6767-3p	NM_001135099	TMPRSS2	0.923076923077	3UTR
hsa-miR-6769a-3p	NM_001135099	TMPRSS2	0.923076923077	3UTR
hsa-miR-6771-5p	NM_001135099	TMPRSS2	0.923076923077	3UTR
hsa-miR-6773-3p	NM_001135099	TMPRSS2	0.923076923077	3UTR
hsa-miR-6774-3p	NM_001135099	TMPRSS2	0.923076923077	3UTR
hsa-miR-6776-3p	NM_001135099	TMPRSS2	0.923076923077	3UTR
hsa-miR-6776-3p	NM_001135099	TMPRSS2	0.923076923077	3UTR

hsa-miR-6777-5p	NM_001135099	TMPRSS2	0.923076923077	3UTR
hsa-miR-6779-3p	NM_001135099	TMPRSS2	0.923076923077	3UTR
hsa-miR-6780a-3p	NM_001135099	TMPRSS2	0.923076923077	3UTR
hsa-miR-6781-3p	NM_001135099	TMPRSS2	0.923076923077	3UTR
hsa-miR-6785-5p	NM_001135099	TMPRSS2	0.923076923077	3UTR
hsa-miR-6791-3p	NM_001135099	TMPRSS2	0.923076923077	3UTR
hsa-miR-6792-3p	NM_001135099	TMPRSS2	0.923076923077	3UTR
hsa-miR-6793-3p	NM_001135099	TMPRSS2	0.923076923077	3UTR
hsa-miR-6795-5p	NM_001135099	TMPRSS2	0.923076923077	3UTR
hsa-miR-6797-5p	NM_001135099	TMPRSS2	0.923076923077	3UTR
hsa-miR-6797-3p	NM_001135099	TMPRSS2	0.923076923077	3UTR
hsa-miR-6798-5p	NM_001135099	TMPRSS2	0.923076923077	3UTR
hsa-miR-6799-5p	NM_001135099	TMPRSS2	0.923076923077	3UTR
hsa-miR-6799-3p	NM_001135099	TMPRSS2	0.923076923077	3UTR
hsa-miR-6801-3p	NM_001135099	TMPRSS2	0.923076923077	3UTR
hsa-miR-6802-5p	NM_001135099	TMPRSS2	0.923076923077	3UTR
hsa-miR-6803-3p	NM_001135099	TMPRSS2	0.923076923077	3UTR
hsa-miR-6804-5p	NM_001135099	TMPRSS2	0.923076923077	3UTR
hsa-miR-6805-5p	NM_001135099	TMPRSS2	0.923076923077	3UTR
hsa-miR-6807-3p	NM_001135099	TMPRSS2	0.923076923077	3UTR
hsa-miR-6808-5p	NM_001135099	TMPRSS2	0.923076923077	3UTR
hsa-miR-6809-5p	NM_001135099	TMPRSS2	0.923076923077	3UTR
hsa-miR-6810-5p	NM_001135099	TMPRSS2	0.923076923077	3UTR
hsa-miR-6811-3p	NM_001135099	TMPRSS2	0.923076923077	3UTR
hsa-miR-6812-5p	NM_001135099	TMPRSS2	0.923076923077	3UTR
hsa-miR-6812-3p	NM_001135099	TMPRSS2	0.923076923077	3UTR
hsa-miR-6814-3p	NM_001135099	TMPRSS2	0.923076923077	3UTR
hsa-miR-6815-5p	NM_001135099	TMPRSS2	0.923076923077	3UTR
hsa-miR-6815-3p	NM_001135099	TMPRSS2	0.923076923077	3UTR
hsa-miR-6817-5p	NM_001135099	TMPRSS2	0.923076923077	3UTR
hsa-miR-6819-5p	NM_001135099	TMPRSS2	0.923076923077	3UTR
hsa-miR-6821-3p	NM_001135099	TMPRSS2	0.923076923077	3UTR
hsa-miR-6823-5p	NM_001135099	TMPRSS2	0.923076923077	3UTR
hsa-miR-6825-5p	NM_001135099	TMPRSS2	0.923076923077	3UTR
hsa-miR-6829-3p	NM_001135099	TMPRSS2	0.923076923077	3UTR
hsa-miR-6832-5p	NM_001135099	TMPRSS2	0.923076923077	3UTR
hsa-miR-6832-5p	NM_001135099	TMPRSS2	0.923076923077	3UTR
hsa-miR-6836-3p	NM_001135099	TMPRSS2	0.923076923077	3UTR
hsa-miR-6846-5p	NM_001135099	TMPRSS2	0.923076923077	3UTR
hsa-miR-6846-3p	NM_001135099	TMPRSS2	0.923076923077	3UTR
hsa-miR-6851-5p	NM_001135099	TMPRSS2	0.923076923077	3UTR
hsa-miR-6851-3p	NM_001135099	TMPRSS2	0.923076923077	3UTR
hsa-miR-6854-5p	NM_001135099	TMPRSS2	0.923076923077	3UTR
hsa-miR-6856-5p	NM_001135099	TMPRSS2	0.923076923077	3UTR
hsa-miR-6857-3p	NM_001135099	TMPRSS2	0.923076923077	3UTR
hsa-miR-6859-5p	NM_001135099	TMPRSS2	0.923076923077	3UTR
hsa-miR-6862-5p	NM_001135099	TMPRSS2	0.923076923077	3UTR

hsa-miR-6863	NM_001135099	TMPRSS2	0.923076923077	3UTR
hsa-miR-6864-3p	NM_001135099	TMPRSS2	0.923076923077	3UTR
hsa-miR-6865-5p	NM_001135099	TMPRSS2	0.923076923077	3UTR
hsa-miR-6865-3p	NM_001135099	TMPRSS2	0.923076923077	3UTR
hsa-miR-6867-3p	NM_001135099	TMPRSS2	0.923076923077	3UTR
hsa-miR-6868-5p	NM_001135099	TMPRSS2	0.923076923077	3UTR
hsa-miR-6873-5p	NM_001135099	TMPRSS2	0.923076923077	3UTR
hsa-miR-6873-3p	NM_001135099	TMPRSS2	0.923076923077	3UTR
hsa-miR-6878-3p	NM_001135099	TMPRSS2	0.923076923077	3UTR
hsa-miR-6880-5p	NM_001135099	TMPRSS2	0.923076923077	3UTR
hsa-miR-6883-3p	NM_001135099	TMPRSS2	0.923076923077	3UTR
hsa-miR-6884-5p	NM_001135099	TMPRSS2	0.923076923077	3UTR
hsa-miR-6884-5p	NM_001135099	TMPRSS2	0.923076923077	3UTR
hsa-miR-6886-3p	NM_001135099	TMPRSS2	0.923076923077	3UTR
hsa-miR-6887-5p	NM_001135099	TMPRSS2	0.923076923077	3UTR
hsa-miR-6887-3p	NM_001135099	TMPRSS2	0.923076923077	3UTR
hsa-miR-6890-3p	NM_001135099	TMPRSS2	0.923076923077	3UTR
hsa-miR-6890-3p	NM_001135099	TMPRSS2	0.923076923077	3UTR
hsa-miR-6892-5p	NM_001135099	TMPRSS2	0.923076923077	3UTR
hsa-miR-6892-3p	NM_001135099	TMPRSS2	0.923076923077	3UTR
hsa-miR-6895-3p	NM_001135099	TMPRSS2	0.923076923077	3UTR
hsa-miR-7110-5p	NM_001135099	TMPRSS2	0.923076923077	3UTR
hsa-miR-7111-5p	NM_001135099	TMPRSS2	0.923076923077	3UTR
hsa-miR-7111-3p	NM_001135099	TMPRSS2	0.923076923077	3UTR
hsa-miR-7113-5p	NM_001135099	TMPRSS2	0.923076923077	3UTR
hsa-miR-7114-3p	NM_001135099	TMPRSS2	0.923076923077	3UTR
hsa-miR-7152-3p	NM_001135099	TMPRSS2	0.923076923077	3UTR
hsa-miR-7152-3p	NM_001135099	TMPRSS2	0.923076923077	3UTR
hsa-miR-7152-3p	NM_001135099	TMPRSS2	0.923076923077	3UTR
hsa-miR-7153-3p	NM_001135099	TMPRSS2	0.923076923077	3UTR
hsa-miR-7161-5p	NM_001135099	TMPRSS2	0.923076923077	3UTR
hsa-miR-7161-3p	NM_001135099	TMPRSS2	0.923076923077	3UTR
hsa-miR-7160-3p	NM_001135099	TMPRSS2	0.923076923077	3UTR
hsa-miR-7162-3p	NM_001135099	TMPRSS2	0.923076923077	3UTR
hsa-miR-7703	NM_001135099	TMPRSS2	0.923076923077	3UTR
hsa-miR-7706	NM_001135099	TMPRSS2	0.923076923077	3UTR
hsa-miR-7843-5p	NM_001135099	TMPRSS2	0.923076923077	3UTR
hsa-miR-4433b-5p	NM_001135099	TMPRSS2	0.923076923077	3UTR
hsa-miR-4433b-3p	NM_001135099	TMPRSS2	0.923076923077	3UTR
hsa-miR-4433b-3p	NM_001135099	TMPRSS2	0.923076923077	3UTR
hsa-miR-7848-3p	NM_001135099	TMPRSS2	0.923076923077	3UTR
hsa-miR-7854-3p	NM_001135099	TMPRSS2	0.923076923077	3UTR
hsa-miR-7978	NM_001135099	TMPRSS2	0.923076923077	3UTR
hsa-miR-8077	NM_001135099	TMPRSS2	0.923076923077	3UTR
hsa-miR-8085	NM_001135099	TMPRSS2	0.923076923077	3UTR
hsa-miR-10226	NM_001135099	TMPRSS2	0.923076923077	3UTR
hsa-miR-10392-3p	NM_001135099	TMPRSS2	0.923076923077	3UTR

hsa-miR-10394-5p	NM_001135099	TMPRSS2	0.923076923077	3UTR
hsa-miR-10397-5p	NM_001135099	TMPRSS2	0.923076923077	3UTR
hsa-miR-10399-3p	NM_001135099	TMPRSS2	0.923076923077	3UTR
hsa-miR-10401-5p	NM_001135099	TMPRSS2	0.923076923077	3UTR
hsa-miR-10524-5p	NM_001135099	TMPRSS2	0.923076923077	3UTR
hsa-miR-10526-3p	NM_001135099	TMPRSS2	0.923076923077	3UTR
hsa-miR-11181-5p	NM_001135099	TMPRSS2	0.923076923077	3UTR
hsa-miR-3059-5p	NM_001135099	TMPRSS2	0.923076923077	3UTR
hsa-miR-3059-3p	NM_001135099	TMPRSS2	0.923076923077	3UTR
hsa-miR-3059-3p	NM_001135099	TMPRSS2	0.923076923077	3UTR
hsa-miR-3085-3p	NM_001135099	TMPRSS2	0.923076923077	3UTR
hsa-miR-12115	NM_001135099	TMPRSS2	0.923076923077	3UTR
hsa-miR-12118	NM_001135099	TMPRSS2	0.923076923077	3UTR
hsa-miR-12119	NM_001135099	TMPRSS2	0.923076923077	3UTR
hsa-miR-12122	NM_001135099	TMPRSS2	0.923076923077	3UTR
hsa-miR-12124	NM_001135099	TMPRSS2	0.923076923077	3UTR
hsa-miR-12126	NM_001135099	TMPRSS2	0.923076923077	3UTR
hsa-miR-12128	NM_001135099	TMPRSS2	0.923076923077	3UTR
hsa-miR-12131	NM_001135099	TMPRSS2	0.923076923077	3UTR
hsa-miR-6861-3p	NM_001135099	TMPRSS2	0.935897435897	3UTR
hsa-miR-149-5p	NM_005656	TMPRSS2	0.948717948718	3UTR
hsa-miR-381-3p	NM_005656	TMPRSS2	0.948717948718	3UTR
hsa-miR-3620-5p	NM_005656	TMPRSS2	0.948717948718	3UTR
hsa-miR-6727-5p	NM_005656	TMPRSS2	0.948717948718	3UTR
hsa-miR-6809-3p	NM_005656	TMPRSS2	0.948717948718	3UTR
hsa-miR-149-5p	NM_001135099	TMPRSS2	0.948717948718	3UTR
hsa-miR-6809-3p	NM_001135099	TMPRSS2	0.948717948718	3UTR
hsa-miR-6866-3p	NM_001135099	TMPRSS2	0.948717948718	3UTR
hsa-miR-605-5p	NM_005656	TMPRSS2	0.953846153846	3UTR
hsa-miR-6133	NM_001135099	TMPRSS2	0.953846153846	3UTR
hsa-miR-619-5p	NM_005656	TMPRSS2	0.961538461538	3UTR
hsa-miR-4429	NM_005656	TMPRSS2	0.961538461538	3UTR
hsa-miR-12115	NM_005656	TMPRSS2	0.961538461538	3UTR
hsa-miR-12131	NM_005656	TMPRSS2	0.961538461538	3UTR
hsa-let-7a-5p	NM_001135099	TMPRSS2	0.961538461538	3UTR
hsa-let-7f-5p	NM_001135099	TMPRSS2	0.961538461538	3UTR
hsa-miR-98-5p	NM_001135099	TMPRSS2	0.961538461538	3UTR
hsa-miR-140-5p	NM_001135099	TMPRSS2	0.961538461538	3UTR
hsa-miR-3177-3p	NM_001135099	TMPRSS2	0.961538461538	3UTR
hsa-miR-4448	NM_001135099	TMPRSS2	0.961538461538	3UTR
hsa-miR-4689	NM_001135099	TMPRSS2	0.961538461538	3UTR
hsa-miR-5094	NM_001135099	TMPRSS2	0.961538461538	3UTR
hsa-miR-6812-3p	NM_001135099	TMPRSS2	0.961538461538	3UTR
hsa-miR-6887-5p	NM_001135099	TMPRSS2	0.961538461538	3UTR
hsa-miR-10394-3p	NM_001135099	TMPRSS2	0.961538461538	3UTR
hsa-miR-3929	NM_005656	TMPRSS2	0.969230769231	3UTR
hsa-miR-1304-3p	NM_001135099	TMPRSS2	0.969230769231	3UTR

hsa-miR-3174	NM_001135099	TMPRSS2	0.969230769231	3UTR
hsa-miR-141-3p	NM_005656	TMPRSS2	0.974358974359	3UTR
hsa-miR-452-5p	NM_005656	TMPRSS2	0.974358974359	3UTR
hsa-miR-504-3p	NM_005656	TMPRSS2	0.974358974359	3UTR
hsa-miR-652-3p	NM_005656	TMPRSS2	0.974358974359	3UTR
hsa-miR-4519	NM_005656	TMPRSS2	0.974358974359	3UTR
hsa-miR-6781-3p	NM_005656	TMPRSS2	0.974358974359	3UTR
hsa-miR-452-5p	NM_001135099	TMPRSS2	0.974358974359	3UTR
hsa-miR-504-3p	NM_001135099	TMPRSS2	0.974358974359	3UTR
hsa-miR-652-3p	NM_001135099	TMPRSS2	0.974358974359	3UTR
hsa-miR-1296-3p	NM_001135099	TMPRSS2	0.974358974359	3UTR
hsa-miR-4519	NM_001135099	TMPRSS2	0.974358974359	3UTR
hsa-miR-6781-3p	NM_001135099	TMPRSS2	0.974358974359	3UTR
hsa-let-7b-5p	NM_005656	TMPRSS2	1.0	3UTR
hsa-miR-21-3p	NM_005656	TMPRSS2	1.0	3UTR
hsa-miR-23a-5p	NM_005656	TMPRSS2	1.0	3UTR
hsa-miR-25-5p	NM_005656	TMPRSS2	1.0	3UTR
hsa-miR-26b-3p	NM_005656	TMPRSS2	1.0	3UTR
hsa-miR-28-5p	NM_005656	TMPRSS2	1.0	3UTR
hsa-miR-96-5p	NM_005656	TMPRSS2	1.0	3UTR
hsa-miR-98-5p	NM_005656	TMPRSS2	1.0	3UTR
hsa-miR-101-5p	NM_005656	TMPRSS2	1.0	3UTR
hsa-miR-103a-3p	NM_005656	TMPRSS2	1.0	3UTR
hsa-miR-107	NM_005656	TMPRSS2	1.0	3UTR
hsa-miR-129-1-3p	NM_005656	TMPRSS2	1.0	3UTR
hsa-miR-30d-5p	NM_005656	TMPRSS2	1.0	3UTR
hsa-miR-139-5p	NM_005656	TMPRSS2	1.0	3UTR
hsa-miR-34a-5p	NM_005656	TMPRSS2	1.0	3UTR
hsa-miR-34a-5p	NM_005656	TMPRSS2	1.0	3UTR
hsa-miR-183-5p	NM_005656	TMPRSS2	1.0	3UTR
hsa-miR-200b-5p	NM_005656	TMPRSS2	1.0	3UTR
hsa-let-7g-5p	NM_005656	TMPRSS2	1.0	3UTR
hsa-let-7i-5p	NM_005656	TMPRSS2	1.0	3UTR
hsa-miR-30b-3p	NM_005656	TMPRSS2	1.0	3UTR
hsa-miR-124-5p	NM_005656	TMPRSS2	1.0	3UTR
hsa-miR-140-5p	NM_005656	TMPRSS2	1.0	3UTR
hsa-miR-129-2-3p	NM_005656	TMPRSS2	1.0	3UTR
hsa-miR-134-5p	NM_005656	TMPRSS2	1.0	3UTR
hsa-miR-185-5p	NM_005656	TMPRSS2	1.0	3UTR
hsa-miR-185-3p	NM_005656	TMPRSS2	1.0	3UTR
hsa-miR-188-5p	NM_005656	TMPRSS2	1.0	3UTR
hsa-miR-320a-3p	NM_005656	TMPRSS2	1.0	3UTR
hsa-miR-194-3p	NM_005656	TMPRSS2	1.0	3UTR
hsa-miR-106b-3p	NM_005656	TMPRSS2	1.0	3UTR
hsa-miR-302c-5p	NM_005656	TMPRSS2	1.0	3UTR
hsa-miR-367-3p	NM_005656	TMPRSS2	1.0	3UTR
hsa-miR-371a-5p	NM_005656	TMPRSS2	1.0	3UTR

hsa-miR-373-5p	NM_005656	TMPRSS2	1.0	3UTR
hsa-miR-379-5p	NM_005656	TMPRSS2	1.0	3UTR
hsa-miR-151a-5p	NM_005656	TMPRSS2	1.0	3UTR
hsa-miR-151a-3p	NM_005656	TMPRSS2	1.0	3UTR
hsa-miR-324-5p	NM_005656	TMPRSS2	1.0	3UTR
hsa-miR-324-3p	NM_005656	TMPRSS2	1.0	3UTR
hsa-miR-339-5p	NM_005656	TMPRSS2	1.0	3UTR
hsa-miR-423-5p	NM_005656	TMPRSS2	1.0	3UTR
hsa-miR-423-3p	NM_005656	TMPRSS2	1.0	3UTR
hsa-miR-20b-3p	NM_005656	TMPRSS2	1.0	3UTR
hsa-miR-431-3p	NM_005656	TMPRSS2	1.0	3UTR
hsa-miR-412-3p	NM_005656	TMPRSS2	1.0	3UTR
hsa-miR-491-5p	NM_005656	TMPRSS2	1.0	3UTR
hsa-miR-146b-3p	NM_005656	TMPRSS2	1.0	3UTR
hsa-miR-202-3p	NM_005656	TMPRSS2	1.0	3UTR
hsa-miR-493-3p	NM_005656	TMPRSS2	1.0	3UTR
hsa-miR-432-5p	NM_005656	TMPRSS2	1.0	3UTR
hsa-miR-193b-5p	NM_005656	TMPRSS2	1.0	3UTR
hsa-miR-181d-5p	NM_005656	TMPRSS2	1.0	3UTR
hsa-miR-520c-3p	NM_005656	TMPRSS2	1.0	3UTR
hsa-miR-518a-5p	NM_005656	TMPRSS2	1.0	3UTR
hsa-miR-527	NM_005656	TMPRSS2	1.0	3UTR
hsa-miR-500a-5p	NM_005656	TMPRSS2	1.0	3UTR
hsa-miR-502-3p	NM_005656	TMPRSS2	1.0	3UTR
hsa-miR-503-3p	NM_005656	TMPRSS2	1.0	3UTR
hsa-miR-504-3p	NM_005656	TMPRSS2	1.0	3UTR
hsa-miR-505-5p	NM_005656	TMPRSS2	1.0	3UTR
hsa-miR-505-5p	NM_005656	TMPRSS2	1.0	3UTR
hsa-miR-505-3p	NM_005656	TMPRSS2	1.0	3UTR
hsa-miR-514a-5p	NM_005656	TMPRSS2	1.0	3UTR
hsa-miR-455-5p	NM_005656	TMPRSS2	1.0	3UTR
hsa-miR-455-3p	NM_005656	TMPRSS2	1.0	3UTR
hsa-miR-554	NM_005656	TMPRSS2	1.0	3UTR
hsa-miR-557	NM_005656	TMPRSS2	1.0	3UTR
hsa-miR-575	NM_005656	TMPRSS2	1.0	3UTR
hsa-miR-584-3p	NM_005656	TMPRSS2	1.0	3UTR
hsa-miR-550a-5p	NM_005656	TMPRSS2	1.0	3UTR
hsa-miR-550a-5p	NM_005656	TMPRSS2	1.0	3UTR
hsa-miR-600	NM_005656	TMPRSS2	1.0	3UTR
hsa-miR-608	NM_005656	TMPRSS2	1.0	3UTR
hsa-miR-608	NM_005656	TMPRSS2	1.0	3UTR
hsa-miR-614	NM_005656	TMPRSS2	1.0	3UTR
hsa-miR-622	NM_005656	TMPRSS2	1.0	3UTR
hsa-miR-645	NM_005656	TMPRSS2	1.0	3UTR
hsa-miR-648	NM_005656	TMPRSS2	1.0	3UTR
hsa-miR-650	NM_005656	TMPRSS2	1.0	3UTR
hsa-miR-449b-5p	NM_005656	TMPRSS2	1.0	3UTR

hsa-miR-549a-5p	NM_005656	TMPRSS2	1.0	3UTR
hsa-miR-671-5p	NM_005656	TMPRSS2	1.0	3UTR
hsa-miR-671-5p	NM_005656	TMPRSS2	1.0	3UTR
hsa-miR-671-3p	NM_005656	TMPRSS2	1.0	3UTR
hsa-miR-767-5p	NM_005656	TMPRSS2	1.0	3UTR
hsa-miR-320b	NM_005656	TMPRSS2	1.0	3UTR
hsa-miR-1296-3p	NM_005656	TMPRSS2	1.0	3UTR
hsa-miR-1271-3p	NM_005656	TMPRSS2	1.0	3UTR
hsa-miR-449c-3p	NM_005656	TMPRSS2	1.0	3UTR
hsa-miR-769-5p	NM_005656	TMPRSS2	1.0	3UTR
hsa-miR-766-5p	NM_005656	TMPRSS2	1.0	3UTR
hsa-miR-675-5p	NM_005656	TMPRSS2	1.0	3UTR
hsa-miR-890	NM_005656	TMPRSS2	1.0	3UTR
hsa-miR-708-5p	NM_005656	TMPRSS2	1.0	3UTR
hsa-miR-147b-3p	NM_005656	TMPRSS2	1.0	3UTR
hsa-miR-921	NM_005656	TMPRSS2	1.0	3UTR
hsa-miR-922	NM_005656	TMPRSS2	1.0	3UTR
hsa-miR-933	NM_005656	TMPRSS2	1.0	3UTR
hsa-miR-933	NM_005656	TMPRSS2	1.0	3UTR
hsa-miR-939-5p	NM_005656	TMPRSS2	1.0	3UTR
hsa-miR-939-5p	NM_005656	TMPRSS2	1.0	3UTR
hsa-miR-943	NM_005656	TMPRSS2	1.0	3UTR
hsa-miR-1180-5p	NM_005656	TMPRSS2	1.0	3UTR
hsa-miR-1181	NM_005656	TMPRSS2	1.0	3UTR
hsa-miR-1227-5p	NM_005656	TMPRSS2	1.0	3UTR
hsa-miR-1227-3p	NM_005656	TMPRSS2	1.0	3UTR
hsa-miR-1229-5p	NM_005656	TMPRSS2	1.0	3UTR
hsa-miR-1234-3p	NM_005656	TMPRSS2	1.0	3UTR
hsa-miR-1234-3p	NM_005656	TMPRSS2	1.0	3UTR
hsa-miR-1207-3p	NM_005656	TMPRSS2	1.0	3UTR
hsa-miR-1286	NM_005656	TMPRSS2	1.0	3UTR
hsa-miR-1304-3p	NM_005656	TMPRSS2	1.0	3UTR
hsa-miR-1245a	NM_005656	TMPRSS2	1.0	3UTR
hsa-miR-1249-3p	NM_005656	TMPRSS2	1.0	3UTR
hsa-miR-1253	NM_005656	TMPRSS2	1.0	3UTR
hsa-miR-1256	NM_005656	TMPRSS2	1.0	3UTR
hsa-miR-1263	NM_005656	TMPRSS2	1.0	3UTR
hsa-miR-1266-5p	NM_005656	TMPRSS2	1.0	3UTR
hsa-miR-1275	NM_005656	TMPRSS2	1.0	3UTR
hsa-miR-1255b-2-3p	NM_005656	TMPRSS2	1.0	3UTR
hsa-miR-103b	NM_005656	TMPRSS2	1.0	3UTR
hsa-miR-1825	NM_005656	TMPRSS2	1.0	3UTR
hsa-miR-1909-5p	NM_005656	TMPRSS2	1.0	3UTR
hsa-miR-1910-3p	NM_005656	TMPRSS2	1.0	3UTR
hsa-miR-2117	NM_005656	TMPRSS2	1.0	3UTR
hsa-miR-2277-5p	NM_005656	TMPRSS2	1.0	3UTR
hsa-miR-2681-5p	NM_005656	TMPRSS2	1.0	3UTR

hsa-miR-2682-3p	NM_005656	TMPRSS2	1.0	3UTR
hsa-miR-3125	NM_005656	TMPRSS2	1.0	3UTR
hsa-miR-3129-5p	NM_005656	TMPRSS2	1.0	3UTR
hsa-miR-3131	NM_005656	TMPRSS2	1.0	3UTR
hsa-miR-3137	NM_005656	TMPRSS2	1.0	3UTR
hsa-miR-3144-5p	NM_005656	TMPRSS2	1.0	3UTR
hsa-miR-3150a-5p	NM_005656	TMPRSS2	1.0	3UTR
hsa-miR-3153	NM_005656	TMPRSS2	1.0	3UTR
hsa-miR-3156-5p	NM_005656	TMPRSS2	1.0	3UTR
hsa-miR-3162-5p	NM_005656	TMPRSS2	1.0	3UTR
hsa-miR-3166	NM_005656	TMPRSS2	1.0	3UTR
hsa-miR-3170	NM_005656	TMPRSS2	1.0	3UTR
hsa-miR-3175	NM_005656	TMPRSS2	1.0	3UTR
hsa-miR-3177-3p	NM_005656	TMPRSS2	1.0	3UTR
hsa-miR-3187-3p	NM_005656	TMPRSS2	1.0	3UTR
hsa-miR-3191-3p	NM_005656	TMPRSS2	1.0	3UTR
hsa-miR-3192-5p	NM_005656	TMPRSS2	1.0	3UTR
hsa-miR-3193	NM_005656	TMPRSS2	1.0	3UTR
hsa-miR-3194-5p	NM_005656	TMPRSS2	1.0	3UTR
hsa-miR-3202	NM_005656	TMPRSS2	1.0	3UTR
hsa-miR-4298	NM_005656	TMPRSS2	1.0	3UTR
hsa-miR-4313	NM_005656	TMPRSS2	1.0	3UTR
hsa-miR-4321	NM_005656	TMPRSS2	1.0	3UTR
hsa-miR-4259	NM_005656	TMPRSS2	1.0	3UTR
hsa-miR-4260	NM_005656	TMPRSS2	1.0	3UTR
hsa-miR-4252	NM_005656	TMPRSS2	1.0	3UTR
hsa-miR-4327	NM_005656	TMPRSS2	1.0	3UTR
hsa-miR-4327	NM_005656	TMPRSS2	1.0	3UTR
hsa-miR-4269	NM_005656	TMPRSS2	1.0	3UTR
hsa-miR-3619-5p	NM_005656	TMPRSS2	1.0	3UTR
hsa-miR-3621	NM_005656	TMPRSS2	1.0	3UTR
hsa-miR-3646	NM_005656	TMPRSS2	1.0	3UTR
hsa-miR-3652	NM_005656	TMPRSS2	1.0	3UTR
hsa-miR-3652	NM_005656	TMPRSS2	1.0	3UTR
hsa-miR-3655	NM_005656	TMPRSS2	1.0	3UTR
hsa-miR-3663-5p	NM_005656	TMPRSS2	1.0	3UTR
hsa-miR-3663-5p	NM_005656	TMPRSS2	1.0	3UTR
hsa-miR-3664-3p	NM_005656	TMPRSS2	1.0	3UTR
hsa-miR-3667-3p	NM_005656	TMPRSS2	1.0	3UTR
hsa-miR-3678-3p	NM_005656	TMPRSS2	1.0	3UTR
hsa-miR-3679-5p	NM_005656	TMPRSS2	1.0	3UTR
hsa-miR-3679-5p	NM_005656	TMPRSS2	1.0	3UTR
hsa-miR-3689a-3p	NM_005656	TMPRSS2	1.0	3UTR
hsa-miR-3907	NM_005656	TMPRSS2	1.0	3UTR
hsa-miR-3917	NM_005656	TMPRSS2	1.0	3UTR
hsa-miR-3150b-5p	NM_005656	TMPRSS2	1.0	3UTR
hsa-miR-3926	NM_005656	TMPRSS2	1.0	3UTR

hsa-miR-3928-3p	NM_005656	TMPRSS2	1.0	3UTR
hsa-miR-3934-3p	NM_005656	TMPRSS2	1.0	3UTR
hsa-miR-3944-5p	NM_005656	TMPRSS2	1.0	3UTR
hsa-miR-642b-3p	NM_005656	TMPRSS2	1.0	3UTR
hsa-miR-642b-3p	NM_005656	TMPRSS2	1.0	3UTR
hsa-miR-550b-2-5p	NM_005656	TMPRSS2	1.0	3UTR
hsa-miR-4418	NM_005656	TMPRSS2	1.0	3UTR
hsa-miR-378f	NM_005656	TMPRSS2	1.0	3UTR
hsa-miR-4425	NM_005656	TMPRSS2	1.0	3UTR
hsa-miR-4428	NM_005656	TMPRSS2	1.0	3UTR
hsa-miR-4433a-3p	NM_005656	TMPRSS2	1.0	3UTR
hsa-miR-4436a	NM_005656	TMPRSS2	1.0	3UTR
hsa-miR-4439	NM_005656	TMPRSS2	1.0	3UTR
hsa-miR-4441	NM_005656	TMPRSS2	1.0	3UTR
hsa-miR-4446-3p	NM_005656	TMPRSS2	1.0	3UTR
hsa-miR-4448	NM_005656	TMPRSS2	1.0	3UTR
hsa-miR-4449	NM_005656	TMPRSS2	1.0	3UTR
hsa-miR-548ah-5p	NM_005656	TMPRSS2	1.0	3UTR
hsa-miR-378h	NM_005656	TMPRSS2	1.0	3UTR
hsa-miR-4469	NM_005656	TMPRSS2	1.0	3UTR
hsa-miR-4472	NM_005656	TMPRSS2	1.0	3UTR
hsa-miR-4481	NM_005656	TMPRSS2	1.0	3UTR
hsa-miR-4484	NM_005656	TMPRSS2	1.0	3UTR
hsa-miR-4488	NM_005656	TMPRSS2	1.0	3UTR
hsa-miR-4489	NM_005656	TMPRSS2	1.0	3UTR
hsa-miR-4501	NM_005656	TMPRSS2	1.0	3UTR
hsa-miR-4505	NM_005656	TMPRSS2	1.0	3UTR
hsa-miR-4516	NM_005656	TMPRSS2	1.0	3UTR
hsa-miR-4517	NM_005656	TMPRSS2	1.0	3UTR
hsa-miR-4525	NM_005656	TMPRSS2	1.0	3UTR
hsa-miR-4533	NM_005656	TMPRSS2	1.0	3UTR
hsa-miR-378i	NM_005656	TMPRSS2	1.0	3UTR
hsa-miR-378i	NM_005656	TMPRSS2	1.0	3UTR
hsa-miR-3978	NM_005656	TMPRSS2	1.0	3UTR
hsa-miR-4632-5p	NM_005656	TMPRSS2	1.0	3UTR
hsa-miR-4646-5p	NM_005656	TMPRSS2	1.0	3UTR
hsa-miR-4651	NM_005656	TMPRSS2	1.0	3UTR
hsa-miR-4653-3p	NM_005656	TMPRSS2	1.0	3UTR
hsa-miR-4657	NM_005656	TMPRSS2	1.0	3UTR
hsa-miR-4669	NM_005656	TMPRSS2	1.0	3UTR
hsa-miR-4685-3p	NM_005656	TMPRSS2	1.0	3UTR
hsa-miR-4688	NM_005656	TMPRSS2	1.0	3UTR
hsa-miR-4691-3p	NM_005656	TMPRSS2	1.0	3UTR
hsa-miR-4695-5p	NM_005656	TMPRSS2	1.0	3UTR
hsa-miR-4698	NM_005656	TMPRSS2	1.0	3UTR
hsa-miR-4700-5p	NM_005656	TMPRSS2	1.0	3UTR
hsa-miR-4705	NM_005656	TMPRSS2	1.0	3UTR

hsa-miR-4716-3p	NM_005656	TMPRSS2	1.0	3UTR
hsa-miR-4717-5p	NM_005656	TMPRSS2	1.0	3UTR
hsa-miR-4723-5p	NM_005656	TMPRSS2	1.0	3UTR
hsa-miR-4726-5p	NM_005656	TMPRSS2	1.0	3UTR
hsa-miR-4741	NM_005656	TMPRSS2	1.0	3UTR
hsa-miR-4746-5p	NM_005656	TMPRSS2	1.0	3UTR
hsa-miR-4747-5p	NM_005656	TMPRSS2	1.0	3UTR
hsa-miR-4747-3p	NM_005656	TMPRSS2	1.0	3UTR
hsa-miR-4750-5p	NM_005656	TMPRSS2	1.0	3UTR
hsa-miR-4761-3p	NM_005656	TMPRSS2	1.0	3UTR
hsa-miR-4763-3p	NM_005656	TMPRSS2	1.0	3UTR
hsa-miR-4779	NM_005656	TMPRSS2	1.0	3UTR
hsa-miR-4436b-3p	NM_005656	TMPRSS2	1.0	3UTR
hsa-miR-1245b-5p	NM_005656	TMPRSS2	1.0	3UTR
hsa-miR-4787-5p	NM_005656	TMPRSS2	1.0	3UTR
hsa-miR-4787-5p	NM_005656	TMPRSS2	1.0	3UTR
hsa-miR-4788	NM_005656	TMPRSS2	1.0	3UTR
hsa-miR-4793-5p	NM_005656	TMPRSS2	1.0	3UTR
hsa-miR-5002-5p	NM_005656	TMPRSS2	1.0	3UTR
hsa-miR-5002-3p	NM_005656	TMPRSS2	1.0	3UTR
hsa-miR-5003-3p	NM_005656	TMPRSS2	1.0	3UTR
hsa-miR-5004-3p	NM_005656	TMPRSS2	1.0	3UTR
hsa-miR-5010-5p	NM_005656	TMPRSS2	1.0	3UTR
hsa-miR-5088-3p	NM_005656	TMPRSS2	1.0	3UTR
hsa-miR-5093	NM_005656	TMPRSS2	1.0	3UTR
hsa-miR-5195-3p	NM_005656	TMPRSS2	1.0	3UTR
hsa-miR-5195-3p	NM_005656	TMPRSS2	1.0	3UTR
hsa-miR-4524b-3p	NM_005656	TMPRSS2	1.0	3UTR
hsa-miR-5581-5p	NM_005656	TMPRSS2	1.0	3UTR
hsa-miR-5585-3p	NM_005656	TMPRSS2	1.0	3UTR
hsa-miR-1295b-3p	NM_005656	TMPRSS2	1.0	3UTR
hsa-miR-5589-3p	NM_005656	TMPRSS2	1.0	3UTR
hsa-miR-5682	NM_005656	TMPRSS2	1.0	3UTR
hsa-miR-5687	NM_005656	TMPRSS2	1.0	3UTR
hsa-miR-5691	NM_005656	TMPRSS2	1.0	3UTR
hsa-miR-5698	NM_005656	TMPRSS2	1.0	3UTR
hsa-miR-1199-3p	NM_005656	TMPRSS2	1.0	3UTR
hsa-miR-6074	NM_005656	TMPRSS2	1.0	3UTR
hsa-miR-6081	NM_005656	TMPRSS2	1.0	3UTR
hsa-miR-6086	NM_005656	TMPRSS2	1.0	3UTR
hsa-miR-6088	NM_005656	TMPRSS2	1.0	3UTR
hsa-miR-6090	NM_005656	TMPRSS2	1.0	3UTR
hsa-miR-6133	NM_005656	TMPRSS2	1.0	3UTR
hsa-miR-6133	NM_005656	TMPRSS2	1.0	3UTR
hsa-miR-6134	NM_005656	TMPRSS2	1.0	3UTR
hsa-miR-6165	NM_005656	TMPRSS2	1.0	3UTR
hsa-miR-6505-5p	NM_005656	TMPRSS2	1.0	3UTR

hsa-miR-6510-5p	NM_005656	TMPRSS2	1.0	3UTR
hsa-miR-6510-5p	NM_005656	TMPRSS2	1.0	3UTR
hsa-miR-6514-5p	NM_005656	TMPRSS2	1.0	3UTR
hsa-miR-6515-5p	NM_005656	TMPRSS2	1.0	3UTR
hsa-miR-6715b-3p	NM_005656	TMPRSS2	1.0	3UTR
hsa-miR-6717-5p	NM_005656	TMPRSS2	1.0	3UTR
hsa-miR-6511b-5p	NM_005656	TMPRSS2	1.0	3UTR
hsa-miR-6721-5p	NM_005656	TMPRSS2	1.0	3UTR
hsa-miR-6722-3p	NM_005656	TMPRSS2	1.0	3UTR
hsa-miR-892c-3p	NM_005656	TMPRSS2	1.0	3UTR
hsa-miR-6731-3p	NM_005656	TMPRSS2	1.0	3UTR
hsa-miR-6735-5p	NM_005656	TMPRSS2	1.0	3UTR
hsa-miR-6740-3p	NM_005656	TMPRSS2	1.0	3UTR
hsa-miR-6743-5p	NM_005656	TMPRSS2	1.0	3UTR
hsa-miR-6747-5p	NM_005656	TMPRSS2	1.0	3UTR
hsa-miR-6747-3p	NM_005656	TMPRSS2	1.0	3UTR
hsa-miR-6748-5p	NM_005656	TMPRSS2	1.0	3UTR
hsa-miR-6749-3p	NM_005656	TMPRSS2	1.0	3UTR
hsa-miR-6749-3p	NM_005656	TMPRSS2	1.0	3UTR
hsa-miR-6751-3p	NM_005656	TMPRSS2	1.0	3UTR
hsa-miR-6753-5p	NM_005656	TMPRSS2	1.0	3UTR
hsa-miR-6753-5p	NM_005656	TMPRSS2	1.0	3UTR
hsa-miR-6755-3p	NM_005656	TMPRSS2	1.0	3UTR
hsa-miR-6759-3p	NM_005656	TMPRSS2	1.0	3UTR
hsa-miR-6765-5p	NM_005656	TMPRSS2	1.0	3UTR
hsa-miR-6767-5p	NM_005656	TMPRSS2	1.0	3UTR
hsa-miR-6769a-5p	NM_005656	TMPRSS2	1.0	3UTR
hsa-miR-6771-5p	NM_005656	TMPRSS2	1.0	3UTR
hsa-miR-6771-5p	NM_005656	TMPRSS2	1.0	3UTR
hsa-miR-6772-5p	NM_005656	TMPRSS2	1.0	3UTR
hsa-miR-6772-5p	NM_005656	TMPRSS2	1.0	3UTR
hsa-miR-6773-3p	NM_005656	TMPRSS2	1.0	3UTR
hsa-miR-6774-5p	NM_005656	TMPRSS2	1.0	3UTR
hsa-miR-6776-3p	NM_005656	TMPRSS2	1.0	3UTR
hsa-miR-6777-5p	NM_005656	TMPRSS2	1.0	3UTR
hsa-miR-6778-5p	NM_005656	TMPRSS2	1.0	3UTR
hsa-miR-6779-5p	NM_005656	TMPRSS2	1.0	3UTR
hsa-miR-6779-5p	NM_005656	TMPRSS2	1.0	3UTR
hsa-miR-6780a-5p	NM_005656	TMPRSS2	1.0	3UTR
hsa-miR-6780a-5p	NM_005656	TMPRSS2	1.0	3UTR
hsa-miR-6782-5p	NM_005656	TMPRSS2	1.0	3UTR
hsa-miR-6792-3p	NM_005656	TMPRSS2	1.0	3UTR
hsa-miR-6793-5p	NM_005656	TMPRSS2	1.0	3UTR
hsa-miR-6794-5p	NM_005656	TMPRSS2	1.0	3UTR
hsa-miR-6795-5p	NM_005656	TMPRSS2	1.0	3UTR
hsa-miR-6795-3p	NM_005656	TMPRSS2	1.0	3UTR
hsa-miR-6797-3p	NM_005656	TMPRSS2	1.0	3UTR

hsa-miR-6798-5p	NM_005656	TMPRSS2	1.0	3UTR
hsa-miR-6799-5p	NM_005656	TMPRSS2	1.0	3UTR
hsa-miR-6802-3p	NM_005656	TMPRSS2	1.0	3UTR
hsa-miR-6804-3p	NM_005656	TMPRSS2	1.0	3UTR
hsa-miR-6805-5p	NM_005656	TMPRSS2	1.0	3UTR
hsa-miR-6807-5p	NM_005656	TMPRSS2	1.0	3UTR
hsa-miR-6807-3p	NM_005656	TMPRSS2	1.0	3UTR
hsa-miR-6808-5p	NM_005656	TMPRSS2	1.0	3UTR
hsa-miR-6810-5p	NM_005656	TMPRSS2	1.0	3UTR
hsa-miR-6813-3p	NM_005656	TMPRSS2	1.0	3UTR
hsa-miR-6814-3p	NM_005656	TMPRSS2	1.0	3UTR
hsa-miR-6816-3p	NM_005656	TMPRSS2	1.0	3UTR
hsa-miR-6818-5p	NM_005656	TMPRSS2	1.0	3UTR
hsa-miR-6819-5p	NM_005656	TMPRSS2	1.0	3UTR
hsa-miR-6819-3p	NM_005656	TMPRSS2	1.0	3UTR
hsa-miR-6822-5p	NM_005656	TMPRSS2	1.0	3UTR
hsa-miR-6823-5p	NM_005656	TMPRSS2	1.0	3UTR
hsa-miR-6824-5p	NM_005656	TMPRSS2	1.0	3UTR
hsa-miR-6824-3p	NM_005656	TMPRSS2	1.0	3UTR
hsa-miR-6828-5p	NM_005656	TMPRSS2	1.0	3UTR
hsa-miR-6829-5p	NM_005656	TMPRSS2	1.0	3UTR
hsa-miR-6832-5p	NM_005656	TMPRSS2	1.0	3UTR
hsa-miR-6835-5p	NM_005656	TMPRSS2	1.0	3UTR
hsa-miR-6837-5p	NM_005656	TMPRSS2	1.0	3UTR
hsa-miR-6841-5p	NM_005656	TMPRSS2	1.0	3UTR
hsa-miR-6842-5p	NM_005656	TMPRSS2	1.0	3UTR
hsa-miR-6842-3p	NM_005656	TMPRSS2	1.0	3UTR
hsa-miR-6843-3p	NM_005656	TMPRSS2	1.0	3UTR
hsa-miR-6845-3p	NM_005656	TMPRSS2	1.0	3UTR
hsa-miR-6846-5p	NM_005656	TMPRSS2	1.0	3UTR
hsa-miR-6849-5p	NM_005656	TMPRSS2	1.0	3UTR
hsa-miR-6852-5p	NM_005656	TMPRSS2	1.0	3UTR
hsa-miR-6854-3p	NM_005656	TMPRSS2	1.0	3UTR
hsa-miR-6769b-5p	NM_005656	TMPRSS2	1.0	3UTR
hsa-miR-6860	NM_005656	TMPRSS2	1.0	3UTR
hsa-miR-6864-5p	NM_005656	TMPRSS2	1.0	3UTR
hsa-miR-6868-5p	NM_005656	TMPRSS2	1.0	3UTR
hsa-miR-6871-5p	NM_005656	TMPRSS2	1.0	3UTR
hsa-miR-6872-5p	NM_005656	TMPRSS2	1.0	3UTR
hsa-miR-6875-3p	NM_005656	TMPRSS2	1.0	3UTR
hsa-miR-6876-3p	NM_005656	TMPRSS2	1.0	3UTR
hsa-miR-6878-3p	NM_005656	TMPRSS2	1.0	3UTR
hsa-miR-6879-5p	NM_005656	TMPRSS2	1.0	3UTR
hsa-miR-6879-5p	NM_005656	TMPRSS2	1.0	3UTR
hsa-miR-6880-3p	NM_005656	TMPRSS2	1.0	3UTR
hsa-miR-6883-5p	NM_005656	TMPRSS2	1.0	3UTR
hsa-miR-6883-3p	NM_005656	TMPRSS2	1.0	3UTR

hsa-miR-6884-5p	NM_005656	TMPRSS2	1.0	3UTR
hsa-miR-6888-5p	NM_005656	TMPRSS2	1.0	3UTR
hsa-miR-6888-5p	NM_005656	TMPRSS2	1.0	3UTR
hsa-miR-6890-5p	NM_005656	TMPRSS2	1.0	3UTR
hsa-miR-6893-5p	NM_005656	TMPRSS2	1.0	3UTR
hsa-miR-6894-5p	NM_005656	TMPRSS2	1.0	3UTR
hsa-miR-7107-5p	NM_005656	TMPRSS2	1.0	3UTR
hsa-miR-7110-3p	NM_005656	TMPRSS2	1.0	3UTR
hsa-miR-7112-5p	NM_005656	TMPRSS2	1.0	3UTR
hsa-miR-7112-3p	NM_005656	TMPRSS2	1.0	3UTR
hsa-miR-7113-5p	NM_005656	TMPRSS2	1.0	3UTR
hsa-miR-7114-3p	NM_005656	TMPRSS2	1.0	3UTR
hsa-miR-7151-3p	NM_005656	TMPRSS2	1.0	3UTR
hsa-miR-7151-3p	NM_005656	TMPRSS2	1.0	3UTR
hsa-miR-7158-5p	NM_005656	TMPRSS2	1.0	3UTR
hsa-miR-7702	NM_005656	TMPRSS2	1.0	3UTR
hsa-miR-7843-5p	NM_005656	TMPRSS2	1.0	3UTR
hsa-miR-7846-3p	NM_005656	TMPRSS2	1.0	3UTR
hsa-miR-7846-3p	NM_005656	TMPRSS2	1.0	3UTR
hsa-miR-7847-3p	NM_005656	TMPRSS2	1.0	3UTR
hsa-miR-7849-3p	NM_005656	TMPRSS2	1.0	3UTR
hsa-miR-7854-3p	NM_005656	TMPRSS2	1.0	3UTR
hsa-miR-8052	NM_005656	TMPRSS2	1.0	3UTR
hsa-miR-8063	NM_005656	TMPRSS2	1.0	3UTR
hsa-miR-8069	NM_005656	TMPRSS2	1.0	3UTR
hsa-miR-8072	NM_005656	TMPRSS2	1.0	3UTR
hsa-miR-8078	NM_005656	TMPRSS2	1.0	3UTR
hsa-miR-8088	NM_005656	TMPRSS2	1.0	3UTR
hsa-miR-9985	NM_005656	TMPRSS2	1.0	3UTR
hsa-miR-10394-3p	NM_005656	TMPRSS2	1.0	3UTR
hsa-miR-10397-5p	NM_005656	TMPRSS2	1.0	3UTR
hsa-miR-10398-5p	NM_005656	TMPRSS2	1.0	3UTR
hsa-miR-10526-3p	NM_005656	TMPRSS2	1.0	3UTR
hsa-miR-11181-3p	NM_005656	TMPRSS2	1.0	3UTR
hsa-miR-3059-3p	NM_005656	TMPRSS2	1.0	3UTR
hsa-miR-3059-3p	NM_005656	TMPRSS2	1.0	3UTR
hsa-miR-3085-5p	NM_005656	TMPRSS2	1.0	3UTR
hsa-miR-3085-3p	NM_005656	TMPRSS2	1.0	3UTR
hsa-miR-6529-5p	NM_005656	TMPRSS2	1.0	3UTR
hsa-miR-9851-5p	NM_005656	TMPRSS2	1.0	3UTR
hsa-miR-9851-3p	NM_005656	TMPRSS2	1.0	3UTR
hsa-miR-12119	NM_005656	TMPRSS2	1.0	3UTR
hsa-miR-12119	NM_005656	TMPRSS2	1.0	3UTR
hsa-miR-12120	NM_005656	TMPRSS2	1.0	3UTR
hsa-miR-12128	NM_005656	TMPRSS2	1.0	3UTR
hsa-miR-12131	NM_005656	TMPRSS2	1.0	3UTR
hsa-let-7b-5p	NM_001135099	TMPRSS2	1.0	3UTR

hsa-let-7c-5p	NM_001135099	TMPRSS2	1.0	3UTR
hsa-miR-23a-5p	NM_001135099	TMPRSS2	1.0	3UTR
hsa-miR-26a-5p	NM_001135099	TMPRSS2	1.0	3UTR
hsa-miR-26b-3p	NM_001135099	TMPRSS2	1.0	3UTR
hsa-miR-28-5p	NM_001135099	TMPRSS2	1.0	3UTR
hsa-miR-92a-1-5p	NM_001135099	TMPRSS2	1.0	3UTR
hsa-miR-93-5p	NM_001135099	TMPRSS2	1.0	3UTR
hsa-miR-95-3p	NM_001135099	TMPRSS2	1.0	3UTR
hsa-miR-96-5p	NM_001135099	TMPRSS2	1.0	3UTR
hsa-miR-101-5p	NM_001135099	TMPRSS2	1.0	3UTR
hsa-miR-103a-2-5p	NM_001135099	TMPRSS2	1.0	3UTR
hsa-miR-103a-3p	NM_001135099	TMPRSS2	1.0	3UTR
hsa-miR-103a-1-5p	NM_001135099	TMPRSS2	1.0	3UTR
hsa-miR-107	NM_001135099	TMPRSS2	1.0	3UTR
hsa-miR-197-3p	NM_001135099	TMPRSS2	1.0	3UTR
hsa-miR-198	NM_001135099	TMPRSS2	1.0	3UTR
hsa-miR-129-1-3p	NM_001135099	TMPRSS2	1.0	3UTR
hsa-miR-30d-5p	NM_001135099	TMPRSS2	1.0	3UTR
hsa-miR-34a-5p	NM_001135099	TMPRSS2	1.0	3UTR
hsa-miR-182-5p	NM_001135099	TMPRSS2	1.0	3UTR
hsa-miR-183-5p	NM_001135099	TMPRSS2	1.0	3UTR
hsa-miR-204-3p	NM_001135099	TMPRSS2	1.0	3UTR
hsa-miR-218-1-3p	NM_001135099	TMPRSS2	1.0	3UTR
hsa-miR-200b-5p	NM_001135099	TMPRSS2	1.0	3UTR
hsa-let-7g-5p	NM_001135099	TMPRSS2	1.0	3UTR
hsa-let-7i-5p	NM_001135099	TMPRSS2	1.0	3UTR
hsa-miR-27b-5p	NM_001135099	TMPRSS2	1.0	3UTR
hsa-miR-30b-3p	NM_001135099	TMPRSS2	1.0	3UTR
hsa-miR-124-5p	NM_001135099	TMPRSS2	1.0	3UTR
hsa-miR-135a-2-3p	NM_001135099	TMPRSS2	1.0	3UTR
hsa-miR-152-3p	NM_001135099	TMPRSS2	1.0	3UTR
hsa-miR-191-5p	NM_001135099	TMPRSS2	1.0	3UTR
hsa-miR-9-5p	NM_001135099	TMPRSS2	1.0	3UTR
hsa-miR-134-5p	NM_001135099	TMPRSS2	1.0	3UTR
hsa-miR-149-5p	NM_001135099	TMPRSS2	1.0	3UTR
hsa-miR-185-5p	NM_001135099	TMPRSS2	1.0	3UTR
hsa-miR-188-5p	NM_001135099	TMPRSS2	1.0	3UTR
hsa-miR-193a-5p	NM_001135099	TMPRSS2	1.0	3UTR
hsa-miR-320a-3p	NM_001135099	TMPRSS2	1.0	3UTR
hsa-miR-200c-5p	NM_001135099	TMPRSS2	1.0	3UTR
hsa-miR-106b-3p	NM_001135099	TMPRSS2	1.0	3UTR
hsa-miR-361-5p	NM_001135099	TMPRSS2	1.0	3UTR
hsa-miR-302c-5p	NM_001135099	TMPRSS2	1.0	3UTR
hsa-miR-379-5p	NM_001135099	TMPRSS2	1.0	3UTR
hsa-miR-328-5p	NM_001135099	TMPRSS2	1.0	3UTR
hsa-miR-151a-5p	NM_001135099	TMPRSS2	1.0	3UTR
hsa-miR-151a-3p	NM_001135099	TMPRSS2	1.0	3UTR

hsa-miR-331-5p	NM_001135099	TMPRSS2	1.0	3UTR
hsa-miR-331-3p	NM_001135099	TMPRSS2	1.0	3UTR
hsa-miR-324-5p	NM_001135099	TMPRSS2	1.0	3UTR
hsa-miR-324-3p	NM_001135099	TMPRSS2	1.0	3UTR
hsa-miR-339-5p	NM_001135099	TMPRSS2	1.0	3UTR
hsa-miR-422a	NM_001135099	TMPRSS2	1.0	3UTR
hsa-miR-423-5p	NM_001135099	TMPRSS2	1.0	3UTR
hsa-miR-423-5p	NM_001135099	TMPRSS2	1.0	3UTR
hsa-miR-423-3p	NM_001135099	TMPRSS2	1.0	3UTR
hsa-miR-20b-3p	NM_001135099	TMPRSS2	1.0	3UTR
hsa-miR-329-5p	NM_001135099	TMPRSS2	1.0	3UTR
hsa-miR-483-3p	NM_001135099	TMPRSS2	1.0	3UTR
hsa-miR-491-5p	NM_001135099	TMPRSS2	1.0	3UTR
hsa-miR-146b-3p	NM_001135099	TMPRSS2	1.0	3UTR
hsa-miR-432-5p	NM_001135099	TMPRSS2	1.0	3UTR
hsa-miR-432-5p	NM_001135099	TMPRSS2	1.0	3UTR
hsa-miR-193b-5p	NM_001135099	TMPRSS2	1.0	3UTR
hsa-miR-181d-5p	NM_001135099	TMPRSS2	1.0	3UTR
hsa-miR-526b-5p	NM_001135099	TMPRSS2	1.0	3UTR
hsa-miR-520b-5p	NM_001135099	TMPRSS2	1.0	3UTR
hsa-miR-520c-3p	NM_001135099	TMPRSS2	1.0	3UTR
hsa-miR-519a-2-5p	NM_001135099	TMPRSS2	1.0	3UTR
hsa-miR-501-5p	NM_001135099	TMPRSS2	1.0	3UTR
hsa-miR-502-5p	NM_001135099	TMPRSS2	1.0	3UTR
hsa-miR-502-3p	NM_001135099	TMPRSS2	1.0	3UTR
hsa-miR-503-3p	NM_001135099	TMPRSS2	1.0	3UTR
hsa-miR-504-3p	NM_001135099	TMPRSS2	1.0	3UTR
hsa-miR-505-5p	NM_001135099	TMPRSS2	1.0	3UTR
hsa-miR-505-5p	NM_001135099	TMPRSS2	1.0	3UTR
hsa-miR-505-3p	NM_001135099	TMPRSS2	1.0	3UTR
hsa-miR-507	NM_001135099	TMPRSS2	1.0	3UTR
hsa-miR-508-5p	NM_001135099	TMPRSS2	1.0	3UTR
hsa-miR-509-3p	NM_001135099	TMPRSS2	1.0	3UTR
hsa-miR-514a-5p	NM_001135099	TMPRSS2	1.0	3UTR
hsa-miR-455-5p	NM_001135099	TMPRSS2	1.0	3UTR
hsa-miR-455-3p	NM_001135099	TMPRSS2	1.0	3UTR
hsa-miR-562	NM_001135099	TMPRSS2	1.0	3UTR
hsa-miR-575	NM_001135099	TMPRSS2	1.0	3UTR
hsa-miR-584-3p	NM_001135099	TMPRSS2	1.0	3UTR
hsa-miR-548b-3p	NM_001135099	TMPRSS2	1.0	3UTR
hsa-miR-589-5p	NM_001135099	TMPRSS2	1.0	3UTR
hsa-miR-550a-5p	NM_001135099	TMPRSS2	1.0	3UTR
hsa-miR-550a-5p	NM_001135099	TMPRSS2	1.0	3UTR
hsa-miR-602	NM_001135099	TMPRSS2	1.0	3UTR
hsa-miR-608	NM_001135099	TMPRSS2	1.0	3UTR
hsa-miR-613	NM_001135099	TMPRSS2	1.0	3UTR
hsa-miR-614	NM_001135099	TMPRSS2	1.0	3UTR

hsa-miR-619-5p	NM_001135099	TMPRSS2	1.0	3UTR
hsa-miR-622	NM_001135099	TMPRSS2	1.0	3UTR
hsa-miR-622	NM_001135099	TMPRSS2	1.0	3UTR
hsa-miR-33b-3p	NM_001135099	TMPRSS2	1.0	3UTR
hsa-miR-642a-5p	NM_001135099	TMPRSS2	1.0	3UTR
hsa-miR-647	NM_001135099	TMPRSS2	1.0	3UTR
hsa-miR-648	NM_001135099	TMPRSS2	1.0	3UTR
hsa-miR-650	NM_001135099	TMPRSS2	1.0	3UTR
hsa-miR-449b-5p	NM_001135099	TMPRSS2	1.0	3UTR
hsa-miR-671-5p	NM_001135099	TMPRSS2	1.0	3UTR
hsa-miR-671-3p	NM_001135099	TMPRSS2	1.0	3UTR
hsa-miR-550a-3-5p	NM_001135099	TMPRSS2	1.0	3UTR
hsa-miR-767-5p	NM_001135099	TMPRSS2	1.0	3UTR
hsa-miR-767-5p	NM_001135099	TMPRSS2	1.0	3UTR
hsa-miR-1224-3p	NM_001135099	TMPRSS2	1.0	3UTR
hsa-miR-320b	NM_001135099	TMPRSS2	1.0	3UTR
hsa-miR-1271-5p	NM_001135099	TMPRSS2	1.0	3UTR
hsa-miR-1271-3p	NM_001135099	TMPRSS2	1.0	3UTR
hsa-miR-449c-3p	NM_001135099	TMPRSS2	1.0	3UTR
hsa-miR-769-5p	NM_001135099	TMPRSS2	1.0	3UTR
hsa-miR-766-5p	NM_001135099	TMPRSS2	1.0	3UTR
hsa-miR-675-5p	NM_001135099	TMPRSS2	1.0	3UTR
hsa-miR-890	NM_001135099	TMPRSS2	1.0	3UTR
hsa-miR-875-3p	NM_001135099	TMPRSS2	1.0	3UTR
hsa-miR-708-5p	NM_001135099	TMPRSS2	1.0	3UTR
hsa-miR-665	NM_001135099	TMPRSS2	1.0	3UTR
hsa-miR-921	NM_001135099	TMPRSS2	1.0	3UTR
hsa-miR-933	NM_001135099	TMPRSS2	1.0	3UTR
hsa-miR-936	NM_001135099	TMPRSS2	1.0	3UTR
hsa-miR-939-5p	NM_001135099	TMPRSS2	1.0	3UTR
hsa-miR-1182	NM_001135099	TMPRSS2	1.0	3UTR
hsa-miR-1227-3p	NM_001135099	TMPRSS2	1.0	3UTR
hsa-miR-1229-5p	NM_001135099	TMPRSS2	1.0	3UTR
hsa-miR-1229-3p	NM_001135099	TMPRSS2	1.0	3UTR
hsa-miR-1234-3p	NM_001135099	TMPRSS2	1.0	3UTR
hsa-miR-1234-3p	NM_001135099	TMPRSS2	1.0	3UTR
hsa-miR-1236-5p	NM_001135099	TMPRSS2	1.0	3UTR
hsa-miR-1237-3p	NM_001135099	TMPRSS2	1.0	3UTR
hsa-miR-1238-3p	NM_001135099	TMPRSS2	1.0	3UTR
hsa-miR-1207-3p	NM_001135099	TMPRSS2	1.0	3UTR
hsa-miR-1243	NM_001135099	TMPRSS2	1.0	3UTR
hsa-miR-1250-5p	NM_001135099	TMPRSS2	1.0	3UTR
hsa-miR-1253	NM_001135099	TMPRSS2	1.0	3UTR
hsa-miR-1265	NM_001135099	TMPRSS2	1.0	3UTR
hsa-miR-1266-5p	NM_001135099	TMPRSS2	1.0	3UTR
hsa-miR-1275	NM_001135099	TMPRSS2	1.0	3UTR
hsa-miR-1255b-2-3p	NM_001135099	TMPRSS2	1.0	3UTR

hsa-miR-1321	NM_001135099	TMPRSS2	1.0	3UTR
hsa-miR-1324	NM_001135099	TMPRSS2	1.0	3UTR
hsa-miR-103b	NM_001135099	TMPRSS2	1.0	3UTR
hsa-miR-1825	NM_001135099	TMPRSS2	1.0	3UTR
hsa-miR-1908-5p	NM_001135099	TMPRSS2	1.0	3UTR
hsa-miR-1909-5p	NM_001135099	TMPRSS2	1.0	3UTR
hsa-miR-1910-3p	NM_001135099	TMPRSS2	1.0	3UTR
hsa-miR-1912-3p	NM_001135099	TMPRSS2	1.0	3UTR
hsa-miR-2116-5p	NM_001135099	TMPRSS2	1.0	3UTR
hsa-miR-2277-5p	NM_001135099	TMPRSS2	1.0	3UTR
hsa-miR-2682-3p	NM_001135099	TMPRSS2	1.0	3UTR
hsa-miR-3126-5p	NM_001135099	TMPRSS2	1.0	3UTR
hsa-miR-3129-5p	NM_001135099	TMPRSS2	1.0	3UTR
hsa-miR-3131	NM_001135099	TMPRSS2	1.0	3UTR
hsa-miR-3137	NM_001135099	TMPRSS2	1.0	3UTR
hsa-miR-3138	NM_001135099	TMPRSS2	1.0	3UTR
hsa-miR-3144-5p	NM_001135099	TMPRSS2	1.0	3UTR
hsa-miR-3153	NM_001135099	TMPRSS2	1.0	3UTR
hsa-miR-3158-5p	NM_001135099	TMPRSS2	1.0	3UTR
hsa-miR-3158-3p	NM_001135099	TMPRSS2	1.0	3UTR
hsa-miR-3166	NM_001135099	TMPRSS2	1.0	3UTR
hsa-miR-1260b	NM_001135099	TMPRSS2	1.0	3UTR
hsa-miR-3170	NM_001135099	TMPRSS2	1.0	3UTR
hsa-miR-3179	NM_001135099	TMPRSS2	1.0	3UTR
hsa-miR-3184-3p	NM_001135099	TMPRSS2	1.0	3UTR
hsa-miR-3187-3p	NM_001135099	TMPRSS2	1.0	3UTR
hsa-miR-3190-3p	NM_001135099	TMPRSS2	1.0	3UTR
hsa-miR-3192-5p	NM_001135099	TMPRSS2	1.0	3UTR
hsa-miR-3193	NM_001135099	TMPRSS2	1.0	3UTR
hsa-miR-3194-5p	NM_001135099	TMPRSS2	1.0	3UTR
hsa-miR-4300	NM_001135099	TMPRSS2	1.0	3UTR
hsa-miR-4314	NM_001135099	TMPRSS2	1.0	3UTR
hsa-miR-4318	NM_001135099	TMPRSS2	1.0	3UTR
hsa-miR-4259	NM_001135099	TMPRSS2	1.0	3UTR
hsa-miR-4260	NM_001135099	TMPRSS2	1.0	3UTR
hsa-miR-4327	NM_001135099	TMPRSS2	1.0	3UTR
hsa-miR-4327	NM_001135099	TMPRSS2	1.0	3UTR
hsa-miR-4269	NM_001135099	TMPRSS2	1.0	3UTR
hsa-miR-4283	NM_001135099	TMPRSS2	1.0	3UTR
hsa-miR-3619-5p	NM_001135099	TMPRSS2	1.0	3UTR
hsa-miR-3620-5p	NM_001135099	TMPRSS2	1.0	3UTR
hsa-miR-3652	NM_001135099	TMPRSS2	1.0	3UTR
hsa-miR-3655	NM_001135099	TMPRSS2	1.0	3UTR
hsa-miR-3664-3p	NM_001135099	TMPRSS2	1.0	3UTR
hsa-miR-3665	NM_001135099	TMPRSS2	1.0	3UTR
hsa-miR-3667-3p	NM_001135099	TMPRSS2	1.0	3UTR
hsa-miR-3679-5p	NM_001135099	TMPRSS2	1.0	3UTR

hsa-miR-3682-3p	NM_001135099	TMPRSS2	1.0	3UTR
hsa-miR-3907	NM_001135099	TMPRSS2	1.0	3UTR
hsa-miR-3911	NM_001135099	TMPRSS2	1.0	3UTR
hsa-miR-3913-3p	NM_001135099	TMPRSS2	1.0	3UTR
hsa-miR-3926	NM_001135099	TMPRSS2	1.0	3UTR
hsa-miR-3928-3p	NM_001135099	TMPRSS2	1.0	3UTR
hsa-miR-3936	NM_001135099	TMPRSS2	1.0	3UTR
hsa-miR-3939	NM_001135099	TMPRSS2	1.0	3UTR
hsa-miR-3940-5p	NM_001135099	TMPRSS2	1.0	3UTR
hsa-miR-3944-5p	NM_001135099	TMPRSS2	1.0	3UTR
hsa-miR-642b-3p	NM_001135099	TMPRSS2	1.0	3UTR
hsa-miR-550b-2-5p	NM_001135099	TMPRSS2	1.0	3UTR
hsa-miR-4418	NM_001135099	TMPRSS2	1.0	3UTR
hsa-miR-378f	NM_001135099	TMPRSS2	1.0	3UTR
hsa-miR-4425	NM_001135099	TMPRSS2	1.0	3UTR
hsa-miR-4428	NM_001135099	TMPRSS2	1.0	3UTR
hsa-miR-4429	NM_001135099	TMPRSS2	1.0	3UTR
hsa-miR-4433a-3p	NM_001135099	TMPRSS2	1.0	3UTR
hsa-miR-4436a	NM_001135099	TMPRSS2	1.0	3UTR
hsa-miR-4439	NM_001135099	TMPRSS2	1.0	3UTR
hsa-miR-4441	NM_001135099	TMPRSS2	1.0	3UTR
hsa-miR-4446-3p	NM_001135099	TMPRSS2	1.0	3UTR
hsa-miR-4449	NM_001135099	TMPRSS2	1.0	3UTR
hsa-miR-4456	NM_001135099	TMPRSS2	1.0	3UTR
hsa-miR-4458	NM_001135099	TMPRSS2	1.0	3UTR
hsa-miR-4469	NM_001135099	TMPRSS2	1.0	3UTR
hsa-miR-4481	NM_001135099	TMPRSS2	1.0	3UTR
hsa-miR-4484	NM_001135099	TMPRSS2	1.0	3UTR
hsa-miR-4489	NM_001135099	TMPRSS2	1.0	3UTR
hsa-miR-4501	NM_001135099	TMPRSS2	1.0	3UTR
hsa-miR-4505	NM_001135099	TMPRSS2	1.0	3UTR
hsa-miR-4516	NM_001135099	TMPRSS2	1.0	3UTR
hsa-miR-4519	NM_001135099	TMPRSS2	1.0	3UTR
hsa-miR-4533	NM_001135099	TMPRSS2	1.0	3UTR
hsa-miR-378i	NM_001135099	TMPRSS2	1.0	3UTR
hsa-miR-3978	NM_001135099	TMPRSS2	1.0	3UTR
hsa-miR-4638-3p	NM_001135099	TMPRSS2	1.0	3UTR
hsa-miR-4646-5p	NM_001135099	TMPRSS2	1.0	3UTR
hsa-miR-4653-3p	NM_001135099	TMPRSS2	1.0	3UTR
hsa-miR-4653-3p	NM_001135099	TMPRSS2	1.0	3UTR
hsa-miR-4654	NM_001135099	TMPRSS2	1.0	3UTR
hsa-miR-4655-5p	NM_001135099	TMPRSS2	1.0	3UTR
hsa-miR-4667-5p	NM_001135099	TMPRSS2	1.0	3UTR
hsa-miR-4685-3p	NM_001135099	TMPRSS2	1.0	3UTR
hsa-miR-4688	NM_001135099	TMPRSS2	1.0	3UTR
hsa-miR-4698	NM_001135099	TMPRSS2	1.0	3UTR
hsa-miR-4700-5p	NM_001135099	TMPRSS2	1.0	3UTR

hsa-miR-4716-3p	NM_001135099	TMPRSS2	1.0	3UTR
hsa-miR-4716-3p	NM_001135099	TMPRSS2	1.0	3UTR
hsa-miR-4717-5p	NM_001135099	TMPRSS2	1.0	3UTR
hsa-miR-4722-5p	NM_001135099	TMPRSS2	1.0	3UTR
hsa-miR-4723-5p	NM_001135099	TMPRSS2	1.0	3UTR
hsa-miR-4725-5p	NM_001135099	TMPRSS2	1.0	3UTR
hsa-miR-4739	NM_001135099	TMPRSS2	1.0	3UTR
hsa-miR-4741	NM_001135099	TMPRSS2	1.0	3UTR
hsa-miR-4746-3p	NM_001135099	TMPRSS2	1.0	3UTR
hsa-miR-4749-3p	NM_001135099	TMPRSS2	1.0	3UTR
hsa-miR-4750-5p	NM_001135099	TMPRSS2	1.0	3UTR
hsa-miR-4760-3p	NM_001135099	TMPRSS2	1.0	3UTR
hsa-miR-4761-3p	NM_001135099	TMPRSS2	1.0	3UTR
hsa-miR-4764-3p	NM_001135099	TMPRSS2	1.0	3UTR
hsa-miR-4776-5p	NM_001135099	TMPRSS2	1.0	3UTR
hsa-miR-4436b-3p	NM_001135099	TMPRSS2	1.0	3UTR
hsa-miR-4436b-3p	NM_001135099	TMPRSS2	1.0	3UTR
hsa-miR-2467-3p	NM_001135099	TMPRSS2	1.0	3UTR
hsa-miR-4799-3p	NM_001135099	TMPRSS2	1.0	3UTR
hsa-miR-5001-3p	NM_001135099	TMPRSS2	1.0	3UTR
hsa-miR-5002-3p	NM_001135099	TMPRSS2	1.0	3UTR
hsa-miR-5003-3p	NM_001135099	TMPRSS2	1.0	3UTR
hsa-miR-5004-3p	NM_001135099	TMPRSS2	1.0	3UTR
hsa-miR-548ao-3p	NM_001135099	TMPRSS2	1.0	3UTR
hsa-miR-5006-5p	NM_001135099	TMPRSS2	1.0	3UTR
hsa-miR-5006-5p	NM_001135099	TMPRSS2	1.0	3UTR
hsa-miR-5010-5p	NM_001135099	TMPRSS2	1.0	3UTR
hsa-miR-5088-5p	NM_001135099	TMPRSS2	1.0	3UTR
hsa-miR-5093	NM_001135099	TMPRSS2	1.0	3UTR
hsa-miR-5190	NM_001135099	TMPRSS2	1.0	3UTR
hsa-miR-5192	NM_001135099	TMPRSS2	1.0	3UTR
hsa-miR-5195-3p	NM_001135099	TMPRSS2	1.0	3UTR
hsa-miR-5196-5p	NM_001135099	TMPRSS2	1.0	3UTR
hsa-miR-4524b-3p	NM_001135099	TMPRSS2	1.0	3UTR
hsa-miR-5571-3p	NM_001135099	TMPRSS2	1.0	3UTR
hsa-miR-5572	NM_001135099	TMPRSS2	1.0	3UTR
hsa-miR-5581-5p	NM_001135099	TMPRSS2	1.0	3UTR
hsa-miR-548au-3p	NM_001135099	TMPRSS2	1.0	3UTR
hsa-miR-1295b-3p	NM_001135099	TMPRSS2	1.0	3UTR
hsa-miR-1295b-3p	NM_001135099	TMPRSS2	1.0	3UTR
hsa-miR-5589-3p	NM_001135099	TMPRSS2	1.0	3UTR
hsa-miR-5682	NM_001135099	TMPRSS2	1.0	3UTR
hsa-miR-5691	NM_001135099	TMPRSS2	1.0	3UTR
hsa-miR-5698	NM_001135099	TMPRSS2	1.0	3UTR
hsa-miR-5704	NM_001135099	TMPRSS2	1.0	3UTR
hsa-miR-1199-3p	NM_001135099	TMPRSS2	1.0	3UTR
hsa-miR-6068	NM_001135099	TMPRSS2	1.0	3UTR

hsa-miR-6070	NM_001135099	TMPRSS2	1.0	3UTR
hsa-miR-6074	NM_001135099	TMPRSS2	1.0	3UTR
hsa-miR-6077	NM_001135099	TMPRSS2	1.0	3UTR
hsa-miR-6086	NM_001135099	TMPRSS2	1.0	3UTR
hsa-miR-6088	NM_001135099	TMPRSS2	1.0	3UTR
hsa-miR-6090	NM_001135099	TMPRSS2	1.0	3UTR
hsa-miR-6133	NM_001135099	TMPRSS2	1.0	3UTR
hsa-miR-6134	NM_001135099	TMPRSS2	1.0	3UTR
hsa-miR-6165	NM_001135099	TMPRSS2	1.0	3UTR
hsa-miR-548ay-3p	NM_001135099	TMPRSS2	1.0	3UTR
hsa-miR-6500-5p	NM_001135099	TMPRSS2	1.0	3UTR
hsa-miR-6505-5p	NM_001135099	TMPRSS2	1.0	3UTR
hsa-miR-6510-5p	NM_001135099	TMPRSS2	1.0	3UTR
hsa-miR-6515-5p	NM_001135099	TMPRSS2	1.0	3UTR
hsa-miR-6515-5p	NM_001135099	TMPRSS2	1.0	3UTR
hsa-miR-6717-5p	NM_001135099	TMPRSS2	1.0	3UTR
hsa-miR-6511b-3p	NM_001135099	TMPRSS2	1.0	3UTR
hsa-miR-6720-3p	NM_001135099	TMPRSS2	1.0	3UTR
hsa-miR-892c-3p	NM_001135099	TMPRSS2	1.0	3UTR
hsa-miR-6727-5p	NM_001135099	TMPRSS2	1.0	3UTR
hsa-miR-6728-3p	NM_001135099	TMPRSS2	1.0	3UTR
hsa-miR-6729-5p	NM_001135099	TMPRSS2	1.0	3UTR
hsa-miR-6731-5p	NM_001135099	TMPRSS2	1.0	3UTR
hsa-miR-6734-5p	NM_001135099	TMPRSS2	1.0	3UTR
hsa-miR-6735-5p	NM_001135099	TMPRSS2	1.0	3UTR
hsa-miR-6735-3p	NM_001135099	TMPRSS2	1.0	3UTR
hsa-miR-6736-3p	NM_001135099	TMPRSS2	1.0	3UTR
hsa-miR-6737-5p	NM_001135099	TMPRSS2	1.0	3UTR
hsa-miR-6737-3p	NM_001135099	TMPRSS2	1.0	3UTR
hsa-miR-6740-5p	NM_001135099	TMPRSS2	1.0	3UTR
hsa-miR-6740-3p	NM_001135099	TMPRSS2	1.0	3UTR
hsa-miR-6748-5p	NM_001135099	TMPRSS2	1.0	3UTR
hsa-miR-6751-3p	NM_001135099	TMPRSS2	1.0	3UTR
hsa-miR-6754-3p	NM_001135099	TMPRSS2	1.0	3UTR
hsa-miR-6755-3p	NM_001135099	TMPRSS2	1.0	3UTR
hsa-miR-6757-3p	NM_001135099	TMPRSS2	1.0	3UTR
hsa-miR-6759-3p	NM_001135099	TMPRSS2	1.0	3UTR
hsa-miR-6761-3p	NM_001135099	TMPRSS2	1.0	3UTR
hsa-miR-6767-5p	NM_001135099	TMPRSS2	1.0	3UTR
hsa-miR-6769a-5p	NM_001135099	TMPRSS2	1.0	3UTR
hsa-miR-6771-5p	NM_001135099	TMPRSS2	1.0	3UTR
hsa-miR-6772-5p	NM_001135099	TMPRSS2	1.0	3UTR
hsa-miR-6772-3p	NM_001135099	TMPRSS2	1.0	3UTR
hsa-miR-6773-3p	NM_001135099	TMPRSS2	1.0	3UTR
hsa-miR-6774-5p	NM_001135099	TMPRSS2	1.0	3UTR
hsa-miR-6774-5p	NM_001135099	TMPRSS2	1.0	3UTR
hsa-miR-6775-3p	NM_001135099	TMPRSS2	1.0	3UTR

hsa-miR-6777-3p	NM_001135099	TMPRSS2	1.0	3UTR
hsa-miR-6778-5p	NM_001135099	TMPRSS2	1.0	3UTR
hsa-miR-6780a-5p	NM_001135099	TMPRSS2	1.0	3UTR
hsa-miR-6780a-5p	NM_001135099	TMPRSS2	1.0	3UTR
hsa-miR-6787-5p	NM_001135099	TMPRSS2	1.0	3UTR
hsa-miR-6793-5p	NM_001135099	TMPRSS2	1.0	3UTR
hsa-miR-6794-5p	NM_001135099	TMPRSS2	1.0	3UTR
hsa-miR-6795-3p	NM_001135099	TMPRSS2	1.0	3UTR
hsa-miR-6799-3p	NM_001135099	TMPRSS2	1.0	3UTR
hsa-miR-6800-3p	NM_001135099	TMPRSS2	1.0	3UTR
hsa-miR-6802-3p	NM_001135099	TMPRSS2	1.0	3UTR
hsa-miR-6804-3p	NM_001135099	TMPRSS2	1.0	3UTR
hsa-miR-6805-5p	NM_001135099	TMPRSS2	1.0	3UTR
hsa-miR-6810-5p	NM_001135099	TMPRSS2	1.0	3UTR
hsa-miR-6813-5p	NM_001135099	TMPRSS2	1.0	3UTR
hsa-miR-6813-3p	NM_001135099	TMPRSS2	1.0	3UTR
hsa-miR-6815-3p	NM_001135099	TMPRSS2	1.0	3UTR
hsa-miR-6816-3p	NM_001135099	TMPRSS2	1.0	3UTR
hsa-miR-6818-5p	NM_001135099	TMPRSS2	1.0	3UTR
hsa-miR-6819-3p	NM_001135099	TMPRSS2	1.0	3UTR
hsa-miR-6823-5p	NM_001135099	TMPRSS2	1.0	3UTR
hsa-miR-6823-3p	NM_001135099	TMPRSS2	1.0	3UTR
hsa-miR-6824-5p	NM_001135099	TMPRSS2	1.0	3UTR
hsa-miR-6824-3p	NM_001135099	TMPRSS2	1.0	3UTR
hsa-miR-6825-3p	NM_001135099	TMPRSS2	1.0	3UTR
hsa-miR-6828-5p	NM_001135099	TMPRSS2	1.0	3UTR
hsa-miR-6829-5p	NM_001135099	TMPRSS2	1.0	3UTR
hsa-miR-6833-3p	NM_001135099	TMPRSS2	1.0	3UTR
hsa-miR-6834-5p	NM_001135099	TMPRSS2	1.0	3UTR
hsa-miR-6835-5p	NM_001135099	TMPRSS2	1.0	3UTR
hsa-miR-6780b-3p	NM_001135099	TMPRSS2	1.0	3UTR
hsa-miR-6837-5p	NM_001135099	TMPRSS2	1.0	3UTR
hsa-miR-6841-5p	NM_001135099	TMPRSS2	1.0	3UTR
hsa-miR-6842-5p	NM_001135099	TMPRSS2	1.0	3UTR
hsa-miR-6842-3p	NM_001135099	TMPRSS2	1.0	3UTR
hsa-miR-6843-3p	NM_001135099	TMPRSS2	1.0	3UTR
hsa-miR-6845-3p	NM_001135099	TMPRSS2	1.0	3UTR
hsa-miR-6848-5p	NM_001135099	TMPRSS2	1.0	3UTR
hsa-miR-6852-5p	NM_001135099	TMPRSS2	1.0	3UTR
hsa-miR-6852-5p	NM_001135099	TMPRSS2	1.0	3UTR
hsa-miR-6854-3p	NM_001135099	TMPRSS2	1.0	3UTR
hsa-miR-6859-5p	NM_001135099	TMPRSS2	1.0	3UTR
hsa-miR-6859-3p	NM_001135099	TMPRSS2	1.0	3UTR
hsa-miR-6769b-5p	NM_001135099	TMPRSS2	1.0	3UTR
hsa-miR-6769b-3p	NM_001135099	TMPRSS2	1.0	3UTR
hsa-miR-6860	NM_001135099	TMPRSS2	1.0	3UTR
hsa-miR-6860	NM_001135099	TMPRSS2	1.0	3UTR

hsa-miR-6861-5p	NM_001135099	TMPRSS2	1.0	3UTR
hsa-miR-6864-5p	NM_001135099	TMPRSS2	1.0	3UTR
hsa-miR-6865-5p	NM_001135099	TMPRSS2	1.0	3UTR
hsa-miR-6871-5p	NM_001135099	TMPRSS2	1.0	3UTR
hsa-miR-6872-5p	NM_001135099	TMPRSS2	1.0	3UTR
hsa-miR-6875-3p	NM_001135099	TMPRSS2	1.0	3UTR
hsa-miR-6876-3p	NM_001135099	TMPRSS2	1.0	3UTR
hsa-miR-6876-3p	NM_001135099	TMPRSS2	1.0	3UTR
hsa-miR-6877-3p	NM_001135099	TMPRSS2	1.0	3UTR
hsa-miR-6878-3p	NM_001135099	TMPRSS2	1.0	3UTR
hsa-miR-6879-5p	NM_001135099	TMPRSS2	1.0	3UTR
hsa-miR-6880-3p	NM_001135099	TMPRSS2	1.0	3UTR
hsa-miR-6883-5p	NM_001135099	TMPRSS2	1.0	3UTR
hsa-miR-6888-5p	NM_001135099	TMPRSS2	1.0	3UTR
hsa-miR-6889-3p	NM_001135099	TMPRSS2	1.0	3UTR
hsa-miR-6891-5p	NM_001135099	TMPRSS2	1.0	3UTR
hsa-miR-6894-3p	NM_001135099	TMPRSS2	1.0	3UTR
hsa-miR-7107-5p	NM_001135099	TMPRSS2	1.0	3UTR
hsa-miR-7108-5p	NM_001135099	TMPRSS2	1.0	3UTR
hsa-miR-7108-3p	NM_001135099	TMPRSS2	1.0	3UTR
hsa-miR-7110-3p	NM_001135099	TMPRSS2	1.0	3UTR
hsa-miR-7111-3p	NM_001135099	TMPRSS2	1.0	3UTR
hsa-miR-7112-5p	NM_001135099	TMPRSS2	1.0	3UTR
hsa-miR-7112-3p	NM_001135099	TMPRSS2	1.0	3UTR
hsa-miR-7112-3p	NM_001135099	TMPRSS2	1.0	3UTR
hsa-miR-7113-5p	NM_001135099	TMPRSS2	1.0	3UTR
hsa-miR-7151-3p	NM_001135099	TMPRSS2	1.0	3UTR
hsa-miR-7152-5p	NM_001135099	TMPRSS2	1.0	3UTR
hsa-miR-7158-5p	NM_001135099	TMPRSS2	1.0	3UTR
hsa-miR-7702	NM_001135099	TMPRSS2	1.0	3UTR
hsa-miR-7703	NM_001135099	TMPRSS2	1.0	3UTR
hsa-miR-7843-5p	NM_001135099	TMPRSS2	1.0	3UTR
hsa-miR-7845-5p	NM_001135099	TMPRSS2	1.0	3UTR
hsa-miR-7846-3p	NM_001135099	TMPRSS2	1.0	3UTR
hsa-miR-7846-3p	NM_001135099	TMPRSS2	1.0	3UTR
hsa-miR-7847-3p	NM_001135099	TMPRSS2	1.0	3UTR
hsa-miR-7851-3p	NM_001135099	TMPRSS2	1.0	3UTR
hsa-miR-8052	NM_001135099	TMPRSS2	1.0	3UTR
hsa-miR-8063	NM_001135099	TMPRSS2	1.0	3UTR
hsa-miR-8072	NM_001135099	TMPRSS2	1.0	3UTR
hsa-miR-8085	NM_001135099	TMPRSS2	1.0	3UTR
hsa-miR-8088	NM_001135099	TMPRSS2	1.0	3UTR
hsa-miR-9985	NM_001135099	TMPRSS2	1.0	3UTR
hsa-miR-10395-5p	NM_001135099	TMPRSS2	1.0	3UTR
hsa-miR-10398-5p	NM_001135099	TMPRSS2	1.0	3UTR
hsa-miR-10526-3p	NM_001135099	TMPRSS2	1.0	3UTR
hsa-miR-11181-3p	NM_001135099	TMPRSS2	1.0	3UTR

hsa-miR-3085-5p	NM_001135099	TMPRSS2	1.0	3UTR
hsa-miR-6529-5p	NM_001135099	TMPRSS2	1.0	3UTR
hsa-miR-9851-5p	NM_001135099	TMPRSS2	1.0	3UTR
hsa-miR-9851-3p	NM_001135099	TMPRSS2	1.0	3UTR
hsa-miR-12116	NM_001135099	TMPRSS2	1.0	3UTR
hsa-miR-12119	NM_001135099	TMPRSS2	1.0	3UTR

Supplementary Table 2

miRNA-target experimental validated interactions (miRNet)

ID	Target	Experiment
hsa-mir-193b-3p	AAMP	CLASH
hsa-mir-193b-3p	AARS	CLASH
hsa-mir-193b-3p	ACACA	CLASH
hsa-mir-193b-3p	ACTG1	CLASH
hsa-mir-193b-3p	ACTN4	CLASH//HITS-CLIP
hsa-mir-193b-3p	ACTN1	Proteomics
hsa-mir-193b-3p	ADARB1	CLASH
hsa-mir-193b-3p	ADCY9	Microarray
hsa-mir-193b-3p	PARP1	CLASH
hsa-mir-193b-3p	AKT1	CLASH
hsa-mir-193b-3p	ALDH3A2	CLASH
hsa-mir-193b-3p	APEH	CLASH
hsa-mir-193b-3p	ARCN1	CLASH
hsa-mir-193b-3p	ATP1A1	Proteomics
hsa-mir-193b-3p	ALDH7A1	CLASH
hsa-mir-193b-3p	ATP5B	Proteomics
hsa-mir-193b-3p	BARD1	Microarray
hsa-mir-193b-3p	BCKDHA	CLASH
hsa-mir-193b-3p	CCND1	CLASH//Immunohistochemistry//Luciferase reporter assay//Microarray//qRT-PCR//Reporter assay//Western blot
hsa-mir-193b-3p	BLM	Microarray
hsa-mir-193b-3p	BRCA1	Microarray
hsa-mir-193b-3p	BUB1	PAR-CLIP
hsa-mir-193b-3p	BUB1B	CLASH//Microarray
hsa-mir-193b-3p	C1QBP	HITS-CLIP
hsa-mir-193b-3p	C5	Microarray
hsa-mir-193b-3p	CAPNS1	CLASH
hsa-mir-193b-3p	CASP9	Microarray
hsa-mir-193b-3p	CBS	CLASH
hsa-mir-193b-3p	KYAT1	CLASH
hsa-mir-193b-3p	CCNA2	Microarray
hsa-mir-193b-3p	CDK1	Microarray
hsa-mir-193b-3p	CDC6	Microarray
hsa-mir-193b-3p	CDC20	Microarray
hsa-mir-193b-3p	CDC25A	Microarray
hsa-mir-193b-3p	CDH1	Proteomics
hsa-mir-193b-3p	CDK4	CLASH
hsa-mir-193b-3p	CDK6	Microarray
hsa-mir-193b-3p	CDK8	CLASH
hsa-mir-193b-3p	CDK9	CLASH
hsa-mir-193b-3p	CFL1	CLASH
hsa-mir-193b-3p	RCC1	CLASH//Microarray
hsa-mir-193b-3p	CHD4	CLASH//Proteomics

hsa-mir-193b-3p	CHEK1	Microarray
hsa-mir-193b-3p	COL4A1	CLASH
hsa-mir-193b-3p	COPA	CLASH
hsa-mir-193b-3p	COX7C	CLASH
hsa-mir-193b-3p	CS	CLASH
hsa-mir-193b-3p	SLC25A10	CLASH
hsa-mir-193b-3p	DDB1	Proteomics
hsa-mir-193b-3p	AKR1C2	Luciferase reporter assay//Proteomics//Reporter assay
hsa-mir-193b-3p	DLX1	Microarray
hsa-mir-193b-3p	DNMT1	Microarray
hsa-mir-193b-3p	DNMT3A	Microarray
hsa-mir-193b-3p	DPH1	CLASH
hsa-mir-193b-3p	DRG2	CLASH
hsa-mir-193b-3p	DUT	Proteomics
hsa-mir-193b-3p	E2F1	Microarray
hsa-mir-193b-3p	E2F2	Microarray
hsa-mir-193b-3p	E2F6	Microarray
hsa-mir-193b-3p	ECH1	Proteomics
hsa-mir-193b-3p	ECT2	Microarray
hsa-mir-193b-3p	EEF2	CLASH
hsa-mir-193b-3p	MEGF8	CLASH
hsa-mir-193b-3p	EPHA2	Microarray
hsa-mir-193b-3p	EIF4B	CLASH//Proteomics
hsa-mir-193b-3p	ELK3	Microarray
hsa-mir-193b-3p	MARK2	CLASH
hsa-mir-193b-3p	EP300	CLASH
hsa-mir-193b-3p	EPHX1	Proteomics
hsa-mir-193b-3p	EPRS	CLASH
hsa-mir-193b-3p	ESD	CLASH
hsa-mir-193b-3p	ESR1	Luciferase reporter assay//Microarray
hsa-mir-193b-3p	ETS1	Luciferase reporter assay//qRT-PCR//Western blot
hsa-mir-193b-3p	EZH2	Microarray
hsa-mir-193b-3p	FANCA	Microarray
hsa-mir-193b-3p	FANCD2	Microarray
hsa-mir-193b-3p	FANCE	Microarray
hsa-mir-193b-3p	FANCG	Microarray
hsa-mir-193b-3p	FASN	CLASH
hsa-mir-193b-3p	FEN1	Microarray//Proteomics
hsa-mir-193b-3p	FGF11	CLASH
hsa-mir-193b-3p	FOXC1	CLASH
hsa-mir-193b-3p	FLNA	CLASH
hsa-mir-193b-3p	KDSR	HITS-CLIP
hsa-mir-193b-3p	SLC37A4	CLASH
hsa-mir-193b-3p	XRCC6	CLASH
hsa-mir-193b-3p	GDI2	CLASH
hsa-mir-193b-3p	GJB2	Microarray
hsa-mir-193b-3p	GLO1	Microarray

hsa-mir-193b-3p	GLUD1	CLASH
hsa-mir-193b-3p	GPI	CLASH
hsa-mir-193b-3p	RAPGEF1	CLASH
hsa-mir-193b-3p	GSS	CLASH
hsa-mir-193b-3p	MSH6	Microarray
hsa-mir-193b-3p	HIST1H1D	Microarray
hsa-mir-193b-3p	HIST1H1E	CLASH
hsa-mir-193b-3p	HIST1H1B	CLASH
hsa-mir-193b-3p	HADHB	Proteomics
hsa-mir-193b-3p	HCFC1	CLASH
hsa-mir-193b-3p	HDGF	CLASH
hsa-mir-193b-3p	HELLS	Microarray
hsa-mir-193b-3p	HMGB1	Microarray
hsa-mir-193b-3p	HMGCR	Microarray
hsa-mir-193b-3p	HNRNPAB	CLASH
hsa-mir-193b-3p	HNRNPH1	CLASH
hsa-mir-193b-3p	HNRNPL	Proteomics
hsa-mir-193b-3p	HPRT1	Microarray
hsa-mir-193b-3p	DNAJA1	CLASH
hsa-mir-193b-3p	HSPA1B	CLASH
hsa-mir-193b-3p	HSPA1L	CLASH
hsa-mir-193b-3p	HSP90AB1	Proteomics
hsa-mir-193b-3p	NDST1	CLASH
hsa-mir-193b-3p	IDI1	Proteomics
hsa-mir-193b-3p	IGFBP5	PAR-CLIP
hsa-mir-193b-3p	INSIG1	CLASH
hsa-mir-193b-3p	IRAK1	CLASH
hsa-mir-193b-3p	IRF1	HITS-CLIP
hsa-mir-193b-3p	ITPKA	Microarray
hsa-mir-193b-3p	KIT	Luciferase reporter assay
hsa-mir-193b-3p	KIF11	Microarray
hsa-mir-193b-3p	KIF22	Microarray
hsa-mir-193b-3p	KPNA2	Proteomics
hsa-mir-193b-3p	KRAS	In situ hybridization//Luciferase reporter assay//Microarray//PAR-CLIP//RTPCR//Western blot
hsa-mir-193b-3p	KRT19	Proteomics
hsa-mir-193b-3p	LAMB1	CLASH
hsa-mir-193b-3p	LAMC1	PAR-CLIP
hsa-mir-193b-3p	STMN1	Microarray
hsa-mir-193b-3p	LASP1	Proteomics
hsa-mir-193b-3p	LETM1	Proteomics
hsa-mir-193b-3p	LMNB1	CLASH
hsa-mir-193b-3p	LOXL1	Microarray
hsa-mir-193b-3p	SMAD3	CLASH//Luciferase reporter assay//qRT-PCR//Western blot
hsa-mir-193b-3p	MAGEB2	CLASH
hsa-mir-193b-3p	MARS	CLASH
hsa-mir-193b-3p	MAT2A	Proteomics

hsa-mir-193b-3p	MAX	In situ hybridization//Luciferase reporter assay//Microarray//RTPCR//Western blot
hsa-mir-193b-3p	MAZ	CLASH
hsa-mir-193b-3p	MCL1	Luciferase reporter assay//Microarray//qRT-PCR//Western blot
hsa-mir-193b-3p	MCM3	Microarray
hsa-mir-193b-3p	MCM4	Microarray//Proteomics
hsa-mir-193b-3p	MCM5	Microarray//Proteomics
hsa-mir-193b-3p	MCM6	Microarray//Proteomics
hsa-mir-193b-3p	MCM7	Microarray//Proteomics
hsa-mir-193b-3p	DNAJB9	PAR-CLIP
hsa-mir-193b-3p	MDH2	CLASH//PAR-CLIP
hsa-mir-193b-3p	MAP3K1	HITS-CLIP
hsa-mir-193b-3p	MAP3K3	CLASH//HITS-CLIP
hsa-mir-193b-3p	MPST	Microarray
hsa-mir-193b-3p	COX1	CLASH
hsa-mir-193b-3p	MTHFD1	Proteomics
hsa-mir-193b-3p	MTR	CLASH
hsa-mir-193b-3p	MYB	Luciferase reporter assay//Microarray
hsa-mir-193b-3p	MYBL1	Microarray
hsa-mir-193b-3p	MYH9	CLASH
hsa-mir-193b-3p	MYLK	Microarray
hsa-mir-193b-3p	MYO1D	CLASH
hsa-mir-193b-3p	MYO5A	CLASH
hsa-mir-193b-3p	NAGA	Microarray
hsa-mir-193b-3p	HNRNPM	CLASH
hsa-mir-193b-3p	NCL	CLASH
hsa-mir-193b-3p	NDUFS6	CLASH
hsa-mir-193b-3p	NF1	CLASH//HITS-CLIP//In situ hybridization//Luciferase reporter assay//qRT-PCR//Western blot
hsa-mir-193b-3p	NF2	Microarray
hsa-mir-193b-3p	NFIA	CLASH
hsa-mir-193b-3p	NFKB2	Microarray
hsa-mir-193b-3p	NME4	CLASH
hsa-mir-193b-3p	NQO2	HITS-CLIP
hsa-mir-193b-3p	NMT1	CLASH
hsa-mir-193b-3p	NONO	CLASH
hsa-mir-193b-3p	NPM1	CLASH
hsa-mir-193b-3p	NPPC	Microarray
hsa-mir-193b-3p	NSF	CLASH
hsa-mir-193b-3p	NUCB1	Proteomics
hsa-mir-193b-3p	NUCB2	Proteomics
hsa-mir-193b-3p	ODC1	CLASH
hsa-mir-193b-3p	OPHN1	PAR-CLIP
hsa-mir-193b-3p	PAK2	CLASH
hsa-mir-193b-3p	PCNA	Microarray
hsa-mir-193b-3p	PCNT	Microarray
hsa-mir-193b-3p	CDK17	CLASH

hsa-mir-193b-3p	PFDN2	Proteomics
hsa-mir-193b-3p	PFKP	CLASH
hsa-mir-193b-3p	PFN1	CLASH
hsa-mir-193b-3p	PGAM1	CLASH
hsa-mir-193b-3p	PGM3	HITS-CLIP
hsa-mir-193b-3p	PIP4K2A	CLASH
hsa-mir-193b-3p	PLAU	HITS-CLIP//Luciferase reporter assay//Microarray//qRT-PCR//Western blot
hsa-mir-193b-3p	PLS3	CLASH
hsa-mir-193b-3p	PMAIP1	CLASH//PAR-CLIP
hsa-mir-193b-3p	EXOSC10	CLASH
hsa-mir-193b-3p	POLA1	Microarray
hsa-mir-193b-3p	POLD1	Microarray
hsa-mir-193b-3p	POLE	Microarray
hsa-mir-193b-3p	POLE2	Microarray
hsa-mir-193b-3p	POLR2C	CLASH
hsa-mir-193b-3p	PPP2R5C	HITS-CLIP
hsa-mir-193b-3p	PRIM1	Microarray
hsa-mir-193b-3p	PRKCA	CLASH
hsa-mir-193b-3p	MAPK8	Microarray//PAR-CLIP
hsa-mir-193b-3p	PRNP	Microarray
hsa-mir-193b-3p	PSMA7	CLASH
hsa-mir-193b-3p	PSMC5	CLASH
hsa-mir-193b-3p	PTEN	CLASH
hsa-mir-193b-3p	PTK7	CLASH
hsa-mir-193b-3p	TWF1	CLASH
hsa-mir-193b-3p	PTMS	Proteomics
hsa-mir-193b-3p	PTPN9	HITS-CLIP
hsa-mir-193b-3p	PTPN11	CLASH
hsa-mir-193b-3p	PTPRG	CLASH
hsa-mir-193b-3p	RABGGTB	CLASH
hsa-mir-193b-3p	RAB5C	Proteomics
hsa-mir-193b-3p	RAC2	Microarray
hsa-mir-193b-3p	RAD51	ChIP-seq//Luciferase reporter assay//Microarray//qRT-PCR//Western blot
hsa-mir-193b-3p	RBBP5	Microarray
hsa-mir-193b-3p	RBL1	PAR-CLIP
hsa-mir-193b-3p	RFC4	Microarray
hsa-mir-193b-3p	RFC5	Microarray
hsa-mir-193b-3p	RPL8	CLASH
hsa-mir-193b-3p	RPL9	CLASH
hsa-mir-193b-3p	RPL12	CLASH
hsa-mir-193b-3p	RPL22	CLASH
hsa-mir-193b-3p	RPL23A	CLASH
hsa-mir-193b-3p	RPL26	CLASH
hsa-mir-193b-3p	RPL27A	CLASH
hsa-mir-193b-3p	RPS3	CLASH

hsa-mir-193b-3p	RPS6KA1	CLASH
hsa-mir-193b-3p	RPS10	CLASH
hsa-mir-193b-3p	RPS18	CLASH
hsa-mir-193b-3p	RPS21	PAR-CLIP
hsa-mir-193b-3p	RRM2	CLASH//Microarray
hsa-mir-193b-3p	RTKN	PAR-CLIP
hsa-mir-193b-3p	SCO1	CLASH
hsa-mir-193b-3p	SFPQ	CLASH
hsa-mir-193b-3p	SRSF1	CLASH
hsa-mir-193b-3p	SH3GL1	CLASH
hsa-mir-193b-3p	SHMT1	Microarray
hsa-mir-193b-3p	SHMT2	CLASH//Luciferase reporter assay//Microarray//Proteomics//Reporter assay
hsa-mir-193b-3p	SIX1	Microarray
hsa-mir-193b-3p	SLC1A5	Proteomics
hsa-mir-193b-3p	SLC3A2	CLASH
hsa-mir-193b-3p	SNRNP70	CLASH
hsa-mir-193b-3p	SNRPB	CLASH
hsa-mir-193b-3p	SNRPD3	CLASH
hsa-mir-193b-3p	SOAT1	Microarray
hsa-mir-193b-3p	CAPN15	CLASH
hsa-mir-193b-3p	SPTBN1	CLASH
hsa-mir-193b-3p	SPTBN2	CLASH
hsa-mir-193b-3p	SSRP1	CLASH
hsa-mir-193b-3p	SURF4	Proteomics
hsa-mir-193b-3p	TACC1	Microarray
hsa-mir-193b-3p	ELOA	CLASH
hsa-mir-193b-3p	TCF7L2	PAR-CLIP
hsa-mir-193b-3p	TERT	Microarray
hsa-mir-193b-3p	TGFBR3	Microarray
hsa-mir-193b-3p	TK1	Microarray
hsa-mir-193b-3p	TLE4	CLASH
hsa-mir-193b-3p	TLN1	HITS-CLIP
hsa-mir-193b-3p	TM7SF2	Proteomics
hsa-mir-193b-3p	TRAPPC10	CLASH
hsa-mir-193b-3p	TNFRSF1B	HITS-CLIP
hsa-mir-193b-3p	TOP2A	Microarray
hsa-mir-193b-3p	TPI1	CLASH
hsa-mir-193b-3p	TSC1	CLASH
hsa-mir-193b-3p	PHLDA2	Microarray//PAR-CLIP
hsa-mir-193b-3p	TYMS	Microarray
hsa-mir-193b-3p	UCHL1	CLASH
hsa-mir-193b-3p	UGP2	Proteomics
hsa-mir-193b-3p	VASP	Proteomics
hsa-mir-193b-3p	VAV2	CLASH
hsa-mir-193b-3p	NSD2	CLASH//Microarray
hsa-mir-193b-3p	XPO1	Proteomics

hsa-mir-193b-3p	XRCC1	CLASH
hsa-mir-193b-3p	YY1	PAR-CLIP
hsa-mir-193b-3p	YWHAZ	CLASH//HITS-CLIP//Luciferase reporter assay//Proteomics//Reporter assay
hsa-mir-193b-3p	ZNF3	CLASH
hsa-mir-193b-3p	SLC30A1	CLASH
hsa-mir-193b-3p	ZYX	CLASH
hsa-mir-193b-3p	PRRC2A	CLASH
hsa-mir-193b-3p	BAG6	CLASH
hsa-mir-193b-3p	AIMP2	Microarray
hsa-mir-193b-3p	KAT6A	CLASH
hsa-mir-193b-3p	NUP214	CLASH
hsa-mir-193b-3p	PDHX	CLASH
hsa-mir-193b-3p	AAAS	CLASH
hsa-mir-193b-3p	SLC7A5	Microarray
hsa-mir-193b-3p	SYMPK	CLASH
hsa-mir-193b-3p	CHAF1B	Microarray
hsa-mir-193b-3p	ARID1A	CLASH
hsa-mir-193b-3p	TRRAP	CLASH
hsa-mir-193b-3p	HIST1H2AJ	CLASH
hsa-mir-193b-3p	HIST2H2AA3	CLASH
hsa-mir-193b-3p	HIST1H3D	CLASH
hsa-mir-193b-3p	HIST1H3H	CLASH
hsa-mir-193b-3p	HIST1H3B	CLASH
hsa-mir-193b-3p	STX7	CLASH
hsa-mir-193b-3p	DYRK2	HITS-CLIP
hsa-mir-193b-3p	CUL4A	CLASH
hsa-mir-193b-3p	TEAD2	CLASH
hsa-mir-193b-3p	IRS4	CLASH
hsa-mir-193b-3p	YBX3	CLASH
hsa-mir-193b-3p	YARS	CLASH
hsa-mir-193b-3p	PDXK	Proteomics
hsa-mir-193b-3p	RUVBL1	CLASH
hsa-mir-193b-3p	SSNA1	CLASH
hsa-mir-193b-3p	EIF3I	CLASH//Proteomics
hsa-mir-193b-3p	VAMP8	Microarray//Proteomics
hsa-mir-193b-3p	STX16	CLASH
hsa-mir-193b-3p	MBTPS1	CLASH
hsa-mir-193b-3p	GBF1	Proteomics
hsa-mir-193b-3p	PEX11B	Microarray
hsa-mir-193b-3p	DLEU2	Microarray
hsa-mir-193b-3p	ALDH1A2	CLASH
hsa-mir-193b-3p	HERC2	CLASH
hsa-mir-193b-3p	HERC1	CLASH
hsa-mir-193b-3p	BTRC	Microarray
hsa-mir-193b-3p	BAZ1B	CLASH
hsa-mir-193b-3p	SPAG9	CLASH

hsa-mir-193b-3p	DOK2	CLASH
hsa-mir-193b-3p	SLC7A6	CLASH
hsa-mir-193b-3p	PKMYT1	Microarray
hsa-mir-193b-3p	DNAJA3	CLASH
hsa-mir-193b-3p	MTMR4	CLASH
hsa-mir-193b-3p	SMC3	CLASH
hsa-mir-193b-3p	FAM50A	CLASH
hsa-mir-193b-3p	CTDP1	CLASH
hsa-mir-193b-3p	EXO1	Microarray
hsa-mir-193b-3p	EBAG9	PAR-CLIP
hsa-mir-193b-3p	DDX21	Proteomics
hsa-mir-193b-3p	MAPKAPK2	CLASH
hsa-mir-193b-3p	CYTH1	Microarray
hsa-mir-193b-3p	TRIP13	Microarray
hsa-mir-193b-3p	TRIP12	CLASH
hsa-mir-193b-3p	EFTUD2	CLASH
hsa-mir-193b-3p	CIAO1	CLASH//HITS-CLIP
hsa-mir-193b-3p	RECQL4	Microarray
hsa-mir-193b-3p	MED21	PAR-CLIP
hsa-mir-193b-3p	TJP2	Microarray
hsa-mir-193b-3p	SH3BP5	CLASH
hsa-mir-193b-3p	TECR	Proteomics
hsa-mir-193b-3p	GOSR1	CLASH
hsa-mir-193b-3p	APOBEC3B	Microarray
hsa-mir-193b-3p	GCC2	CLASH
hsa-mir-193b-3p	ESPL1	Microarray
hsa-mir-193b-3p	KNTC1	Microarray
hsa-mir-193b-3p	CCP110	Microarray
hsa-mir-193b-3p	JADE3	Microarray
hsa-mir-193b-3p	PCLAF	Microarray
hsa-mir-193b-3p	TMEM94	CLASH
hsa-mir-193b-3p	DAZAP2	Microarray
hsa-mir-193b-3p	NUP58	Microarray
hsa-mir-193b-3p	RB1CC1	CLASH
hsa-mir-193b-3p	ARHGAP11A	Microarray
hsa-mir-193b-3p	MELK	Microarray
hsa-mir-193b-3p	AREL1	Microarray
hsa-mir-193b-3p	DENND4B	CLASH
hsa-mir-193b-3p	FAM20B	Microarray
hsa-mir-193b-3p	NCAPD2	Proteomics
hsa-mir-193b-3p	KBTBD11	Microarray
hsa-mir-193b-3p	ZBTB5	Microarray
hsa-mir-193b-3p	LPGAT1	CLASH
hsa-mir-193b-3p	MFN2	CLASH
hsa-mir-193b-3p	RBM8A	CLASH//Microarray
hsa-mir-193b-3p	FARSB	CLASH
hsa-mir-193b-3p	ABCF2	CLASH

hsa-mir-193b-3p	HUWE1	CLASH
hsa-mir-193b-3p	HHLA1	PAR-CLIP
hsa-mir-193b-3p	PPIF	HITS-CLIP
hsa-mir-193b-3p	CTDSP2	Microarray
hsa-mir-193b-3p	ACTR1A	CLASH
hsa-mir-193b-3p	TRAP1	CLASH
hsa-mir-193b-3p	G3BP1	CLASH
hsa-mir-193b-3p	ABI2	Microarray//PAR-CLIP
hsa-mir-193b-3p	PLXNC1	Microarray
hsa-mir-193b-3p	TRIM28	CLASH
hsa-mir-193b-3p	SLC25A15	Microarray
hsa-mir-193b-3p	NUTF2	CLASH
hsa-mir-193b-3p	EIF1	CLASH
hsa-mir-193b-3p	GDF11	PAR-CLIP
hsa-mir-193b-3p	PLIN3	Proteomics
hsa-mir-193b-3p	COQ7	HITS-CLIP
hsa-mir-193b-3p	DCAF7	HITS-CLIP//Microarray//PAR-CLIP
hsa-mir-193b-3p	EFS	Microarray
hsa-mir-193b-3p	SF3A1	CLASH
hsa-mir-193b-3p	TFG	CLASH
hsa-mir-193b-3p	TUBA1B	Proteomics
hsa-mir-193b-3p	TUBB3	CLASH//Proteomics
hsa-mir-193b-3p	NDC80	Microarray
hsa-mir-193b-3p	BASP1	Proteomics
hsa-mir-193b-3p	LYPLA1	HITS-CLIP
hsa-mir-193b-3p	MCRS1	CLASH
hsa-mir-193b-3p	VAV3	Microarray
hsa-mir-193b-3p	TACC3	CLASH//Microarray
hsa-mir-193b-3p	EIF3M	CLASH
hsa-mir-193b-3p	SEC23A	Proteomics
hsa-mir-193b-3p	ENOX2	Microarray
hsa-mir-193b-3p	NCOA2	CLASH
hsa-mir-193b-3p	MYBBP1A	CLASH
hsa-mir-193b-3p	HYOU1	CLASH//Microarray
hsa-mir-193b-3p	IPO8	CLASH
hsa-mir-193b-3p	PITRM1	CLASH
hsa-mir-193b-3p	ANP32B	CLASH
hsa-mir-193b-3p	AGPAT1	Microarray
hsa-mir-193b-3p	CCT4	CLASH
hsa-mir-193b-3p	CCT2	CLASH
hsa-mir-193b-3p	PRPF8	CLASH
hsa-mir-193b-3p	AHSA1	CLASH
hsa-mir-193b-3p	USP39	Microarray
hsa-mir-193b-3p	POLQ	Microarray
hsa-mir-193b-3p	TCFL5	CLASH
hsa-mir-193b-3p	STAG2	CLASH
hsa-mir-193b-3p	HSPH1	CLASH

hsa-mir-193b-3p	TRAFD1	HITS-CLIP
hsa-mir-193b-3p	TXNL4A	CLASH
hsa-mir-193b-3p	PRDX3	Proteomics
hsa-mir-193b-3p	AP3M2	CLASH
hsa-mir-193b-3p	CBX1	Microarray
hsa-mir-193b-3p	TMED2	Proteomics
hsa-mir-193b-3p	EBNA1BP2	CLASH
hsa-mir-193b-3p	YWHAQ	CLASH
hsa-mir-193b-3p	RAB32	PAR-CLIP
hsa-mir-193b-3p	SF3B2	CLASH
hsa-mir-193b-3p	ILVBL	CLASH
hsa-mir-193b-3p	RAB35	CLASH
hsa-mir-193b-3p	SLC35D2	Microarray
hsa-mir-193b-3p	UBE2C	Microarray
hsa-mir-193b-3p	CACFD1	HITS-CLIP
hsa-mir-193b-3p	HNRNPUL1	CLASH
hsa-mir-193b-3p	FAF1	CLASH
hsa-mir-193b-3p	ZWINT	Microarray
hsa-mir-193b-3p	CDC37	Proteomics
hsa-mir-193b-3p	PSIP1	CLASH//Microarray
hsa-mir-193b-3p	BAZ2A	HITS-CLIP
hsa-mir-193b-3p	AKAP13	CLASH
hsa-mir-193b-3p	PACSIN2	CLASH
hsa-mir-193b-3p	SYNRG	HITS-CLIP
hsa-mir-193b-3p	PHB2	CLASH
hsa-mir-193b-3p	OIP5	Microarray
hsa-mir-193b-3p	CASC3	CLASH
hsa-mir-193b-3p	SEC31A	Microarray
hsa-mir-193b-3p	CLSTN1	Microarray
hsa-mir-193b-3p	ZNF365	Microarray
hsa-mir-193b-3p	DHX30	CLASH
hsa-mir-193b-3p	SEPHS2	CLASH
hsa-mir-193b-3p	SEPHS1	Microarray
hsa-mir-193b-3p	TPX2	CLASH
hsa-mir-193b-3p	NT5C2	CLASH
hsa-mir-193b-3p	SPEN	CLASH
hsa-mir-193b-3p	CNOT1	CLASH
hsa-mir-193b-3p	SNRNP200	CLASH
hsa-mir-193b-3p	XPO7	CLASH
hsa-mir-193b-3p	PDXDC1	Proteomics
hsa-mir-193b-3p	ENDOD1	Microarray
hsa-mir-193b-3p	TBC1D9B	CLASH
hsa-mir-193b-3p	WAPL	CLASH
hsa-mir-193b-3p	EMC1	CLASH
hsa-mir-193b-3p	CMTR1	CLASH
hsa-mir-193b-3p	PEG10	CLASH
hsa-mir-193b-3p	KIF1B	Microarray

hsa-mir-193b-3p	ZBTB43	CLASH
hsa-mir-193b-3p	PHF8	CLASH
hsa-mir-193b-3p	CIC	CLASH
hsa-mir-193b-3p	TTLL12	CLASH
hsa-mir-193b-3p	CYFIP1	CLASH
hsa-mir-193b-3p	FAF2	CLASH
hsa-mir-193b-3p	TBC1D1	Microarray
hsa-mir-193b-3p	SYNE2	Microarray
hsa-mir-193b-3p	DNAJC9	Microarray
hsa-mir-193b-3p	ZC3H7B	CLASH
hsa-mir-193b-3p	BICD2	CLASH
hsa-mir-193b-3p	ESYT1	CLASH
hsa-mir-193b-3p	SUN1	CLASH
hsa-mir-193b-3p	USP24	CLASH
hsa-mir-193b-3p	SMG5	CLASH
hsa-mir-193b-3p	PIP5K1C	CLASH
hsa-mir-193b-3p	NCAPH	Microarray
hsa-mir-193b-3p	ATP13A2	CLASH
hsa-mir-193b-3p	COTL1	Proteomics
hsa-mir-193b-3p	SF3B3	CLASH
hsa-mir-193b-3p	SCRIB	CLASH
hsa-mir-193b-3p	DDAH1	PAR-CLIP
hsa-mir-193b-3p	CDC42EP4	Microarray
hsa-mir-193b-3p	ORC6	Microarray
hsa-mir-193b-3p	ACOT9	CLASH
hsa-mir-193b-3p	AMACR	CLASH
hsa-mir-193b-3p	RUSC1	Microarray
hsa-mir-193b-3p	SH3BP4	CLASH
hsa-mir-193b-3p	PITPNB	Microarray
hsa-mir-193b-3p	PRPF40B	Microarray
hsa-mir-193b-3p	GCA	CLASH
hsa-mir-193b-3p	YIPF3	CLASH
hsa-mir-193b-3p	CCDC28A	Microarray
hsa-mir-193b-3p	CLIC4	Proteomics
hsa-mir-193b-3p	WWTR1	Microarray
hsa-mir-193b-3p	KANK2	Microarray
hsa-mir-193b-3p	C2CD2	CLASH
hsa-mir-193b-3p	TSKU	PAR-CLIP
hsa-mir-193b-3p	TENM4	CLASH
hsa-mir-193b-3p	CHTOP	CLASH
hsa-mir-193b-3p	PRPF31	CLASH
hsa-mir-193b-3p	PHF19	Microarray
hsa-mir-193b-3p	RSL1D1	CLASH
hsa-mir-193b-3p	GIMAP2	Microarray
hsa-mir-193b-3p	FBXO5	Microarray
hsa-mir-193b-3p	PLEK2	Microarray
hsa-mir-193b-3p	ago-01	CLASH

hsa-mir-193b-3p	DAZAP1	CLASH
hsa-mir-193b-3p	PABPC1	CLASH
hsa-mir-193b-3p	TRMT2A	CLASH
hsa-mir-193b-3p	PELP1	CLASH
hsa-mir-193b-3p	NSG1	Microarray
hsa-mir-193b-3p	PPA2	Proteomics
hsa-mir-193b-3p	TNFRSF21	Microarray
hsa-mir-193b-3p	AHDC1	CLASH
hsa-mir-193b-3p	RNF115	CLASH
hsa-mir-193b-3p	PRPF19	CLASH
hsa-mir-193b-3p	TMEM97	CLASH
hsa-mir-193b-3p	HDHD5	CLASH
hsa-mir-193b-3p	DCPS	CLASH
hsa-mir-193b-3p	MRPL42	Microarray
hsa-mir-193b-3p	DBNL	CLASH
hsa-mir-193b-3p	ATAD2	CLASH
hsa-mir-193b-3p	ANKRD11	CLASH
hsa-mir-193b-3p	RACGAP1	Microarray
hsa-mir-193b-3p	ABT1	CLASH
hsa-mir-193b-3p	NCAPH2	Microarray
hsa-mir-193b-3p	SENP1	Microarray
hsa-mir-193b-3p	PSMC3IP	Microarray
hsa-mir-193b-3p	GMPPB	CLASH
hsa-mir-193b-3p	SEC61A1	Microarray
hsa-mir-193b-3p	PKN3	Microarray
hsa-mir-193b-3p	PRICKLE4	CLASH
hsa-mir-193b-3p	UBQLN2	CLASH
hsa-mir-193b-3p	DONSON	Microarray
hsa-mir-193b-3p	LMCD1	Microarray
hsa-mir-193b-3p	BICRA	CLASH
hsa-mir-193b-3p	GEMIN4	CLASH
hsa-mir-193b-3p	HP1BP3	CLASH
hsa-mir-193b-3p	NSDHL	Proteomics
hsa-mir-193b-3p	GMNN	Microarray
hsa-mir-193b-3p	NMD3	Proteomics
hsa-mir-193b-3p	NOSIP	Proteomics
hsa-mir-193b-3p	MRPL4	CLASH//Proteomics
hsa-mir-193b-3p	UTP18	HITS-CLIP
hsa-mir-193b-3p	ASB3	HITS-CLIP
hsa-mir-193b-3p	LEF1	Microarray
hsa-mir-193b-3p	GMPR2	CLASH
hsa-mir-193b-3p	TAOK3	CLASH
hsa-mir-193b-3p	SNX9	Microarray
hsa-mir-193b-3p	ANKFY1	HITS-CLIP
hsa-mir-193b-3p	HACD3	Proteomics
hsa-mir-193b-3p	CTDSPL2	CLASH
hsa-mir-193b-3p	SCLY	CLASH

hsa-mir-193b-3p	NT5DC3	Microarray
hsa-mir-193b-3p	METTL13	CLASH
hsa-mir-193b-3p	TAF9B	CLASH
hsa-mir-193b-3p	PTRH2	CLASH
hsa-mir-193b-3p	GINS2	Microarray
hsa-mir-193b-3p	CDK12	CLASH
hsa-mir-193b-3p	RSF1	Microarray
hsa-mir-193b-3p	C11orf24	Microarray
hsa-mir-193b-3p	A4GALT	Microarray
hsa-mir-193b-3p	ERRFI1	Microarray
hsa-mir-193b-3p	SLC38A2	CLASH
hsa-mir-193b-3p	TOLLIP	CLASH
hsa-mir-193b-3p	CHPF2	CLASH
hsa-mir-193b-3p	FBXL19	CLASH
hsa-mir-193b-3p	EPDR1	Microarray
hsa-mir-193b-3p	IL17RD	CLASH
hsa-mir-193b-3p	ALKBH4	CLASH
hsa-mir-193b-3p	SNRK	CLASH
hsa-mir-193b-3p	NSUN2	CLASH
hsa-mir-193b-3p	ALKBH5	HITS-CLIP
hsa-mir-193b-3p	INO80D	PAR-CLIP
hsa-mir-193b-3p	SEMA4C	CLASH
hsa-mir-193b-3p	SARS2	CLASH
hsa-mir-193b-3p	PARP16	Microarray
hsa-mir-193b-3p	HEATR3	CLASH
hsa-mir-193b-3p	CDCA4	Microarray
hsa-mir-193b-3p	C9orf40	Microarray
hsa-mir-193b-3p	WDYHV1	Microarray
hsa-mir-193b-3p	ELP3	CLASH
hsa-mir-193b-3p	UBR7	Microarray
hsa-mir-193b-3p	ARMC1	HITS-CLIP
hsa-mir-193b-3p	TMEM33	Proteomics
hsa-mir-193b-3p	CENPQ	Microarray
hsa-mir-193b-3p	RMDN3	CLASH
hsa-mir-193b-3p	FANCI	Microarray
hsa-mir-193b-3p	ADI1	Proteomics
hsa-mir-193b-3p	C5orf22	PAR-CLIP
hsa-mir-193b-3p	CHDH	Microarray
hsa-mir-193b-3p	COPRS	Microarray
hsa-mir-193b-3p	MCM10	CLASH//Microarray
hsa-mir-193b-3p	STRADB	Microarray
hsa-mir-193b-3p	SVOP	HITS-CLIP
hsa-mir-193b-3p	ZNF823	Microarray
hsa-mir-193b-3p	LRRC40	Microarray
hsa-mir-193b-3p	HIF1AN	CLASH
hsa-mir-193b-3p	IWS1	CLASH
hsa-mir-193b-3p	ASF1B	Microarray

hsa-mir-193b-3p	C1orf112	Microarray
hsa-mir-193b-3p	HHAT	Microarray
hsa-mir-193b-3p	DNAJC11	CLASH
hsa-mir-193b-3p	ENAH	CLASH
hsa-mir-193b-3p	AP5M1	Microarray
hsa-mir-193b-3p	TMEM30A	HITS-CLIP
hsa-mir-193b-3p	WDR12	CLASH
hsa-mir-193b-3p	TDP1	CLASH
hsa-mir-193b-3p	CENPJ	Microarray
hsa-mir-193b-3p	ASH1L	CLASH
hsa-mir-193b-3p	ZNF395	Microarray
hsa-mir-193b-3p	FAM212B	CLASH
hsa-mir-193b-3p	LIN37	CLASH
hsa-mir-193b-3p	SULF2	Microarray
hsa-mir-193b-3p	UBFD1	HITS-CLIP
hsa-mir-193b-3p	MEPCE	CLASH
hsa-mir-193b-3p	LRRC8A	Microarray
hsa-mir-193b-3p	TM9SF3	Proteomics
hsa-mir-193b-3p	STARD7	CLASH//HITS-CLIP//Microarray
hsa-mir-193b-3p	SMCO4	Microarray
hsa-mir-193b-3p	ARNTL2	Microarray
hsa-mir-193b-3p	RGMA	PAR-CLIP
hsa-mir-193b-3p	ATXN7L3	CLASH
hsa-mir-193b-3p	KIF15	Microarray
hsa-mir-193b-3p	CIAPIN1	CLASH
hsa-mir-193b-3p	TIGAR	CLASH//HITS-CLIP
hsa-mir-193b-3p	SELENON	Microarray
hsa-mir-193b-3p	LYRM2	PAR-CLIP
hsa-mir-193b-3p	S100A14	Proteomics
hsa-mir-193b-3p	SPC25	Microarray
hsa-mir-193b-3p	GATAD2B	CLASH
hsa-mir-193b-3p	CNOT6	Microarray
hsa-mir-193b-3p	ZNF512B	CLASH//Microarray
hsa-mir-193b-3p	ESYT2	Proteomics
hsa-mir-193b-3p	HACE1	Microarray
hsa-mir-193b-3p	NUFIP2	HITS-CLIP
hsa-mir-193b-3p	MIB1	CLASH
hsa-mir-193b-3p	KLHL42	PAR-CLIP
hsa-mir-193b-3p	CRAMP1	CLASH
hsa-mir-193b-3p	DHX37	CLASH
hsa-mir-193b-3p	PHRF1	CLASH
hsa-mir-193b-3p	ZNF317	CLASH
hsa-mir-193b-3p	FANCM	Microarray
hsa-mir-193b-3p	C6orf47	PAR-CLIP
hsa-mir-193b-3p	ZNF71	CLASH
hsa-mir-193b-3p	SINHCAF	CLASH
hsa-mir-193b-3p	PLEKHA2	HITS-CLIP

hsa-mir-193b-3p	EEFSEC	CLASH
hsa-mir-193b-3p	HEATR6	Proteomics
hsa-mir-193b-3p	FAM111A	Microarray
hsa-mir-193b-3p	ELMO2	HITS-CLIP//Microarray
hsa-mir-193b-3p	CHTF18	Microarray
hsa-mir-193b-3p	MCCC2	Proteomics
hsa-mir-193b-3p	TMBIM1	CLASH
hsa-mir-193b-3p	DUS1L	CLASH
hsa-mir-193b-3p	NCAPG	Microarray
hsa-mir-193b-3p	IRF2BPL	PAR-CLIP
hsa-mir-193b-3p	NFKBIZ	CLASH
hsa-mir-193b-3p	ZMAT3	PAR-CLIP
hsa-mir-193b-3p	MTMR14	CLASH
hsa-mir-193b-3p	GIGYF1	CLASH
hsa-mir-193b-3p	ANAPC1	CLASH
hsa-mir-193b-3p	NUCKS1	CLASH
hsa-mir-193b-3p	C16orf58	CLASH
hsa-mir-193b-3p	C6orf106	HITS-CLIP
hsa-mir-193b-3p	GINS3	Microarray
hsa-mir-193b-3p	TUT1	CLASH
hsa-mir-193b-3p	MRPS9	Proteomics
hsa-mir-193b-3p	MRPL32	CLASH
hsa-mir-193b-3p	NOL6	CLASH
hsa-mir-193b-3p	SOWAHC	CLASH
hsa-mir-193b-3p	WNK1	CLASH
hsa-mir-193b-3p	UBE2Z	CLASH
hsa-mir-193b-3p	FUNDC2	CLASH
hsa-mir-193b-3p	TRAK2	CLASH
hsa-mir-193b-3p	PCYOX1L	Microarray
hsa-mir-193b-3p	AUNIP	Microarray
hsa-mir-193b-3p	NUP37	CLASH
hsa-mir-193b-3p	C7orf26	CLASH
hsa-mir-193b-3p	KXD1	CLASH
hsa-mir-193b-3p	TSEN34	CLASH
hsa-mir-193b-3p	METTL16	CLASH
hsa-mir-193b-3p	DSCC1	Microarray
hsa-mir-193b-3p	BRCC3	CLASH
hsa-mir-193b-3p	TANGO6	CLASH
hsa-mir-193b-3p	ARMC7	CLASH
hsa-mir-193b-3p	MAP7D3	Microarray
hsa-mir-193b-3p	TMEM204	Microarray
hsa-mir-193b-3p	RTL10	CLASH
hsa-mir-193b-3p	CENPU	Microarray
hsa-mir-193b-3p	MSANTD2	PAR-CLIP
hsa-mir-193b-3p	QTRT2	CLASH
hsa-mir-193b-3p	MORC4	Microarray
hsa-mir-193b-3p	IPO4	CLASH

hsa-mir-193b-3p	ISOC2	CLASH
hsa-mir-193b-3p	IQCA1	CLASH
hsa-mir-193b-3p	MYH14	CLASH
hsa-mir-193b-3p	C12orf49	Microarray
hsa-mir-193b-3p	SHCBP1	Microarray
hsa-mir-193b-3p	PIP4K2C	Proteomics
hsa-mir-193b-3p	BORA	Microarray
hsa-mir-193b-3p	LPCAT1	HITS-CLIP//Microarray
hsa-mir-193b-3p	DSN1	Microarray
hsa-mir-193b-3p	RMI1	CLASH
hsa-mir-193b-3p	FBXL18	CLASH
hsa-mir-193b-3p	VCPIP1	CLASH
hsa-mir-193b-3p	PTGES2	Proteomics
hsa-mir-193b-3p	CTC1	HITS-CLIP//PAR-CLIP
hsa-mir-193b-3p	TEDC2	Microarray
hsa-mir-193b-3p	EFHD1	Microarray
hsa-mir-193b-3p	PUS1	CLASH
hsa-mir-193b-3p	WDR82	HITS-CLIP
hsa-mir-193b-3p	REEP4	CLASH
hsa-mir-193b-3p	THAP7	CLASH
hsa-mir-193b-3p	INTS5	CLASH
hsa-mir-193b-3p	CEP44	Microarray
hsa-mir-193b-3p	PTDSS2	CLASH
hsa-mir-193b-3p	HM13	CLASH
hsa-mir-193b-3p	YIPF5	CLASH
hsa-mir-193b-3p	CLPB	PAR-CLIP
hsa-mir-193b-3p	APOLD1	Microarray
hsa-mir-193b-3p	CDT1	Microarray
hsa-mir-193b-3p	RNF146	HITS-CLIP
hsa-mir-193b-3p	EMC6	CLASH
hsa-mir-193b-3p	EPPK1	Proteomics
hsa-mir-193b-3p	RASSF5	CLASH
hsa-mir-193b-3p	SESN2	Microarray
hsa-mir-193b-3p	LONP2	CLASH
hsa-mir-193b-3p	RBM4B	CLASH
hsa-mir-193b-3p	TMTC1	Microarray
hsa-mir-193b-3p	CDCA7	Microarray
hsa-mir-193b-3p	KCTD10	CLASH
hsa-mir-193b-3p	HASPIN	CLASH//Microarray
hsa-mir-193b-3p	SPRTN	PAR-CLIP
hsa-mir-193b-3p	KREMEN1	CLASH
hsa-mir-193b-3p	MND1	Microarray
hsa-mir-193b-3p	ENKD1	CLASH
hsa-mir-193b-3p	TMEM164	Microarray
hsa-mir-193b-3p	SLC25A33	CLASH
hsa-mir-193b-3p	CCDC77	Microarray
hsa-mir-193b-3p	ACBD6	CLASH

hsa-mir-193b-3p	LCOR	CLASH
hsa-mir-193b-3p	MCM8	Microarray
hsa-mir-193b-3p	DCTN5	PAR-CLIP
hsa-mir-193b-3p	KIAA1841	HITS-CLIP
hsa-mir-193b-3p	PSRC1	Microarray
hsa-mir-193b-3p	PRRC2B	CLASH
hsa-mir-193b-3p	LMNB2	CLASH
hsa-mir-193b-3p	KLHL22	CLASH
hsa-mir-193b-3p	NFATC2IP	CLASH
hsa-mir-193b-3p	ATOH8	Microarray
hsa-mir-193b-3p	ARHGAP19	CLASH//Microarray
hsa-mir-193b-3p	REPS1	CLASH
hsa-mir-193b-3p	STRIP1	CLASH
hsa-mir-193b-3p	TUBGCP6	CLASH
hsa-mir-193b-3p	TICRR	Microarray
hsa-mir-193b-3p	CCDC32	Microarray
hsa-mir-193b-3p	JPT2	Proteomics
hsa-mir-193b-3p	MSANTD3	Microarray
hsa-mir-193b-3p	PXYLP1	Microarray
hsa-mir-193b-3p	MRRF	PAR-CLIP
hsa-mir-193b-3p	TIMM50	Proteomics
hsa-mir-193b-3p	HAUS8	Microarray
hsa-mir-193b-3p	MAPK1IP1L	CLASH
hsa-mir-193b-3p	CEP41	Microarray
hsa-mir-193b-3p	MYL12B	CLASH
hsa-mir-193b-3p	SNX18	CLASH
hsa-mir-193b-3p	MTFR2	Microarray
hsa-mir-193b-3p	CDCA5	Microarray
hsa-mir-193b-3p	SCAMP4	CLASH
hsa-mir-193b-3p	CHST14	Microarray
hsa-mir-193b-3p	NLRP3	Microarray
hsa-mir-193b-3p	C1QTNF2	Microarray
hsa-mir-193b-3p	ZNF618	Microarray
hsa-mir-193b-3p	NT5C3B	CLASH
hsa-mir-193b-3p	ARHGAP33	CLASH
hsa-mir-193b-3p	SLC18B1	CLASH
hsa-mir-193b-3p	SSX2IP	Microarray
hsa-mir-193b-3p	PRAP1	Luciferase reporter assay
hsa-mir-193b-3p	ZFYVE27	CLASH
hsa-mir-193b-3p	SFXN2	Microarray
hsa-mir-193b-3p	LRR1	Microarray
hsa-mir-193b-3p	RAVER1	Proteomics
hsa-mir-193b-3p	TYW3	Microarray
hsa-mir-193b-3p	AHSA2	CLASH
hsa-mir-193b-3p	PPARGC1B	CLASH
hsa-mir-193b-3p	PRRC1	Proteomics
hsa-mir-193b-3p	WDR36	CLASH

hsa-mir-193b-3p	PM20D2	Microarray
hsa-mir-193b-3p	S100A16	Proteomics
hsa-mir-193b-3p	CEP128	Microarray
hsa-mir-193b-3p	TMC8	Microarray
hsa-mir-193b-3p	TRIM16L	CLASH
hsa-mir-193b-3p	APCDD1	Microarray
hsa-mir-193b-3p	TICAM1	CLASH
hsa-mir-193b-3p	SLC30A7	Microarray
hsa-mir-193b-3p	YDJC	CLASH
hsa-mir-193b-3p	FAM109B	Microarray
hsa-mir-193b-3p	CKAP2L	Microarray
hsa-mir-193b-3p	GAREM2	CLASH
hsa-mir-193b-3p	ERFE	Microarray
hsa-mir-193b-3p	SGO1	Microarray
hsa-mir-193b-3p	CDCA2	Microarray
hsa-mir-193b-3p	SLC35G1	Microarray
hsa-mir-193b-3p	PARP15	HITS-CLIP
hsa-mir-193b-3p	DACT2	CLASH
hsa-mir-193b-3p	ZNF384	HITS-CLIP
hsa-mir-193b-3p	ASXL1	CLASH
hsa-mir-193b-3p	RHOV	CLASH
hsa-mir-193b-3p	UBR1	CLASH
hsa-mir-193b-3p	TXLNA	CLASH
hsa-mir-193b-3p	TET3	HITS-CLIP
hsa-mir-193b-3p	HACD2	Proteomics
hsa-mir-193b-3p	SMIM14	PAR-CLIP
hsa-mir-193b-3p	TUBB	CLASH
hsa-mir-193b-3p	SENP5	HITS-CLIP
hsa-mir-193b-3p	ATAD3C	CLASH
hsa-mir-193b-3p	UNC5B	CLASH
hsa-mir-193b-3p	CCNY	CLASH
hsa-mir-193b-3p	RTKN2	Microarray
hsa-mir-193b-3p	SKA1	Microarray
hsa-mir-193b-3p	SKA3	CLASH
hsa-mir-193b-3p	DAGLB	Proteomics
hsa-mir-193b-3p	NAPEPLD	PAR-CLIP
hsa-mir-193b-3p	RNASEH1	PAR-CLIP
hsa-mir-193b-3p	NEIL2	CLASH
hsa-mir-193b-3p	GPATCH11	Microarray
hsa-mir-193b-3p	ASPM	Microarray
hsa-mir-193b-3p	BCL9L	CLASH
hsa-mir-193b-3p	RASSF3	Microarray
hsa-mir-193b-3p	GXYLT1	HITS-CLIP
hsa-mir-193b-3p	WDR62	Microarray
hsa-mir-193b-3p	RPL7L1	CLASH
hsa-mir-193b-3p	HIST2H3A	CLASH
hsa-mir-193b-3p	NAT8L	CLASH

hsa-mir-193b-3p	SLC10A6	HITS-CLIP
hsa-mir-193b-3p	RFLNB	Microarray
hsa-mir-193b-3p	NHLRC2	Microarray
hsa-mir-193b-3p	KMT5A	Microarray
hsa-mir-193b-3p	LOC391247	Microarray
hsa-mir-193b-3p	FAM221B	HITS-CLIP
hsa-mir-193b-3p	MEX3D	PAR-CLIP
hsa-mir-193b-3p	MYO18A	CLASH
hsa-mir-193b-3p	CHCHD10	CLASH
hsa-mir-193b-3p	TOMM5	CLASH
hsa-mir-193b-3p	MXRA7	CLASH
hsa-mir-193b-3p	NRARP	CLASH
hsa-mir-193b-3p	PEF1	CLASH
hsa-mir-193b-3p	DHFRP1	Microarray
hsa-mir-193b-3p	TMPPE	PAR-CLIP
hsa-mir-193b-3p	SHISA9	PAR-CLIP
hsa-mir-193b-3p	ZNF814	CLASH
hsa-mir-193b-3p	EPOP	CLASH
hsa-mir-193b-3p	HSBP1P2	Microarray
hsa-mir-503-5p	AP2B1	PAR-CLIP
hsa-mir-503-5p	ANK3	HITS-CLIP
hsa-mir-503-5p	ATP5G3	PAR-CLIP
hsa-mir-503-5p	ATP6V1B2	CLASH
hsa-mir-503-5p	CCND1	Immunohistochemistry//Luciferase reporter assay//Northern blot//PAR-CLIP//qRT-PCR//Western blot
hsa-mir-503-5p	BCL2	Flow//Immunohistochemistry//Immunoprecipitaion//Luciferase reporter assay//qRT-PCR//Western blot
hsa-mir-503-5p	CA8	PAR-CLIP
hsa-mir-503-5p	CANX	PAR-CLIP
hsa-mir-503-5p	CAPZA2	PAR-CLIP
hsa-mir-503-5p	CCND2	PAR-CLIP
hsa-mir-503-5p	CCND3	Flow//qRT-PCR//Western blot
hsa-mir-503-5p	CCNE1	Luciferase reporter assay//PAR-CLIP
hsa-mir-503-5p	CCNF	Luciferase reporter assay
hsa-mir-503-5p	CCNG2	CLASH
hsa-mir-503-5p	CCNT1	PAR-CLIP
hsa-mir-503-5p	CD40	Luciferase reporter assay
hsa-mir-503-5p	CDC25A	Luciferase reporter assay//PAR-CLIP
hsa-mir-503-5p	CDKN1A	Luciferase reporter assay
hsa-mir-503-5p	CHEK1	HITS-CLIP//Immunohistochemistry//Luciferase reporter assay//qRT-PCR//Western blot
hsa-mir-503-5p	CLTC	CLASH
hsa-mir-503-5p	COL1A1	PAR-CLIP
hsa-mir-503-5p	CREBL2	PAR-CLIP
hsa-mir-503-5p	CRK	PAR-CLIP
hsa-mir-503-5p	CTSD	CLASH
hsa-mir-503-5p	DDX3X	PAR-CLIP
hsa-mir-503-5p	DECR1	PAR-CLIP

hsa-mir-503-5p	DHFR	CLASH
hsa-mir-503-5p	DLST	CLASH
hsa-mir-503-5p	E2F3	Luciferase reporter assay//Western blot
hsa-mir-503-5p	EFNB2	PAR-CLIP
hsa-mir-503-5p	EPOR	HITS-CLIP
hsa-mir-503-5p	EXT1	PAR-CLIP
hsa-mir-503-5p	FANCA	LacZ reporter assay//qRT-PCR
hsa-mir-503-5p	FARSA	CLASH
hsa-mir-503-5p	FGF2	Immunohistochemistry//Luciferase reporter assay//Microarray//Northern blot//PAR-CLIP//qRT-PCR
hsa-mir-503-5p	FGF8	Luciferase reporter assay//qRT-PCR//Western blot
hsa-mir-503-5p	FGFR1	Immunohistochemistry//Luciferase reporter assay//Microarray//Northern blot//qRT-PCR
hsa-mir-503-5p	NR6A1	PAR-CLIP
hsa-mir-503-5p	GNAT1	HITS-CLIP//PAR-CLIP
hsa-mir-503-5p	GPR27	PAR-CLIP
hsa-mir-503-5p	HDGF	CLASH
hsa-mir-503-5p	HNRNPA1	HITS-CLIP
hsa-mir-503-5p	HNRNPK	CLASH
hsa-mir-503-5p	HSPA8	PAR-CLIP
hsa-mir-503-5p	IGF1R	Immunohistochemistry//Luciferase reporter assay//qRT-PCR//Western blot
hsa-mir-503-5p	IKBKB	Immunohistochemistry//In situ hybridization//Luciferase reporter assay//qRT-PCR//Western blot
hsa-mir-503-5p	IVD	CLASH
hsa-mir-503-5p	JARID2	PAR-CLIP
hsa-mir-503-5p	KIF5B	PAR-CLIP
hsa-mir-503-5p	KPNA3	HITS-CLIP//PAR-CLIP
hsa-mir-503-5p	SMAD2	HITS-CLIP
hsa-mir-503-5p	SMAD7	PAR-CLIP
hsa-mir-503-5p	MCM7	CLASH
hsa-mir-503-5p	MT1E	CLASH
hsa-mir-503-5p	MYB	Luciferase reporter assay//qRT-PCR//Western blot
hsa-mir-503-5p	MYO5A	PAR-CLIP
hsa-mir-503-5p	OCRL	HITS-CLIP
hsa-mir-503-5p	ORC4	PAR-CLIP
hsa-mir-503-5p	CDK17	PAR-CLIP
hsa-mir-503-5p	PHKA1	HITS-CLIP//PAR-CLIP
hsa-mir-503-5p	PIK3R1	HITS-CLIP//Immunohistochemistry//In situ hybridization//Luciferase reporter assay//PAR-CLIP//qRT-PCR//Western blot
hsa-mir-503-5p	PLAG1	PAR-CLIP
hsa-mir-503-5p	CTSA	CLASH
hsa-mir-503-5p	PPM1A	PAR-CLIP
hsa-mir-503-5p	PPP2R5C	PAR-CLIP
hsa-mir-503-5p	PRKAR2A	PAR-CLIP
hsa-mir-503-5p	PTPRD	PAR-CLIP
hsa-mir-503-5p	MAP4K2	PAR-CLIP

hsa-mir-503-5p	REL	PAR-CLIP
hsa-mir-503-5p	RPL15	CLASH
hsa-mir-503-5p	RPL18A	CLASH
hsa-mir-503-5p	RPS5	CLASH
hsa-mir-503-5p	RPS6KA3	CLASH
hsa-mir-503-5p	RS1	HITS-CLIP
hsa-mir-503-5p	SALL1	PAR-CLIP
hsa-mir-503-5p	SBF1	CLASH
hsa-mir-503-5p	SIAH2	CLASH
hsa-mir-503-5p	SKI	PAR-CLIP
hsa-mir-503-5p	SLC2A3	PAR-CLIP
hsa-mir-503-5p	SNRPB2	PAR-CLIP
hsa-mir-503-5p	SNTB2	PAR-CLIP
hsa-mir-503-5p	SRPRA	HITS-CLIP//PAR-CLIP
hsa-mir-503-5p	MAP3K7	PAR-CLIP
hsa-mir-503-5p	PPP1R11	HITS-CLIP
hsa-mir-503-5p	TFAP2A	PAR-CLIP
hsa-mir-503-5p	TLL1	PAR-CLIP
hsa-mir-503-5p	TRAPPC10	PAR-CLIP
hsa-mir-503-5p	UGT2B4	PAR-CLIP
hsa-mir-503-5p	VEGFA	HITS-CLIP//Luciferase reporter assay//PAR-CLIP
hsa-mir-503-5p	EIF4H	PAR-CLIP
hsa-mir-503-5p	WEE1	Luciferase reporter assay//PAR-CLIP
hsa-mir-503-5p	ZNF282	PAR-CLIP
hsa-mir-503-5p	RECK	PAR-CLIP
hsa-mir-503-5p	CUL3	HITS-CLIP
hsa-mir-503-5p	CBX4	PAR-CLIP
hsa-mir-503-5p	CDC14A	Luciferase reporter assay
hsa-mir-503-5p	CASK	HITS-CLIP//PAR-CLIP
hsa-mir-503-5p	SLC25A12	PAR-CLIP
hsa-mir-503-5p	DNAH17	PAR-CLIP
hsa-mir-503-5p	NAPG	HITS-CLIP//PAR-CLIP
hsa-mir-503-5p	TNFRSF11A	Luciferase reporter assay//Microarray//Northern blot//qRT-PCR//Western blot
hsa-mir-503-5p	MTMR3	PAR-CLIP
hsa-mir-503-5p	CCNE2	Luciferase reporter assay//PAR-CLIP
hsa-mir-503-5p	SLC33A1	HITS-CLIP
hsa-mir-503-5p	B4GALT5	CLASH//PAR-CLIP
hsa-mir-503-5p	GLP2R	HITS-CLIP
hsa-mir-503-5p	RPL23	CLASH
hsa-mir-503-5p	KIF23	PAR-CLIP
hsa-mir-503-5p	SOCS5	PAR-CLIP
hsa-mir-503-5p	N4BP1	PAR-CLIP
hsa-mir-503-5p	BZW1	PAR-CLIP
hsa-mir-503-5p	TOMM20	CLASH
hsa-mir-503-5p	TSC22D2	PAR-CLIP
hsa-mir-503-5p	TLK1	PAR-CLIP

hsa-mir-503-5p	SEC16A	PAR-CLIP
hsa-mir-503-5p	DCLRE1A	CLASH
hsa-mir-503-5p	DMTF1	PAR-CLIP
hsa-mir-503-5p	AKT3	PAR-CLIP
hsa-mir-503-5p	ACTR2	PAR-CLIP
hsa-mir-503-5p	RNF41	PAR-CLIP
hsa-mir-503-5p	CTDSPL	PAR-CLIP
hsa-mir-503-5p	LANCL1	PAR-CLIP
hsa-mir-503-5p	BTN3A3	PAR-CLIP
hsa-mir-503-5p	AHSA1	CLASH
hsa-mir-503-5p	HEXIM1	CLASH
hsa-mir-503-5p	NUP50	PAR-CLIP
hsa-mir-503-5p	SEC24A	PAR-CLIP
hsa-mir-503-5p	PNPLA6	PAR-CLIP
hsa-mir-503-5p	PRSS21	PAR-CLIP
hsa-mir-503-5p	PRDM4	PAR-CLIP
hsa-mir-503-5p	TRAK1	HITS-CLIP
hsa-mir-503-5p	ATF6	Luciferase reporter assay
hsa-mir-503-5p	SETD1B	PAR-CLIP
hsa-mir-503-5p	TRIM35	PAR-CLIP
hsa-mir-503-5p	KANK1	PAR-CLIP
hsa-mir-503-5p	DDHD2	PAR-CLIP
hsa-mir-503-5p	LARP1	PAR-CLIP
hsa-mir-503-5p	SF3B3	CLASH
hsa-mir-503-5p	NNT	PAR-CLIP
hsa-mir-503-5p	CD2AP	PAR-CLIP
hsa-mir-503-5p	TMEM245	HITS-CLIP//PAR-CLIP
hsa-mir-503-5p	PISD	PAR-CLIP
hsa-mir-503-5p	MOB4	PAR-CLIP
hsa-mir-503-5p	SZRD1	PAR-CLIP
hsa-mir-503-5p	WIPI2	PAR-CLIP
hsa-mir-503-5p	ago-01	Luciferase reporter assay
hsa-mir-503-5p	ZBTB44	PAR-CLIP
hsa-mir-503-5p	HCFC2	PAR-CLIP
hsa-mir-503-5p	SEC61A1	PAR-CLIP
hsa-mir-503-5p	PSAT1	PAR-CLIP
hsa-mir-503-5p	PIK3R4	CLASH
hsa-mir-503-5p	CUZD1	CLASH
hsa-mir-503-5p	ASCC1	PAR-CLIP
hsa-mir-503-5p	ZNF691	PAR-CLIP
hsa-mir-503-5p	NT5DC3	HITS-CLIP
hsa-mir-503-5p	MRPS23	CLASH
hsa-mir-503-5p	CDK12	CLASH
hsa-mir-503-5p	DNAJC10	PAR-CLIP
hsa-mir-503-5p	ANLN	Luciferase reporter assay
hsa-mir-503-5p	USP53	PAR-CLIP
hsa-mir-503-5p	CNNM2	PAR-CLIP

hsa-mir-503-5p	CDCA4	PAR-CLIP
hsa-mir-503-5p	RIF1	PAR-CLIP
hsa-mir-503-5p	SBNO1	PAR-CLIP
hsa-mir-503-5p	MRGBP	CLASH
hsa-mir-503-5p	TMEM100	PAR-CLIP
hsa-mir-503-5p	FBXW7	Luciferase reporter assay//Western blot
hsa-mir-503-5p	PI4K2B	PAR-CLIP
hsa-mir-503-5p	RFK	PAR-CLIP
hsa-mir-503-5p	PNRC2	PAR-CLIP
hsa-mir-503-5p	CDC37L1	HITS-CLIP//PAR-CLIP
hsa-mir-503-5p	NPLOC4	PAR-CLIP
hsa-mir-503-5p	ASH1L	PAR-CLIP
hsa-mir-503-5p	UBFD1	CLASH
hsa-mir-503-5p	CYP26B1	PAR-CLIP
hsa-mir-503-5p	CDC42SE2	PAR-CLIP
hsa-mir-503-5p	RTN4	PAR-CLIP
hsa-mir-503-5p	RALGAPB	PAR-CLIP
hsa-mir-503-5p	ACTR3B	PAR-CLIP
hsa-mir-503-5p	ODF2L	PAR-CLIP
hsa-mir-503-5p	NUFIP2	PAR-CLIP
hsa-mir-503-5p	TAOK1	PAR-CLIP
hsa-mir-503-5p	KIAA1456	PAR-CLIP
hsa-mir-503-5p	DLGAP3	PAR-CLIP
hsa-mir-503-5p	PLEKHA1	PAR-CLIP
hsa-mir-503-5p	GREM2	PAR-CLIP
hsa-mir-503-5p	ZMAT3	PAR-CLIP
hsa-mir-503-5p	NUCKS1	CLASH
hsa-mir-503-5p	CERK	PAR-CLIP
hsa-mir-503-5p	TUT1	CLASH
hsa-mir-503-5p	PAPOLG	PAR-CLIP
hsa-mir-503-5p	RAPH1	PAR-CLIP
hsa-mir-503-5p	WNK3	PAR-CLIP
hsa-mir-503-5p	CHAC1	PAR-CLIP
hsa-mir-503-5p	DCAF10	PAR-CLIP
hsa-mir-503-5p	YRDC	PAR-CLIP
hsa-mir-503-5p	ZFHX4	HITS-CLIP//PAR-CLIP
hsa-mir-503-5p	ATAD5	PAR-CLIP
hsa-mir-503-5p	L2HGDH	PAR-CLIP
hsa-mir-503-5p	FBXL18	PAR-CLIP
hsa-mir-503-5p	DCAF17	HITS-CLIP
hsa-mir-503-5p	VOPP1	PAR-CLIP
hsa-mir-503-5p	RCC1L	CLASH
hsa-mir-503-5p	C1orf21	PAR-CLIP
hsa-mir-503-5p	TXNDC5	PAR-CLIP
hsa-mir-503-5p	GSG1	PAR-CLIP
hsa-mir-503-5p	BCL2L12	PAR-CLIP
hsa-mir-503-5p	SPNS1	CLASH

hsa-mir-503-5p	ZNRF3	PAR-CLIP
hsa-mir-503-5p	USP48	PAR-CLIP
hsa-mir-503-5p	DCTN5	PAR-CLIP
hsa-mir-503-5p	CBX2	PAR-CLIP
hsa-mir-503-5p	KIAA1671	CLASH
hsa-mir-503-5p	ZNF622	PAR-CLIP
hsa-mir-503-5p	SESTD1	PAR-CLIP
hsa-mir-503-5p	YTHDC1	PAR-CLIP
hsa-mir-503-5p	ELMSAN1	PAR-CLIP
hsa-mir-503-5p	SCAMP4	PAR-CLIP
hsa-mir-503-5p	RAB3IP	PAR-CLIP
hsa-mir-503-5p	MRPL10	CLASH
hsa-mir-503-5p	OSCAR	PAR-CLIP
hsa-mir-503-5p	UHMK1	CLASH
hsa-mir-503-5p	LIX1L	CLASH
hsa-mir-503-5p	ARHGEF19	Luciferase reporter assay//qRT-PCR//Western blot
hsa-mir-503-5p	UBR3	HITS-CLIP//PAR-CLIP
hsa-mir-503-5p	MTPN	CLASH
hsa-mir-503-5p	DYNLL2	PAR-CLIP
hsa-mir-503-5p	SREK1	HITS-CLIP//PAR-CLIP
hsa-mir-503-5p	CACUL1	PAR-CLIP
hsa-mir-503-5p	HNRNPA1L2	HITS-CLIP
hsa-mir-503-5p	CMTM4	CLASH
hsa-mir-503-5p	CREBRF	PAR-CLIP
hsa-mir-503-5p	CNKSR3	PAR-CLIP
hsa-mir-503-5p	SPRED1	PAR-CLIP
hsa-mir-503-5p	ZNF367	HITS-CLIP//PAR-CLIP
hsa-mir-503-5p	ZNF449	PAR-CLIP
hsa-mir-503-5p	CCDC83	PAR-CLIP
hsa-mir-503-5p	FOXK1	PAR-CLIP
hsa-mir-503-5p	LRWD1	PAR-CLIP
hsa-mir-503-5p	ZNRF2	PAR-CLIP
hsa-mir-503-5p	ZNF620	PAR-CLIP
hsa-mir-503-5p	UBN2	PAR-CLIP
hsa-mir-503-5p	RNF149	HITS-CLIP//PAR-CLIP
hsa-mir-503-5p	LURAP1L	PAR-CLIP
hsa-mir-503-5p	XKR7	PAR-CLIP
hsa-mir-503-5p	NDUFA4P1	PAR-CLIP
hsa-mir-503-5p	ZBTB34	PAR-CLIP
hsa-mir-503-5p	FAM229B	PAR-CLIP
hsa-mir-503-5p	ZNF704	PAR-CLIP
hsa-mir-503-5p	KIAA0895L	CLASH
hsa-mir-503-5p	HSPE1-MOB4	PAR-CLIP
hsa-mir-455-5p	APLP2	PAR-CLIP
hsa-mir-455-5p	TRIM23	CLASH
hsa-mir-455-5p	RHOH	HITS-CLIP
hsa-mir-455-5p	RUNX1T1	HITS-CLIP//PAR-CLIP

hsa-mir-455-5p	CD36	HITS-CLIP
hsa-mir-455-5p	CDKN1B	PAR-CLIP
hsa-mir-455-5p	CRKL	PAR-CLIP
hsa-mir-455-5p	DDX3X	PAR-CLIP
hsa-mir-455-5p	DYRK1A	HITS-CLIP
hsa-mir-455-5p	ERCC4	PAR-CLIP
hsa-mir-455-5p	ETS2	PAR-CLIP
hsa-mir-455-5p	HOXA1	PAR-CLIP
hsa-mir-455-5p	KPNA3	HITS-CLIP//PAR-CLIP
hsa-mir-455-5p	MAP3K9	PAR-CLIP
hsa-mir-455-5p	MYBL1	PAR-CLIP
hsa-mir-455-5p	DRG1	CLASH
hsa-mir-455-5p	OTX1	HITS-CLIP//PAR-CLIP
hsa-mir-455-5p	PCCA	CLASH
hsa-mir-455-5p	PCCB	HITS-CLIP
hsa-mir-455-5p	PIK3R1	HITS-CLIP//PAR-CLIP
hsa-mir-455-5p	PTPRB	PAR-CLIP
hsa-mir-455-5p	REL	HITS-CLIP
hsa-mir-455-5p	RPS6KB1	PAR-CLIP//qRT-PCR
hsa-mir-455-5p	RPS14	PAR-CLIP
hsa-mir-455-5p	SLC1A5	HITS-CLIP
hsa-mir-455-5p	SOX11	PAR-CLIP
hsa-mir-455-5p	UBA7	Luciferase reporter assay//qRT-PCR//Western blot
hsa-mir-455-5p	ZNF134	PAR-CLIP
hsa-mir-455-5p	ZNF138	PAR-CLIP
hsa-mir-455-5p	ZFAND5	PAR-CLIP
hsa-mir-455-5p	SOCS3	qRT-PCR
hsa-mir-455-5p	TXNL1	PAR-CLIP
hsa-mir-455-5p	QKI	PAR-CLIP
hsa-mir-455-5p	PCLAF	HITS-CLIP
hsa-mir-455-5p	RASSF2	PAR-CLIP
hsa-mir-455-5p	G3BP1	PAR-CLIP
hsa-mir-455-5p	VAV3	HITS-CLIP//PAR-CLIP
hsa-mir-455-5p	IPO7	PAR-CLIP
hsa-mir-455-5p	ZNF460	PAR-CLIP
hsa-mir-455-5p	CCNI	HITS-CLIP
hsa-mir-455-5p	POLI	PAR-CLIP
hsa-mir-455-5p	FKBP9	HITS-CLIP
hsa-mir-455-5p	PLEKHA6	PAR-CLIP
hsa-mir-455-5p	SEPHS1	PAR-CLIP
hsa-mir-455-5p	RAB18	Luciferase reporter assay//qRT-PCR//Western blot
hsa-mir-455-5p	IGSF9B	HITS-CLIP
hsa-mir-455-5p	FBXO28	PAR-CLIP
hsa-mir-455-5p	ARC	PAR-CLIP
hsa-mir-455-5p	MGRN1	PAR-CLIP
hsa-mir-455-5p	NCSTN	ELISA//Luciferase reporter assay
hsa-mir-455-5p	PRKD2	HITS-CLIP

hsa-mir-455-5p	LYPD3	HITS-CLIP
hsa-mir-455-5p	ZNF544	PAR-CLIP
hsa-mir-455-5p	MYLIP	PAR-CLIP
hsa-mir-455-5p	ZNF354C	PAR-CLIP
hsa-mir-455-5p	ZNF117	PAR-CLIP
hsa-mir-455-5p	TRPV2	PAR-CLIP
hsa-mir-455-5p	TRIM33	PAR-CLIP
hsa-mir-455-5p	NUP54	HITS-CLIP//PAR-CLIP
hsa-mir-455-5p	PRR13	HITS-CLIP
hsa-mir-455-5p	DDX4	PAR-CLIP
hsa-mir-455-5p	BNC2	HITS-CLIP
hsa-mir-455-5p	AHI1	PAR-CLIP
hsa-mir-455-5p	PIWIL2	PAR-CLIP
hsa-mir-455-5p	MTPAP	PAR-CLIP
hsa-mir-455-5p	DARS2	HITS-CLIP
hsa-mir-455-5p	DEPDC1B	PAR-CLIP
hsa-mir-455-5p	NUFIP2	PAR-CLIP
hsa-mir-455-5p	FAM160B1	PAR-CLIP
hsa-mir-455-5p	ATP13A3	PAR-CLIP
hsa-mir-455-5p	MOB3B	PAR-CLIP
hsa-mir-455-5p	TMC7	PAR-CLIP
hsa-mir-455-5p	DSN1	PAR-CLIP
hsa-mir-455-5p	WDR26	PAR-CLIP
hsa-mir-455-5p	KLHL15	PAR-CLIP
hsa-mir-455-5p	GSG1	PAR-CLIP
hsa-mir-455-5p	SLC9A7	PAR-CLIP
hsa-mir-455-5p	UBASH3B	PAR-CLIP
hsa-mir-455-5p	ZNF625	PAR-CLIP
hsa-mir-455-5p	LYRM7	HITS-CLIP
hsa-mir-455-5p	NT5C1B	PAR-CLIP
hsa-mir-455-5p	MOGAT1	HITS-CLIP
hsa-mir-455-5p	C15orf40	PAR-CLIP
hsa-mir-455-5p	TMEM170A	PAR-CLIP
hsa-mir-455-5p	TNFAIP8L1	HITS-CLIP
hsa-mir-455-5p	PABPC4L	PAR-CLIP
hsa-mir-455-5p	UBXN2B	PAR-CLIP
hsa-mir-455-5p	LETM2	HITS-CLIP
hsa-mir-455-5p	TRUB1	PAR-CLIP
hsa-mir-455-5p	BCDIN3D	CLASH
hsa-mir-455-5p	TCF23	PAR-CLIP
hsa-mir-455-5p	CAMSAP1	HITS-CLIP
hsa-mir-455-5p	DNAJC18	HITS-CLIP
hsa-mir-455-5p	PATL1	PAR-CLIP
hsa-mir-455-5p	YIPF6	PAR-CLIP
hsa-mir-455-5p	ZNF772	PAR-CLIP
hsa-mir-455-5p	ZBTB34	HITS-CLIP
hsa-mir-455-5p	FAM229B	PAR-CLIP

hsa-mir-455-5p	NT5C1B-RDH14	PAR-CLIP
hsa-mir-31-3p	ACADVL	CLASH
hsa-mir-31-3p	RHOA	qRT-PCR//Western blot
hsa-mir-31-3p	BACH1	HITS-CLIP
hsa-mir-31-3p	CDH13	HITS-CLIP
hsa-mir-31-3p	CRK	PAR-CLIP
hsa-mir-31-3p	CRKL	PAR-CLIP
hsa-mir-31-3p	DCK	PAR-CLIP
hsa-mir-31-3p	E2F2	Luciferase reporter assay
hsa-mir-31-3p	GABRB1	HITS-CLIP
hsa-mir-31-3p	HSPA6	HITS-CLIP//PAR-CLIP
hsa-mir-31-3p	INHBA	CLASH
hsa-mir-31-3p	MCM4	CLASH
hsa-mir-31-3p	NUCB1	HITS-CLIP
hsa-mir-31-3p	PAX6	HITS-CLIP
hsa-mir-31-3p	PDE4D	PAR-CLIP
hsa-mir-31-3p	PPIA	CLASH
hsa-mir-31-3p	PPIC	PAR-CLIP
hsa-mir-31-3p	PPP2R5C	CLASH
hsa-mir-31-3p	RAN	CLASH
hsa-mir-31-3p	SDHA	Luciferase reporter assay//qRT-PCR//Western blot
hsa-mir-31-3p	SP1	PAR-CLIP
hsa-mir-31-3p	TRAF1	PAR-CLIP
hsa-mir-31-3p	XK	CLASH
hsa-mir-31-3p	KIAA0391	PAR-CLIP
hsa-mir-31-3p	SUPT7L	PAR-CLIP
hsa-mir-31-3p	SLC9A6	CLASH
hsa-mir-31-3p	ARL6IP5	CLASH
hsa-mir-31-3p	TXNIP	PAR-CLIP
hsa-mir-31-3p	FRS2	PAR-CLIP
hsa-mir-31-3p	DNAJB4	PAR-CLIP
hsa-mir-31-3p	CBX3	PAR-CLIP
hsa-mir-31-3p	NLGN1	HITS-CLIP
hsa-mir-31-3p	NCBP2	HITS-CLIP
hsa-mir-31-3p	RAB18	PAR-CLIP
hsa-mir-31-3p	FBXL7	PAR-CLIP
hsa-mir-31-3p	PUM2	PAR-CLIP
hsa-mir-31-3p	DICER1	qRT-PCR
hsa-mir-31-3p	MAFF	HITS-CLIP
hsa-mir-31-3p	FBXL5	HITS-CLIP//PAR-CLIP
hsa-mir-31-3p	ago-02	HITS-CLIP//PAR-CLIP
hsa-mir-31-3p	DBR1	CLASH
hsa-mir-31-3p	BRWD1	PAR-CLIP
hsa-mir-31-3p	EPB41L4B	PAR-CLIP
hsa-mir-31-3p	PLEKHB2	PAR-CLIP
hsa-mir-31-3p	BLOC1S4	HITS-CLIP

hsa-mir-31-3p	RBM38	PAR-CLIP
hsa-mir-31-3p	POLR3E	PAR-CLIP
hsa-mir-31-3p	ZNF71	PAR-CLIP
hsa-mir-31-3p	SLC30A5	PAR-CLIP
hsa-mir-31-3p	ZNF614	PAR-CLIP
hsa-mir-31-3p	TNKS2	CLASH
hsa-mir-31-3p	SLC38A1	PAR-CLIP
hsa-mir-31-3p	NECTIN4	Luciferase reporter assay//Western blot
hsa-mir-31-3p	C9orf64	HITS-CLIP//PAR-CLIP
hsa-mir-31-3p	LMNB2	PAR-CLIP
hsa-mir-31-3p	C1orf198	CLASH
hsa-mir-31-3p	PRPF38A	PAR-CLIP
hsa-mir-31-3p	CIPC	CLASH
hsa-mir-31-3p	PNPT1	HITS-CLIP
hsa-mir-31-3p	PAPLN	PAR-CLIP
hsa-mir-31-3p	NUS1	CLASH
hsa-mir-31-3p	CHMP4B	PAR-CLIP
hsa-mir-31-3p	PTPDC1	PAR-CLIP
hsa-mir-31-3p	SPRED1	PAR-CLIP
hsa-mir-31-3p	TET3	HITS-CLIP
hsa-mir-31-3p	SLC16A9	PAR-CLIP
hsa-mir-31-3p	ZNF485	HITS-CLIP//PAR-CLIP
hsa-mir-31-3p	RICTOR	CLASH
hsa-mir-31-3p	RPL7L1	PAR-CLIP
hsa-mir-31-3p	NUP43	PAR-CLIP
hsa-mir-193b-5p	ADD1	PAR-CLIP
hsa-mir-193b-5p	GRK3	HITS-CLIP
hsa-mir-193b-5p	ALDOA	PAR-CLIP
hsa-mir-193b-5p	ART4	HITS-CLIP
hsa-mir-193b-5p	ATP5G1	HITS-CLIP
hsa-mir-193b-5p	BMP7	HITS-CLIP
hsa-mir-193b-5p	BMP8B	PAR-CLIP
hsa-mir-193b-5p	ZFP36L1	PAR-CLIP
hsa-mir-193b-5p	CACNG1	PAR-CLIP
hsa-mir-193b-5p	COL9A2	PAR-CLIP
hsa-mir-193b-5p	CRKL	CLASH
hsa-mir-193b-5p	DNASE2	HITS-CLIP//PAR-CLIP
hsa-mir-193b-5p	SLC26A2	HITS-CLIP
hsa-mir-193b-5p	CLN8	HITS-CLIP
hsa-mir-193b-5p	NR2F6	PAR-CLIP
hsa-mir-193b-5p	FGB	PAR-CLIP
hsa-mir-193b-5p	FXN	PAR-CLIP
hsa-mir-193b-5p	GGCX	PAR-CLIP
hsa-mir-193b-5p	GLA	PAR-CLIP
hsa-mir-193b-5p	GUCA1B	HITS-CLIP
hsa-mir-193b-5p	HOXC8	PAR-CLIP
hsa-mir-193b-5p	IFIT2	Microarray//qRT-PCR

hsa-mir-193b-5p	IFNAR1	HITS-CLIP
hsa-mir-193b-5p	RBPJ	PAR-CLIP
hsa-mir-193b-5p	IPP	HITS-CLIP
hsa-mir-193b-5p	KCNA7	PAR-CLIP
hsa-mir-193b-5p	STMN1	Luciferase reporter assay//qRT-PCR//Western blot
hsa-mir-193b-5p	MGAT5	HITS-CLIP
hsa-mir-193b-5p	ATXN3	HITS-CLIP
hsa-mir-193b-5p	MYO1C	PAR-CLIP
hsa-mir-193b-5p	NDUFV3	PAR-CLIP
hsa-mir-193b-5p	PAK3	HITS-CLIP
hsa-mir-193b-5p	PRKN	PAR-CLIP
hsa-mir-193b-5p	PCYT1A	PAR-CLIP
hsa-mir-193b-5p	ATP8B1	PAR-CLIP
hsa-mir-193b-5p	MAP2K2	PAR-CLIP
hsa-mir-193b-5p	PSMB9	PAR-CLIP
hsa-mir-193b-5p	QSOX1	PAR-CLIP
hsa-mir-193b-5p	PURB	HITS-CLIP
hsa-mir-193b-5p	RAB3B	PAR-CLIP
hsa-mir-193b-5p	MAP4K2	HITS-CLIP
hsa-mir-193b-5p	RAB13	PAR-CLIP
hsa-mir-193b-5p	RAB27A	HITS-CLIP
hsa-mir-193b-5p	RPL4	PAR-CLIP
hsa-mir-193b-5p	RPS19	CLASH
hsa-mir-193b-5p	RRAD	PAR-CLIP
hsa-mir-193b-5p	SLC4A1	PAR-CLIP
hsa-mir-193b-5p	THBS2	HITS-CLIP
hsa-mir-193b-5p	TIAL1	HITS-CLIP
hsa-mir-193b-5p	TMF1	PAR-CLIP
hsa-mir-193b-5p	UGT2B4	PAR-CLIP
hsa-mir-193b-5p	VHL	HITS-CLIP
hsa-mir-193b-5p	ZNF8	PAR-CLIP
hsa-mir-193b-5p	ZNF708	HITS-CLIP
hsa-mir-193b-5p	ZNF24	PAR-CLIP
hsa-mir-193b-5p	ZNF138	PAR-CLIP
hsa-mir-193b-5p	BTG2	PAR-CLIP
hsa-mir-193b-5p	MLF2	PAR-CLIP
hsa-mir-193b-5p	GAN	HITS-CLIP
hsa-mir-193b-5p	CLPP	PAR-CLIP
hsa-mir-193b-5p	CHAF1B	PAR-CLIP
hsa-mir-193b-5p	API5	HITS-CLIP
hsa-mir-193b-5p	DEGS1	PAR-CLIP
hsa-mir-193b-5p	GMPS	PAR-CLIP
hsa-mir-193b-5p	UBE4A	PAR-CLIP
hsa-mir-193b-5p	GSTO1	PAR-CLIP
hsa-mir-193b-5p	AKAP6	HITS-CLIP
hsa-mir-193b-5p	SOCS5	PAR-CLIP
hsa-mir-193b-5p	NUP93	HITS-CLIP

hsa-mir-193b-5p	KIAA0586	HITS-CLIP
hsa-mir-193b-5p	SLC35E2	PAR-CLIP
hsa-mir-193b-5p	CCS	HITS-CLIP
hsa-mir-193b-5p	GNE	HITS-CLIP
hsa-mir-193b-5p	PLIN3	HITS-CLIP
hsa-mir-193b-5p	LYPLA1	HITS-CLIP
hsa-mir-193b-5p	HOXB13	PAR-CLIP
hsa-mir-193b-5p	ANP32B	PAR-CLIP
hsa-mir-193b-5p	PDLIM5	PAR-CLIP
hsa-mir-193b-5p	GMEB1	HITS-CLIP
hsa-mir-193b-5p	SRSF10	PAR-CLIP
hsa-mir-193b-5p	FTCD	HITS-CLIP
hsa-mir-193b-5p	SLC27A4	PAR-CLIP
hsa-mir-193b-5p	RPP14	PAR-CLIP
hsa-mir-193b-5p	RCAN3	PAR-CLIP
hsa-mir-193b-5p	POLR3A	HITS-CLIP
hsa-mir-193b-5p	PHB2	PAR-CLIP
hsa-mir-193b-5p	RHOBTB3	HITS-CLIP
hsa-mir-193b-5p	RTF1	PAR-CLIP
hsa-mir-193b-5p	JADE2	PAR-CLIP
hsa-mir-193b-5p	PUM2	PAR-CLIP
hsa-mir-193b-5p	TMEM245	HITS-CLIP
hsa-mir-193b-5p	TNFAIP8	HITS-CLIP
hsa-mir-193b-5p	BACE2	HITS-CLIP
hsa-mir-193b-5p	HEATR5A	HITS-CLIP
hsa-mir-193b-5p	RSL1D1	PAR-CLIP
hsa-mir-193b-5p	HSPB8	PAR-CLIP
hsa-mir-193b-5p	TNRC6A	PAR-CLIP
hsa-mir-193b-5p	POLL	HITS-CLIP
hsa-mir-193b-5p	TOR2A	PAR-CLIP
hsa-mir-193b-5p	FLVCR1	HITS-CLIP
hsa-mir-193b-5p	NTMT1	HITS-CLIP
hsa-mir-193b-5p	ORMDL2	HITS-CLIP
hsa-mir-193b-5p	PYCARD	PAR-CLIP
hsa-mir-193b-5p	A1CF	PAR-CLIP
hsa-mir-193b-5p	TRAT1	HITS-CLIP
hsa-mir-193b-5p	WDPCP	HITS-CLIP
hsa-mir-193b-5p	ZC2HC1A	HITS-CLIP
hsa-mir-193b-5p	RDH11	PAR-CLIP
hsa-mir-193b-5p	PHF20	HITS-CLIP
hsa-mir-193b-5p	CRIM1	Microarray//qRT-PCR
hsa-mir-193b-5p	TRPV2	PAR-CLIP
hsa-mir-193b-5p	LARS	HITS-CLIP
hsa-mir-193b-5p	GDE1	HITS-CLIP
hsa-mir-193b-5p	FXYD5	PAR-CLIP
hsa-mir-193b-5p	CYCS	HITS-CLIP
hsa-mir-193b-5p	DNAJC10	PAR-CLIP

hsa-mir-193b-5p	SDK2	HITS-CLIP
hsa-mir-193b-5p	GPN2	PAR-CLIP
hsa-mir-193b-5p	PGPEP1	PAR-CLIP
hsa-mir-193b-5p	DPP8	HITS-CLIP
hsa-mir-193b-5p	RBM28	PAR-CLIP
hsa-mir-193b-5p	KIAA1551	PAR-CLIP
hsa-mir-193b-5p	SLC38A7	PAR-CLIP
hsa-mir-193b-5p	MIOX	HITS-CLIP
hsa-mir-193b-5p	IPO9	HITS-CLIP
hsa-mir-193b-5p	ZNF701	HITS-CLIP
hsa-mir-193b-5p	MINDY1	HITS-CLIP
hsa-mir-193b-5p	METTL2B	PAR-CLIP
hsa-mir-193b-5p	FOXJ2	PAR-CLIP
hsa-mir-193b-5p	UTP6	HITS-CLIP
hsa-mir-193b-5p	LMOD3	HITS-CLIP//PAR-CLIP
hsa-mir-193b-5p	ISY1	PAR-CLIP
hsa-mir-193b-5p	MAVS	HITS-CLIP
hsa-mir-193b-5p	TAOK1	HITS-CLIP
hsa-mir-193b-5p	KIAA1456	HITS-CLIP
hsa-mir-193b-5p	ZFP14	PAR-CLIP
hsa-mir-193b-5p	CACNG8	HITS-CLIP
hsa-mir-193b-5p	NPFFR1	PAR-CLIP
hsa-mir-193b-5p	AEN	CLASH
hsa-mir-193b-5p	ZNF747	PAR-CLIP
hsa-mir-193b-5p	CHAC1	PAR-CLIP
hsa-mir-193b-5p	PPDPF	HITS-CLIP
hsa-mir-193b-5p	TTPAL	HITS-CLIP
hsa-mir-193b-5p	NKAP	PAR-CLIP
hsa-mir-193b-5p	CXorf36	PAR-CLIP
hsa-mir-193b-5p	C12orf49	PAR-CLIP
hsa-mir-193b-5p	ZMYM1	HITS-CLIP
hsa-mir-193b-5p	ZNF669	HITS-CLIP
hsa-mir-193b-5p	CPSF7	PAR-CLIP
hsa-mir-193b-5p	SYNPO2L	PAR-CLIP
hsa-mir-193b-5p	ZNF556	HITS-CLIP
hsa-mir-193b-5p	COQ10B	PAR-CLIP
hsa-mir-193b-5p	FAHD1	HITS-CLIP
hsa-mir-193b-5p	KREMEN1	HITS-CLIP
hsa-mir-193b-5p	ZRANB3	PAR-CLIP
hsa-mir-193b-5p	YIPF4	HITS-CLIP
hsa-mir-193b-5p	NOA1	PAR-CLIP
hsa-mir-193b-5p	TNRC18	PAR-CLIP
hsa-mir-193b-5p	MYPN	PAR-CLIP
hsa-mir-193b-5p	ZNF347	PAR-CLIP
hsa-mir-193b-5p	RRP36	HITS-CLIP
hsa-mir-193b-5p	KIR3DX1	HITS-CLIP
hsa-mir-193b-5p	LYRM7	PAR-CLIP

hsa-mir-193b-5p	SLC38A5	HITS-CLIP
hsa-mir-193b-5p	ZBTB47	PAR-CLIP
hsa-mir-193b-5p	ZNF101	HITS-CLIP
hsa-mir-193b-5p	FLYWCH2	HITS-CLIP
hsa-mir-193b-5p	DIS3L	PAR-CLIP
hsa-mir-193b-5p	BORCS7	HITS-CLIP
hsa-mir-193b-5p	SPPL3	HITS-CLIP
hsa-mir-193b-5p	GJD3	PAR-CLIP
hsa-mir-193b-5p	ZNF813	HITS-CLIP
hsa-mir-193b-5p	ZNF491	PAR-CLIP
hsa-mir-193b-5p	ZNF573	PAR-CLIP
hsa-mir-193b-5p	C19orf47	HITS-CLIP
hsa-mir-193b-5p	RNF19B	HITS-CLIP
hsa-mir-193b-5p	ICA1L	HITS-CLIP
hsa-mir-193b-5p	CMBL	PAR-CLIP
hsa-mir-193b-5p	OTUD6A	PAR-CLIP
hsa-mir-193b-5p	SMCR8	HITS-CLIP
hsa-mir-193b-5p	PTGR2	PAR-CLIP
hsa-mir-193b-5p	MANEAL	PAR-CLIP
hsa-mir-193b-5p	TCF23	PAR-CLIP
hsa-mir-193b-5p	GPR155	HITS-CLIP
hsa-mir-193b-5p	PAQR3	HITS-CLIP//PAR-CLIP
hsa-mir-193b-5p	GPR156	HITS-CLIP
hsa-mir-193b-5p	ZNF366	PAR-CLIP
hsa-mir-193b-5p	ZNF384	HITS-CLIP
hsa-mir-193b-5p	THAP8	PAR-CLIP
hsa-mir-193b-5p	DENND6A	HITS-CLIP
hsa-mir-193b-5p	TMEM154	HITS-CLIP//PAR-CLIP
hsa-mir-193b-5p	TMEM192	HITS-CLIP
hsa-mir-193b-5p	C6orf89	HITS-CLIP
hsa-mir-193b-5p	RSBN1L	HITS-CLIP
hsa-mir-193b-5p	FBXL13	PAR-CLIP
hsa-mir-193b-5p	MSRB3	HITS-CLIP
hsa-mir-193b-5p	SLC25A45	PAR-CLIP
hsa-mir-193b-5p	CAVIN1	PAR-CLIP
hsa-mir-193b-5p	GDPD1	PAR-CLIP
hsa-mir-193b-5p	C5orf51	PAR-CLIP
hsa-mir-193b-5p	ZNF677	HITS-CLIP
hsa-mir-193b-5p	MACC1	HITS-CLIP
hsa-mir-193b-5p	GSTK1	PAR-CLIP
hsa-mir-193b-5p	C3orf62	PAR-CLIP
hsa-mir-193b-5p	ZKSCAN4	PAR-CLIP
hsa-mir-193b-5p	ZNF788	PAR-CLIP
hsa-mir-193b-5p	C2orf68	HITS-CLIP
hsa-mir-193b-5p	ZNF793	HITS-CLIP
hsa-mir-193b-5p	HACD4	PAR-CLIP
hsa-mir-193b-5p	TRIM72	PAR-CLIP

hsa-mir-193b-5p	C6orf132	HITS-CLIP
hsa-mir-193b-5p	ANKRD33B	HITS-CLIP
hsa-mir-193b-5p	ZNF878	HITS-CLIP
hsa-mir-193b-5p	ERVMER34-1	HITS-CLIP
hsa-mir-193b-5p	C8orf17	HITS-CLIP
hsa-mir-2355-5p	ABL2	HITS-CLIP//PAR-CLIP
hsa-mir-2355-5p	XIAP	PAR-CLIP
hsa-mir-2355-5p	ZFP36L2	PAR-CLIP
hsa-mir-2355-5p	CACNA1A	HITS-CLIP
hsa-mir-2355-5p	CAPZA1	PAR-CLIP
hsa-mir-2355-5p	CCNF	PAR-CLIP
hsa-mir-2355-5p	CDKN1A	PAR-CLIP
hsa-mir-2355-5p	CDKN1B	PAR-CLIP
hsa-mir-2355-5p	CPM	HITS-CLIP
hsa-mir-2355-5p	CTNND1	PAR-CLIP
hsa-mir-2355-5p	ECE1	PAR-CLIP
hsa-mir-2355-5p	GCNT1	HITS-CLIP
hsa-mir-2355-5p	GRIN2A	HITS-CLIP
hsa-mir-2355-5p	HDGF	PAR-CLIP
hsa-mir-2355-5p	HMGA1	HITS-CLIP
hsa-mir-2355-5p	HNRNPH1	HITS-CLIP
hsa-mir-2355-5p	HOXD9	PAR-CLIP
hsa-mir-2355-5p	HSPA4	HITS-CLIP
hsa-mir-2355-5p	HSPA6	HITS-CLIP
hsa-mir-2355-5p	MCC	PAR-CLIP
hsa-mir-2355-5p	MVK	HITS-CLIP
hsa-mir-2355-5p	OAZ2	HITS-CLIP
hsa-mir-2355-5p	CDK16	PAR-CLIP
hsa-mir-2355-5p	PIGA	PAR-CLIP
hsa-mir-2355-5p	POU6F1	HITS-CLIP
hsa-mir-2355-5p	PPP2R5E	HITS-CLIP
hsa-mir-2355-5p	PURA	HITS-CLIP
hsa-mir-2355-5p	PYCR1	PAR-CLIP
hsa-mir-2355-5p	RPL41	PAR-CLIP
hsa-mir-2355-5p	SRSF2	PAR-CLIP
hsa-mir-2355-5p	SOX12	HITS-CLIP
hsa-mir-2355-5p	SP1	HITS-CLIP
hsa-mir-2355-5p	SPTBN2	HITS-CLIP
hsa-mir-2355-5p	TBX15	PAR-CLIP
hsa-mir-2355-5p	TTR	HITS-CLIP
hsa-mir-2355-5p	VASP	HITS-CLIP
hsa-mir-2355-5p	WNT2B	HITS-CLIP
hsa-mir-2355-5p	ZNF79	HITS-CLIP
hsa-mir-2355-5p	ZNF138	PAR-CLIP
hsa-mir-2355-5p	LUZP1	PAR-CLIP
hsa-mir-2355-5p	PTP4A1	PAR-CLIP
hsa-mir-2355-5p	BSND	HITS-CLIP

hsa-mir-2355-5p	PABPN1	PAR-CLIP
hsa-mir-2355-5p	SMC1A	PAR-CLIP
hsa-mir-2355-5p	SUCLG2	HITS-CLIP
hsa-mir-2355-5p	TRIM24	PAR-CLIP
hsa-mir-2355-5p	ALDH1A2	HITS-CLIP
hsa-mir-2355-5p	GYG2	HITS-CLIP
hsa-mir-2355-5p	TM4SF5	PAR-CLIP
hsa-mir-2355-5p	SYT7	PAR-CLIP
hsa-mir-2355-5p	GPR55	HITS-CLIP
hsa-mir-2355-5p	GOSR1	HITS-CLIP
hsa-mir-2355-5p	PTGES	HITS-CLIP
hsa-mir-2355-5p	CCDC144A	PAR-CLIP
hsa-mir-2355-5p	TMEM63A	PAR-CLIP
hsa-mir-2355-5p	RIMS3	HITS-CLIP
hsa-mir-2355-5p	TSC22D2	HITS-CLIP
hsa-mir-2355-5p	UBAP2L	HITS-CLIP
hsa-mir-2355-5p	FAM13A	HITS-CLIP
hsa-mir-2355-5p	DCAF7	PAR-CLIP
hsa-mir-2355-5p	C1D	PAR-CLIP
hsa-mir-2355-5p	IGF2BP1	HITS-CLIP
hsa-mir-2355-5p	PXMP4	HITS-CLIP
hsa-mir-2355-5p	PHB2	PAR-CLIP
hsa-mir-2355-5p	ZFP30	HITS-CLIP
hsa-mir-2355-5p	AAK1	HITS-CLIP
hsa-mir-2355-5p	CLUAP1	HITS-CLIP
hsa-mir-2355-5p	WAPL	PAR-CLIP
hsa-mir-2355-5p	MRPS27	HITS-CLIP
hsa-mir-2355-5p	RPRD2	PAR-CLIP
hsa-mir-2355-5p	VPS8	HITS-CLIP
hsa-mir-2355-5p	CBX6	PAR-CLIP
hsa-mir-2355-5p	PISD	PAR-CLIP
hsa-mir-2355-5p	GABARAPL3	HITS-CLIP
hsa-mir-2355-5p	THUMPD3	HITS-CLIP
hsa-mir-2355-5p	HINFP	HITS-CLIP
hsa-mir-2355-5p	GMEB2	PAR-CLIP
hsa-mir-2355-5p	DKK3	PAR-CLIP
hsa-mir-2355-5p	ZNF638	HITS-CLIP
hsa-mir-2355-5p	PARVB	PAR-CLIP
hsa-mir-2355-5p	TFCP2L1	HITS-CLIP
hsa-mir-2355-5p	NOP53	PAR-CLIP
hsa-mir-2355-5p	SOCS7	PAR-CLIP
hsa-mir-2355-5p	MRPL4	HITS-CLIP
hsa-mir-2355-5p	APH1A	PAR-CLIP
hsa-mir-2355-5p	MRPL30	PAR-CLIP
hsa-mir-2355-5p	FAM8A1	PAR-CLIP
hsa-mir-2355-5p	UFM1	HITS-CLIP
hsa-mir-2355-5p	ERRFI1	PAR-CLIP

hsa-mir-2355-5p	FBXL19	PAR-CLIP
hsa-mir-2355-5p	FNBP1L	HITS-CLIP
hsa-mir-2355-5p	DNAJC28	HITS-CLIP
hsa-mir-2355-5p	CDCA4	HITS-CLIP
hsa-mir-2355-5p	RCBTB1	PAR-CLIP
hsa-mir-2355-5p	LRRC1	PAR-CLIP
hsa-mir-2355-5p	NAGK	HITS-CLIP
hsa-mir-2355-5p	SLC30A6	HITS-CLIP
hsa-mir-2355-5p	TENM3	HITS-CLIP
hsa-mir-2355-5p	NAXD	PAR-CLIP
hsa-mir-2355-5p	ZNF415	PAR-CLIP
hsa-mir-2355-5p	FAM212B	HITS-CLIP
hsa-mir-2355-5p	SAR1A	HITS-CLIP//PAR-CLIP
hsa-mir-2355-5p	EMC7	HITS-CLIP
hsa-mir-2355-5p	ATXN7L3	HITS-CLIP
hsa-mir-2355-5p	TOMM22	HITS-CLIP
hsa-mir-2355-5p	IGSF9	PAR-CLIP
hsa-mir-2355-5p	TAOK1	PAR-CLIP
hsa-mir-2355-5p	WDFY1	PAR-CLIP
hsa-mir-2355-5p	HIVEP3	PAR-CLIP
hsa-mir-2355-5p	BACH2	HITS-CLIP
hsa-mir-2355-5p	FAM217B	HITS-CLIP
hsa-mir-2355-5p	SUSD1	HITS-CLIP
hsa-mir-2355-5p	INF2	HITS-CLIP
hsa-mir-2355-5p	GIGYF1	PAR-CLIP
hsa-mir-2355-5p	C16orf58	PAR-CLIP
hsa-mir-2355-5p	ZNF649	PAR-CLIP
hsa-mir-2355-5p	TMEM109	HITS-CLIP
hsa-mir-2355-5p	OGFOD3	HITS-CLIP
hsa-mir-2355-5p	GTDC1	HITS-CLIP
hsa-mir-2355-5p	ZNF385D	HITS-CLIP
hsa-mir-2355-5p	ZMYM1	HITS-CLIP//PAR-CLIP
hsa-mir-2355-5p	ZSCAN16	HITS-CLIP
hsa-mir-2355-5p	TTYH3	HITS-CLIP
hsa-mir-2355-5p	KCNH6	HITS-CLIP
hsa-mir-2355-5p	SPRY4	HITS-CLIP
hsa-mir-2355-5p	AMMECR1L	HITS-CLIP
hsa-mir-2355-5p	ZNF394	PAR-CLIP
hsa-mir-2355-5p	TMEM246	HITS-CLIP
hsa-mir-2355-5p	CMSS1	PAR-CLIP
hsa-mir-2355-5p	FRMPD3	HITS-CLIP
hsa-mir-2355-5p	NEURL4	PAR-CLIP
hsa-mir-2355-5p	EBPL	HITS-CLIP
hsa-mir-2355-5p	PPP1R9B	PAR-CLIP
hsa-mir-2355-5p	C9orf3	PAR-CLIP
hsa-mir-2355-5p	ARHGAP19	HITS-CLIP
hsa-mir-2355-5p	DCLK3	HITS-CLIP

hsa-mir-2355-5p	MIDN	HITS-CLIP
hsa-mir-2355-5p	ZNF625	PAR-CLIP
hsa-mir-2355-5p	SLC25A46	HITS-CLIP
hsa-mir-2355-5p	SLC39A13	HITS-CLIP
hsa-mir-2355-5p	R3HDM4	PAR-CLIP
hsa-mir-2355-5p	NLRP12	HITS-CLIP
hsa-mir-2355-5p	MYOZ3	HITS-CLIP
hsa-mir-2355-5p	MEX3A	HITS-CLIP
hsa-mir-2355-5p	ASB16	HITS-CLIP
hsa-mir-2355-5p	CHST14	PAR-CLIP
hsa-mir-2355-5p	TOP1MT	PAR-CLIP
hsa-mir-2355-5p	GSTO2	HITS-CLIP
hsa-mir-2355-5p	GJD3	HITS-CLIP
hsa-mir-2355-5p	MFSD12	HITS-CLIP
hsa-mir-2355-5p	RNF19B	HITS-CLIP
hsa-mir-2355-5p	ARHGEF19	HITS-CLIP
hsa-mir-2355-5p	BBS5	HITS-CLIP
hsa-mir-2355-5p	NDUFAF6	HITS-CLIP
hsa-mir-2355-5p	ASB6	PAR-CLIP
hsa-mir-2355-5p	SIRPA	HITS-CLIP
hsa-mir-2355-5p	PRICKLE1	PAR-CLIP
hsa-mir-2355-5p	PTGR2	HITS-CLIP
hsa-mir-2355-5p	KCTD11	HITS-CLIP
hsa-mir-2355-5p	SIX5	HITS-CLIP
hsa-mir-2355-5p	C2orf15	PAR-CLIP
hsa-mir-2355-5p	ROPN1B	PAR-CLIP
hsa-mir-2355-5p	CXorf38	HITS-CLIP
hsa-mir-2355-5p	ZFP1	PAR-CLIP
hsa-mir-2355-5p	ZNF791	PAR-CLIP
hsa-mir-2355-5p	ADAMTS17	HITS-CLIP
hsa-mir-2355-5p	ZNF525	PAR-CLIP
hsa-mir-2355-5p	DOK6	HITS-CLIP
hsa-mir-2355-5p	NT5DC1	HITS-CLIP
hsa-mir-2355-5p	RALGAPA1	HITS-CLIP
hsa-mir-2355-5p	ST6GALNAC3	PAR-CLIP
hsa-mir-2355-5p	ZNF740	HITS-CLIP
hsa-mir-2355-5p	NEK8	HITS-CLIP
hsa-mir-2355-5p	ZNF860	HITS-CLIP
hsa-mir-2355-5p	FAM71F2	HITS-CLIP
hsa-mir-2355-5p	ANKRD36	HITS-CLIP
hsa-mir-2355-5p	ZNF788	PAR-CLIP
hsa-mir-2355-5p	ACBD7	PAR-CLIP
hsa-mir-2355-5p	LURAP1	HITS-CLIP
hsa-mir-2355-5p	ZNF704	PAR-CLIP
hsa-mir-2355-5p	SMIM15	HITS-CLIP
hsa-mir-2355-5p	TMEM78	HITS-CLIP
hsa-mir-2355-5p	ZNF286B	HITS-CLIP

hsa-mir-2355-5p	POM121C	PAR-CLIP
hsa-mir-2355-5p	PPP5D1	PAR-CLIP

Supplementary table 3

List of primers for gene expression analysis

**Gene**	**Sequence**
TMPRSS2	Fw: ACACCAGCCATGATCTGTGC
	Rv: CAGAGGCCCTCCACTGTCA
ACE2	Fw: GGACCCAGGAAATGTTCAGA
	Rv: GGCTGCAGAAAGTGACATGA
GAPDH	Fw: GAGTCAACGGATTTGTCGT
	Rw: GACAAGCTTCCCGTTCTCAG

List of Taqman assays

**ID (gene)**
Hs02800695_m1 (HPRT1)
Hs00154614_m1 (CSTF2)
Hs00961622_m1 (IL10)
Hs00989291_m1 (IFNγ)
Hs99999905_m1 (GAPDH)
002113 (hsa-miR-31-3p)
001048 (hsa-miR-503-5p)
001048 (hsa-miR-503-5p)
001006 (hsa-rnu48)

Supplementary figure 1. (a) Box-plot analysis representing ACE2 gene expression levels in tumoral HNSCC TCGA samples according to the gender (female or male). (b) Box-plot analysis representing TMPRSS2 gene expression levels in non-tumorous (N) and tumor (T) tissues of oral cavity from the HNSCC TCGA dataset.

Supplementary figure 2. qRT-PCR analysis of ACE2 expression levels in Cal-27 and Detroit-562 cell lines upon silencing of p53 (sip53) or YAP (siYAP) or MYC (siMYC) compared to silencing scramble (value=1).

Supplementary figure 3. List of genes for MYC (a) and immune (b) signatures.

Supplementary figure 4. Graphs showing the correlation (Spearman coefficients) between indicated miRNAs and TMPRSS2 in the TCGA LUSC (a) and LUAD (b) datasets.

Supplementary figure 5. Box-plot analysis representing expression levels of miRNAs predicted to target TMPRSS2 in tumoral HNSCC TCGA samples according to TP53 status

Supplementary figure 6. Box-plot analysis representing expression levels of miRNAs predicted to target TMPRSS2 in tumoral HNSCC TCGA samples according to HPV status (a), sex (b) and tumor site (c).

Supplementary table 1. miRNA\TMPRSS2 predicted interactions by miRWalk.

Supplementary table 2. List of experimental validated miRNA-target interactions using miRNet.

Supplementary table 3. List of primers and probes used for qRT-PCR.

Dear Dr. Castelli,
On behalf of all the authors I wish to thank you for proving us with the opportunity to revise our manuscript. We found the comments of the reviewers very insightful and helpful and by addressing them we believe the manuscript has been strongly improved.
Listed below is our point-by-point response to the specific comments of both reviewers:

Reviewer #1:

- The authors proposed a six miRNAs signature negative correlated to their target TMPRSS2 in HNSCC. Since they showed that TMPRSS2 is altered in HNSCC and lung cancer it could be interesting to evaluate whether the association miRNAs\TMPRSS2 is confirmed also in lung cancer.

  We did not include the requested correlations because the manuscript was main focused on HNSCC. However, we did observe that miR-193b-5p and e miR-193b-3p resulted to be inversely correlated to TMPRSS2 both in LUAD as LUSC, miR-31-3p resulted to be inversely correlated to TMPRSS2 in LUAD, miR-503-5p and miR-2355-5p resulted to be weakly inversely correlated to TMPRSS2 in LUAD. We added the information in the text (pg 9, line 44-50) and added the results as new supplementary figure 4.

- The authors showed a significant modulation of the miRNAs in patients with mutant P53. Is there any differences among missense mutation and other type of mutation in HNSCC?

  Only miR-31-3p resulted to be slightly increased in patients carrying TP53 missense mutations compared to other TP53 mutations.

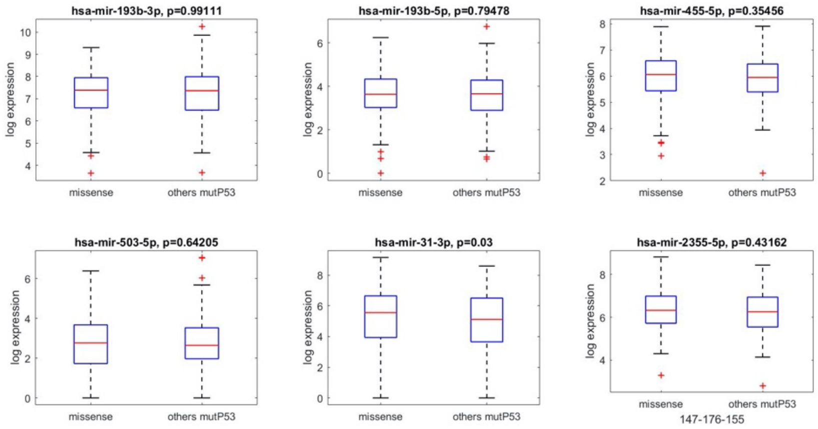

- How does the miRNAs signature correlate with HPV status, gender or tumor site (OC, larynx, pharynx)?

  We observed a number of different correlations between the miR signature, or part of it, and HPV status, gender and tumor site. As matter of fact, miR-31-3p, miR-193b-5p, miR-193b-3p, miR-455-5p and miR-2355-5p resulted to be downregulated in HPV^+ patients while the same miRs were upregulated in female patients compared to male ones. Finally, miR-193b-5p, miR-193b-3p, miR-455-5p, miR-503-5p and miR-2355-5p resulted to be significantly deregulated according to tumor site (new supplementary figure 6).

Reviewer #2

To strengthen the mechanism the authors should address the following points:

- Figure 2F: The authors should verify if TMPRSS2 and ACE2 expression could be regulated by wild-type p53 also by in vitro experiments (ectopic expression of p53 or si-p53 in wt-p53 head-neck cell line).

To address the specific comment raised by the reviewer we assessed the expression of both TMPRSS2 and ACE2 in wt-p53 head and neck cell line, HN091 upon depletion of wt-p53 protein expression. We found that while the expression of TMPRSS2 was unchanged that of ACE2 was slightly modulated (Suppl. Fig. 2b-c). These findings strength further the specificity of mutant p53 protein to modulate aberrantly TMPRSS2 expression in HNSCC.

- What is the status of p53 in the tumor of the COVID-19 patient analyzed?

To address this insightful comment we performed two complementary analyses.

1. The sequence of the entire coding region of TP53 revealed a mutation in the codon 524 (G>A) which led to the production of mutant p53-R175H protein (Fig. 6d).
2. Immunohystochemical analysis revealed intense and diffuse p53 staining as for mutant p53 proteins whose half-life is strongly increased (Fig. 6e).
   In aggregate these findings confirm the mutated status of TP53 gene in the analyzed COVID-19 patient.

- The authors should show, at least for miR-31-3p and miR-503-5p, that their inhibition increases TMPRSS2 expression in HNSCC cell lines.

- Is the expression of miR-31-3p and miR-503-5p dependent on mutant p53 in the CAL-27 and Detroit cell lines?

Both comments are of importance to detail mechanistically the role of specific microRNAs in the regulation of TMPRSS2. This will be the main scope of a future manuscript in which we will aim to dissect and validate molecularly the role of microRNAs in blunting TMPRSS2 activity in HNSCC thereby leading to resistance to SARS-CoV-2 infection.

We strongly hope that the revised version of the manuscript will meet your scientific interest and will be suitable for publication in JEECR.

With best regards

Giovanni Blandino MD
